# Unravelling the Functional Diversity of Type III Polyketide Synthases in Fungi

**DOI:** 10.1002/anie.202514786

**Published:** 2025-09-04

**Authors:** Nika Sokolova, Stepan S. Denisov, Thomas Hackl, Kristina Haslinger

**Affiliations:** ^1^ Department of Chemical and Pharmaceutical Biology University of Groningen Antonius Deusinglaan 1 Groningen 9713AV The Netherlands; ^2^ Institute of Biological Chemistry University of Vienna Währinger Straße 38 Vienna 1090 Austria; ^3^ Groningen Institute for Evolutionary Life Sciences University of Groningen Nijenborgh 7 Groningen 9747 AG The Netherlands

**Keywords:** Biocatalysis, Fungi, Genome mining, Machine learning, Polyketides

## Abstract

Type III polyketide synthases (T3PKSs) are enzymes that produce diverse compounds of ecological and clinical importance. While well‐studied in plants, only a handful of T3PKSs from fungi have been characterised to date. Here, we developed a comprehensive workflow for kingdom‐wide characterisation of T3PKSs. Using publicly available genomes, we mined more than 1000 putative enzymes and analysed their active site architecture and genomic neighbourhood. From there, we selected 37 representative PKS candidates for cell‐free expression and prototyping with a diverse set of Coenzyme A activated substrates, revealing unique patterns in substrate and cyclisation specificity, as well as the preferred number of malonyl‐Coenzyme A extensions. Using the 341 enzyme‐substrate pairs generated in this study, we trained a machine learning model to predict T3PKS substrate specificity and experimentally validated it with an extended panel of non‐natural substrates. In addition, we applied the model to identify two more promiscuous T3PKSs from fungi. We anticipate that the ML model will be useful for in silico screening of T3PKSs, while the insight into the product scope of these enzymes offers interesting starting points for further exploration.

## Graphical abstract

Type III polyketide synthases (T3PKSs) produce diverse compounds of ecological and clinical importance. Here, the activity of 37 fungal T3PKSs was profiled, revealing unexpected products and generating several pharmaceutical precursors. The machine learning model trained on the activity data accurately extrapolates the predictions to other enzymes and substrates, facilitating the development of biocatalytic routes towards (un)natural polyketides.

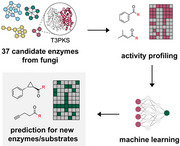

## Introduction

The production of bulk and fine chemicals relies heavily on fossil fuels and rare earth catalysts. Biocatalysis, capitalising on renewable starting materials and enzyme catalysts, may shift the paradigm towards greener synthesis of drugs^[^ ^1^ ^]^ industrial chemicals^[^ ^2^ ^]^ and polymers.^[^ ^3^ ^]^ Despite the growing industrial interest in enzymatic processes, the wider use of biocatalysis is fundamentally hindered by our ability to select enzymes for reactions and substrates of interest.^[^ ^4^, ^5^ ^]^ To be able to compete with the organic synthesis toolbox refined over several centuries, biocatalysis needs more promiscuous enzymes that are active on non‐native substrates.^[^ ^6^ ^]^

**Figure 1:**
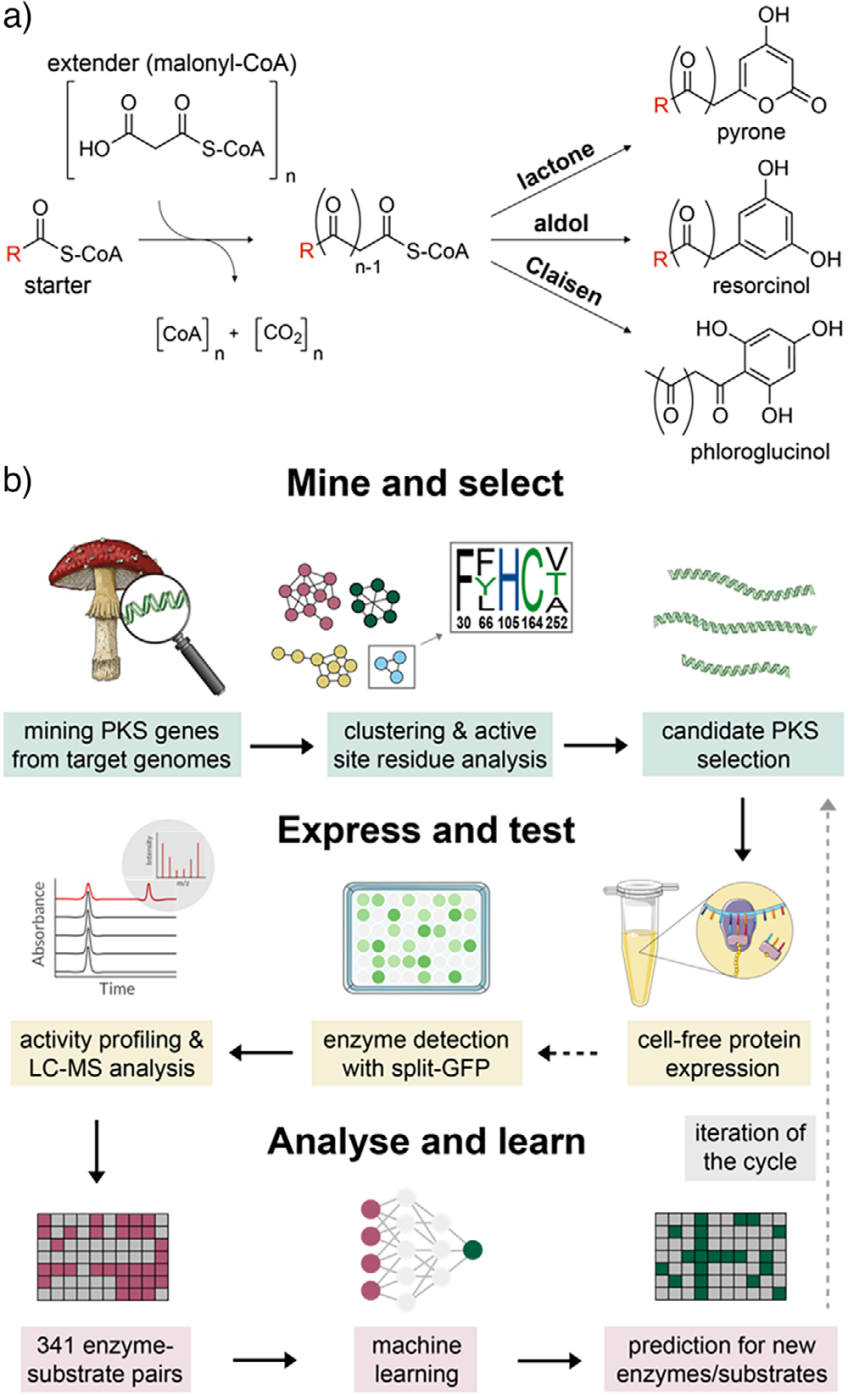
Scope of this study. a) Typical reactions catalysed by T3PKSs. Iterative decarboxylation of extender units (usually malonyl‐CoA) and condensation with the starter unit (variable). Cyclisation of the linear polyketide yielding pyrone (lactone), resorcinol (aldol), or phloroglucinol (Claisen) product. b) Workflow for kingdom‐wide characterisation of T3PKS. Dotted arrows represent optional steps.

Enzymes catalysing the formation of molecular scaffolds that are commonly found in marketed drugs are particularly interesting.^[^ ^7^ ^]^ One example are type III polyketide synthases (T3PKSs) giving rise to distinct polyketide scaffolds (Figure 1a). These enzymes use coenzyme A (CoA)‐bound substrates for iterative decarboxylation, elongation, cyclisation, and aromatisation—all within a single active site—with variable number of elongations and three distinct cyclisation mechanisms.^[^ ^8^ ^]^ Thanks to their relaxed substrate specificity, broad product range, and structural simplicity, T3PKSs comprise a rich pool for the selection and evolution of powerful biocatalysts.

T3PKSs are best studied in plants, where they synthesise more than 10 different groups of natural products, including medicinally relevant cannabinoid, quinolone, and tropane alkaloid scaffolds.^[^ ^9^ ^]^ In bacteria, T3PKSs synthesise naphthalene pigments, spore germination inhibitors, and phenolic lipids required for cyst formation.^[^ ^10^ ^]^ The first fungal representative, 2‐oxoalkylresorcylic acid synthase from *Neurospora crassa*, was characterised in 2007.^[^ ^11^ ^]^ Since then, several more T3PKSs were cloned from filamentous fungi^[^ ^12^, ^13^, ^14^, ^15^, ^16^, ^17^, ^18^, ^19^, ^20^, ^21^ ^]^ and yeast,^[^ ^22^ ^]^ and genome mining projects hint at the presence of many more.^[^ ^23^ ^]^ Most fungal T3PKSs can synthesise tri‐, tetra‐ or pentaketide pyrones and/or resorcinols from a range of fatty acyl‐CoA substrates in vitro but lack activity on ring‐type substrates typical for plant T3PKSs.^[^ ^8^ ^]^ However, reports on fungal T3PKSs remain somewhat anecdotal and our understanding of their functional diversity and native functions is lagging compared to their plant and bacterial counterparts.

Here, we devised a comprehensive workflow for the characterisation of fungal T3PKSs based on computational and experimental methods (Figure 1b). We closely examined the entire fungal T3PKS sequence space and selected 37 representative candidates for cell‐free expression and experimental characterisation. We then mapped the activity landscape of candidate T3PKSs using a panel of CoA‐bound substrates, revealing unexpected products and highlighting enzymes worth further investigation. With this unique set of enzyme‐substrate pairs at hand, we trained and experimentally validated the first machine learning model for T3PKS substrate specificity prediction. We also applied machine learning to propose residues important for substrate specificity. Lastly, we examined the genomic context of these enzymes to derive hypotheses on their elusive natural functions in fungi.

## Results and Discussion

### Kingdom‐wide Genome Mining Suggests Functionally Diverse T3PKSs

First, we set out to explore the prevalence and diversity of T3PKSs in the fungal kingdom. To mine for enzyme candidates, we collected sequences of experimentally characterised T3PKSs from plants and fungi (Supporting File 1) and used them to query 2096 fungal genomes (on 2021–12–22) available in the JGI MycoCosm database.^[^ ^24^ ^]^ A total of 1148 putative T3PKSs were detected in 806 fungal strains (38% of all included genomes). Of these, 582 carried a single sequence and 224 had between 2 and 7 copies of T3PKS genes (Supporting File 2). In parallel, we also retrieved all putative T3PKS sequences from the InterPro database^[^ ^25^ ^]^ (domain IPR011141), since we had noticed that the MycoCosm and InterPro databases are not mutually redundant. The merged and dereplicated dataset contained 1640 sequences (Supporting File 3).

**Figure 2:**
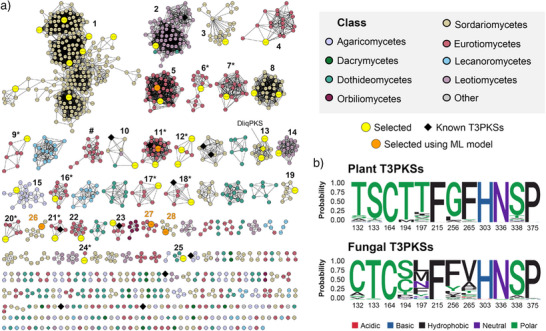
Clustering and active site residue analysis of fungal T3PKSs. a) Sequence similarity network of the mined enzyme candidates at 80% sequence identity cutoff. Each circle is a representative node grouping protein sequences with >95% sequence identity. Diamond‐shaped nodes represent enzymes with literature precedent. Clusters from which sequences were selected for experimental characterisation are numbered. Clusters composed exclusively of sequences from *Aspergillus* are marked with an asterisk. The only major cluster where the catalytic triad is not conserved is marked with # (amino acid substitutions: H303D and N336S). b) Comparison of the active site residues between plant T3PKSs (184 reviewed sequences from InterPro) and the mined fungal T3PKSs. The numbering of the residues corresponds to that in MsCHS (model chalcone synthase from *Medicago sativa*). The active site residue logo was generated using the R package ggseqlogo.^[^ ^29^ ^]^

To identify potential functional groups of fungal T3PKSs, we constructed a sequence similarity network of the mined T3PKSs using the EFI‐EST webtool^[^ ^26^ ^]^ (Supporting File 4). A sequence identity cutoff of around 57% resulted in cluster separation reflecting the phylogenetic clades defined in a recent evolutionary analysis of fungal T3PKSs^[^ ^23^ ^]^ (Figure S1). However, we further increased the cutoff to 80% (Figure 2a) in view of literature evidence that plant T3PKSs can employ distinct cyclisation mechanisms despite sharing 74% sequence identity.^[^ ^27^ ^]^ Even at such a high cutoff, the clusters were not exclusively monogeneric, and cluster 10 contained sequences from three different fungal classes. Previously characterised T3PKSs localised to smaller clusters on the network or appeared as singletons, suggesting that they may not be representative of the typical fungal T3PKSs. As commonly observed with biosynthetic genes in fungi,^[^ ^28^ ^]^ sequences mined from *Aspergillus* separated into more than 10 clusters in the network, hinting at distinct functional groups of T3PKSs in this genus.

**Figure 3:**
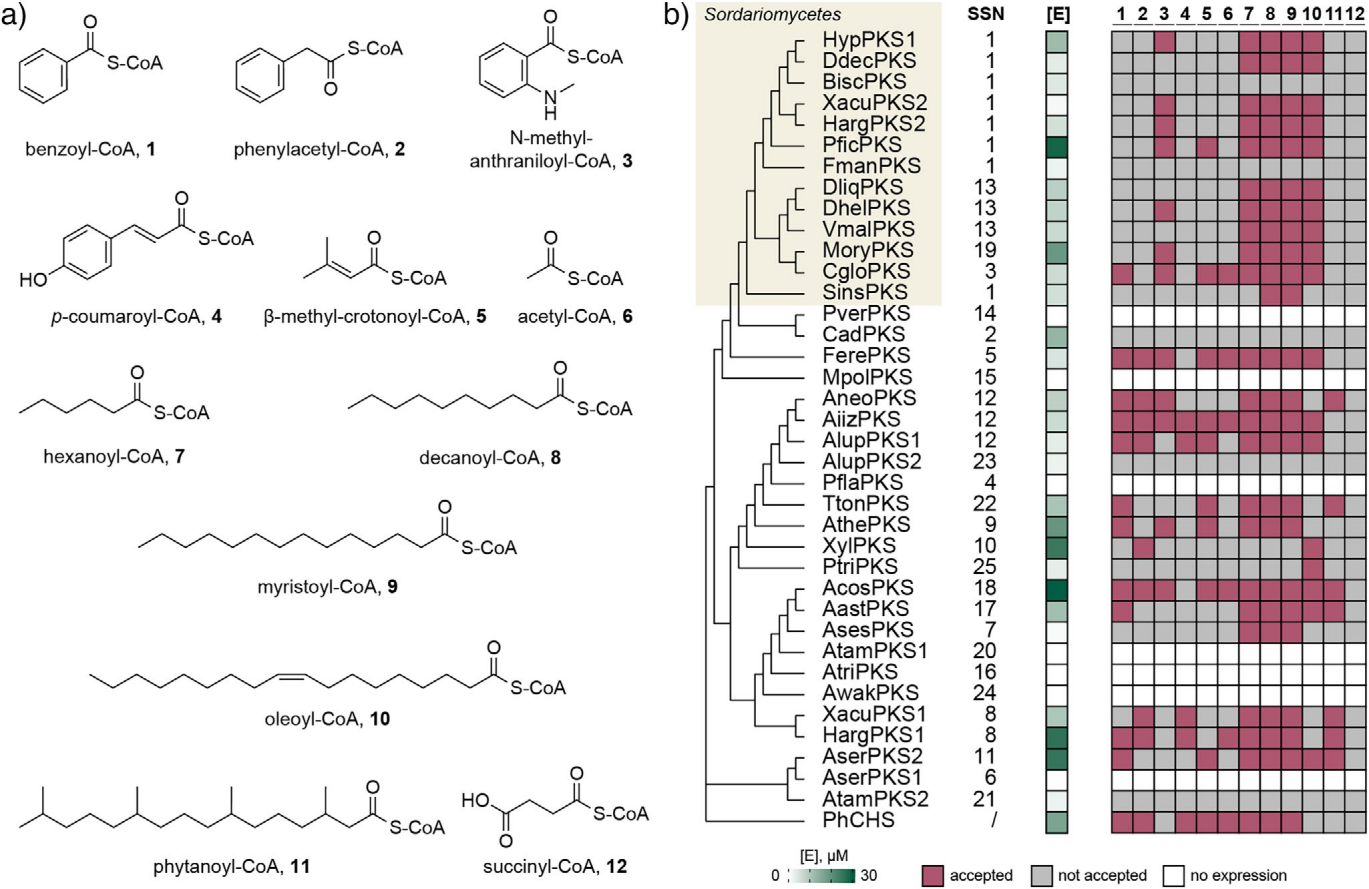
Activity profiling of the cell‐free expressed T3PKSs. a) Panel of CoA thioesters **1**–**12** used as substrates for activity profiling. b) Maximum‐likelihood phylogenetic tree based on the multiple sequence alignment of 37 fungal T3PKSs (outgroup: PhCHS) combined with a heatmap of enzyme concentrations in the cell‐free lysate and a binary representation of enzyme activity in cell‐free lysates supplemented with substrates **1**–**12** and malonyl‐CoA. The “SSN” column specifies the corresponding cluster in the sequence similarity network. Enzyme concentrations [E] (0 to 30 µM) were determined with a split‐GFP complementation assay.

Within each cluster, we analysed the active site residues predicted based on well‐studied plant T3PKS (Figure 2b). The catalytic cysteine (residue 164 in MsCHS, the model chalcone synthase from *Medicago sativa* ^[^ ^30^ ^]^) is conserved across all fungal T3PKSs, while the other residues of the catalytic triad, H303 and N336, are conserved in all but one major cluster. However, one of the residues critical for substrate recognition, F265,^[^ ^30^ ^]^ is variable in fungal T3PKSs (Figure 3b). The F265V substitution, observed in half of the sequences, is also characteristic of divergent plant T3PKSs that produce quinolone and acridone alkaloids from anthranilic acid derivatives.^[^ ^31^ ^]^ The conserved G256 of the substrate‐binding pocket, replaced with leucine in some divergent plant T3PKSs,^[^ ^32^ ^]^ is substituted with bulky aromatic residues in most fungal enzymes. In addition, position 197, which is known to influence substrate^[^ ^33^ ^]^ and product specificity^[^ ^27^ ^]^ in plant chalcone synthases, is variable in fungal enzymes and comprises mostly hydrophobic residues. Overall, the observed variations in the active site residues suggested potential functional differences among fungal T3PKS enzymes.

We then proceeded to sample sequences for experimental characterisation. During selection, we aimed to capture the taxonomic and ecological diversity of the host organisms, as well as the putative functional diversity hinted at by the active site composition. We also oversampled cluster 1 due to its large size and apparent separation into several subclusters reflecting the taxonomic orders of the donor organisms (Figure S2). When selecting sequences within each cluster, we prioritised those with active site residues corresponding to the cluster consensus and those stemming from highly contiguous, high‐quality genome assemblies. Our positive controls included a chalcone synthase from *Petunia hybrida* (PhCHS, UniProt: P08894) and a reported fungal chalcone synthase from *Diaporthe liquidambaris* (DliqPKS, UniProt: A0A7T8G346).^[^ ^34^ ^]^ In total, we selected 37 fungal genes for experimental characterisation (Table S1, Supporting File 5).

### Activity Profiling of the Cell‐Free Expressed T3PKSs

We then turned to functional characterisation of the candidate enzymes. To facilitate the express‐test workflow, we used the recently established myTXTL cell‐free expression platform from linear DNA templates.^[^ ^35^ ^]^ We designed each DNA fragment to contain the codons for split‐GFP (green fluorescent protein) and a hexahistidine tag in‐frame with the 3′ end of the target gene. The split‐GFP tag was used to quantify the expression levels of the target proteins via split‐GFP complementation assay.^[^ ^36^ ^]^ We first verified the express‐test workflow using our positive control enzyme PhCHS. The myTXTL lysate containing 0.78 mg/mL cell‐free expressed PhCHS successfully converted coumaroyl‐CoA and malonyl‐CoA to naringenin chalcone, a part of which then spontaneously cyclised to naringenin (Figure S3).

Next, we collected a panel of substrates for enzyme activity profiling (Figure 3a). During selection, we attempted to capture the diversity of known T3PKS substrates across all phylogenetic groups. Thus, in addition to **4**, which is the canonical substrate of plant chalcone synthases, we included substrates of other plant T3PKSs such as benzophenone and quinolone synthases (**1** and **3**, respectively). Since previously characterised fungal and bacterial T3PKSs tend to prefer aliphatic over aromatic substrates,^[^ ^8^ ^]^ we chose a selection of fatty acyl‐CoA substrates of different chain length, level of branching and degree of unsaturation (**6**–**10**). Finally, we picked several more acyl‐CoA thioesters that, to the best of our knowledge, were never tested with T3PKSs before (**11**–**12**).

We screened the resulting panel of 12 substrates with malonyl‐CoA as a co‐substrate against 38 cell‐free expressed enzymes and analysed the products with liquid chromatography–coupled mass spectrometry (LC–MS). An enzyme was deemed active if we detected one or several new peaks with *m/z* values consistent with the elongation of the carbon chain with up to six CH_2_CO units followed by cyclisation. We also cross‐compared reactions with the same enzyme but different substrates to eliminate any possible background peaks that show up in multiple samples. Based on the results of the split‐GFP assay, 31 enzymes were expressed at detectable levels (Figure 3b). Of these, 26 enzymes were active on at least one substrate. The most promiscuous enzyme, AiizPKS, accepted 10 out of 12 substrates, while the plant enzyme PhCHS accepted 8. The reported fungal chalcone synthase DliqPKS did not produce naringenin chalcone from coumaroyl‐CoA under our assay conditions but was active on fatty acyl substrates **6–10**.

**12** was the only substrate not accepted by any enzyme, possibly due to electronic effects, or because it was diverted to other reactions in the cell‐free lysate. The most broadly accepted substrates in the panel were **8** and **9**, for which 24 out of 26 active enzymes yielded product, followed by **6** (23 enzymes) and **10** (18 enzymes). We observed some specificity for the fatty acyl‐CoA substrates **7–10** in the clade consisting of sequences from *Sordariomycetes* (Figure 3b). The ability to accept aromatic substrates did not correlate with the phylogeny of the enzymes seen in the multiple sequence alignment or the SSN and was scattered across the sequence space. Among the pairs of enzymes that originate from the same organism, in only two both enzymes were active (XacuPKS1 and 2, and HargPKS1 and 2), and the activity profiles within the pairs differed in the ability to accept aromatic and highly branched substrates.

### Machine Learning Can Predict Substrate Specificity of Fungal T3PKSs

**Figure 4:**
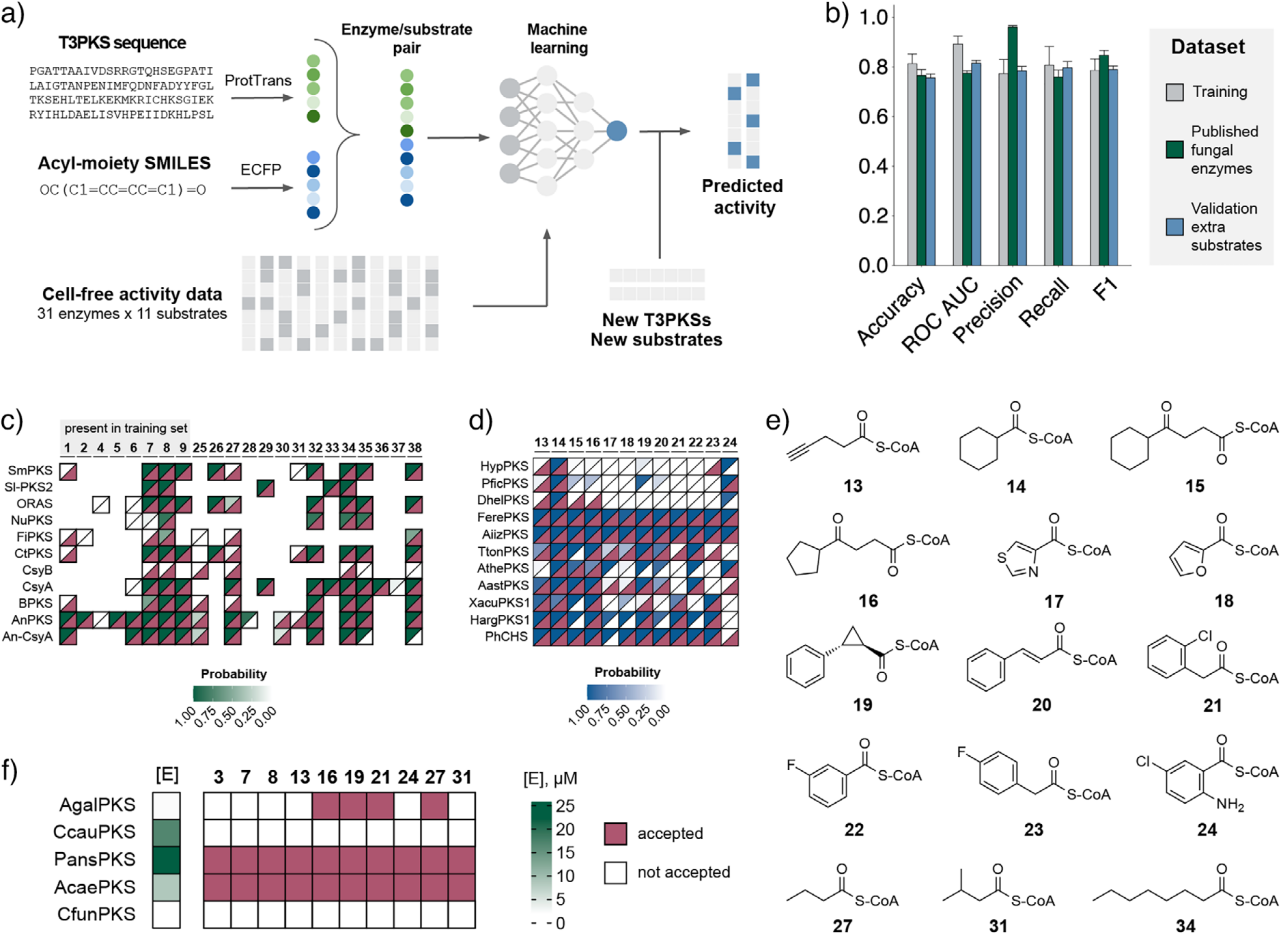
Machine learning model for T3PKS substrate specificity prediction. a) Workflow used to train the predictive machine learning model in this study. b) Performance metrics of the Multilayer Perceptron model for different datasets. The model was trained on the cell‐free activity profiling dataset with ProtTrans‐X5/MACCS Keys feature vectors; error bars indicate standard deviation. c) Heatmap of the predicted versus published substrate specificity for 11 previously characterised T3PKSs from fungi (dataset “Published fungal enzymes”). The activity data were curated from literature (Table S2). The structures of substrates **25**–**38** is depicted in Figure S6. Substrates that were present in the training dataset of our model are highlighted. d) Heatmap of the predicted versus observed substrate specificity for 10 fungal T3PKSs with non‐natural substrates **13**–**24** (dataset “Validation extra substrates”). e) Structures of substrates **13**–**24, 27** and **31** used for experimental validation of the model. f) The observed substrate specificity of 5 new fungal T3PKSs selected using the ML model. All prediction values are averaged from 100 model runs with a random seed. Pink: substrate accepted, white: not accepted.

Our activity profiling showed that the substrate specificity varied even across enzymes from closely related organisms and could not be predicted with human intelligence from phylogeny alone. This led us to hypothesise that machine learning might capture the complex patterns of substrate preference more efficiently. Thus, we used the binary activity data of 31 expressed enzymes with 11 substrates (excluding **12**, which was not accepted by any of the enzymes) to train a machine learning model for substrate specificity prediction in fungal T3PKSs (Figure 4a). In total, 341 enzyme/substrate pairs were included in the data set, of which 146 (43%) were active. Enzymes were represented by calculating and averaging their per‐residue embeddings whereas substrate features were represented by extended‐connectivity fingerprints (ECFPs). The final representation of each enzyme/substrate pair was obtained by concatenating enzyme and substrate features.

We assessed three machine learning algorithms—Decision tree, Random Forest, and feedforward neural network Multilayer Perceptron (MLP)—for binary classification of enzyme activity with three different enzyme/substrate representations (Figure S4). The model accuracy and F1 scores across all tested combinations averaged at 79% and 0.764, respectively. The best‐performing combinations were the MLP model with ProtTrans‐X5/Morgan fingerprints (accuracy 82 ± 4%, F1 score 0.8 ± 0.05) and ProtTrans‐X5/MACCS Keys representations (accuracy 81 ± 4%, F1 score 0.79 ± 0.05) (Figure S4). As no statistically significant difference was found between these models, the shorter ProtTrans‐X5/MACCS Keys combination was used for the following experiments (Figure 4b).

We then evaluated the performance of the model on enzymes which were not included in the training dataset. For that purpose, we collected published data on substrate specificity of 11 T3PKSs from fungi, yielding a total of 110 enzyme/substrate pairs. The dataset exhibited strong positivity bias, as 95 enzyme/substrate pairs (86% of the total number) were active. In addition, more than half of the data points (65) were composed of both the enzyme and the substrate that were not included in our training dataset. When tested on this dataset, the calculated accuracy and F1 score for our model reached 75 ± 3% and 0.84 ± 0.02, respectively (Figure 4c). Additionally, to assess the phylogenetic bias of our model, we gathered 122 enzyme/substrate pairs for 6 plant and 9 bacterial T3PKSs (Table S2), of which 88 were active (72%). When tested on this dataset, the model had lower performance with 56 ± 4% accuracy and 0.7 ± 0.04 F1 score (Figure S5).

In parallel, we used the activity data to train a descriptive machine learning model in order to highlight amino acid residues that might be important for substrate specificity (Supporting Results).

### Experimental Validation of the Machine Learning Model on Additional Substrates and Enzymes

To further test the limits of our model's applicability, we performed experimental validation with a panel of substrates that were not present in the training dataset (Figure 4d). We predicted activity for all our PKSs with 12 chemically diverse carboxylic acids that we found were amenable to enzymatic CoA ligation using Os4CL from *Oryza sativa* ^[^ ^37^ ^]^ or PqsA from *Pseudomonas aeruginosa* ^[^ ^38^ ^]^ (Figure 4e and Figure S7). From there, we chose 10 enzymes with varying degrees of predicted substrate tolerance for experimental testing.

The validation dataset thus consisted of 120 enzyme‐substrate pairs, of which 69 (57.5%) were predicted to be active (Figure 4f). We cloned the genes with a C‐terminal hexahistidine‐tag, expressed them in *E. coli* and purified them to homogeneity alongside the two CoA ligases. We then tested the substrate specificity of the T3PKSs in a two‐step cascade reaction and analysed the products with LC‐MS (Figure 4d and Figure S8). The accuracy of the model was marginally lower than that within the training set (75 ± 2% accuracy and 0.8 ± 0.01 F1 score), indicating its suitability for predictions of substrate specificity with new substrates (Figure 4b). Of note, the model made accurate predictions even for substrates **13–16** that are relatively distant from those present in the training set (Figure S9).

Finally, we applied the predictive model to identify new promiscuous enzymes from thus‐far unsampled fungal T3PKSs. We predicted the activity of all fungal T3PKSs across substrates **1–38** and mapped the predictions onto our SSN (Supporting File 6). From this analysis, we selected five sequences that were predicted to accept 89%–97% of all substrates. These included three sequences from previously unsampled SSN clusters **26–28**, and two from clusters 5 and 11 (Figure 2a, Supporting File 7). We expressed them in the cell‐free system and tested their activity on ten aliphatic and aromatic substrates, including fatty acyl substrates **27** and **31,** which were absent from the training data. Two enzymes (AgalPKS and CfunPKS) expressed poorly, and out of the three well‐expressing enzymes, two (AcaePKS and PansPKS) were active on all ten substrates (Figure 4f). AcaePKS originates from the same cluster as AserPKS2. Despite having a lower expression level than AserPKS2 in the cell‐free system (Figure 4f), it was more efficient at converting several substrates, in particular the aromatic ones (Figure S10). The other highly promiscuous enzyme, PansPKS, originates from a previously unsampled SSN cluster 28 and shares ≤ 59% sequence identity with the active T3PKSs tested earlier (Supporting File 8), making functional prediction challenging. These results highlight the model's effectiveness in predicting substrate promiscuity among diverse fungal T3PKSs.

### Pyrone‐, Resorcinol‐, and Quinolone‐type Polyketides in the Activity Profile of Fungal T3PKSs

Until now, we have focused solely on substrate specificity of the PKSs. However, during activity profiling, many enzymes exhibited remarkable product promiscuity and yielded up to four products from a single substrate (Table S3, Figure 5a). To determine the cyclisation mode and the ketide number of the fungal T3PKS products, we analysed representative reactions using high‐resolution tandem mass spectrometry (HR‐MS/MS) and compared fragmentation patterns with those of known T3PKS reaction products (Figures S11–S60). In addition, we scaled up the in vitro reaction of FerePKS with substrates **13, 19** and **21**, purified the putative triketide pyrone products **13a, 19a,** and **21a**, and analysed them with ^1^H and ^13^C NMR spectroscopy (Figures S61–S66). We also used the purified products to quantify the conversion of substrates **13, 19** and **21** by fungal T3PKSs as well as PhCHS (Figure S67).

**Figure 5:**
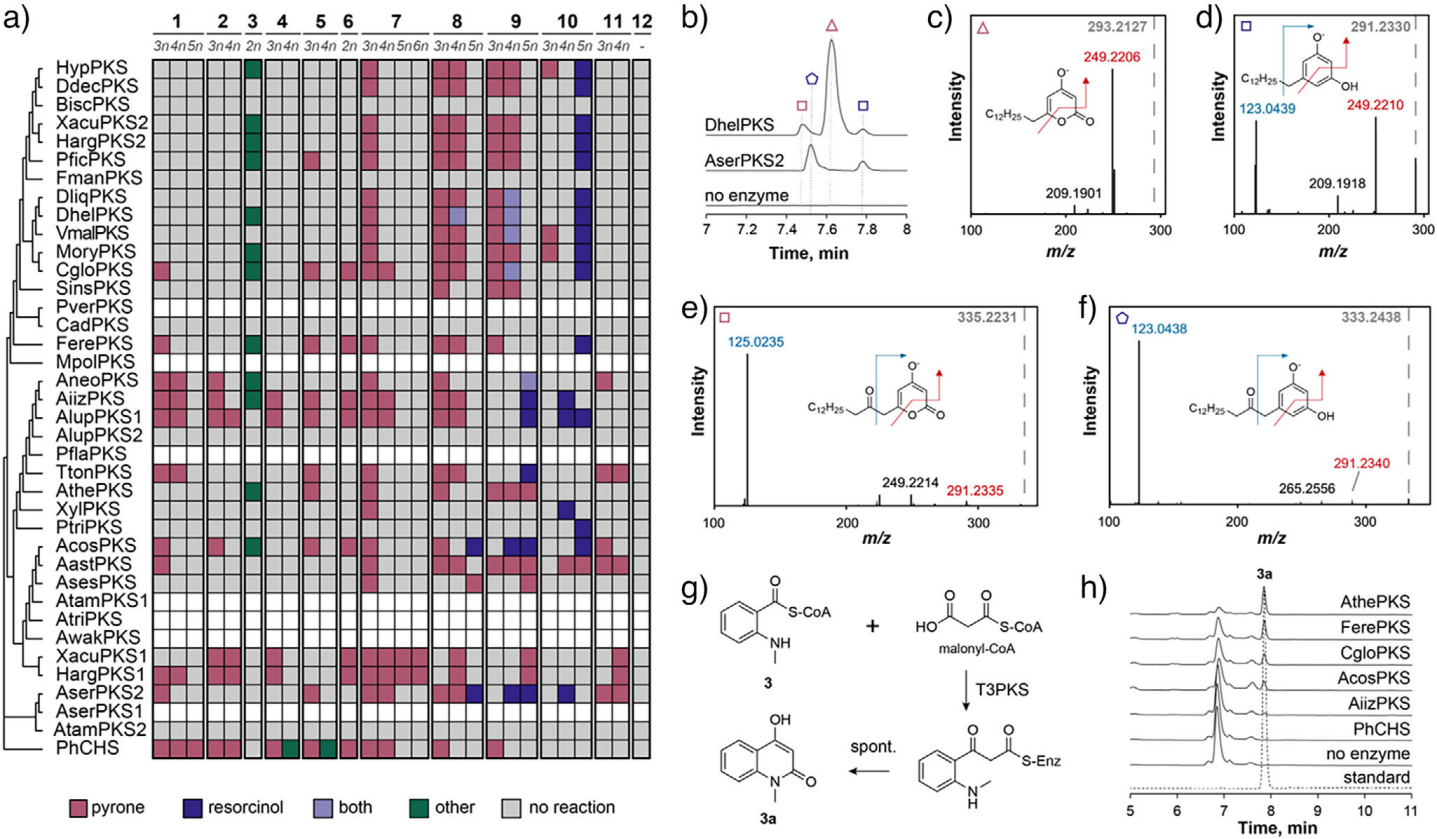
Product scope of the 37 fungal T3PKSs. a) Product profile of the reactions of cell‐free lysates with substrates **1–12**. Ketide number: 2n – diketide; 3n – triketide; 4n – tetraketide; 5n – pentaketide; 6n – hexaketide. b) Extracted ion chromatograms of the reaction products of DhelPKS and AserPKS2 with substrate **9** compared to the “no enzyme” control. Putative tri‐ and tetraketide pyrone products are marked with pink triangle and rectangle, respectively. Putative tetra‐ and pentaketide resorcinol products are marked with blue rectangle and pentagon, respectively. c)–f) HR‐MS/MS spectra and the proposed fragmentation patterns of the above‐mentioned reaction products. The precursor ion is indicated with a dashed grey line labelled with its *m/z* value. g) Proposed^[^ ^39^ ^]^ scheme of diketide quinolone formation from **3**. h) HPLC‐based identification of reaction products of purified T3PKSs with **3** at 273 nm. Dotted line represents the authentic standard of 4‐hydroxy‐1‐methyl‐2‐quinolone.

Since this study was conceived amid the controversy regarding flavonoid production in fungi,^[^ ^37^, ^38^, ^40^ ^]^ and most fungal T3PKSs in public databases are annotated as chalcone synthases, we first looked for *m/z* values corresponding to the fragmentation of phloroglucinol‐type products. However, we did not find such products, suggesting that fungal T3PKSs are unlikely to synthesise flavonoid scaffolds. This conclusion is corroborated by the recent discovery of fungal NRPS‐PKS hybrids that produce flavonoids naringenin^[^ ^41^ ^]^ and chlorflavonin.^[^ ^42^ ^]^

Lactonisation, however, appeared to be the preferred cyclisation mechanism for ring‐type substrates **1–4**, as well as short‐chain (**5**–**7**) and branched‐chain (**11**) fatty acyl substrates. Fragmentation patterns corresponding to resorcinol products were observed only with fatty acyl substrates **8–10**, and the propensity for aldol condensation increased with chain length. Reactions with **8–10** typically yielded a mixture of pyrone and resorcinol products (Figure 5b–f). However, several enzymes exhibited stricter cyclisation specificity: AiizPKS, AlupPKS1, AcosPKS and AserPKS2 were selective for resorcinol products with substrates **9–10**, while AastPKS was the only enzyme that yielded pyrones even from the longest substrate in the panel (**10**).

Within the T3PKS pairs stemming from the same organisms, HargPKS1 and XacuPKS1 were specific for lactonisation, while HargPKS2 and XacuPKS2 produced pentaketide resorcinols from **10**.

Resorcinol‐type phenolic lipids are relatively common in fungi,^[^ ^43^, ^44^, ^45^, ^46^, ^47^, ^48^ ^]^ while reports of 2‐pyrones with a long hydrophobic chain are rare.^[^ ^49^ ^]^ Furthermore, all but one^[^ ^50^ ^]^ previously reported fungal T3PKSs favour aldol over lactone cyclisation with C10‐C20 substrates. The presence of several lactone‐yielding enzymes in our set is thus surprising. It is possible that the pyrones are shunt products caused by the feeding of nonphysiological substrates, as commonly observed with plant chalcone synthases in vitro.^[^ ^51^ ^]^ The true substrates of the PKSs tested here may be provided by enzymes encoded within the underlying biosynthetic gene clusters (BGCs), such as dedicated CoA ligases activating exotic carboxylic acids, or other core biosynthetic enzymes forming complex pathway intermediates.

The product profiles of fungal T3PKSs differed considerably from that of PhCHS. While most of the fungal enzymes yielded resorcinols from longer fatty acyl substrates, we did not detect any resorcinol products in reactions with PhCHS. With substrates **4** and **5**, its physiological substrates, PhCHS predominantly formed the Claisen product of the tetraketide, while no such products were observed with the fungal enzymes. With substrates **13**, **14**, **19,** and **23**, HargPKS1 and XacuPKS1 were selective for the tetraketide pyrone product, while PhCHS yielded predominantly triketide pyrones (Figure S8). With substrate **3**, twelve fungal enzymes, but not PhCHS, produced a compound with an *m/z* of 174 in the negative ion mode, suggesting a diketide product (Figure 5g). The UV absorbance spectrum of the compound with two local maxima at 273 nm and 315 nm suggested the formation of a fused ring structure of the quinolin‐2‐one type^[^ ^52^, ^53^ ^]^ (Figure S15b). Indeed, its MS/MS fragmentation pattern (Figure S15c) was identical to that of 4‐hydroxy‐1‐methyl‐2‐quinolone produced by plant quinolone synthases,^[^ ^31^ ^]^ and the authentic standard of 4‐hydroxy‐1‐methyl‐2‐quinolone co‐eluted with the reaction product peak (Figure 5h). To our knowledge, this is the first report of quinolone formation by a fungal T3PKS. While fungal 6,6‐quinolones are known to originate from an NRPS pathway,^[^ ^54^ ^]^ the potential involvement of T3PKSs in the synthesis of simple fungal 2‐quinolones, such as penicinolone,^[^ ^55^ ^]^ remains to be investigated.

### Genomic Context of Fungal T3PKS Genes

Lastly, we examined the genome neighbourhoods surrounding the genes encoding the T3PKSs tested to assess synteny and possible functional conservation of the encoded genes. Overall, the immediate neighbourhood of the T3PKS genes is well conserved across most SSN clusters (Supporting File 9). However, in some SSN clusters, we observed several distinct groups of putative BGCs (clusters **1–3** and **1–4**) or little conservation in the gene neighbourhood (cluster 15), suggesting that the SSN clusters are often but not always representative of functional similarity.

T3PKS genes are frequently co‐localised with genes encoding putative tailoring enzymes—cytochrome p450s, aldo‐keto reductases and short‐chain dehydrogenases. Furthermore, T3PKS genes from clusters 9, 10, 12, 22, 23, 26 and 28 originate from hybrid biosynthetic gene clusters that also encode a T1PKS (AlupPKS2, AiizPKS, XylPKS, AthePKS, CcauPKS, PansPKS), a nonribosomal peptide synthetase (TtonPKS), or a dimethylallyltryptophane synthase (AiizPKS, AlupPKS1).

**Figure 6:**
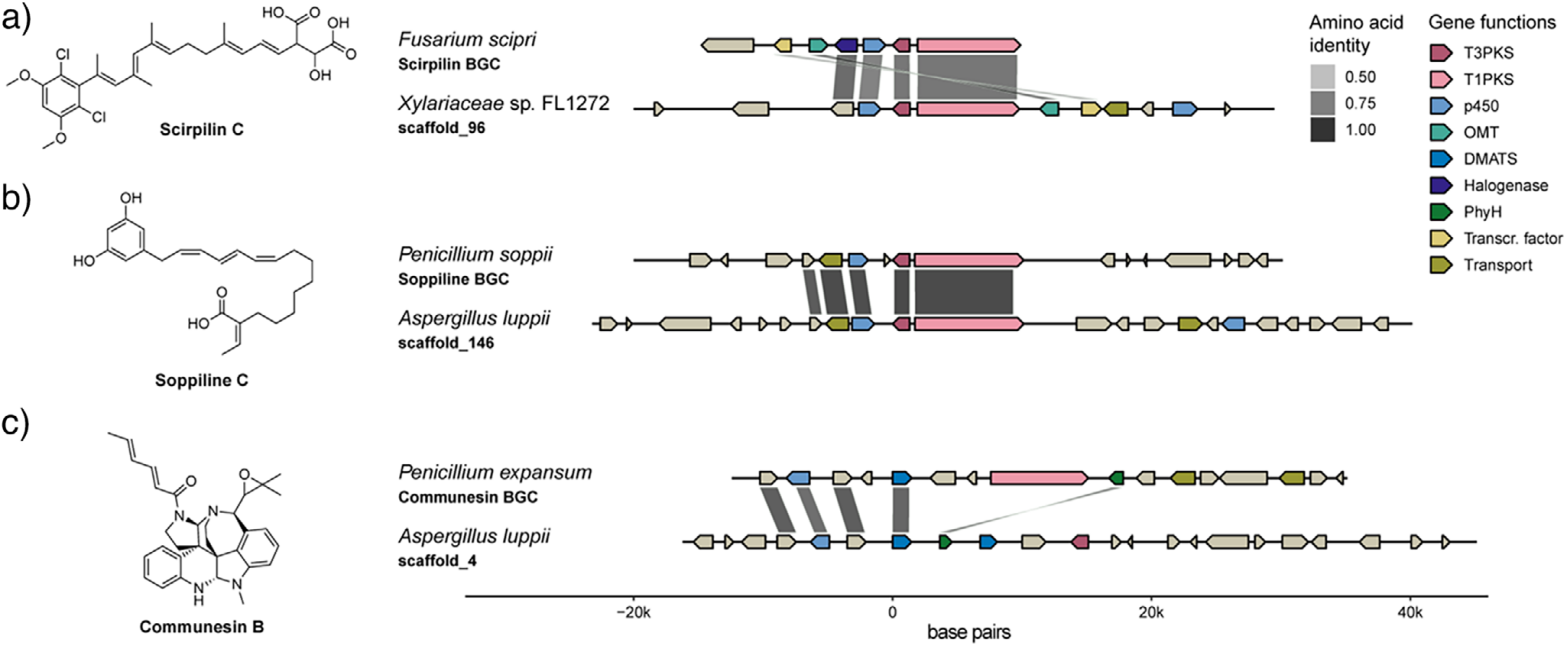
Genomic neighbourhood of the fungal T3PKSs. a) Comparison of the scirpilin biosynthetic gene cluster in *Fusarium scirpi* (GenBank: OR551244) and the genomic neighbourhood of the XylPKS‐encoding gene in *Xylariaceae sp*. FL1272. b) Comparison of soppiline biosynthetic gene cluster in *Penicillium soppii* (MIBiG^[^ ^56^ ^]^: BGC0003032) and the genomic neighbourhood of the AlupPKS2‐encoding gene in *Aspergillus luppii*. c) Comparison of the communesin biosynthetic gene cluster in *Penicillium expansum* (MIBiG: BGC0001205) and the genomic neighbourhood of the AlupPKS1‐encoding gene in *Aspergillus luppii*. The gene clusters and pairwise amino acid sequence identities between the encoded proteins were visualised using gggenomes.^[^ ^57^ ^]^

While some of these enzymes, like AiizPKS, TtonPKS, and PansPKS, displayed broad substrate promiscuity, others such as XylPKS, AlupPKS2 and CcauPKS showed minimal or no activity. This is likely due to their reliance on T1PKS‐derived substrates, as observed in previously characterized hybrid clusters (e.g., FscB and PspB).^[^ ^19^, ^21^ ^]^ These enzymes only produced product when co‐expressed with their corresponding T1PKSs, suggesting functional dependence.^[^ ^19^, ^21^ ^]^ High sequence similarity between XylPKS/FscB and AlupPKS2/PspB, along with conservation of other cluster enzymes, supports a similar biosynthetic logic (Figure 6a,b).

AiizPKS and AlupPKS1 co‐localise with one or two DMATS genes, enzymes typically involved in tryptophan prenylation but also active on tyrosine and aromatic polyketides.^[^ ^58^ ^]^ This unusual co‐localisation with T3PKSs – unreported to date—suggests potential hybrid polyketide‐indole or meroterpenoid biosynthesis. Notably, the AlupPKS1 cluster resembles the communesin B BGC^[^ ^59^ ^]^ but encodes a T3PKS and two DMATSs instead of a T1PKS and one DMATS (Figure 6c). Given the broad substrate scope of AlupPKS1, functional studies are needed to elucidate the nature of the encoded hybrid product.

T3PKS genes from Sordariomycetes (SSN clusters 1, 3, 13, and 19) often lack nearby tailoring genes but are frequently flanked by genes encoding putative carbohydrate‐active enzymes (CAZymes), such as glycosyl hydrolases and pectate lyases (Figure S68). Some fungal CAZymes have been implicated in shaping lifestyles associated with plant biomass, such as endophytism and phytopathogenicity.^[^ ^60^, ^61^ ^]^ Similarly, the presence of a chalcone synthase‐type gene in the genome was recently identified as one of the genetic determinants of endophytism in the *Arabidopsis* mycobiota.^[^ ^62^ ^]^ Although a direct functional interaction between the encoded CAZymes and the T3PKS seems unlikely, their co‐localisation in this syntenic region remains to be investigated.

## Conclusion

Biocatalysis holds promise to reshape our approach to chemical and pharmaceutical synthesis, but its wider implementation is limited by the number of characterised enzymes at our disposal. In this study, we expressed and functionally characterised 42 new T3PKSs, essentially tripling the number of previously studied fungal enzymes in this family. Roughly half of the 24 CoA thioesters used here as substrates had not been tested with fungal—or any—T3PKSs before. As a result, we achieved the production of unnatural 2‐pyrones with pharmaceutically valuable alkyne,^[^ ^63^ ^]^ furan,^[^ ^64^ ^]^ and thiazole^[^ ^65^ ^]^ moieties, as well as several precursors of bioactive natural products. For instance, TtonPKS converted **20** to the direct precursor of kavain, an anticonvulsant from the kava plant, and half of the active PKSs converted derivatives of anthranilic acid to heterocyclic quinolin‐2‐one alkaloids. The ability to synthesise these biologically active scaffolds was thus far believed to be confined to a small group of divergent T3PKSs from Rutaceous plants^[^ ^31^, ^39^, ^52^ ^]^ and PqsD from the biosynthetic pathway of the *Pseudomonas* quinolone signal, PQS.^[^ ^66^ ^]^ This unexpected insight into substrate and product specificities of fungal T3PKSs opens new possibilities for their recruitment in biocatalytic routes towards pharmaceutically relevant compounds.

In combination with high‐throughput enzyme assays, machine learning is gaining traction as a powerful tool for substrate specificity prediction, with several domain‐specific,^[^ ^67^ ^]^ enzyme family‐specific^[^ ^68^, ^69^, ^70^, ^71^ ^]^ and general^[^ ^72^ ^]^ models reported recently. Building on our activity profiling data, we trained the first machine learning model for T3PKS substrate specificity prediction, which achieved reasonable accuracy despite the relatively modest size of our dataset (341 data points). This could be attributed to thorough sampling from the whole fungal T3PKS sequence space and a panel of structurally diverse substrates resulting in a balanced training dataset. Although predictive ML models tend to underperform with small molecules that were not present in the training dataset,^[^ ^72^ ^]^ we found that our model performs well with an extended panel of substrates that are relatively distant from those in the training dataset. We anticipate that this feature can be valuable for narrowing down the number of enzymes to be screened for activity with a given substrate, especially in cases that are counterintuitive or hard to predict with human intelligence alone. For instance, the model correctly predicted that several enzymes would accept the bulky, nonplanar substrate **19** despite being inactive with simpler aromatic substrates **1** or **2**. A current limitation of our model, however, is the fungi‐focused nature of the training dataset. While it adequately predicts the activity of fungal T3PKSs, its performance declines dramatically with plant and bacterial enzymes. A unified model for phylogeny‐independent prediction of T3PKS substrate specificity would thus be a logical follow‐up to this work.

This study addresses two longstanding challenges in T3PKS research: 1) predicting substrate specificity, and 2) the widespread mis‐annotation of fungal T3PKSs as chalcone synthases. Our results may guide future efforts to elucidate the mechanism underlying substrate selection in fungal T3PKS and to shift the substrate specificity by enzyme engineering. From the biocatalytic perspective, our approach identified highly promiscuous enzymes that enabled precursor‐directed biosynthesis of several pharmaceutical precursors and unnatural polyketides. Future experimental efforts will focus on harnessing this promiscuity for chemoenzymatic and in vivo biosynthetic applications and elucidating the native functions of these enzymes.

## Supporting Information

The authors have cited additional references within the Supporting Information.^[^ ^20^, ^73^, ^74^, ^75^, ^76^, ^77^, ^78^, ^79^, ^80^, ^81^, ^82^, ^83^, ^84^, ^85^, ^86^, ^87^, ^88^, ^89^, ^90^, ^91^, ^92^, ^93^, ^94^, ^95^, ^96^, ^97^, ^98^, ^99^, ^100^, ^101^, ^102^, ^103^, ^104^, ^105^, ^106^, ^107^, ^108^, ^109^, ^110^, ^111^, ^112^, ^113^, ^114^, ^115^, ^116^, ^117^, ^118^, ^119^, ^120^, ^121^, ^122^, ^123^, ^124^, ^125^ ^]^

## Conflict of Interests

The authors declare no conflict of interest.

## Supporting information



Supporting Information

Supporting Information

## Data Availability

The data that support the findings of this study are available from the corresponding author upon reasonable request.
