# Supplementary material for: Unravelling the Functional Diversity of Type III Polyketide Synthases in Fungi
Source: Angew Chem Int Ed Engl. 2025 Sep 4;64(44):e202514786. doi: 10.1002/anie.202514786 (PMC12559476; doi:10.1002/anie.202514786)
Supplement: Supplementary file 1 — Supporting Information [file ANIE-64-e202514786-s002.docx]

Supporting Information for

Unravelling the Functional Diversity of Type III Polyketide Synthases in Fungi

Nika Sokolova^1^, Stepan S. Denisov^2^, Thomas Hackl^3^, and Kristina Haslinger^1^

^1^Department of Chemical and Pharmaceutical Biology, University of Groningen, The Netherlands

^2^Institute of Biological Chemistry, University of Vienna, Vienna, Austria

^3^Groningen Institute for Evolutionary Life Sciences, University of Groningen, Groningen, The Netherlands

Correspondence: [k.haslinger@rug.nl](mailto:k.haslinger@rug.nl)

Table of Contents

[Experimental Section 5](#_Toc207365687)

[Materials 5](#_Toc207365688)

[Selection of enzymes of interest and bioinformatic analyses 5](#_Toc207365689)

[Design and synthesis of DNA templates for cell-free expression 6](#_Toc207365690)

[Construction of plasmids 6](#_Toc207365691)

[Cell-free expression 6](#_Toc207365692)

[Split-GFP complementation assay 6](#_Toc207365693)

[*In vitro* T3PKS assays 7](#_Toc207365694)

[Analysis of T3PKS reaction products 7](#_Toc207365695)

[Semi-preparative scale enzymatic reaction, purification and NMR analysis of **13a, 19a** and **21a** 8](#_Toc207365696)

[Quantification of T3PKS reaction products 9](#_Toc207365697)

[Synthesis of CoA thioesters 9](#_Toc207365698)

[Expression and purification of T3PKSs 10](#_Toc207365699)

[Machine learning 10](#_Toc207365700)

[Supporting Tables 12](#_Toc207365701)

[**Table S1**. List of putative fungal T3PKSs selected for experimental characterisation. 12](#_Toc207365702)

[**Table S2**. List of T3PKSs from fungi, plants and bacteria with published activity data that were used in the ML validation experiments in this study. 13](#_Toc207365703)

[**Table S3**. Low-resolution LC-MS analysis of the T3PKS reaction products detected during activity profiling with substrates **1-12** and machine learning validation experiments with substrates **13-24, 27** and **31**. High-resolution LC-MS/MS analysis of the products is shown in Figures S11-S60. 14](#_Toc207365704)

[**Table S4**. List of plasmids used in this study. 16](#_Toc207365705)

[Supporting Figures 17](#_Toc207365706)

[**Figure S1**. A sequence similarity network of the putative fungal T3PKSs mined from the Mycocosm database at 57% sequence identity cutoff. 17](#_Toc207365707)

[**Figure S2.** Cluster 1 of the sequence similarity network of fungal T3PKSs. 18](#_Toc207365708)

[**Figure S3.** Benchmarking of the express-test workflow with PhCHS. 18](#_Toc207365709)

[**Figure S4.** Boxplots of ML performance metrics for the tested ML algorithms and feature vectors obtained on the cell-free substrate activity dataset. 19](#_Toc207365710)

[**Figure S5.** Testing of phylogenetic bias of the predictive ML model. 20](#_Toc207365711)

[**Figure S6.** Structures of compounds **25-38** previously reported to be accepted by fungal T3PKSs. 21](#_Toc207365712)

[**Figure S7.** Enzyme/substrate specificity prediction for 31 active T3PKSs with substrates **13-24** by the Multi-Layer Perceptron algorithm with the ProtTrans-X5/MACCS Keys feature vectors. 22](#_Toc207365713)

[**Figure S8.** Extracted ion chromatograms in low-resolution LC-MS of reaction products of the purified fungal T3PKSs and PhCHS with substrates **13-24** compared to the "no enzyme" control. 25](#_Toc207365714)

[**Figure S9.** Representation of the chemical space of T3PKS substrates obtained by t-SNE dimensionality reduction of a substrate similarity matrix. 27](#_Toc207365715)

[**Figure S10.** Extracted ion chromatograms in low-resolution LC-MS of reaction products of the cell-free expressed AcaePKS and AserPKS2. 28](#_Toc207365716)

[**Figure S11.** Spectral data analysis of product **1a**. 29](#_Toc207365717)

[**Figure S12.** Spectral data analysis of product **1b**. 30](#_Toc207365718)

[**Figure S13.** Spectral data analysis of product **2a**. 31](#_Toc207365719)

[**Figure S14.** Spectral data analysis of product **2b**. 32](#_Toc207365720)

[**Figure S15.** Spectral data analysis of product **3a**. 33](#_Toc207365721)

[**Figure S16.** Spectral data analysis of product **4a**. 34](#_Toc207365722)

[**Figure S17.** Spectral data analysis of product **5a**. 35](#_Toc207365723)

[**Figure S18.** Spectral data analysis of product **6a**. 36](#_Toc207365724)

[**Figure S19.** Spectral data analysis of product **7a**. 37](#_Toc207365725)

[**Figure S20.** Spectral data analysis of product **7b**. 38](#_Toc207365726)

[**Figure S21.** Spectral data analysis of product **7c**. 39](#_Toc207365727)

[**Figure S22.** Spectral data analysis of product **7d**. 40](#_Toc207365728)

[**Figure S23.** Spectral data analysis of product **8a**. 41](#_Toc207365729)

[**Figure S24.** Spectral data analysis of product **8b**. 42](#_Toc207365730)

[**Figure S25.** Spectral data analysis of product **8c**. 43](#_Toc207365731)

[**Figure S26.** Spectral data analysis of product **8d**. 44](#_Toc207365732)

[**Figure S27.** Spectral data analysis of product **8e**. 45](#_Toc207365733)

[**Figure S28.** Spectral data analysis of product **9a**. 46](#_Toc207365734)

[**Figure S29.** Spectral data analysis of product **9b**. 47](#_Toc207365735)

[**Figure S30.** Spectral data analysis of product **9c**. 48](#_Toc207365736)

[**Figure S31.** Spectral data analysis of product **9d**. 49](#_Toc207365737)

[**Figure S32.** Spectral data analysis of product **9e**. 50](#_Toc207365738)

[**Figure S33.** Spectral data analysis of product **10a**. 51](#_Toc207365739)

[**Figure S34.** Spectral data analysis of product **10b**. 52](#_Toc207365740)

[**Figure S35.** Spectral data analysis of product **10c**. 53](#_Toc207365741)

[**Figure S36.** Spectral data analysis of product **10d**. 54](#_Toc207365742)

[**Figure S37.** Spectral data analysis of product **10e**. 55](#_Toc207365743)

[**Figure S38.** Spectral data analysis of product **11a**. 56](#_Toc207365744)

[**Figure S39.** Spectral data analysis of product **11b**. 57](#_Toc207365745)

[**Figure S40.** Spectral data analysis of product **13a**. 58](#_Toc207365746)

[**Figure S41.** Spectral data analysis of product **13b**. 59](#_Toc207365747)

[**Figure S42.** Spectral data analysis of product **14a**. 60](#_Toc207365748)

[**Figure S43.** Spectral data analysis of product **14b**. 61](#_Toc207365749)

[**Figure S44.** Spectral data analysis of product **15a**. 62](#_Toc207365750)

[**Figure S45.** Spectral data analysis of product **16a**. 63](#_Toc207365751)

[**Figure S46.** Spectral data analysis of product **17a**. 64](#_Toc207365752)

[**Figure S47.** Spectral data analysis of product **18a**. 65](#_Toc207365753)

[**Figure S48.** Spectral data analysis of product **19a**. 66](#_Toc207365754)

[**Figure S49.** Spectral data analysis of product **19b**. 67](#_Toc207365755)

[**Figure S50.** Spectral data analysis of product **20a**. 68](#_Toc207365756)

[**Figure S51.** Spectral data analysis of product **21a**. 69](#_Toc207365757)

[**Figure S52.** Spectral data analysis of product **21b**. 70](#_Toc207365758)

[**Figure S53.** Spectral data analysis of product **22a**. 71](#_Toc207365759)

[**Figure S54.** Spectral data analysis of product **22b**. 72](#_Toc207365760)

[**Figure S55.** Spectral data analysis of product **23a**. 73](#_Toc207365761)

[**Figure S56.** Spectral data analysis of product **23b**. 74](#_Toc207365762)

[**Figure S57.** Spectral data analysis of product **24a**. 75](#_Toc207365763)

[**Figure S58.** Spectral data analysis of the authentic standard of compound **24a**. 76](#_Toc207365764)

[**Figure S59.** Spectral data analysis of product **27a**. 77](#_Toc207365765)

[**Figure S60.** Spectral data analysis of product **31a**. 78](#_Toc207365766)

[**Figure S61.** ^1^H NMR spectrum of compound **13a**. 79](#_Toc207365767)

[**Figure S62.** ^13^C NMR spectrum of compound **13a**. 80](#_Toc207365768)

[**Figure S63.** ^1^H NMR spectrum of compound **19a**. 81](#_Toc207365769)

[**Figure S64**. ^13^C NMR spectrum of compound **19a** 82](#_Toc207365770)

[**Figure S65.** ^1^H NMR spectrum of compound **21a**. 83](#_Toc207365771)

[**Figure S66.** ^13^C NMR spectrum of compound **21a**. 84](#_Toc207365772)

[**Figure S67.** Conversion of substrates **13**, **19** and **21** by purified fungal T3PKSs and PhCHS. a) conversion to product **13a**; b) conversion to product **19a**; a) conversion to product **21a**. 84](#_Toc207365773)

[**Figure S68.** T3PKSs originating from endophytic or phytopathogenic fungi. 85](#_Toc207365774)

[**Figure S69.** Calibration plots for products **13a**, **19a** and **21a.** 86](#_Toc207365775)

[Supporting results 87](#_Toc207365776)

[Machine learning proposes amino acid residues important for substrate specificity 87](#_Toc207365777)

[**Figure S70.** Descriptive machine learning model. 87](#_Toc207365778)

[**Figure S71.** Multiple sequence alignment of the amino acids lining active site and substrate-binding tunnel in the 31 expressed T3PKSs. 89](#_Toc207365779)

[Supporting References 90](#_Toc207365780)

## Experimental Section

### Materials

CoA thioesters for T3PKS assays were purchased from Sanbio B.V. (Uden, The Netherlands), TransMIT (Gießen, Germany) or Endotherm Life Science Molecules (Saarbrücken, Germany). Malonyl-CoA, CoA and carboxylic acids for CoA ligation were purchased from Sigma-Aldrich (St. Louis, USA) or BLD Pharm (Shanghai, China). The myTXTL® kit for cell-free expression was obtained from Daicel Arbor Biosciences (Ann Arbor, USA). Linear gene fragments used as templates for cell-free expression were ordered as gBlocks™ from Integrated DNA Technologies, Inc. (IDT; Coralville, USA).

### Selection of enzymes of interest and bioinformatic analyses

Two different multiple sequence alignments (MSAs) of known T3PKSs from fungi and plants (Supporting File 1) were generated with mafft v7.505^[1]^ (parameters: --thread 8 --maxiterate 1000 --genafpair --reorder) and converted to Hidden Markov Model (HMM) profiles using the msa2profile function of MMseqs2^[2]^. The resulting HMM profiles were used to query the nucleotide sequences of 2096 fungal genomes from the Joint Genome Institute (JGI) MycoCosm repository^[3]^ using the easy-search function of MMseqs2 (parameters: -s 7). Hits representing exons of the same open reading frame were concatenated and converted to amino acid sequences. The hits were combined with 613 fungal proteins annotated as belonging to the IPR011141 family of the InterPro database^[4]^. The combined and dereplicated set of 1640 sequences was used as input for calculating a sequence similarity network with a webtool of the Enzyme Function Initiative (EFI-EST)^[5]^; edge selection cut-off: alignment score threshold 40. To reduce complexity of the sequence similarity network (SSN), we opted for the "representative node" mode, where sequences sharing 95% sequence identity are grouped and visualised as a single node. The finalised network was visualised in Cytoscape 3.8.2^[6]^ with the yFiles organic layout. The sequences were filtered by length (300 to 600 amino acids) to exclude fragments, and the final sequence identity cutoff was set to 80%.

Sequences from each cluster were retrieved and aligned with a set of reference T3PKSs from plants (Supporting File 1) using mafft (parameters: --genafpair). Residues from positions 132, 133, 164, 194, 197, 215, 256, 265, 303, 336, 338 and 375 (residue numbering from MsCHS, Uniprot: P30074) were extracted and compared across fungal and plant T3PKSs. An MSA of all fungal T3PKSs and 184 reviewed plant T3PKSs from the InterPro database was performed in the same way and visualised as a sequence logo using the R package ggseqlogo^25^. From the thus analysed clusters, enzymes were chosen for experimental characterisation (Table S1). In total, 25 clusters representing taxonomic and ecological diversity of the host organisms, as well as the putative functional diversity hinted at by the active site composition, were selected. For sequence selection within the clusters, we prioritised those with active site residues corresponding to the cluster consensus and those stemming from highly contiguous, high-quality genome assemblies.

To examine the T3PKS-encoding gene neighbourhoods within each SSN cluster, 50,000 base pairs both up- and downstream of the T3PKS-encoding gene were extracted from the corresponding genome using the subseq function of seqkit^[7]^ (parameters: -d 50000 -u 50000). For biosynthetic gene cluster comparisons, the BGCs were first predicted with the command line version of antiSMASH 7.0.0^[8]^ (parameters: --taxon fungi --cassis --clusterhmmer --genefinding-gff3 --genefinding-tool none). BGC comparison and visualisation was performed using gggenomes v0.9.12.9000^[9]^.

### Design and synthesis of DNA templates for cell-free expression

The selected enzymes were back-translated into DNA sequences, codon-optimised for expression in *E. coli* K12 with the IDT optimisation algorithm and manually modified to exclude recognition sites for NcoI, HindIII and XhoI restriction enzymes. The 5' end of all DNA fragments was designed with an identical overhang of 100 bp, the p70a promoter sequence, a ribosome-binding site and an NcoI recognition site to facilitate cloning into the pET28a(+) (Novagen) expression vector. The 3' end of each gene was designed to include the recognition site for HindIII, the in-frame codons for the Tobacco Edge Virus protease cleavage site, a hexahistidine-tag, and a GFP11 tag. After the stop codon, each gene fragment included an XhoI recognition site, the T500 terminator sequence and a 100 bp overhang. The synthetic DNA was obtained as gBlocks Gene Fragments from IDT. The sequences of all synthetic DNA constructs are provided in Supporting File 5, and gene fragment annotations are included in Supporting File 10.

### Construction of plasmids

Genes coding for T3PKSs selected for expression in *E. coli* were amplified by polymerase chain reaction (PCR) from the synthetic DNA fragments and cloned by restriction and ligation (NcoI/HindIII) into pET28a(+) for expression under the T7 promoter. The gene encoding the standard protein PhCHS-GFP11 for split-GFP assays was cloned into pET28a(+) by restriction and ligation with NcoI/XhoI to retain the GFP11 tag. All constructs were verified by Sanger sequencing (Macrogen Europe, Amsterdam). The list of all plasmids used in this study is provided in Table S4.

### Cell-free expression

Cell-free expression was performed with the myTXTL® Linear DNA kit from Daicel Arbor Biosciences according to the manufacturer’s instructions. Briefly, the synthesised DNA fragments were reconstituted in ultrapure water to a final concentration of 80 nM and stored at -20 °C between experiments. myTXTL® lysate was thawed on ice and aliquoted at 9 µL into 1.5 mL tubes or 96 well plates. 3 µL of DNA template were added to each tube or well and mixed by pipetting (final concentration 20 nM). The reactions were briefly centrifuged to collect the liquid at the bottom and transferred to an incubator at 29 °C for 20 h. As a negative control ('no enzyme'), one reaction was performed with 3 µL of ultrapure water instead of DNA. After incubation, the reactions were diluted 2.5-fold with ultrapure water and used directly for the split-GFP and T3PKS assays.

### Split-GFP complementation assay

T3PKS expression in the cell-free system was quantified using a split-GFP complementation assay. The GFP fluorescence complementation fragment GFP1–10 was expressed in *E. coli* BL21(DE3) from the plasmid pAGM22082_sfGFP1-10^[10]^, prepared as inclusion body pellets according to a published protocol^[11]^ and stored at –70 °C between experiments. For the measurement, a pellet of GFP1–10 was fully dissolved in 9 M urea and resuspended in 25 mL of TNG buffer (100 mM Tris-HCl pH 7.4, 100 mM NaCl, 10% (v/v) glycerol). 2.5 µL of the diluted myTXTL® reactions were aliquoted into a 96-well µClear® white plate (Greiner Bio-One, Kremsmünster, Austria) and mixed with 7.5 µL of TNG buffer. In parallel, serial dilutions of the standard protein (purified PhCHS-GFP11, see section “Expression and purification of T3PKSs”) were aliquoted in triplicate into the same plate. 90 µL of the GFP1–10 solution was then added to each well. Immediate fluorescence values were measured using a FLUOstar Omega (BMG LABTECH, Ortenberg, Germany) microplate reader (excitation wavelength: 485 nm; emission wavelength: 520 nm; bottom read mode). The plate was incubated at 4 °C overnight, and the final fluorescence values were measured with the same parameters. Complementation fluorescence ΔF was calculated from equation 1,

$\Delta F=\left( Fs_{final}-Fb_{final} \right)-\left( Fs_{initial}-Fb_{initial} \right)$,

where Fs_final_ and Fs_initial_ are the final and initial fluorescence values for a sample, and Fb_final_ and Fb_initial_ are the final and initial fluorescence values for the blank (myTXTL + ultrapure water). ΔF values were converted to protein concentrations in μM based on the calibration curve with the standard protein.

### *In vitro* T3PKS assays

The standard T3PKS reaction mixture consisted of 0.3 mM starter-CoA, 0.3 mM extender-CoA (malonyl-CoA) and 5 µL of the diluted myTXTL® lysate or purified enzyme (final concentration 3 µM) in 50 mM Tris/HCl pH 7.5. The total reaction volume was 50 µL, and the reaction was initiated by the addition of the enzyme. After 24 h of incubation at 30 °C, 300 µL of 9:1 ethyl acetate-methanol (v/v) supplemented with 0.1% formic acid were added and the plates were shaken for 20 min. After centrifuging at 3,428 × g for 15 min, 250 µL of the organic layer were transferred to another plate with a JANUS liquid handler (PerkinElmer, Waltham, United States) and dried under a stream of N_2_. The dried extracts were resuspended in 25 µL methanol supplemented with 0.1% formic acid and used for chromatographic analysis. Reactions with purified PhCHS or ultrapure water served as the positive and negative controls, respectively.

### Analysis of T3PKS reaction products

The extracted PKS reactions were first analysed using a low-resolution Waters Acquity Arc UHPLC-MS system equipped with a 2998 PDA detector and a QDa single-quadrupole mass detector. The samples were separated over a Waters XBridge BEH C18 3.5 μm 2.1×50 mm column at 40 °C with a concentration gradient (solvent A: water +0.1 % formic acid, and solvent B: acetonitrile + 0.1 % formic acid) at a flow rate of 0.5 mL/min (2 μL injections). The following gradient was used: 5 % B for 2 min, 5–90 % B over 3 min; 90 % B for 2 min; 5 % B for 3 min. For reactions with oleoyl-CoA and phytanoyl-CoA, a different gradient was employed: 50 % B for 2 min, 50–70 % B over 4 min; 70–90 % B over 2 min; 90 % B for 2 min; 90–50% B over 2 min; 50 % B over 2 min. MS analysis was carried out in both positive and negative ion modes with the following parameters: probe temperature of 600 °C; capillary voltage of 1.0 kV; cone voltage of 15 V; scan range 100-1250 *m/z*. The acquired data were analysed using the proprietary software MassLynx.

High-resolution and MS/MS analyses of the extracted PKS reactions were performed using a Shimadzu LC20-XR system (Shimadzu Benelux, Den Bosch, The Netherlands) coupled to a Q Exactive Plus mass spectrometer (Thermo Fisher Scientiﬁc, USA). The samples were separated over a Waters XBridge BEH C18 reversed-phase column at 50 °C with a concentration gradient (solvent A: water + 0.1 % formic acid, and solvent B: acetonitrile + 0.1 % formic acid) at a flow rate of 0.5 mL/min (2 μL injections). The following gradient was used: 5 % B for 2 min, 5–90 % B over 3 min; 90 % B for 2 min; 5 % B for 3 min. MS and MS/MS analyses were performed with electrospray ionisation (ESI) in positive or negative ion mode at a spray voltage of 3.5, and sheath and auxiliary gas flow set at 48 and 11, respectively. The ion transfer tube temperature was 255 °C. Spectra were acquired in data-dependent mode with a survey scan at m/z 80 − 1200 at a resolution of 70,000 followed by MS/MS fragmentation of the top 5 precursor ions at a resolution of 17,500. A stepped collision energy of 30-40-55 was used for fragmentation, and fragmented precursor ions were dynamically excluded for 10 s. The acquired data were analysed using MZmine 4.0.3^[12]^.

We discriminated between different cyclisation modes by the characteristic *m/z* value of the ring fragment (125.0244 for pyrone, calculated for C_6_H_5_O_3_^-^; 123.0452 for resorcinol, calculated for C_7_H_7_O_2_^-^; and 153.0193 for phloroglucinol, calculated for C_7_H_5_O_4_^-^). The identity of triketide pyrones, where the ring fragment is typically absent, was confirmed by observing the mass difference of 1.979 in the precursor ion *m/z* compared to tetraketide resorcinols.

### Semi-preparative scale enzymatic reaction, purification and NMR analysis of **13a, 19a** and **21a**

The semi-preparative scale T3PKS reaction mixture consisted of 0.5 mM chemically synthesised substrate **13, 19** or **21** (see *Synthesis of CoA thioesters*), 1 mM malonyl-CoA and 25 µM purified FerePKS in 100 mM HEPES/NaOH pH 8. The total reaction volume was 12 mL (distributed into 24 reactions of 0.5 mL each). After 16 h of incubation at 37 °C, the reactions were extracted with a double volume of ethyl acetate supplemented with 0.1 % formic acid. The organic layer was dried under N_2_ and resuspended in a total of 1 mL water:acetonitrile.

For purification, high-performance liquid chromatography was carried out on a HPLC Shimadzu system, equipped with a dual LC-20AD pump and an SPD-20M20A photodiode array detector. An Agilent Zorbax Eclipse XDB−C18 column (80 Å, 4.6 x 150 mm, 5 μm) was used for separation at 30 °C. The mobile phase consisted of a gradient of solution A (ultrapure H_2_O + 0.1 % trifluoroacetic acid) and solution B (acetonitrile + 0.1 % trifluoroacetic acid). The following linear gradient was used: 0–2 min 5 % B, 2–12 min linear increase to 95 % B, 12–13 min held at 95 % B, 13–13.01 min decrease to 5% B, and 13.01–17 min held at 5% B. The injection volume was 50 μL, and the flow rate was set to 1 mL/min. Detection was performed at 285 nm, and the presence of **13a**, **19a** or **21a** in the collected fraction was confirmed with LCMS (using the same method as described in *Analysis of T3PKS reaction products*). Repeat injections were performed until a total volume of ∼30 mL of the fraction containing **13a, 19a** or **21a** was collected. The solution was snap-frozen in liquid N_2_ and lyophilized overnight to obtain ~1 mg dry residue (off-white powder). This was dissolved in 500 μL of DMSO-*d_6_*, transferred to a 5 mm NMR tube and analyzed via ^1^H and ^13^C NMR (SFigures S61-S66).

Compound **13a**

**^1^H NMR** (400 MHz, DMSO-*d_6_*) *δ* 11.71 (s, 1H), 6.02 (s, 1H), 5.23 (s, 1H), 2.88 (t, *J*= 2.7, 1H), 2.63 (t, *J*= 7.1, 2H), 2.49 – 2.44 (m, 2H) ppm. **^13^C NMR** (101 MHz, DMSO-*d_6_*) *δ* 170.4, 164.2, 163.7, 100.6, 88.6, 82.6, 72.4, 31.8, 15.4 ppm. **HRMS** (ESI+), observed *m/z* = 165.0545 (theoretical *m/z* = 165.0546, calculated for [C_9_H_9_O_3_]^+^)*.*

Compound **19a**

**^1^H NMR** (600 MHz, DMSO-*d_6_*) *δ* 11.63 (s, 1H), 7.30 – 7.27 (m, 2H), 7.21 – 7.18 (m, 3H), 6.11 (s, 1H), 5.19 (s, 1H), 2.46 (ddd, *J*= 9.1, 6.6, 4.5, 1H), 2.22 (ddd, *J*= 8.7, 5.7, 4.3, 1H), 1.59 – 1.55 (m, 1H), 1.47 (ddd, *J*= 8.8, 6.5, 4.8, 1H) ppm. **^13^C NMR** (151 MHz, DMSO-*d_6_*) *δ* 174.99, 173.31, 166.41, 140.51, 128.39, 126.19, 125.88, 110.78, 87.74, 25.35, 25.26, 16.17 ppm. **HRMS** (ESI-), observed *m/z* = 227.0711 (theoretical *m/z* = 227.0714, calculated for [C_14_H_11_O_3_]^-^).

Compound **21a**

**^1^H NMR** (600 MHz, DMSO-*d_6_*) *δ* 11.69 (s, 1H), 7.52 – 7.48 (m, 1H), 7.44 – 7.40 (m, 1H), 7.36 (dd, *J*= 6.5, 3.2, 2H), 5.75 (s, 1H), 5.19 (s, 1H), 3.93 (s, 2H) ppm. **^13^C NMR** (151 MHz, DMSO-*d_6_*) *δ* 163.69, 160.21, 158.07, 133.46, 133.26, 131.85, 129.52, 129.24, 127.59, 109.62, 88.44, 36.66 ppm. **HRMS** (ESI-), observed *m/z* = 235.0166 (theoretical *m/z* = 235.0167, calculated for [C_12_H_8_ClO_3_]^-^).

### Quantification of T3PKS reaction products

The purified products **13a**, **19a** and **21a** were used to quantify the activity of purified enzymes AiizPKS, AthePKS, DhelPKS, FerePKS, HargPKS, HypPKS, TtonPKS and PhCHS. The reaction mixture consisted of 0.5 mM chemically synthesised substrate **13**, **19** or **21** (see Synthesis of CoA thioesters), 1 mM malonyl-CoA and 25 µM purified enzyme in 100 mM HEPES/NaOH pH 8. The total reaction volume was 25 µL. After 16 h of incubation at 37 °C, the reactions were stopped by adding an equal volume of ice-cold acetonitrile and centrifuged to precipitate the proteins. The supernatants of the quenched T3PKS reactions were analysed by reversed-phase HPLC (as described in *Semi-preparative scale enzymatic reaction, purification and NMR analysis of* ***13a****,* ***19a*** *and* ***21a****)*. The product peak areas were integrated and converted to concentrations in μM based on calibration curves with the purified products **13a**, **19a** and **21a** (Figure S69).

### Synthesis of CoA thioesters

For enzymatic CoA thioester synthesis, Os4CL from *Oryza sativa*^[13]^ or PqsA from *Pseudomonas aeruginosa*^[14]^ were expressed and purified according to the original protocols and used for CoA ligation reactions with carboxylic acid substrates. The reaction mixture consisted of 5 mM MgCl_2_, 6.25 mM ATP, 1 mM carboxylic acid, 1.5 mM CoA and 10 mM enzyme in 100 mM Tris/HCl pH 7.5. The total volume of the reaction was 100 µL or 1,000 µL for analytical and preparative scale, respectively. The reactions were incubated at 30 °C for 16 h and analysed by HPLC as described by Rautengarten *et al.*^[13]^. The reactions were used directly for T3PKS assays or aliquoted and stored at -20 °C until use.

Substrates **13, 19** and **21** for the semi-preparative scale T3PKS reaction were synthesised following the modified Kawaguchi acyl-imidazole method^[15,16]^. Briefly, in a 4 mL glass vial with a stir bar, N,N’-carbonyldiimidazole (5.2 mg, 32 µmol, 1.25 equiv.) was dissolved in 0.25 ml of tetrahydrofuran (THF) followed by dropwise addition of 0.25 ml of the respective carboxylic acid in THF (40 µmol, 1.50 equiv.). This mixture was allowed to stir for 30 min at RT before solution of Coenzyme A (free acid) in 0.25 ml water (20 mg, 26 µmol, 1.00 equiv.) was added dropwise. The pH of the reaction was adjusted to 7.0–7.5 with 0.1 N NaOH. The mixture was allowed to react while stirring at room temperature overnight. The reaction was monitored with TLC and HPLC. THF was evaporated *in vacuo* and the remaining aqueous layer was acidified to pH 3–4 with HCl. This solution was used directly for T3PKS assays or aliquoted and stored at -20 °C until use.

### Expression and purification of T3PKSs

Plasmids harbouring the T3PKS genes were transformed into chemically competent *E. coli* BL21(DE3) and maintained on selective LB agar containing 50 μg/ mL kanamycin. A starter culture was inoculated from a single colony (5 mL, LB with ampicillin) and incubated at 37 °C (180 rpm, overnight). The main culture was inoculated from the starter culture (1 : 100) into 200 mL of TB autoinduction medium (20 g/L tryptone, 24 g/L yeast extract, 4 ml/L glycerol, 17 mM KH_2_PO_4_, 72 mM K_2_HPO_4_, 0.2 % w/v lactose and 0.05 % w/v glucose) and incubated at 37 °C (180 rpm, 2 h), after which the temperature was lowered to 18 °C (180 rpm, overnight). All following steps were performed with chilled buffers. The cells were harvested by centrifugation (15 min, 3,428 × g) and the pellet was resuspended in 5 volumes of lysis buffer (buffer A including one EDTA-free protease inhibitor tablet (Roche, Basel, Switzerland); buffer A: 50 mM Tris/HCl pH 7.5, 500 mM NaCl, 20 mM imidazole). The cell suspension was lysed by sonication (50 % duty cycle, 7 cycles of 35 s ON/60 s OFF) and cleared by centrifugation for 60 min at 17,000 × g. The supernatant was loaded onto the Ni-NTA affinity matrix (Qiagen, Hilden, Germany) equilibrated with buffer A by gravity flow. The column was washed with 20 column volumes of wash buffer (50 mM Tris/HCl pH 7.5, 500 mM NaCl, 30 mM imidazole) and eluted stepwise with one column volume of buffers B1 to B6 (buffers B1-B6: 50 mM Tris/HCl pH 7.4, 500 mM NaCl, 50 mM imidazole/ 100 mM imidazole/ 150 mM imidazole/ 200 mM imidazole/ 250 mM imidazole or 500 mM imidazole, respectively). The eluates of each step were collected in separate fractions and analysed by SDS-PAGE. T3PKS-containing fractions with low protein background were pooled and transferred into the storage buffer (10 mM HEPES/NaOH pH 7.5, 50 mM NaCl, 2 mM dithiothreitol, 5% (v/v) glycerol) by three cycles of concentration (Amicon® Ultra Centrifugal Filter; 3 kDa molecular weight cutoff) and dilution (1:30). The protein concentrations were determined by absorbance at 280 nm (NanoDrop, ThermoFisher Scientific, USA) before the purified enzymes were aliquoted and flash-frozen with liquid nitrogen for storage at −70 °C.

### Machine learning

To generate molecular descriptors for the polyketide starter units, the canonical SMILES for the acyl-moieties were retrieved from PubChem and three types of Extended-Connectivity Fingerprints (ECFPs) were calculated using RDKit^[17]^. Morgan Fingerprints (2048 bits) and RDKit topological fingerprints were calculated using *GetMorganFingerprintAsBitVect* and *GetRDKitFPGenerator().GetFingerprint*, respectively, from *AllChem* module. MACCS keys were generated using the *Chem.MACCSkeys* module. Embeddings for enzymes were derived from full-length sequences with the pre-trained ProtT5-XL-UniRef50 model by ProtTrans^[18]^ using the provided *prott5_embedder.py* script and averaged per-protein. The final enzyme:substrate feature vectors were obtained by concatenation of an enzyme embedding and a substrate ECFP. ML models were trained using scikit-learn 1.5.0^[19]^ with default hyperparameters using 0.7/ 0.3 *StratifiedShuffleSplit* for training/ testing purposes. The model performance was assessed by calculating the average accuracy, area under the receiver operating characteristic curve (AUROC), recall, precision, and F1 score in 100 runs with a random seed.

For building a descriptive ML model, the crystal structures of ORAS (type III pentaketide synthase from *Neurospora crassa,* PDB: 3EUT) and MsCHS (chalcone synthase from *Medicago sativa* (PDB: 1CGK) were used as templates. Residues within 5 Å of eicosanoic acid in 3EUT and within 6 Å of the catalytic cysteine and naringenin in 1CGK were selected, and the corresponding positions in the 31 active fungal T3PKSs were retrieved from an MSA. 12 fully conserved positions were removed, and 88 molecular descriptors were calculated using the peptides.py package – a python realisation of the Peptides R package^[20]^. The final feature vectors for each enzyme/ substrate pair were obtained by concatenating amino acid molecular descriptors with substrate MACCS Keys and had the following dimensions: {41 amino acids x (88 molecular descriptors) + 167 MACCS Keys}. The Decision tree model in scikit-learn 1.5.0^[19]^ was trained and assessed as described above for the predictive model. The model feature importances were extracted using the default *feature_importances_* function and summed up for each residue. The structure models of fungal T3PKSs were obtained using the local installation of AlphaFold 2^[21]^ at the Hábrók high performance computing cluster (University of Groningen). Data visualisation was performed using *ggplot2*^[22]^ *ComplexHeatmap*^[23]^ R packages, and PyMOL. The code used to train machine learning models and visualise gene neighbourhoods of the T3PKSs can be found at: <https://github.com/denisovss/type_III_PKS>. A step-by-step user guide is furthermore available in an accompanying manuscript (manuscript submitted).

## Supporting Tables

#### **Table S1**. List of putative fungal T3PKSs selected for experimental characterisation.

| **Name** | **JGI accession** | **Uniprot** | **Organism** | **Genome reference** |
| --- | --- | --- | --- | --- |
| AastPKS | Aspaste1\|20971 | - | [*Aspergillus astellatus*](https://mycocosm.jgi.doe.gov/Aspaste1/Aspaste1.info.html) | * |
| AcaePKS | Aspcae1\|160373 | A0A5N6ZX19 | [*Aspergillus caelatus CBS 763.97*](https://mycocosm.jgi.doe.gov/Aspcae1/Aspcae1.home.html) | ^[24]^ |
| AcosPKS | Aspcos1\|217105 | - | [*Aspergillus costaricaensis* CBS 115574](https://mycocosm.jgi.doe.gov/Aspcos1/Aspcos1.home.html) | ^[25]^ |
| AgalPKS | Aspgal1\|471214 | - | [*Aspergillus galapagensis CBS 117522*](https://mycocosm.jgi.doe.gov/Aspgal1/Aspgal1.home.html) | * |
| AiizPKS | Aspiiz1\|328085 | - | [*Aspergillus iizukae* CBS 541.69](https://mycocosm.jgi.doe.gov/Aspiiz1/Aspiiz1.info.html) | * |
| AlupPKS1 | Asplup1\|182290 | - | [Aspergillus luppii CBS 653.74](https://mycocosm.jgi.doe.gov/Asplup1/Asplup1.info.html) | * |
| AlupPKS2 | Asplup1\|227097 | - | [*Aspergillus luppii* CBS 653.74](https://mycocosm.jgi.doe.gov/Asplup1/Asplup1.info.html) | * |
| AneoPKS | Aspneof1\|275638 | - | [*Aspergillus neoflavipes* CBS 260.73](https://mycocosm.jgi.doe.gov/Aspneof1/Aspneof1.home.html) | * |
| AserPKS1 | Aspser1\|206060 | - | [*Aspergillus sergii* CBS 130017](https://mycocosm.jgi.doe.gov/Aspser1/Aspser1.home.html) | ^[24]^ |
| AserPKS2 | Aspser1\|218351 | - | [*Aspergillus sergii* CBS 130017](https://mycocosm.jgi.doe.gov/Aspser1/Aspser1.home.html) | ^[24]^ |
| AsesPKS | Aspses1\|335127 | - | [*Aspergillus sesamicola* CBS 137324](https://mycocosm.jgi.doe.gov/Aspses1/Aspses1.info.html) | * |
| AtamPKS1 | Asptam1\|307157 | A0A5N6VBQ0 | [*Aspergillus tamarii* CBS 117626](https://mycocosm.jgi.doe.gov/Asptam1/Asptam1.home.html) | ^[24]^ |
| AtamPKS2 | Asptam1\|310896 | A0A5N6V0I9 | *Aspergillus tamarii* CBS 117626 | ^[24]^ |
| AthePKS | Aspth1\|366720 | - | [*Aspergillus thesauricus* IBT 34227](https://mycocosm.jgi.doe.gov/Aspth1/Aspth1.info.html) | * |
| AtriPKS | Asptri1\|208758 | - | [*Aspergillus trinidadensis* IBT 32571](https://mycocosm.jgi.doe.gov/Asptri1/Asptri1.info.html) | * |
| AwakPKS | Aspwak1\|463812 | - | [*Aspergillus waksmanii* IBT 31900](https://mycocosm.jgi.doe.gov/Aspwak1/Aspwak1.info.html) | * |
| BiscPKS | Biscog1\|570470 | - | [*Biscogniauxia* sp. FL1348](https://mycocosm.jgi.doe.gov/Biscog1/Biscog1.info.html) | ^[26]^ |
| CadPKS | Cadsp1\|525675 | A0A2V1CPT9 | [*Cadophora* sp. DSE1049](https://mycocosm.jgi.doe.gov/Cadsp1/Cadsp1.info.html) | ^[27]^ |
| CcauPKS | Cercau1\|488391 | A0AA39U5E8 | [*Cercophora caudata CBS 606.72*](https://mycocosm.jgi.doe.gov/Cercau1/Cercau1.home.html) | ^[28]^ |
| CfunPKS | Capfu1\|150222 | - | [*Capronia fungicola CBS 614.96*](https://mycocosm.jgi.doe.gov/Capfu1/Capfu1.home.html) | * |
| CglobPKS | Chagl1\|381215 | - | [*Chaetomium tenue* MPI-SDFR-AT-0079 v1.0](https://mycocosm.jgi.doe.gov/Chagl1/Chagl1.info.html) | ^[29]^ |
| DdecPKS | Daldec1\|343198 | - | [*Daldinia decipiens* CBS 113046](https://mycocosm.jgi.doe.gov/Daldec1/Daldec1.info.html) | ^[26]^ |
| DhelPKS | Diahe1\|3921 | A0A2P5I7D7 | [*Diaporthe helianthi* str. 7/96](https://mycocosm.jgi.doe.gov/Diahe1/Diahe1.info.html) | ^[30]^ |
| DliqPKS | - | A0A7T8G346 | *Diaporthe liquidambaris* | - |
| FerePKS | Foner1\|4724 | A0A178ZV18 | [*Fonsecaea erecta* CBS 125763](https://mycocosm.jgi.doe.gov/Foner1/Foner1.home.html) | ^[31]^ |
| FmanPKS | Fusma1\|10567 | - | [*Fusarium mangiferae* MRC7560](https://mycocosm.jgi.doe.gov/Fusma1/Fusma1.info.html) | ^[32]^ |
| HargPKS1 | Hyparg1\|454749 | - | [*Hypoxylon argillaceum* CBS 527.63](https://mycocosm.jgi.doe.gov/Hyparg1/Hyparg1.home.html) | ^[26]^ |
| HargPKS2 | Hyparg1\|272596 | - | [*Hypoxylon argillaceum* CBS 527.63](https://mycocosm.jgi.doe.gov/Hyparg1/Hyparg1.home.html) | ^[26]^ |
| HypPKS | HyNC0597_1\|672645 | - | [*Hypoxylon* sp. NC0597](https://mycocosm.jgi.doe.gov/HyNC0597_1/HyNC0597_1.info.html) | ^[26]^ |
| MoryPKS | Magor1\|2848 | G4MSX3 | [*Pyricularia oryzae* 70-15](https://mycocosm.jgi.doe.gov/Magor1/Magor1.info.html) | ^[33]^ |
| MpolPKS | Mycpol1\|1099419 | - | [*Mycena polygramma* CBHHK137](https://mycocosm.jgi.doe.gov/Mycpol1/Mycpol1.info.html) | ^[34]^ |
| PansPKS | Podan3\|210066 | B2B3U6 | [*Podospora anserina S mat+*](https://mycocosm.jgi.doe.gov/Podan3/Podan3.home.html) | ^[35]^ |
| PficPKS | Pesfi1\|10058 | W3WYM6 | [*Pestalotiopsis fici* W106-1](https://mycocosm.jgi.doe.gov/Pesfi1/Pesfi1.info.html) | ^[36]^ |
| PflaPKS | Penfla1\|6890 | A0A1V6SK38 | [*Penicillium flavigenum* IBT 14082](https://mycocosm.jgi.doe.gov/Penfla1/Penfla1.home.html) | ^[37]^ |
| PtriPKS | - | A0A2W1FIY5 | [*Pyrenophora tritici-repentis*](https://fungi.ensembl.org/Pyrenophora_triticirepentis/Info/Index) | ^[38]^ |
| PverPKS | Psever1\|4162 | A0A1B8GPR4 | [*Pseudogymnoascus verrucosus* UAMH 10579](https://mycocosm.jgi.doe.gov/Psever1/Psever1.info.html) | ^[39]^ |
| SinsPKS | Spoin1\|227 | A0A162JF80 | [*Sporothrix insectorum* RCEF 264](https://mycocosm.jgi.doe.gov/Spoin1/Spoin1.info.html) | ^[40]^ |
| TtonPKS | Trito1\|844 | - | [*Trichophyton tonsurans* CBS 112818](https://mycocosm.jgi.doe.gov/Trito1/Trito1.home.html) | ^[41]^ |
| VmalPKS | - | A0A194VDY8 | [*Valsa mali* var. pyri](https://fungi.ensembl.org/Valsa_mali_var_pyri_gca_000813385/Info/Index) | ^[42]^ |
| XacuPKS1 | Xylacu1\|452307 | - | [*Xylaria acuta* CBS 122032](https://mycocosm.jgi.doe.gov/Xylacu1/Xylacu1.info.html) | ^[26]^ |
| XacuPKS2 | Xylacu1\|516844 | - | [*Xylaria acuta* CBS 122032](https://mycocosm.jgi.doe.gov/Xylacu1/Xylacu1.info.html) | ^[26]^ |
| XylPKS | XyFL1272_2\|442241 | - | [*Xylariaceae* sp. FL1272](https://mycocosm.jgi.doe.gov/XyFL1272_2/XyFL1272_2.info.html) | ^[26]^ |

* = these whole genome sequencing projects were executed by the US Department of Energy Joint Genome Institute https://www.jgi.doe.gov/ in collaboration with the user community.

#### **Table S2**. List of T3PKSs from fungi, plants and bacteria with published activity data that were used in the ML validation experiments in this study.

| **Enzyme** | **Origin** | **Donor organism** | **Reference** |
| --- | --- | --- | --- |
| ORAS | fungal | *Neurospora crassa* | ^[43]^ |
| AnPKS | fungal | *Aspergillus niger CBS 513.88* | ^[44]^ |
| An-CsyA | fungal | *Aspergillus niger NRRL 328* | ^[45]^ |
| CsyA | fungal | *Aspergillus oryzae* | ^[46]^ |
| BPKS | fungal | *Botrytis cinerea* | ^[47]^ |
| Sl-PKS2 | fungal | *Sporotrichum laxum* | ^[48]^ |
| SmPKS | fungal | *Sordaria macrospora* | ^[49]^ |
| CtPKS | fungal | *Chaetomium thermophilum* | ^[49]^ |
| CsyB | fungal | *Aspergillus oryzae* | ^[50]^ |
| NuPKS | fungal | *Naganishia uzbekistanensis* | ^[51]^ |
| FiPKS | fungal | *Fusarium incarnatum* | ^[52]^ |
| QNS-Marmelos | plant | *Aegle marmelos* | ^[53]^ |
| QNS-Microcarpa | plant | *Citrus microcarpa* | ^[54]^ |
| ANS-Microcarpa | plant | *Citrus microcarpa* | ^[54]^ |
| BNS-Palmatum | plant | *Rheum palmatum* | ^[55–57]^ |
| ARAS1 | plant | *Oryza sativa* | ^[58]^ |
| ARAS2 | plant | *Oryza sativa* | ^[58]^ |
| Sg-RppA | bacterial | *Streptomyces griseus* | ^[59]^ |
| ArsB | bacterial | *Azotobacter vinelandii* | ^[60]^ |
| ArsC | bacterial | *Azotobacter vinelandii* | ^[60]^ |
| Ncs | bacterial | *Streptomyces clavuligerus* | ^[61]^ |
| BpsA | bacterial | *Bacillus subtilis* | ^[62]^ |
| PhlD | bacterial | *Pseudomonas fluorescens* | ^[63]^ |
| gcs | bacterial | *Streptomyces coelicolor* | ^[64]^ |
| Se-RppA | bacterial | *Saccharopolyspora erythraea* | ^[65]^ |
| Sc-RppA | bacterial | *Streptomyces coelicolor* | ^[66]^ |

#### **Table S3**. Low-resolution LC-MS analysis of the T3PKS reaction products detected during activity profiling with substrates **1-12** and machine learning validation experiments with substrates **13-24, 27** and **31**. High-resolution LC-MS/MS analysis of the products is shown in Figures S11-S60.

| **Starter substrate** | **Product** | **t_R_, min** | **Ion mode** | **Detected *m/z*** | **Ketide number*** | **Cyclisation type*** |
| --- | --- | --- | --- | --- | --- | --- |
| benzoyl-CoA (**1**) | **1a** | 5.46 | ESI- | 187 | 3 | lactone |
|  | **1b** | 5.36 | ESI- | 229 | 4 | lactone |
| phenylacetyl-CoA (**2**) | **2a** | 5.57 | ESI- | 201 | 3 | lactone |
|  | **2b** | 5.47 | ESI- | 243 | 4 | lactone |
| N-methylanthraniloyl-CoA (**3**) | **3a** | 5.25 | ESI- | 174 | 2 | quinolone |
| p-coumaroyl-CoA (**4**) | **4a** | 5.25 | ESI- | 229 | 3 | lactone |
|  | **4b** | 5.18; 5.61 | ESI- | 271 | 4 | phloroglucinol |
| β-methylcrotonoyl-CoA (**5**) | **5a** | 5.36 | ESI+ | 167 | 3 | lactone |
|  | **5b** | 5.19 | ESI+ | 209 | 4 | lactone |
| acetyl-CoA (**6**) | **6a** | 0.97 | ESI+ | 127 | 3 | lactone |
| hexanoyl-CoA (**7**) | **7a** | 5.83 | ESI- | 181 | 3 | lactone |
|  | **7b** | 5.76 | ESI- | 223 | 4 | lactone |
|  | **7c** | 6.15 | ESI- | 265 | 5 | lactone |
|  | **7d** | 5.41 | ESI- | 307 | 6 | lactone |
| decanoyl-CoA (**8**) | **8a** | 6.72 | ESI- | 237 | 3 | lactone |
|  | **8b** | 6.64 | ESI- | 279 | 4 | lactone |
|  | **8c** | 6.95 | ESI- | 321 | 5 | lactone |
|  | **8d** | 6.89 | ESI- | 235 | 4 | resorcinol |
|  | **8e** | 6.68 | ESI- | 277 | 5 | resorcinol |
| myristoyl-CoA (**9**) | **9a** | 7.62 | ESI- | 293 | 3 | lactone |
|  | **9b** | 7.48 | ESI- | 335 | 4 | lactone |
|  | **9c** | 7.80 | ESI- | 377 | 5 | lactone |
|  | **9d** | 7.79 | ESI- | 291 | 4 | resorcinol |
|  | **9e** | 7.52 | ESI- | 333 | 5 | resorcinol |
| oleoyl-CoA (**10**) | **10a** | 6.14 | ESI- | 347 | 3 | lactone |
|  | **10b** | 5.86 | ESI+ | 391 | 4 | lactone |
|  | **10c** | 6.57 | ESI+ | 433 | 5 | lactone |
|  | **10d** | 6.52 | ESI+ | 347 | 4 | resorcinol |
|  | **10e** | 5.96 | ESI- | 387 | 5 | resorcinol |
| phytanoyl-CoA (**11**) | **11a** | 7.38 | ESI- | 377 | 3 | lactone |
|  | **11b** | 7.06 | ESI- | 419 | 4 | lactone |
| pentynoyl-CoA (**13**) | **13a** | 3.52 | ESI+ | 165 | 3 | lactone |
|  | **13b** | 3.01 | ESI+ | 207 | 4 | lactone |
| cyclohexanoyl-CoA (**14**) | **14a** | 5.79 | ESI+ | 195 | 3 | lactone |
|  | **14b** | 5.69 | ESI+ | 237 | 4 | lactone |
| 4-cyclohexyl-4-oxobutanoyl-CoA (**15**) | **15a** | 5.80 | ESI+ | 251 | 3 | lactone |
| 4-cyclopentyl-4-  oxobutanoyl-CoA (**16**) | **16a** | 5.59 | ESI+ | 237 | 3 | lactone |
| thiazole-4-carboxylyl-CoA (**17**) | **17a** |  | ESI- | 194 | 3 | lactone |
| furoyl-CoA (**18**) | **18a** | 5.09 | ESI+ | 179 | 3 | lactone |
| trans-2-phenylcyclopropane-  1-carboxylyl-CoA (**19**) | **19a** | 5.84 | ESI- | 227 | 3 | lactone |
|  | **19b** | 5.75 | ESI- | 269 | 4 | lactone |
| cinnamoyl-CoA (**20**) | **20a** | 5.80 | ESI+ | 215 | 3 | lactone |
|  | **20b** | 5.67 | ESI+ | 257 | 4 | phloroglucinol |
| 2-chlorophenylacetyl-CoA (**21**) | **21a** | 5.77 | ESI- | 235 | 3 | lactone |
|  | **21b** | 5.65 | ESI- | 277 | 4 | lactone |
| 3-fluorobenzoyl-CoA (**22**) | **22a** | 5.55 | ESI- | 205 | 3 | lactone |
|  | **22b** | 5.45 | ESI- | 247 | 4 | lactone |
| 4-fluorophenylacetyl-CoA (**23**) | **23a** | 5.62 | ESI- | 219 | 3 | lactone |
|  | **23b** | 5.54 | ESI- | 261 | 4 | lactone |
| 2-amino-5-chlorobenzoyl-CoA (**24**) | **24a** | 5.40 | ESI+ | 196 | 2 | quinolone |
| butyryl-CoA (**27**) | **27a** | 5.12 | ESI+ | 155 | 3 | lactone |
| isovaleryl-CoA (**31**) | **31a** | 5.50 | ESI+ | 169 | 3 | lactone |
|  |  |  |  |  |  |  |

t_R_ = retention time; * = putative

#### **Table S4**. List of plasmids used in this study.

| **Construct name** | **Backbone** | **Enzyme encoded** | **Source** |
| --- | --- | --- | --- |
| pET28a::6His-Os4CL | pET-28a(+) | Os4CL | ^[67]^ |
| pAGM22082_sfGFP1-10 | pAGM22082 | sfGFP1-10 | ^[10]^ |
| p70a::mcbR | p70a | mcbR | ^[68]^ |
| p70a::PhCHS-6His-GFP11 | p70a | PhCHS-GFP11 | This study |
| pET28a::PhCHS-6His-GFP11 | pET-28a(+) | PhCHS-GFP11 | This study |
| pET28a::AastPKS-6His | pET-28a(+) | AastPKS | This study |
| pET28a::AiizPKS-6His | pET-28a(+) | AiizPKS | This study |
| pET28a::AthePKS-6His | pET-28a(+) | AthePKS | This study |
| pET28a::CgloPKS-6His | pET-28a(+) | CgloPKS | This study |
| pET28a::DhelPKS-6His | pET-28a(+) | DhelPKS | This study |
| pET28a::FerePKS-6His | pET-28a(+) | FerePKS | This study |
| pET28a::HargPKS1-6His | pET-28a(+) | HargPKS1 | This study |
| pET28a::PficPKS-6His | pET-28a(+) | PficPKS | This study |
| pET28a::PhCHS-6His | pET-28a(+) | PhCHS | This study |
| pET28a::XacuPKS1-6His | pET-28a(+) | XacuPKS1 | This study |
| pET28a::AcosPKS-6His | pET-28a(+) | AcosPKS | This study |
| pET28a::TtonPKS-6His | pET-28a(+) | TtonPKS | This study |
| pET28a::HypPKS-6His | pET-28a(+) | HypPKS | This study |
| pET28a::6His-PqsA | pET-28a(+) | PqsA | This study |

## Supporting Figures


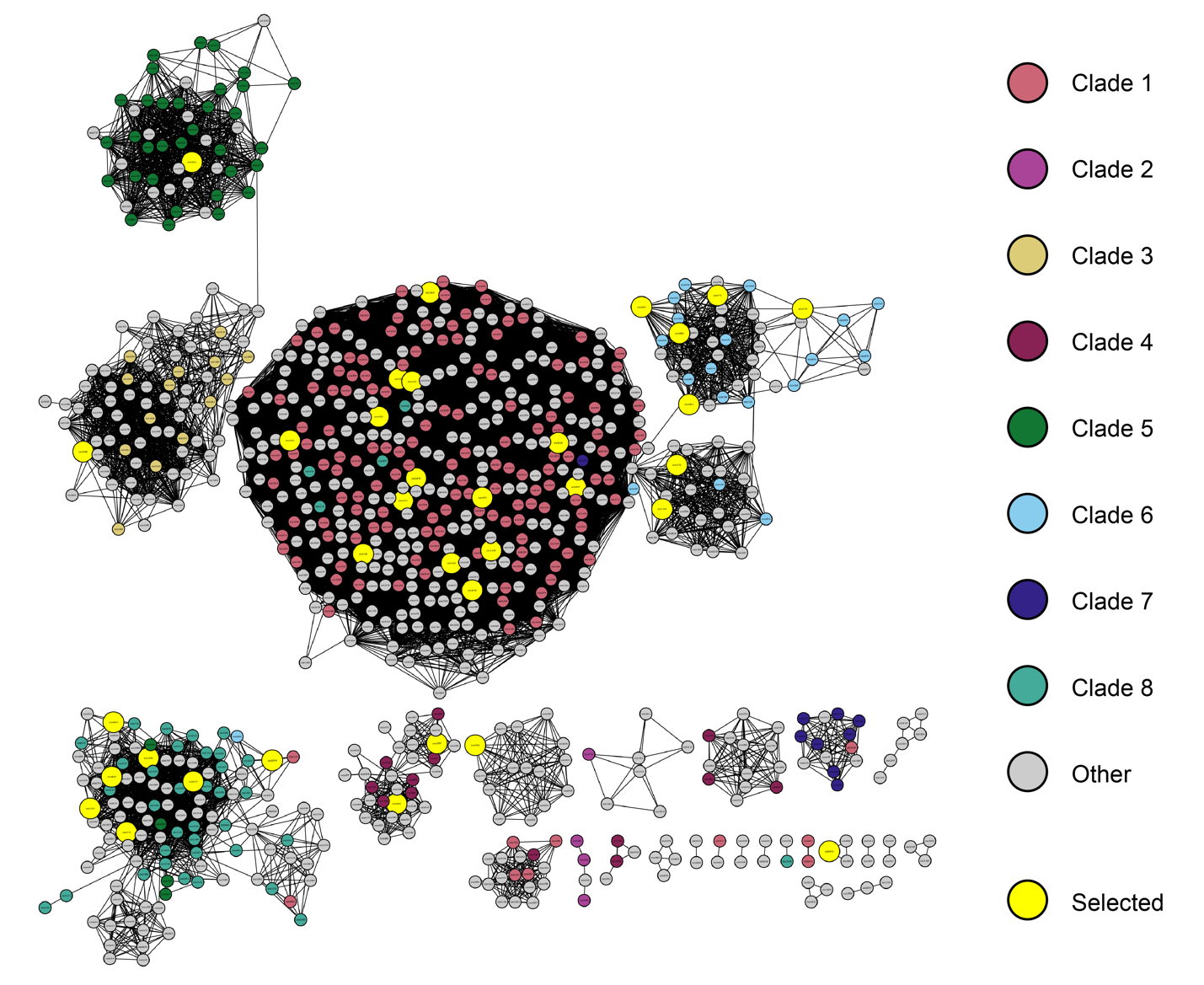


**Figure S1**. A sequence similarity network of the putative fungal T3PKSs mined from the Mycocosm database at 57% sequence identity cutoff. Each circle is a representative node grouping protein sequences with >95% sequence identity. The colours reflect phylogenetic clades proposed by Navarro-Muños and Collemare in a recent evolutionary analysis^[69]^.


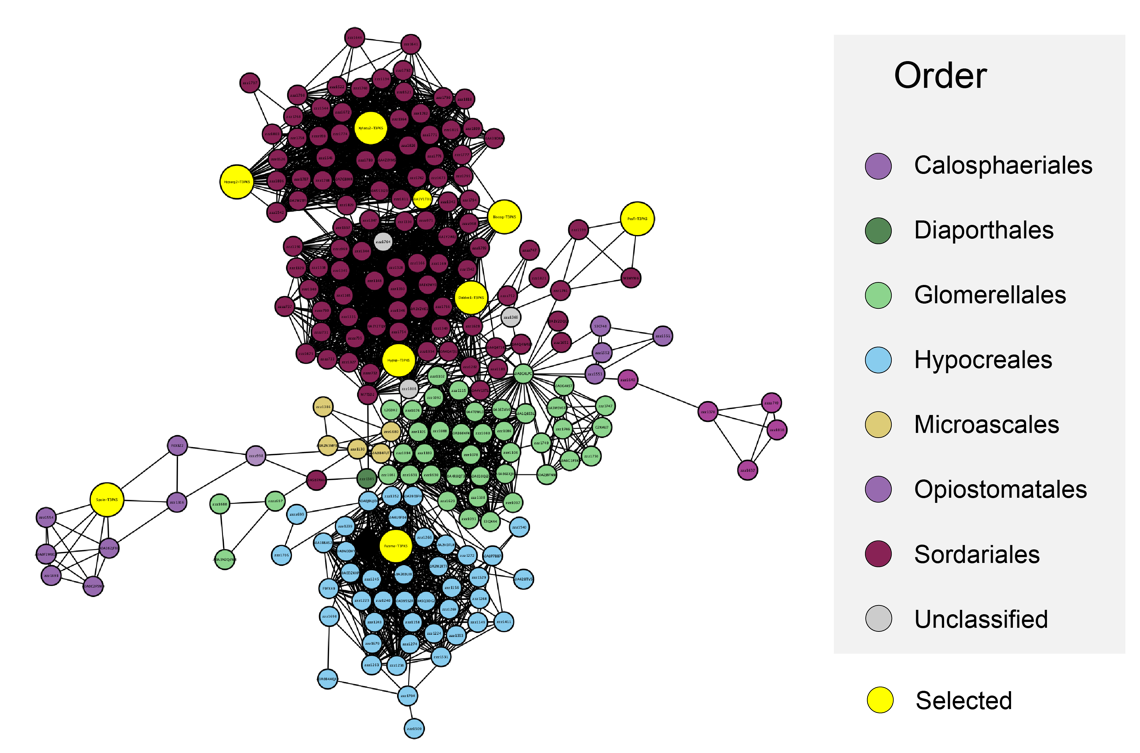


**Figure S2.** Cluster 1 of the sequence similarity network of fungal T3PKSs. The nodes are coloured based on the taxonomic order of the donor organisms.


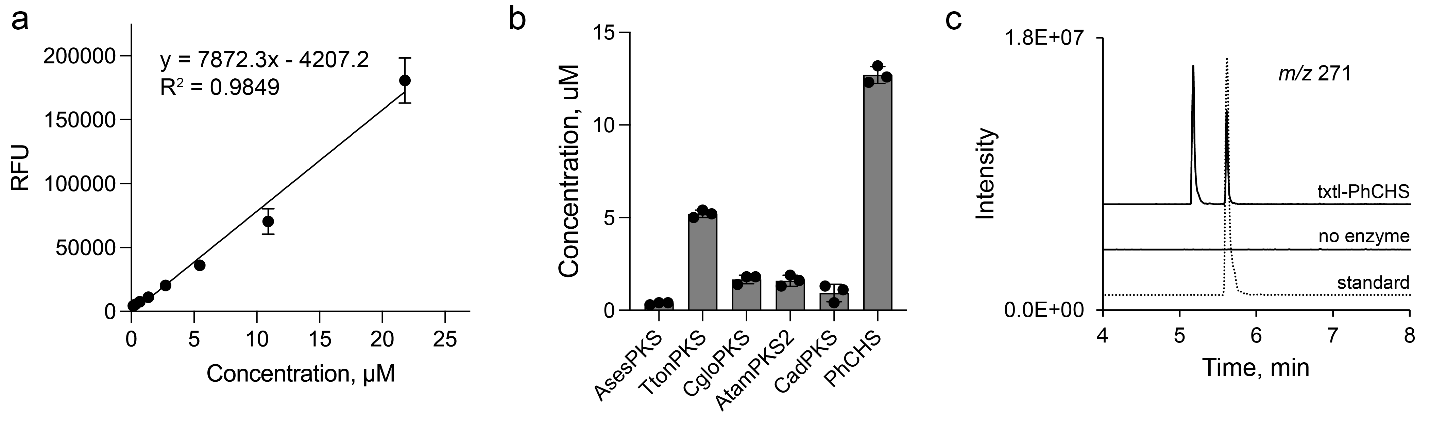


**Figure S3.** Benchmarking of the express-test workflow with PhCHS. a) Calibration plot of the purified PhCHS-GFP11 fusion standard protein in TNG buffer used for quantifying soluble cell-free expressed protein; RFU - relative fluorescence units; data points and error bars reflect mean ± SD; n=3. b) Comparison of enzyme concentrations across different batches of cell-free expression reactions using PCR-amplified linear templates. Enzyme concentrations were determined using the split-GFP assay; histograms and error bars reflect mean ± SD; n=3. c) Low-resolution LC-MS analysis of the EtOAc extract of the enzymatic reaction of the cell-free expressed PhCHS (txtl-PhCHS) with substrate **4**; extracted ion chromatogram of the predicted *m/z* of naringenin chalcone and naringenin (271, negative mode). Y-axis shows relative ion abundance. Dotted line represents the authentic standard of naringenin.


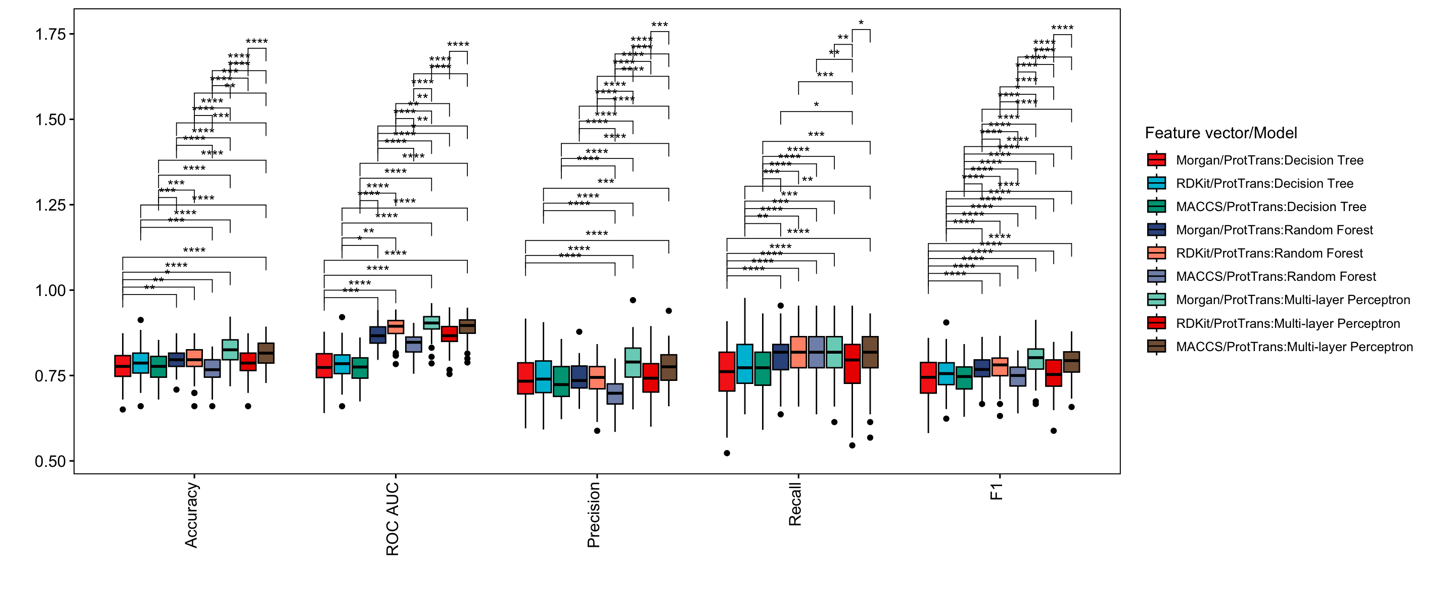


**Figure S4.** Boxplots of ML performance metrics for the tested ML algorithms and feature vectors obtained on the cell-free substrate activity dataset. Values are averaged from 100 datasets with random splits. * - *p* ≤ 0.05, ** - *p* ≤ 0.01, *** - *p* ≤ 0.001, **** - *p* ≤ 0.0001, ns is not shown for visibility.


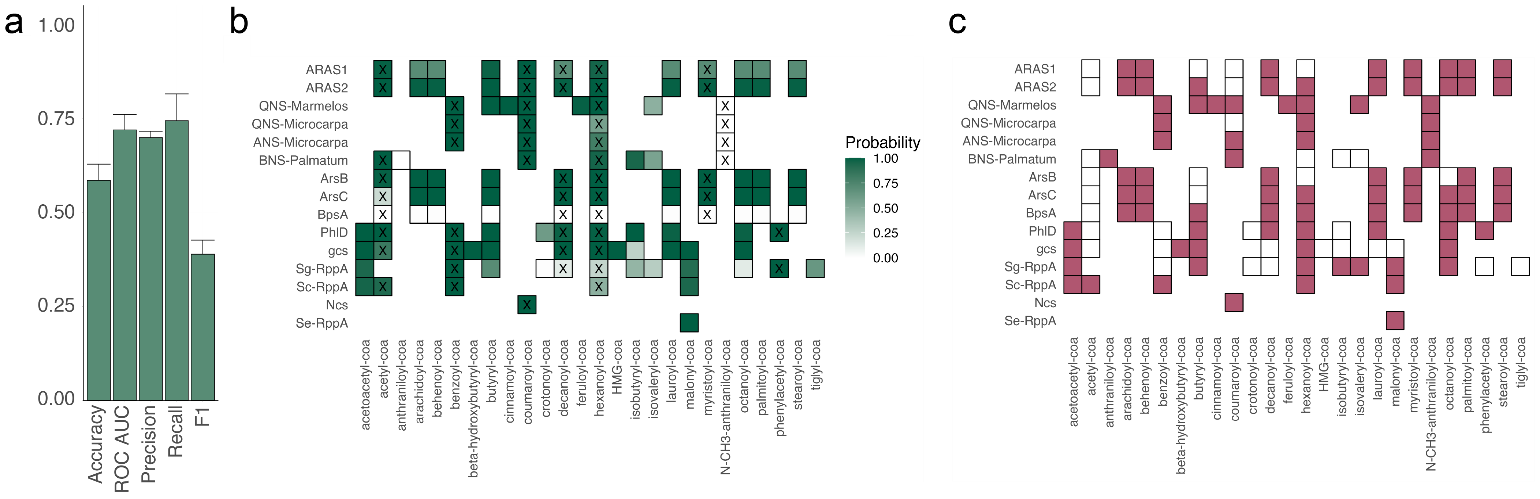


**Figure S5.** Testing of phylogenetic bias of the predictive ML model. a) ML performance metrics of the Multi-Layer Perceptron algorithm with ProtTrans-X5/MACCS Keys feature vectors obtained for enzyme-substrate pairs from plant and bacteria from literature (see Table S2). Predicted (b) and experimental (c) enzyme/substrate specificity of plant and bacterial T3PKSs. Substrates which were present in the training dataset are marked with X. Performance metrics and predicted enzyme/substrate specificity are averaged from 100 model runs with a random seed.


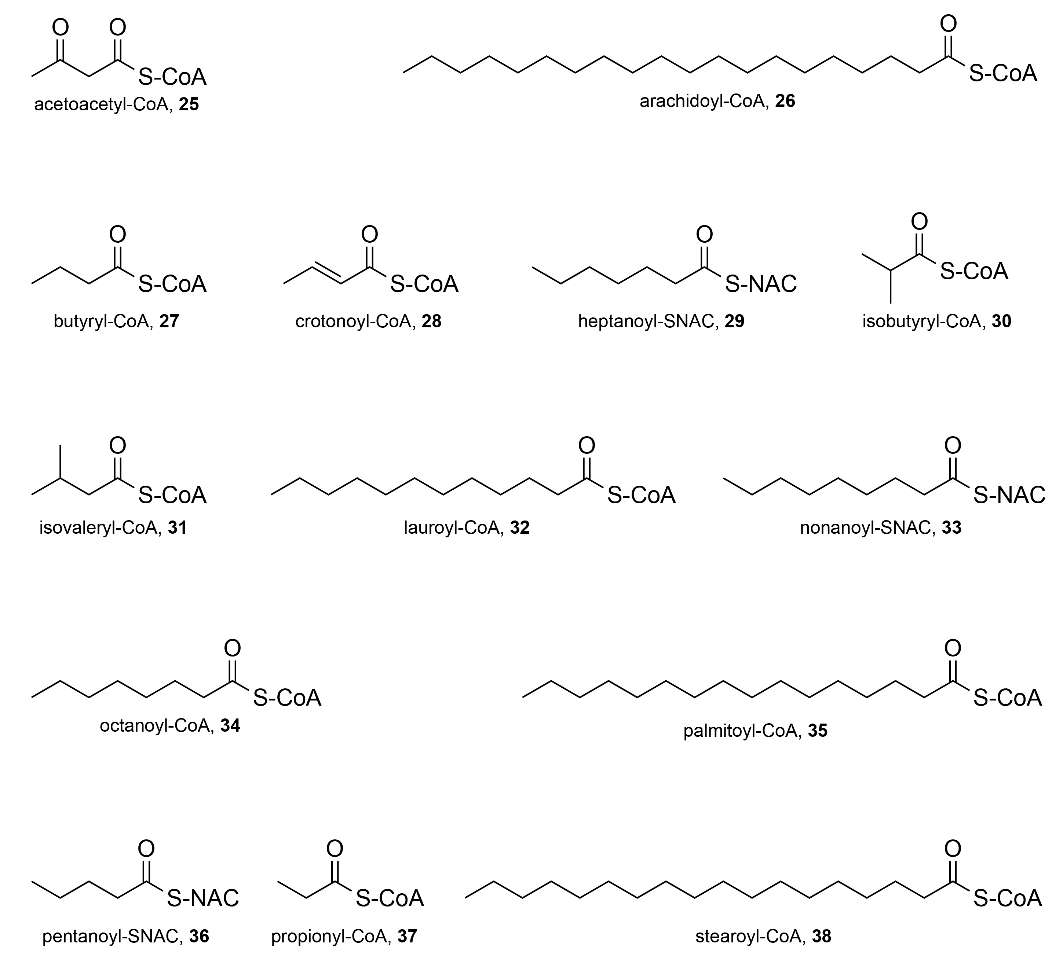


**Figure S6.** Structures of compounds **25-38** previously reported to be accepted by fungal T3PKSs. NAC = N-acetylcysteamine, a synthetic CoA analogue.


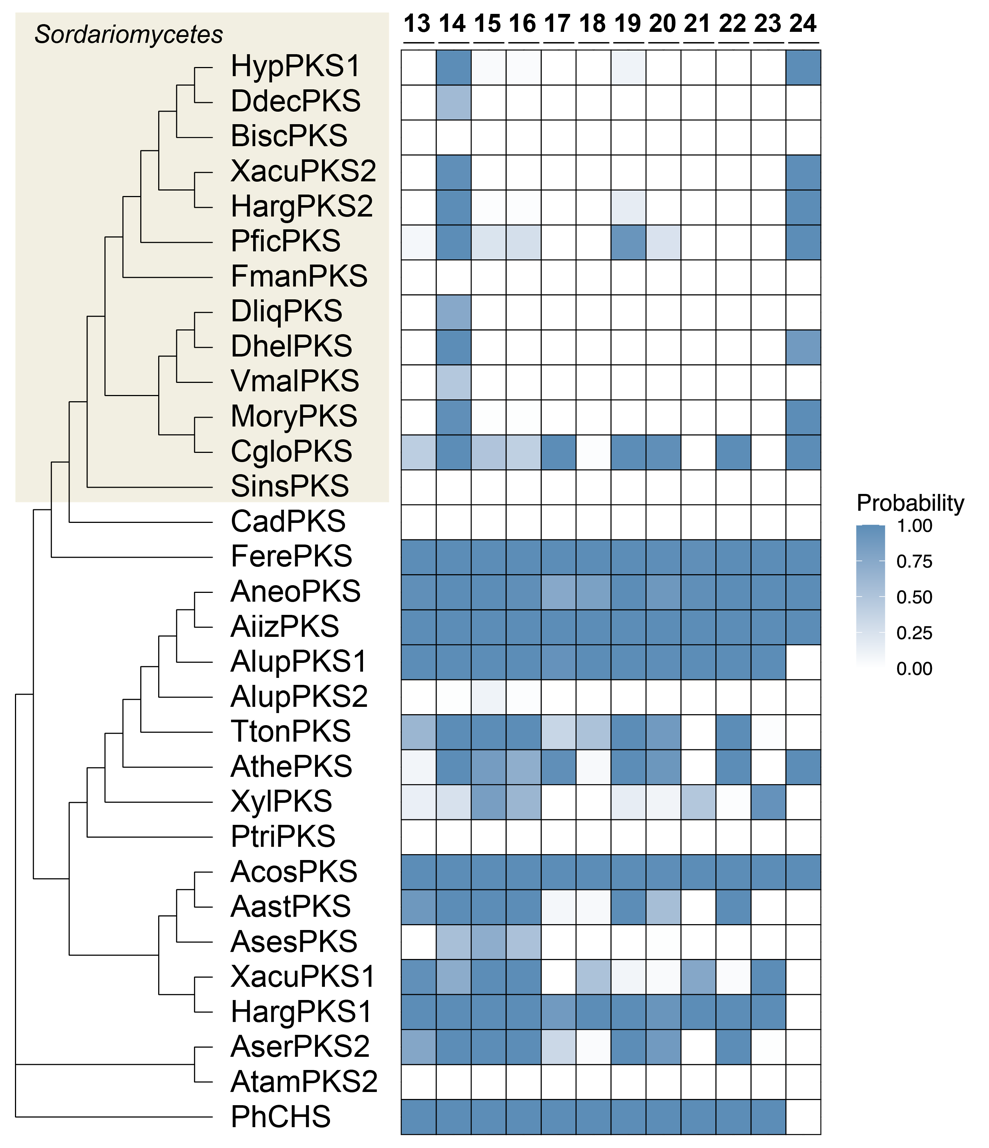


**Figure S7.** Enzyme/substrate specificity prediction for 31 active T3PKSs with substrates **13-24** by the Multi-Layer Perceptron algorithm with the ProtTrans-X5/MACCS Keys feature vectors. Predictions are averaged from 100 model runs with a random seed. Seven T3PKSs that were not expressed at detectable levels were excluded from the analysis.

**
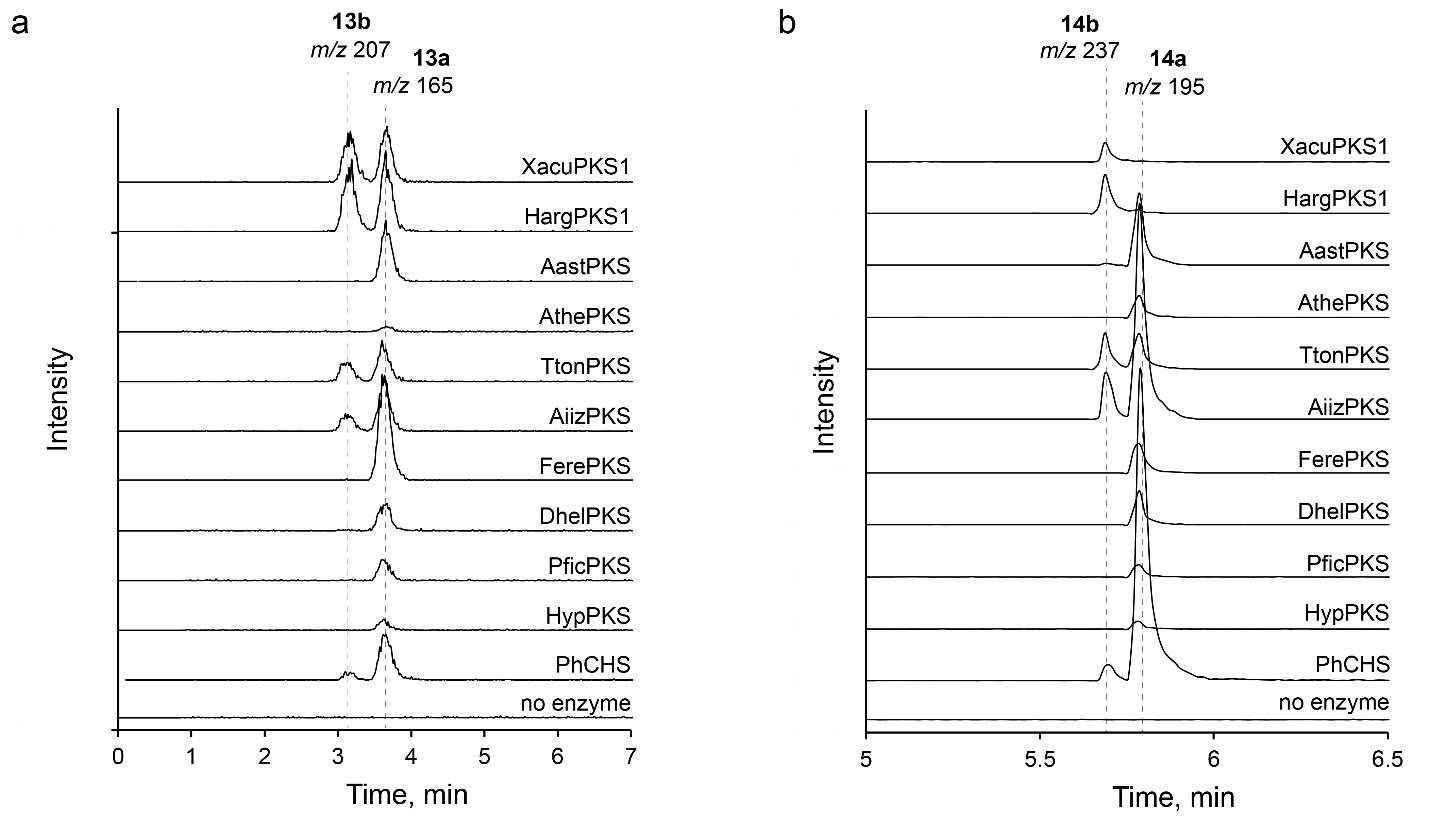

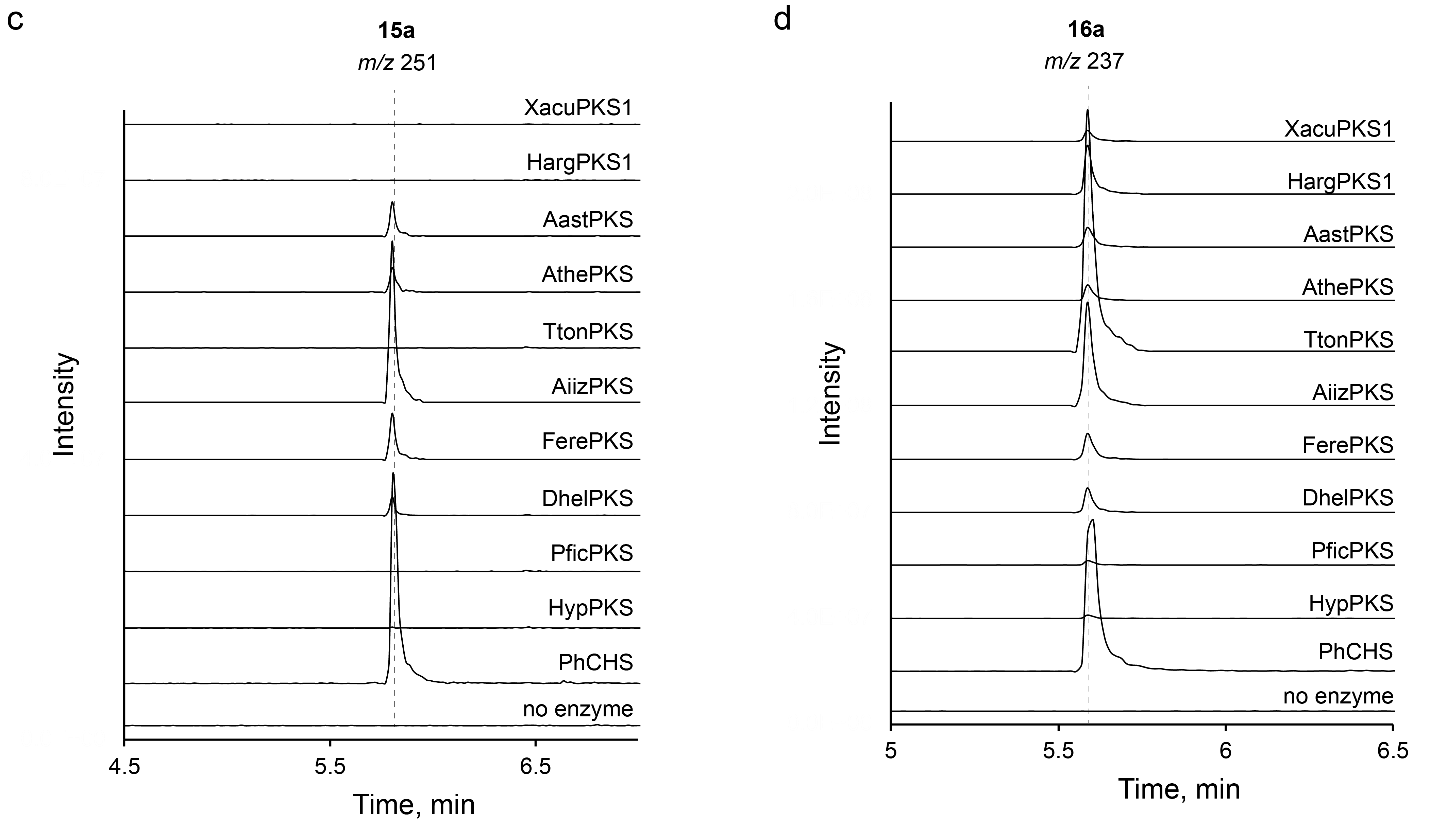

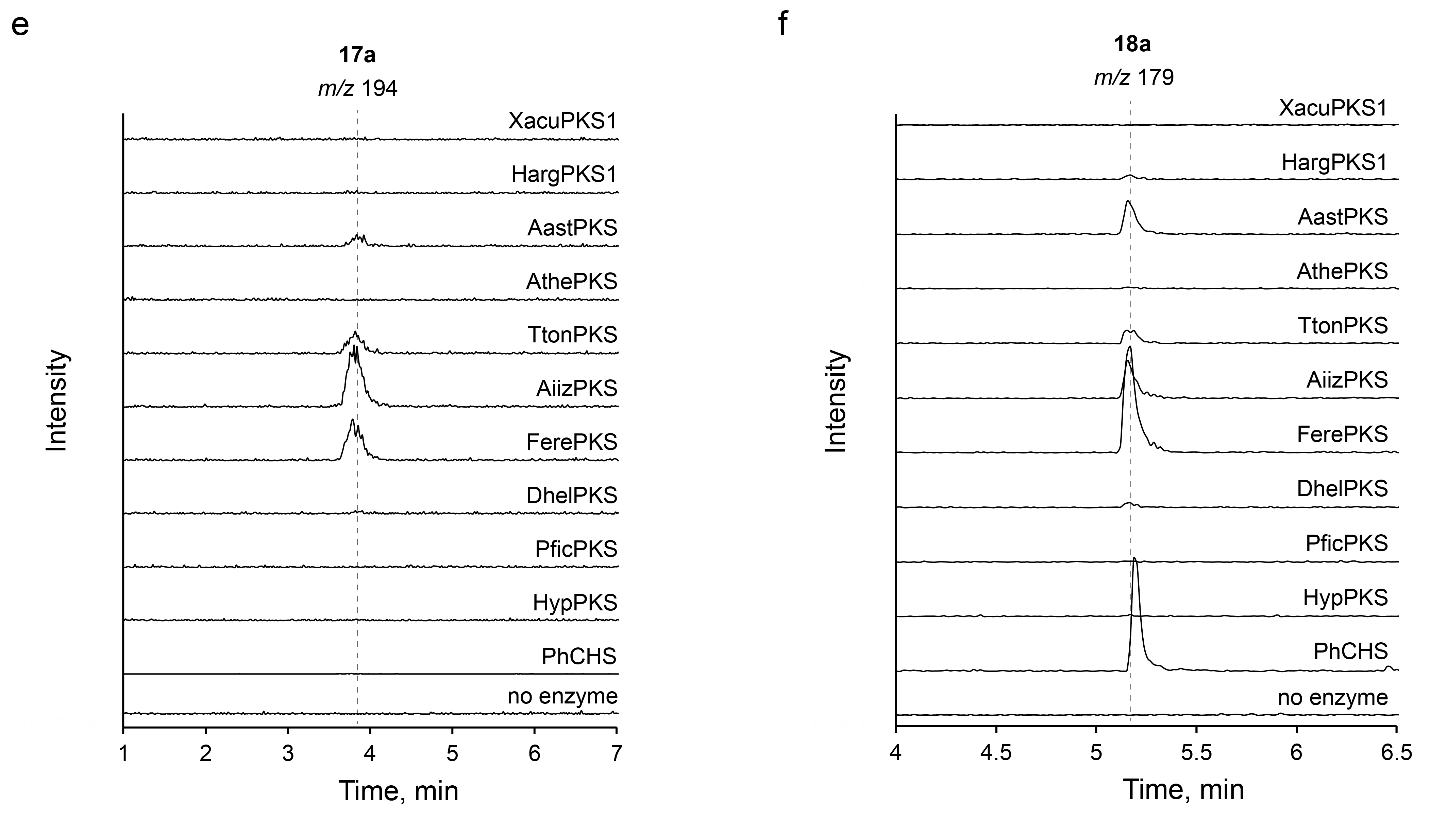

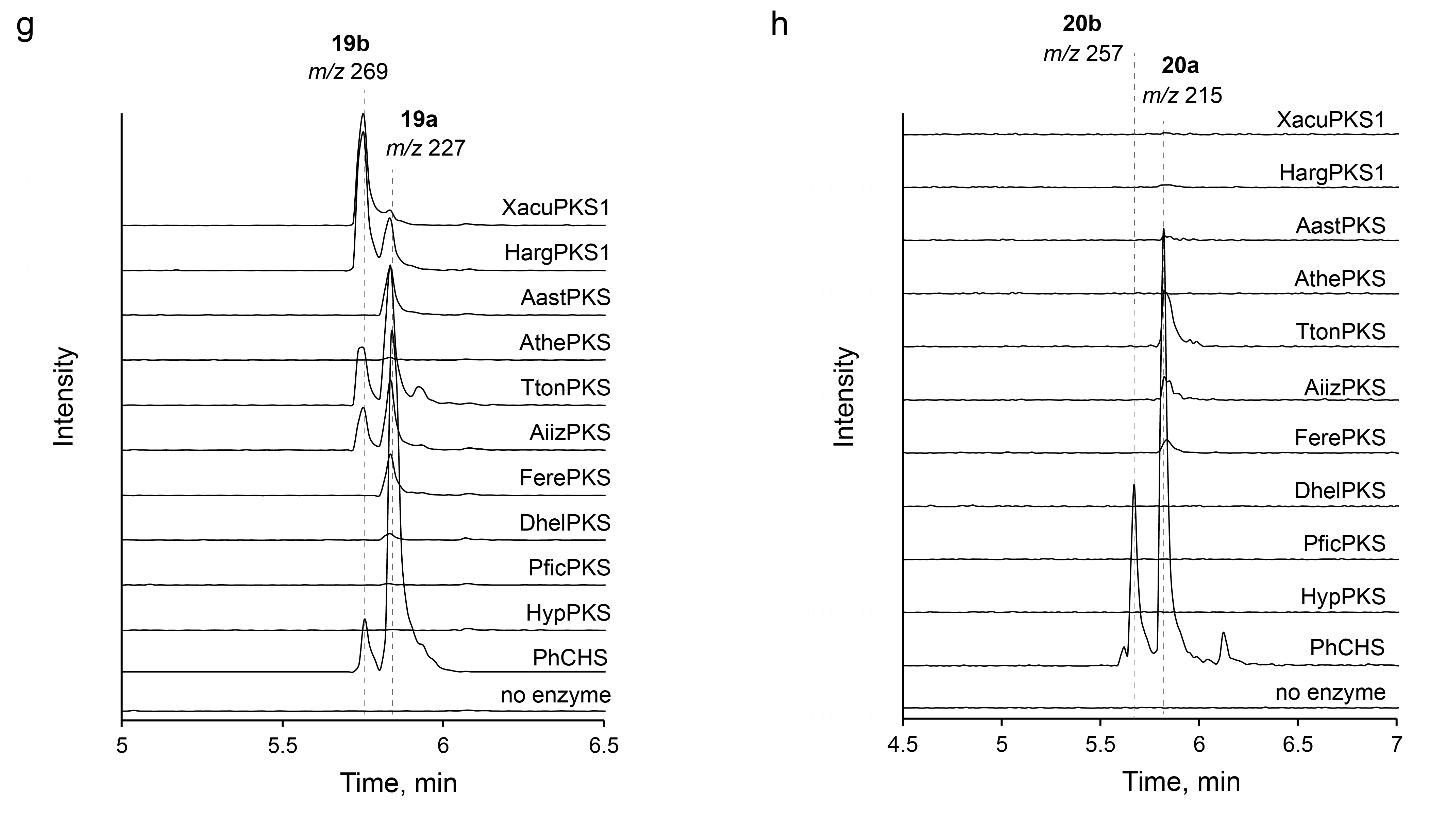

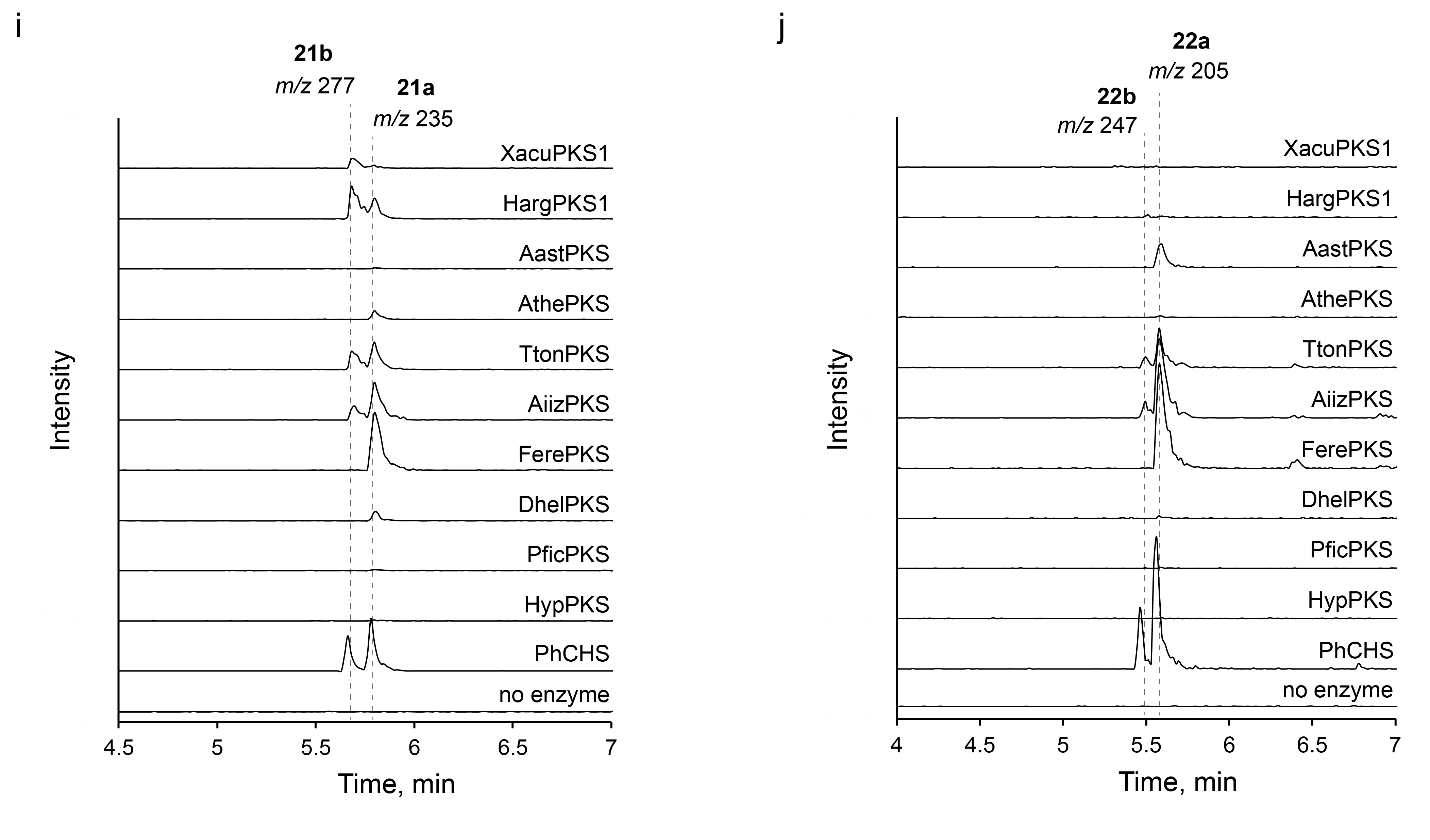

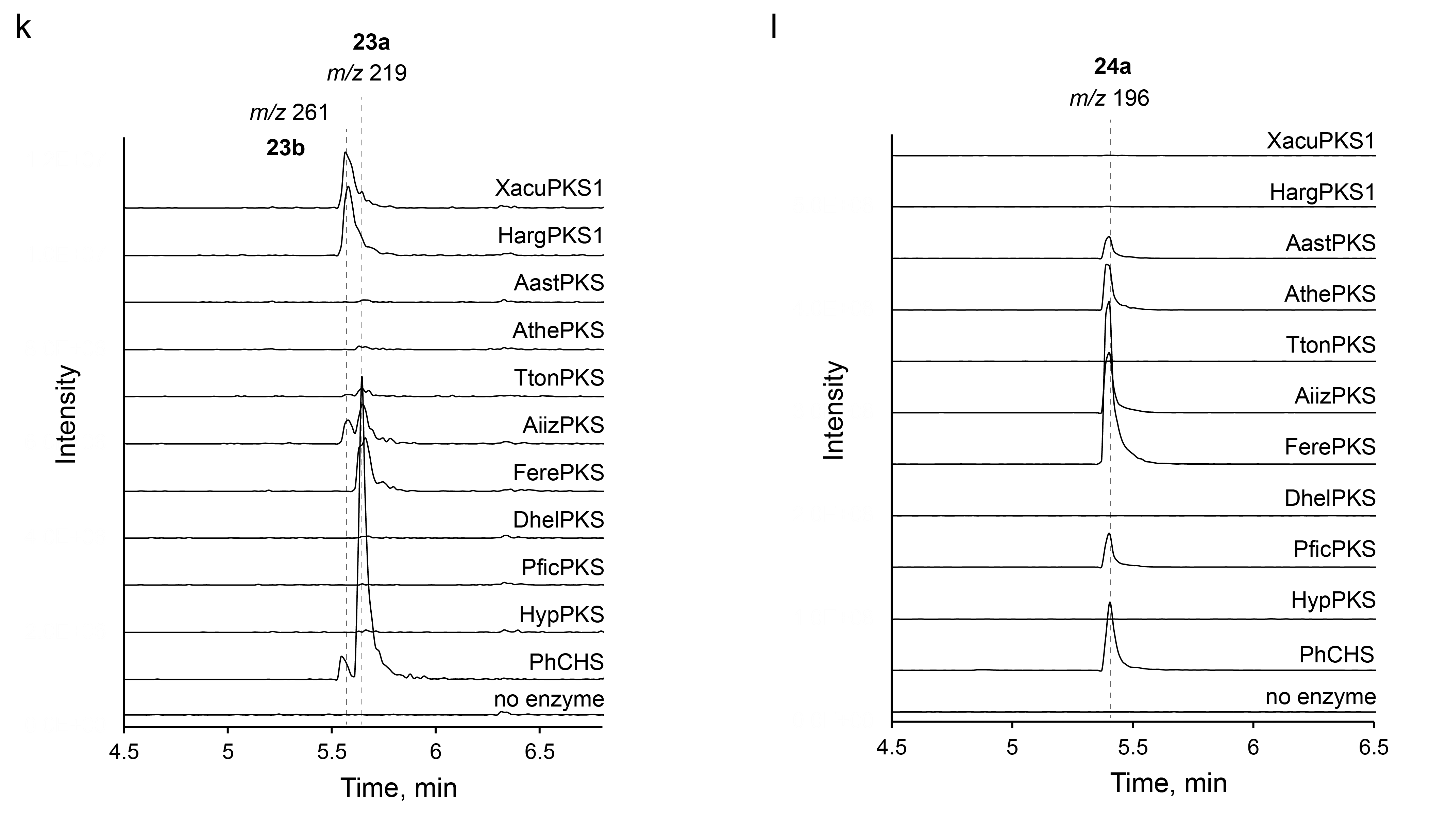
**

**Figure S8.** Extracted ion chromatograms in low-resolution LC-MS of reaction products of the purified fungal T3PKSs and PhCHS with substrates **13-24** compared to the "no enzyme" control. a) Substrate **13**; b) substrate **14**; c) substrate **15**; d) substrate **16**; e) substrate **17**; f) substrate **18**; g) substrate **19**; h) substrate **20**; i) substrate **21**; j) substrate **22**; k) substrate **23**; l) substrate **24**.

The following mass-to-charge ratios (*m/z*) were detected in positive ion mode: putative triketide pyrone from **13**, detected *m/z* = 165 (theoretical *m/z* = 165.0546, calculated for [C_9_H_9_O_3_]^+^); putative tetraketide pyrone from **13**, detected *m/z* = 207 (theoretical *m/z* = 207.0652, calculated for [C_11_H_11_O_4_]^+^); putative triketide pyrone from **14**, detected *m/z* = 195 (theoretical *m/z* = 195.1016, calculated for [C_11_H_15_O_3_]^+^); putative tetraketide pyrone from **14**, detected *m/z* = 237 (theoretical *m/z* = 237.1121, calculated for [C_13_H_17_O_4_]^+^); putative triketide pyrone from **15**, detected *m/z* = 251 (theoretical *m/z* = 251.1278, calculated for [C_14_H_19_O_4_]^+^); putative triketide pyrone from **16**, detected *m/z* = 237 (theoretical *m/z* = 237.1121, calculated for [C_13_H_17_O_4_]^+^); putative triketide pyrone from **18**, detected *m/z* = 179 (theoretical *m/z* = 179.0339, calculated for [C_9_H_7_O_4_]^+^); putative triketide pyrone from **20**, detected *m/z* = 215 (theoretical *m/z* = 215.0703, calculated for [C_13_H_11_O_3_]^+^); putative tetraketide phloroglucinol from **20**, detected *m/z* = 257 (theoretical *m/z* = 257.0808, calculated for [C_15_H_13_O_4_]^+^); putative diketide quinolone from **24**, detected *m/z* = 196 (theoretical *m/z* = 196.0160, calculated for [C_9_H_7_ClNO_2_]^+^).

The following mass-to-charge ratios (*m/z*) were detected in negative ion mode: putative triketide pyrone from **17**, detected *m/z* = 194 (theoretical *m/z* = 193.9917, calculated for [C_8_H_4_NO_3_S]^-^); putative triketide pyrone from **19**, detected *m/z* = 227 (theoretical *m/z* = 227.0714, calculated for [C_14_H_11_O_3_]^-^); putative tetraketide pyrone from **19**, detected *m/z* = 269 (theoretical *m/z* = 269.0819, calculated for [C_16_H_13_O_4_]^-^); putative triketide pyrone from **21**, detected *m/z* = 235 (theoretical *m/z* = 235.0167, calculated for [C_12_H_8_ClO_3_]^-^); putative tetraketide pyrone from **21**, detected *m/z* = 277 (theoretical *m/z* = 277.0273, calculated for [C_14_H_10_ClO_4_]^-^); putative triketide pyrone from **22**, detected *m/z* = 205 (theoretical *m/z* = 205.0306, calculated for [C_11_H_6_FO_3_]^-^); putative tetraketide pyrone from **22**, detected *m/z* = 247 (theoretical *m/z* = 247.0412, calculated for [C_13_H_8_FO_4_]^-^); putative triketide pyrone from **23**, detected *m/z* = 219 (theoretical *m/z* = 219.0463, calculated for [C_12_H_8_FO_3_]^-^); putative tetraketide pyrone from **23**, detected *m/z* = 261 (theoretical *m/z* = 261.0569, calculated for [C_14_H_10_FO_4_]^-^).


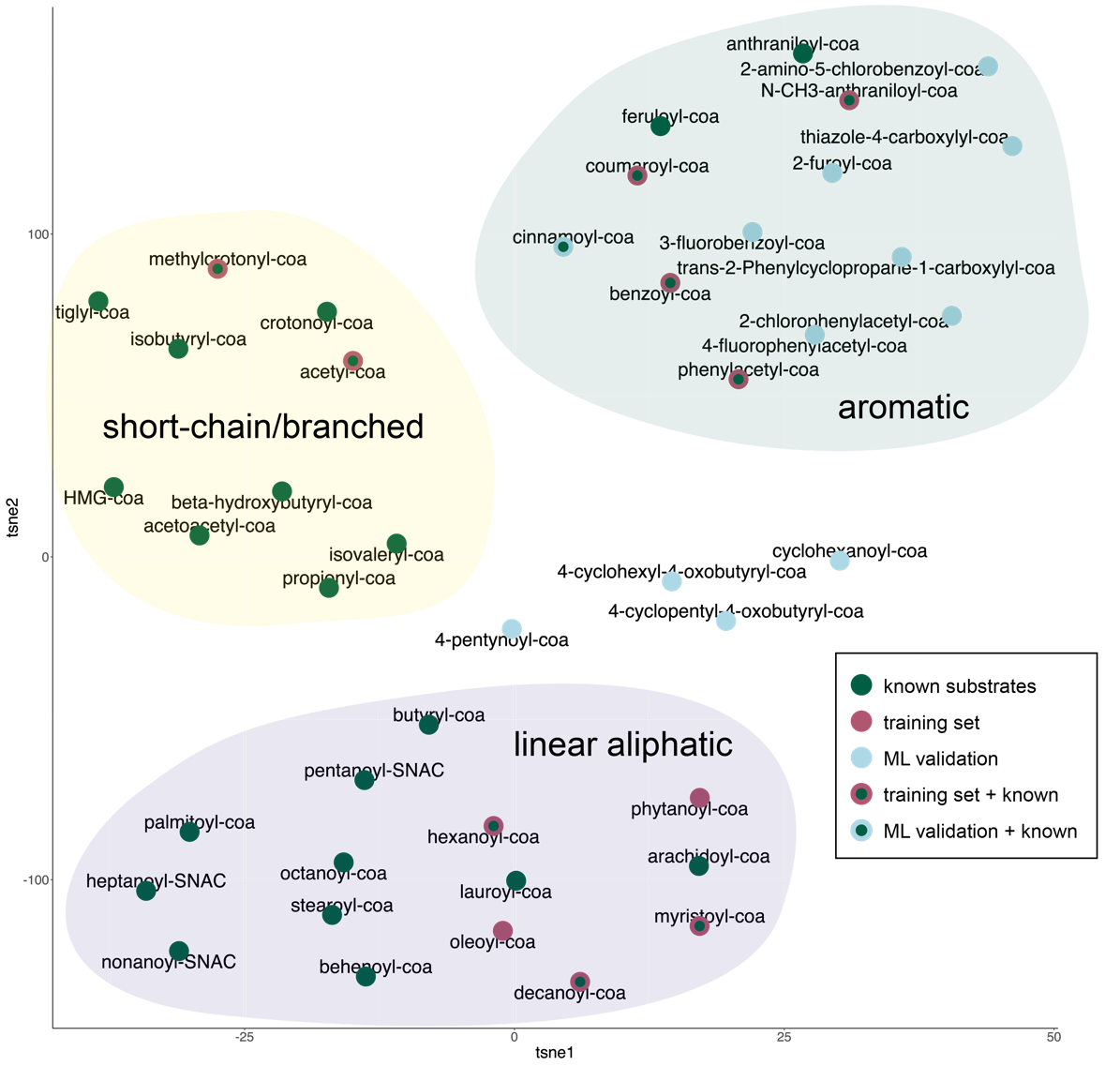


**Figure S9.** Representation of the chemical space of T3PKS substrates obtained by t-SNE dimensionality reduction of a substrate similarity matrix. The matrix was obtained by calculating pairwise similarity of the substrates’ MACCS Keys using the RDKit *DataStructs.DiceSimilarity* function. Dimensionality reduction was performed using scikit-learn *manifold.TSNE* with perplexity 10. Clusters containing linear, short chain/branched and aromatic CoA thioesters are highlighted. Nodes are coloured based on the source of the substrate.


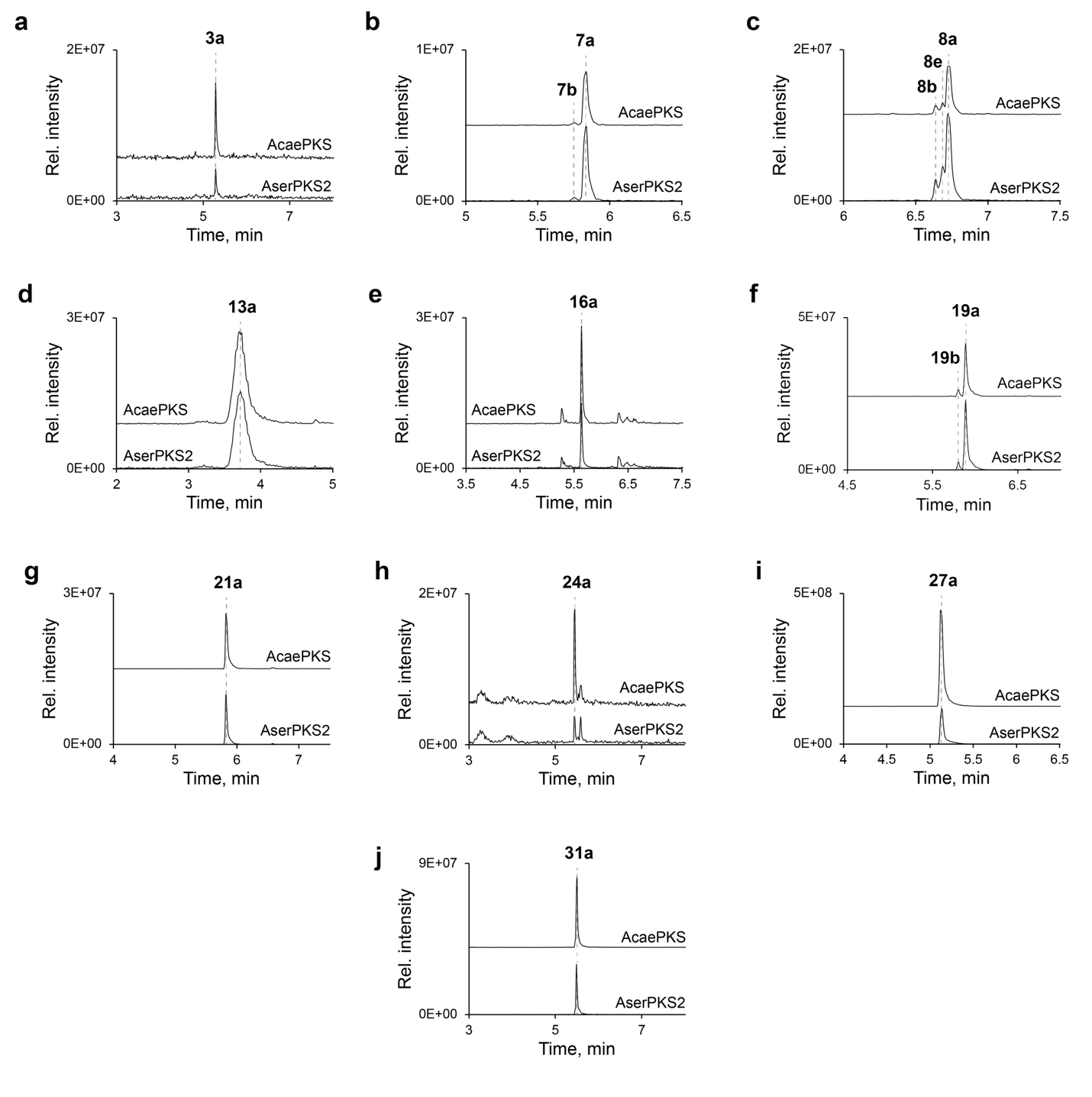


**Figure S10.** Extracted ion chromatograms in low-resolution LC-MS of reaction products of the cell-free expressed AcaePKS and AserPKS2. a) substrate **3**; b) substrate **7**; c) substrate **8**; d) substrate **13**; e) substrate **16**; f) substrate **19**; g) substrate **21**; h) substrate **24**; i) substrate **27**; and j) substrate **31**.


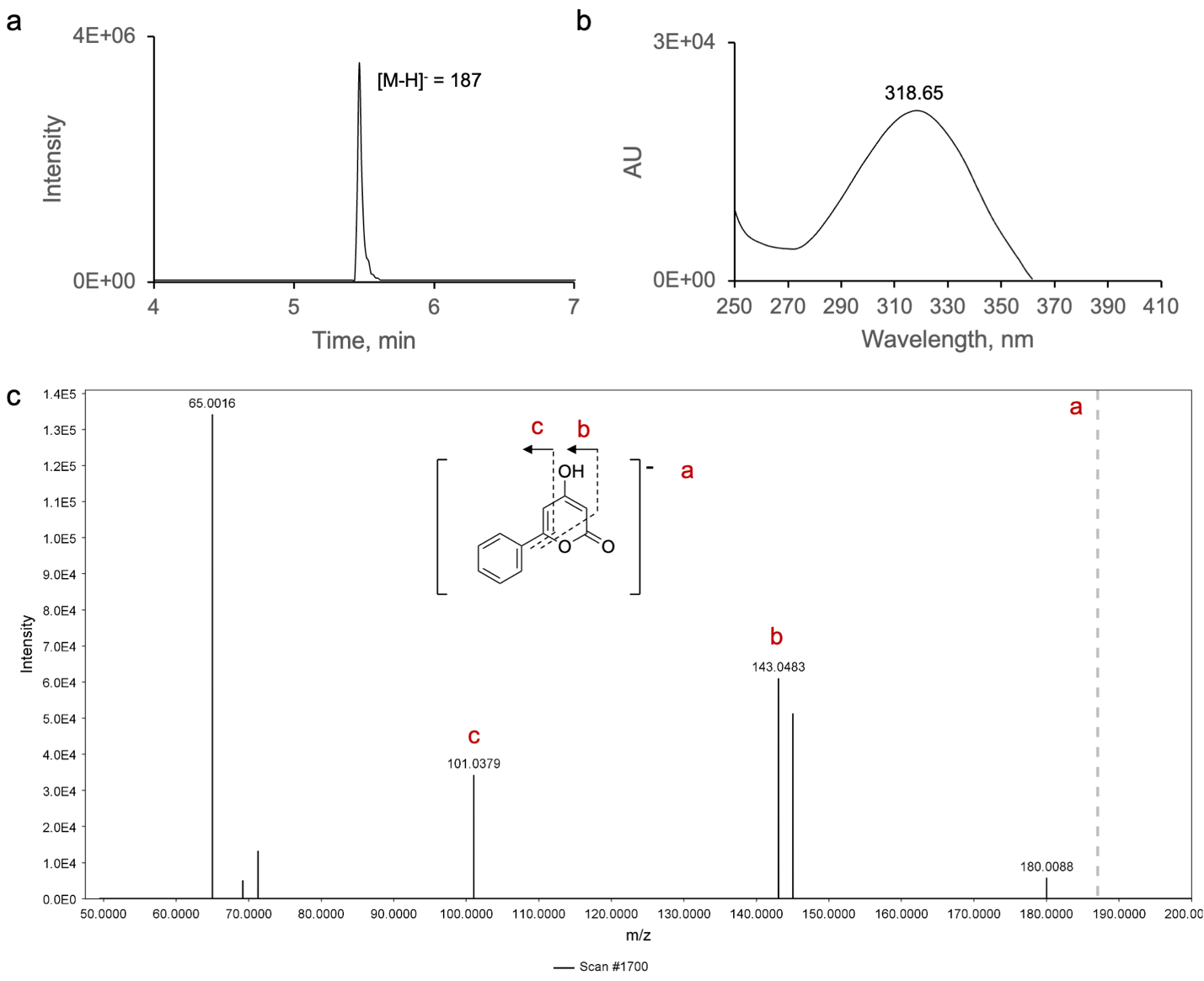


**Figure S11.** Spectral data analysis of product **1a**. a) Low-resolution LC-MS analysis of the EtOAc extract of the enzymatic reaction of AiizPKS with **1**; extracted ion chromatogram of the predicted *m/z* of 187 (negative mode). Y-axis shows relative ion intensity. b) Corresponding UV absorption spectrum. c) ESI-HR-MS/MS (negative mode) with ions matching expected fragments of **1a**; observed *m/z* = 187.0391 (theoretical *m/z* = 187.0401, calculated for [C_11_H_7_O_3_]^-^). The precursor ion is indicated with a dashed grey line.


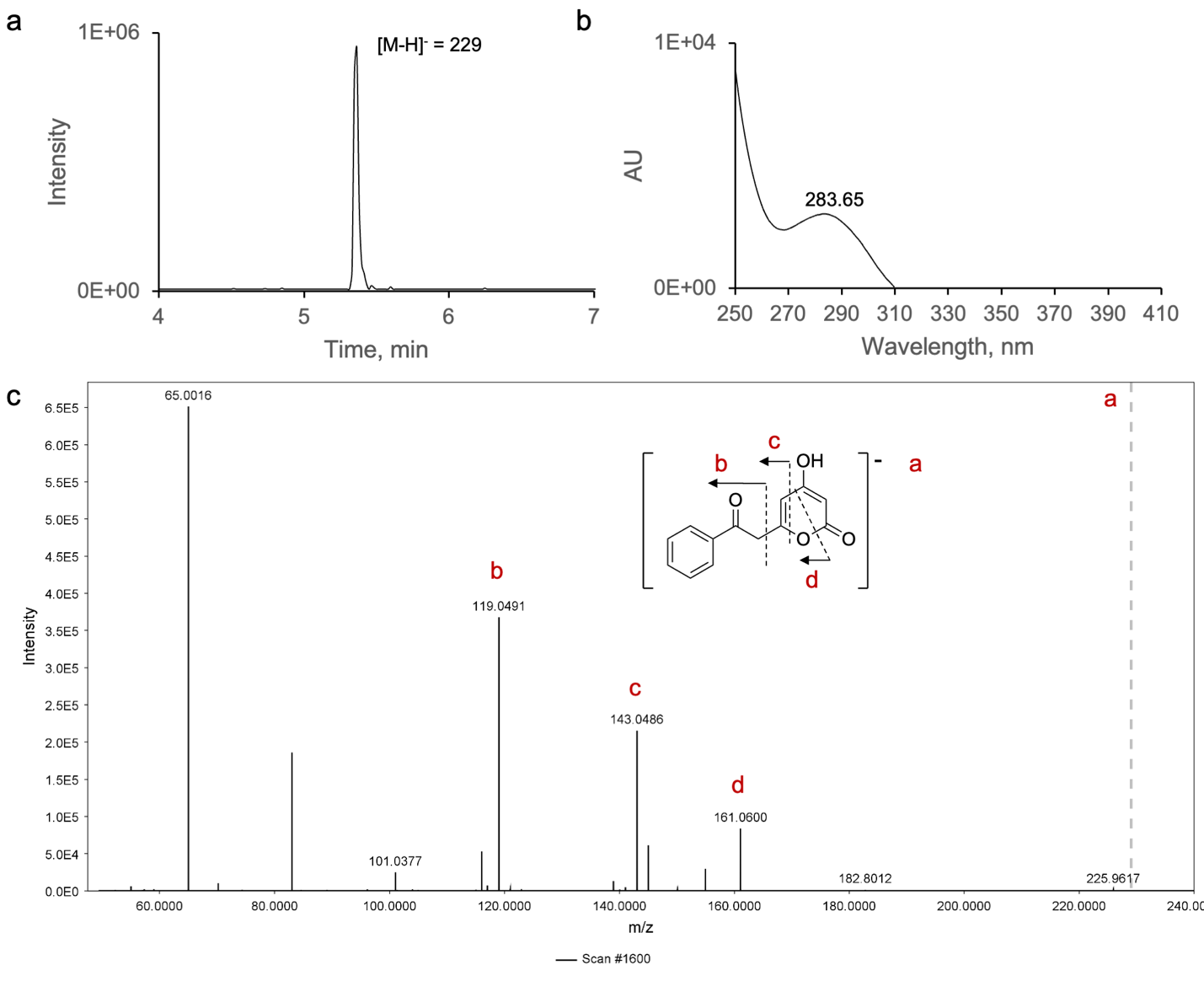


**Figure S12.** Spectral data analysis of product **1b**. a) Low-resolution LC-MS analysis of the EtOAc extract of the enzymatic reaction of AiizPKS with **1**; extracted ion chromatogram of the predicted *m/z* of 229 (negative mode). Y-axis shows relative ion intensity. b) Corresponding UV absorption spectrum. c) ESI-HR-MS/MS (negative mode) with ions matching expected fragments of **1b**; observed *m/z* = 229.0503 (theoretical *m/z* = 229.0506, calculated for [C_13_H_9_O_4_]^-^). The precursor ion is indicated with a dashed grey line.


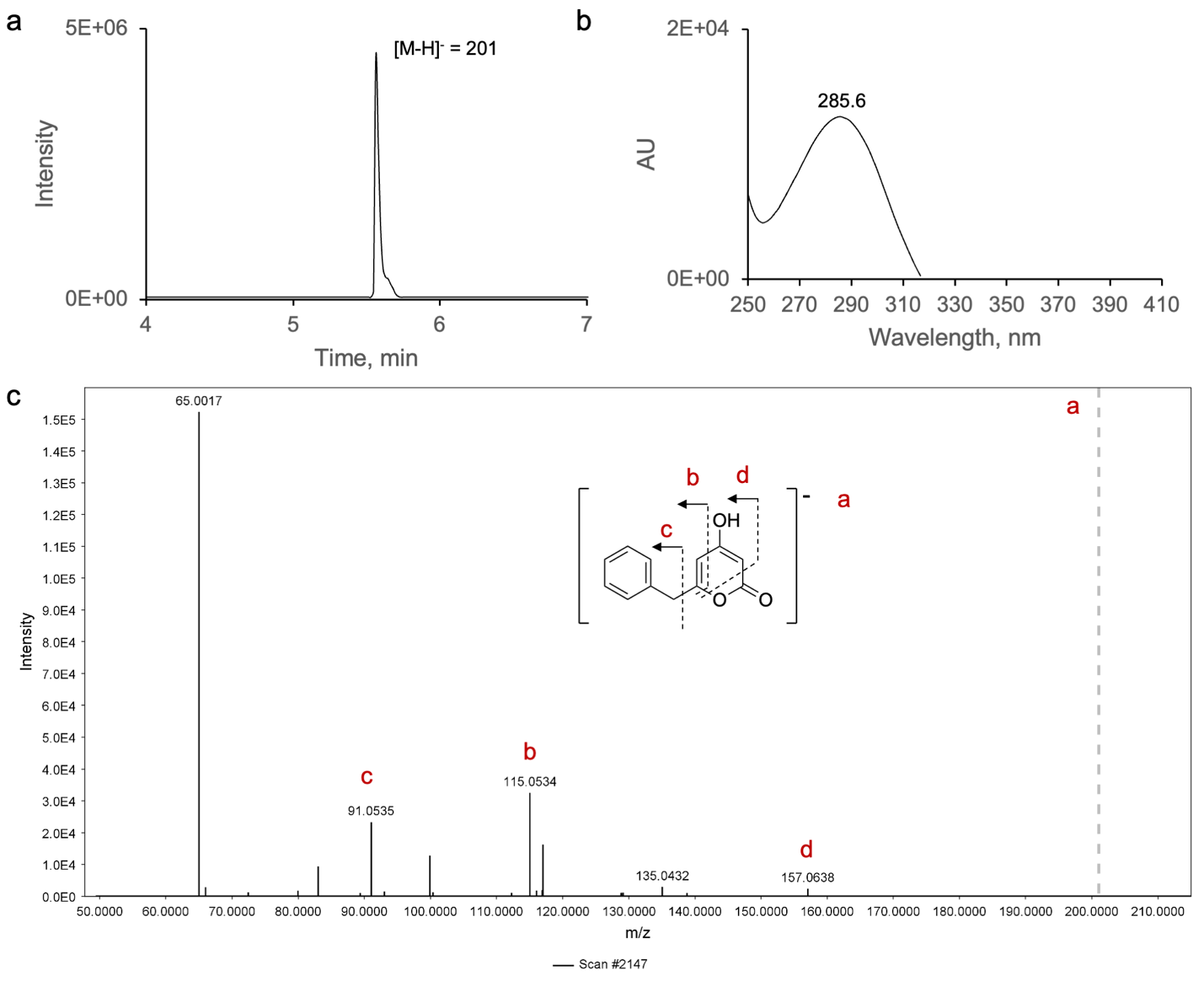


**Figure S13.** Spectral data analysis of product **2a**. a) Low-resolution LC-MS analysis of the EtOAc extract of the enzymatic reaction of AlupPKS1 with **2**; extracted ion chromatogram of the predicted *m/z* of 201 (negative mode). Y-axis shows relative ion intensity. b) Corresponding UV absorption spectrum. c) ESI-HR-MS/MS (negative mode) with ions matching expected fragments of **2a**; observed *m/z* = 201.0547 (theoretical *m/z* = 201.0557, calculated for [C_12_H_9_O_3_]^-^). The precursor ion is indicated with a dashed grey line.


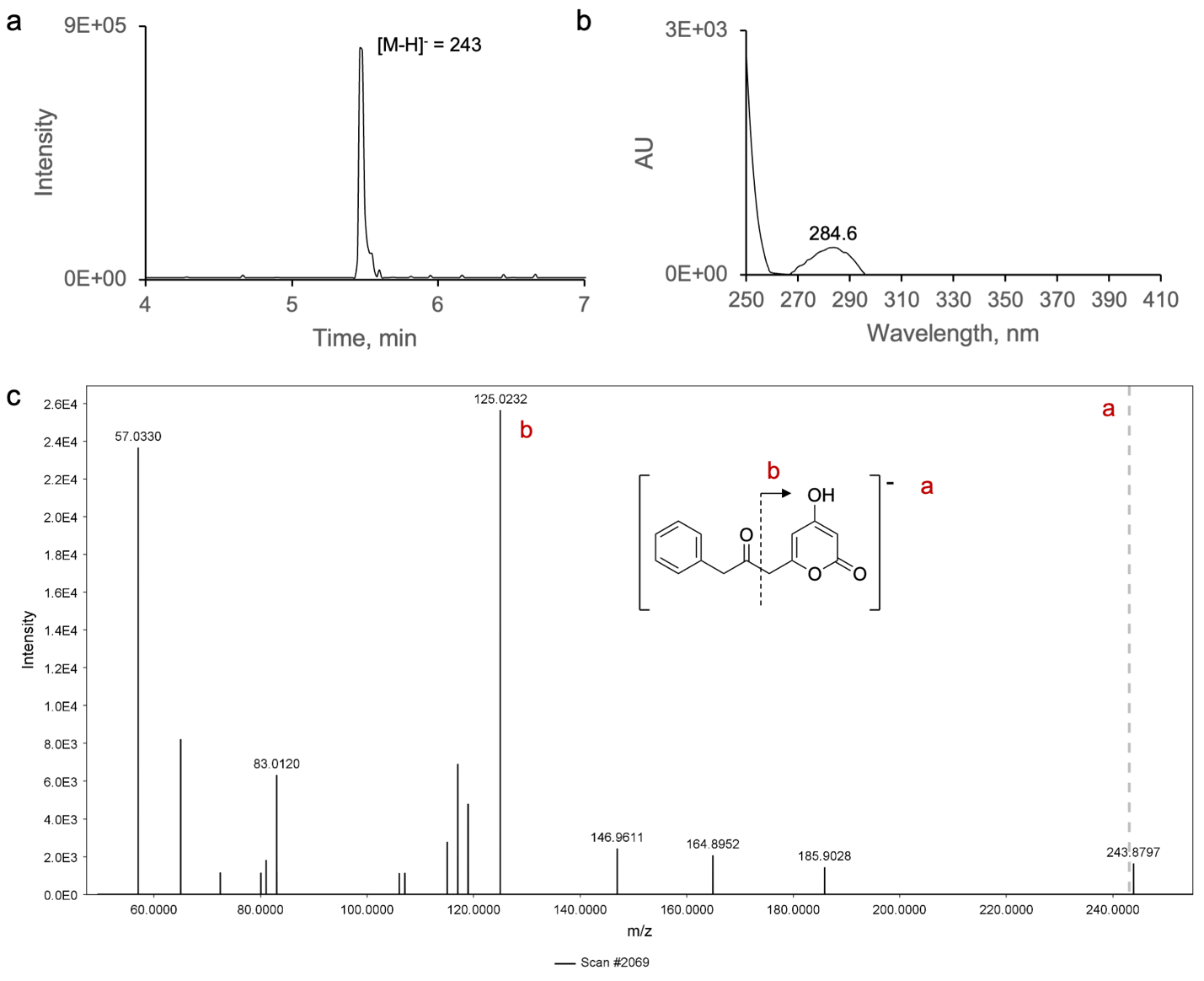


**Figure S14.** Spectral data analysis of product **2b**. a) Low-resolution LC-MS analysis of the EtOAc extract of the enzymatic reaction of AlupPKS1 with **2**; extracted ion chromatogram of the predicted *m/z* of 243 (negative mode). Y-axis shows relative ion intensity. b) Corresponding UV absorption spectrum. c) ESI-HR-MS/MS (negative mode) with ions matching expected fragments of **2b**; observed *m/z* = 243.0657 (theoretical *m/z* = 243.0663, calculated for [C_14_H_11_O_4_]^-^). The precursor ion is indicated with a dashed grey line.


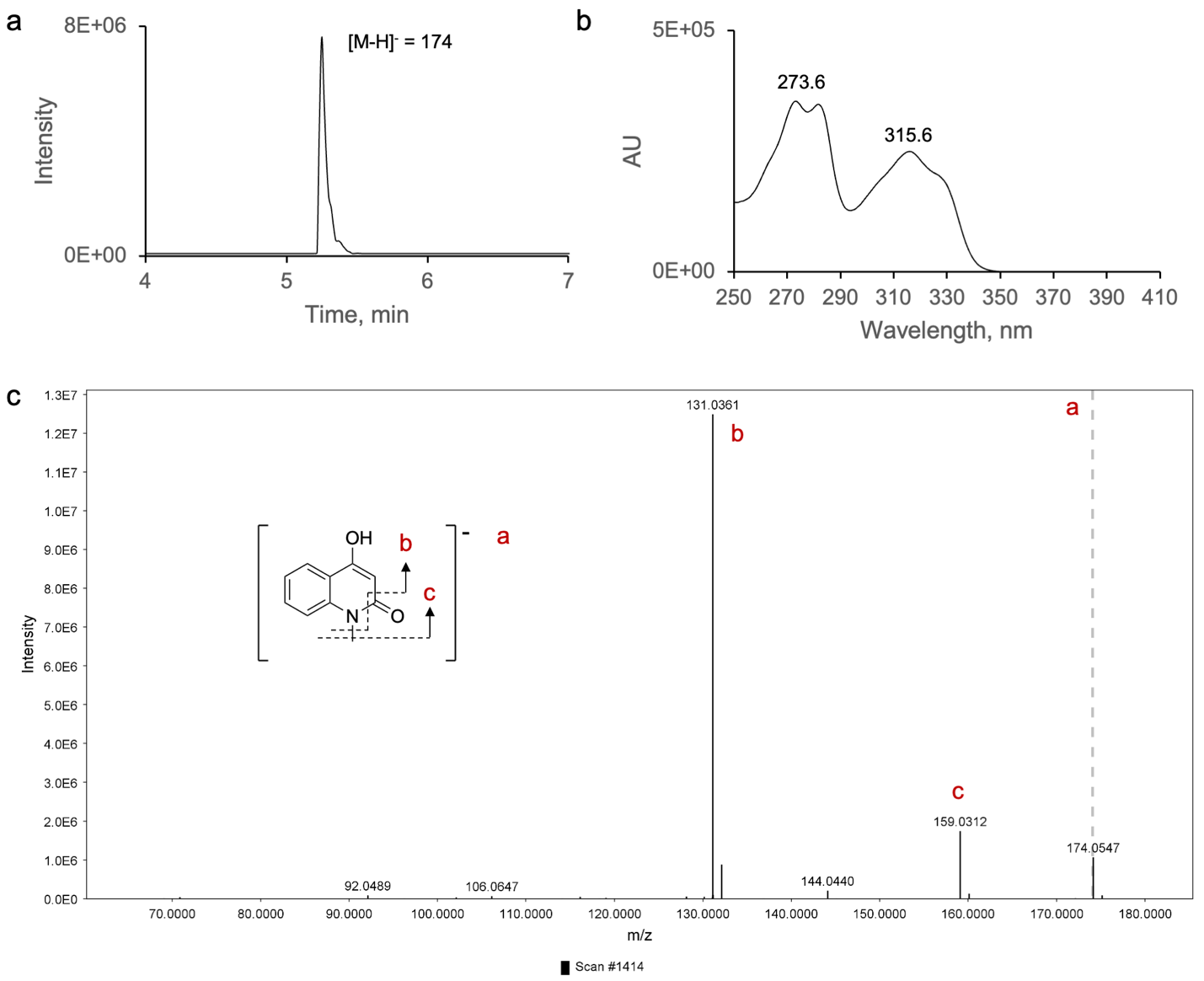


**Figure S15.** Spectral data analysis of product **3a**. a) Low-resolution LC-MS analysis of the EtOAc extract of the enzymatic reaction of AthePKS with **3**; extracted ion chromatogram of the predicted *m/z* of 174 (negative mode). Y-axis shows relative ion intensity. b) Corresponding UV absorption spectrum. c) ESI-HR-MS/MS (negative mode) with ions matching expected fragments of **3a**; observed *m/z* = 174.0547 (theoretical *m/z* = 174.0561, calculated for [C_10_H_8_NO_2_]^-^). The precursor ion is indicated with a dashed grey line.


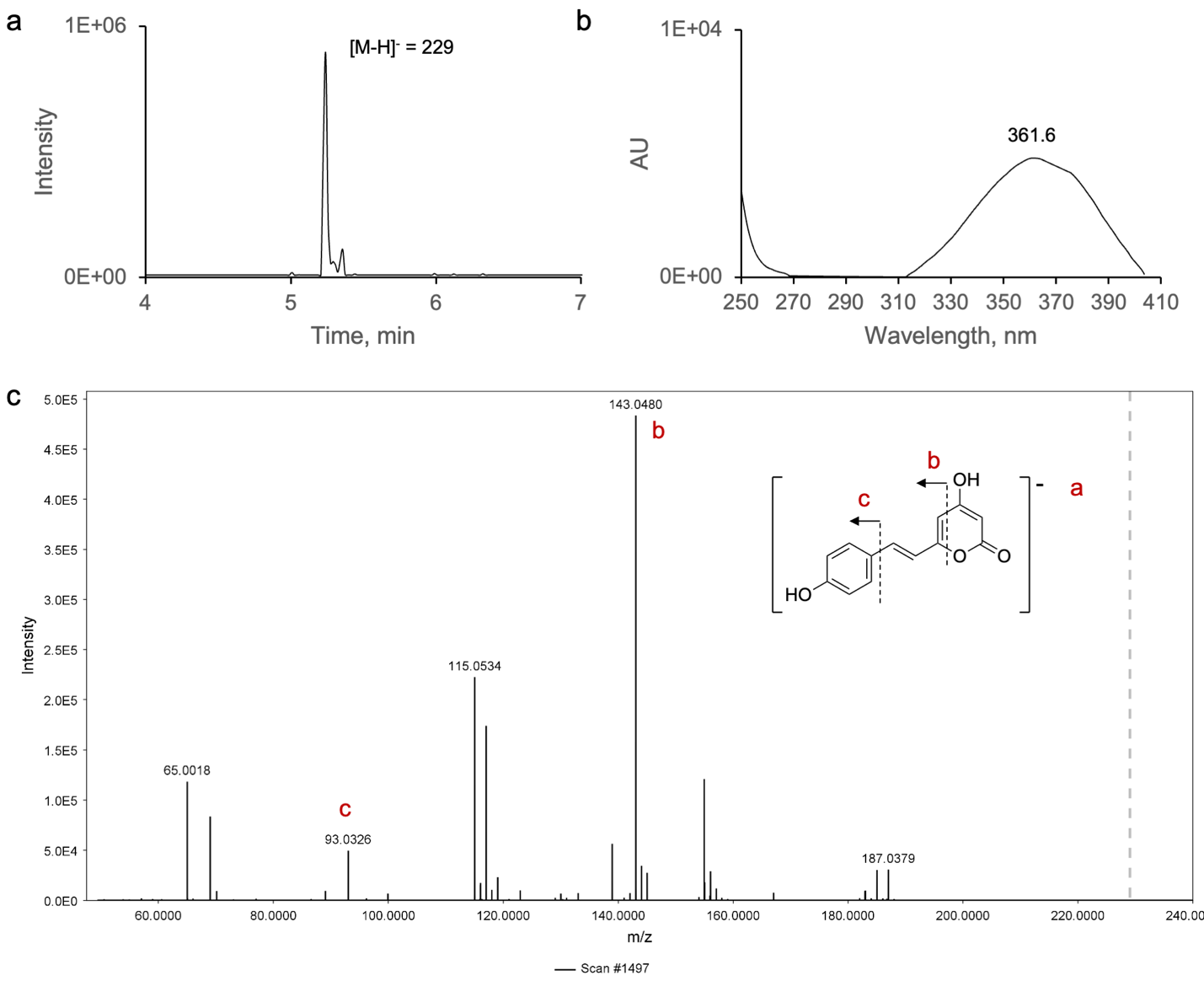


**Figure S16.** Spectral data analysis of product **4a**. a) Low-resolution LC-MS analysis of the EtOAc extract of the enzymatic reaction of 4 with HargPKS1; extracted ion chromatogram of the predicted *m/z* of 229 (negative mode). Y-axis shows relative ion intensity. b) Corresponding UV absorption spectrum. c) ESI-HR-MS/MS (negative mode) with ions matching expected fragments of **4a**; observed *m/z* = 229.0500 (theoretical *m/z* = 229.0506, calculated for [C_13_H_9_O_4_]^-^). The precursor ion is indicated with a dashed grey line.


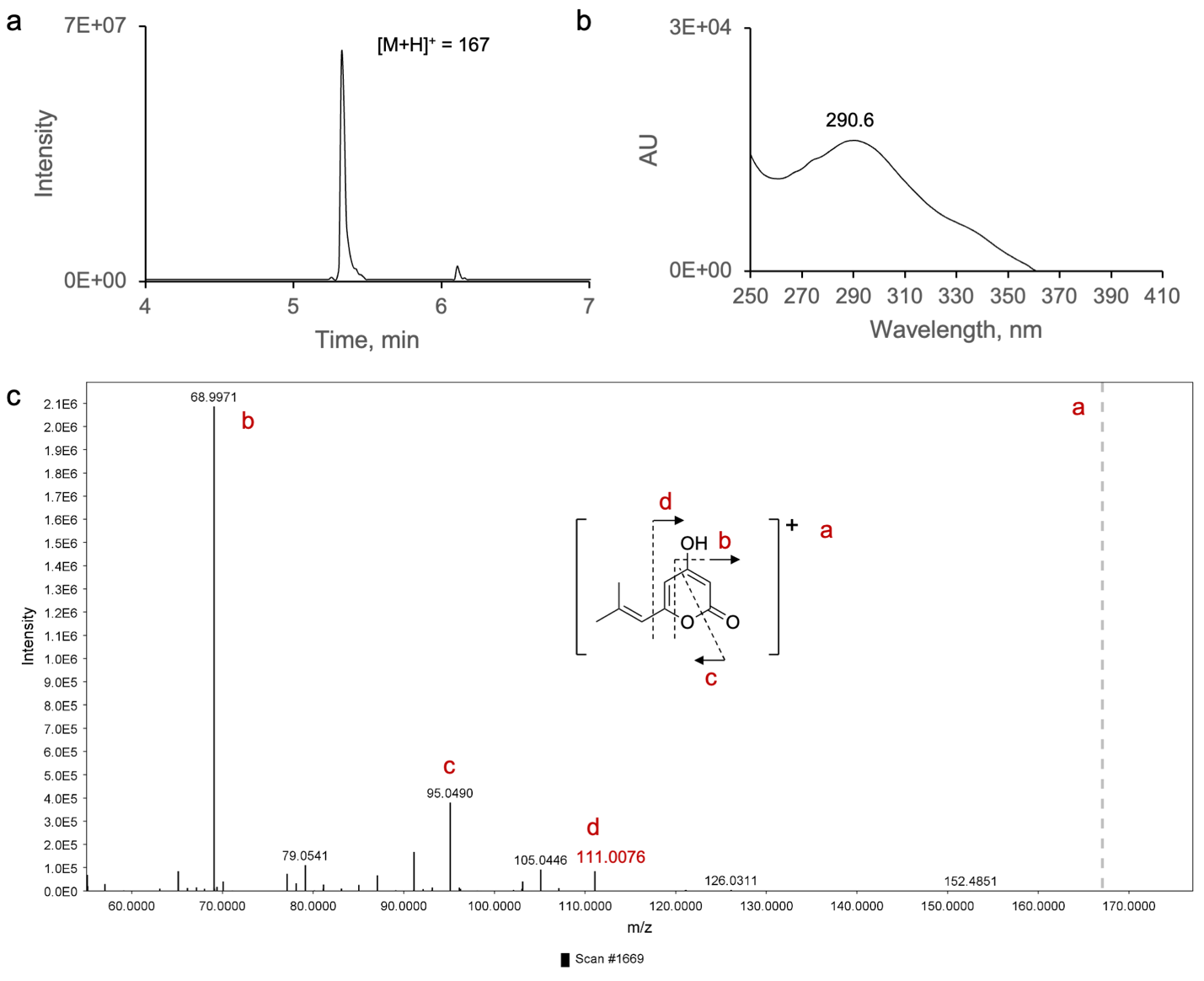


**Figure S17.** Spectral data analysis of product **5a**. a) Low-resolution LC-MS analysis of the EtOAc extract of the enzymatic reaction of CgloPKS with **5**; extracted ion chromatogram of the predicted *m/z* of 167 (positive mode). Y-axis shows relative ion intensity. b) Corresponding UV absorption spectrum. c) ESI-HR-MS/MS (positive mode) with ions matching expected fragments of **5a**; observed *m/z* = 167.0703 (theoretical *m/z* = 167.0703, calculated for [C_9_H_11_O_3_]^+^). The precursor ion is indicated with a dashed grey line.


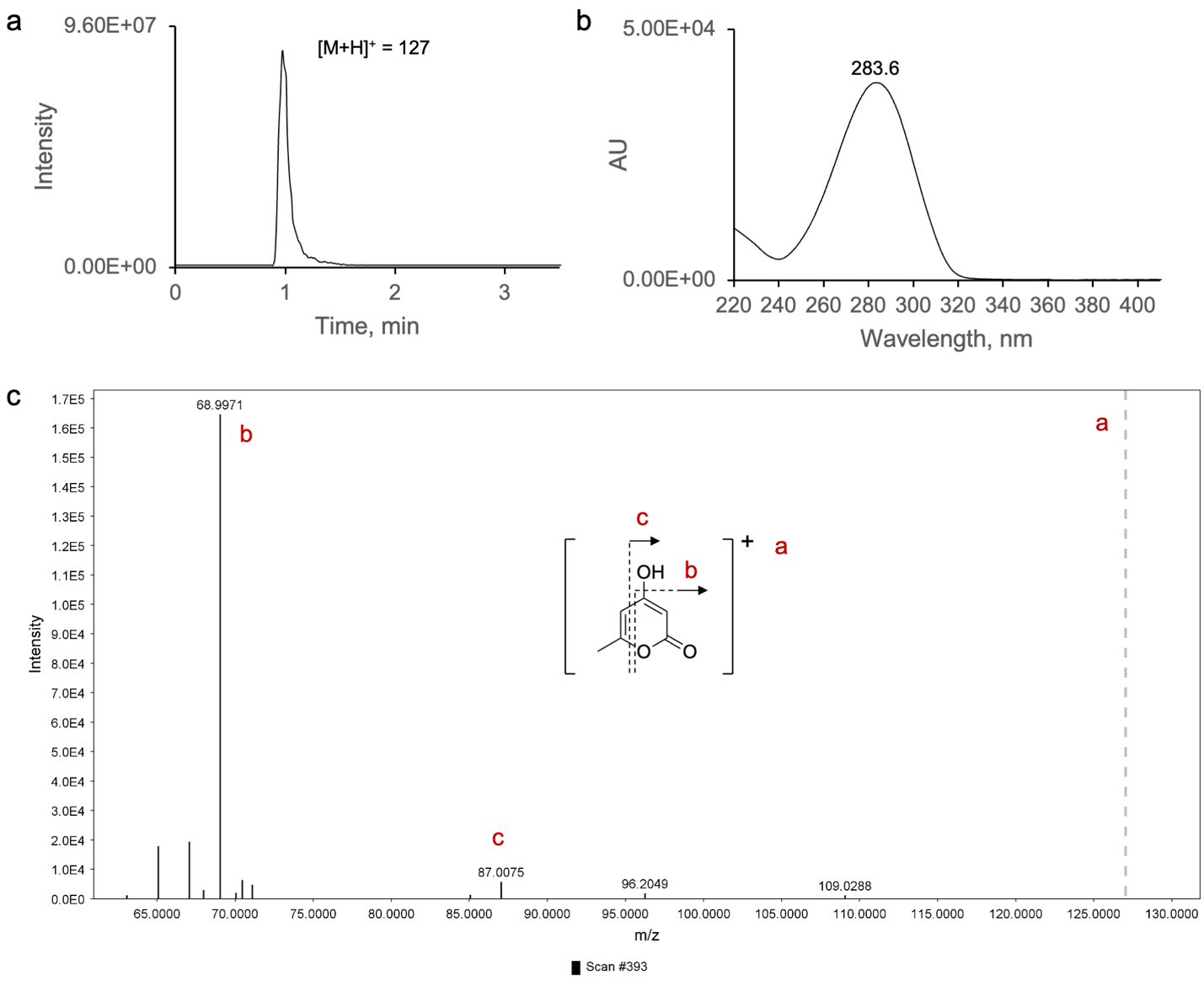


**Figure S18.** Spectral data analysis of product **6a**. a) Low-resolution LC-MS analysis of the EtOAc extract of the enzymatic reaction of FerePKS with **6**; extracted ion chromatogram of the predicted *m/z* of 127 (positive mode). Y-axis shows relative ion intensity. b) Corresponding UV absorption spectrum. c) ESI-HR-MS/MS (positive mode) with ions matching expected fragments of **6a**; observed *m/z* = 127.0388 (theoretical *m/z* = 127.0390, calculated for [C_6_H_7_O_3_]^+^). The precursor ion is indicated with a dashed grey line.


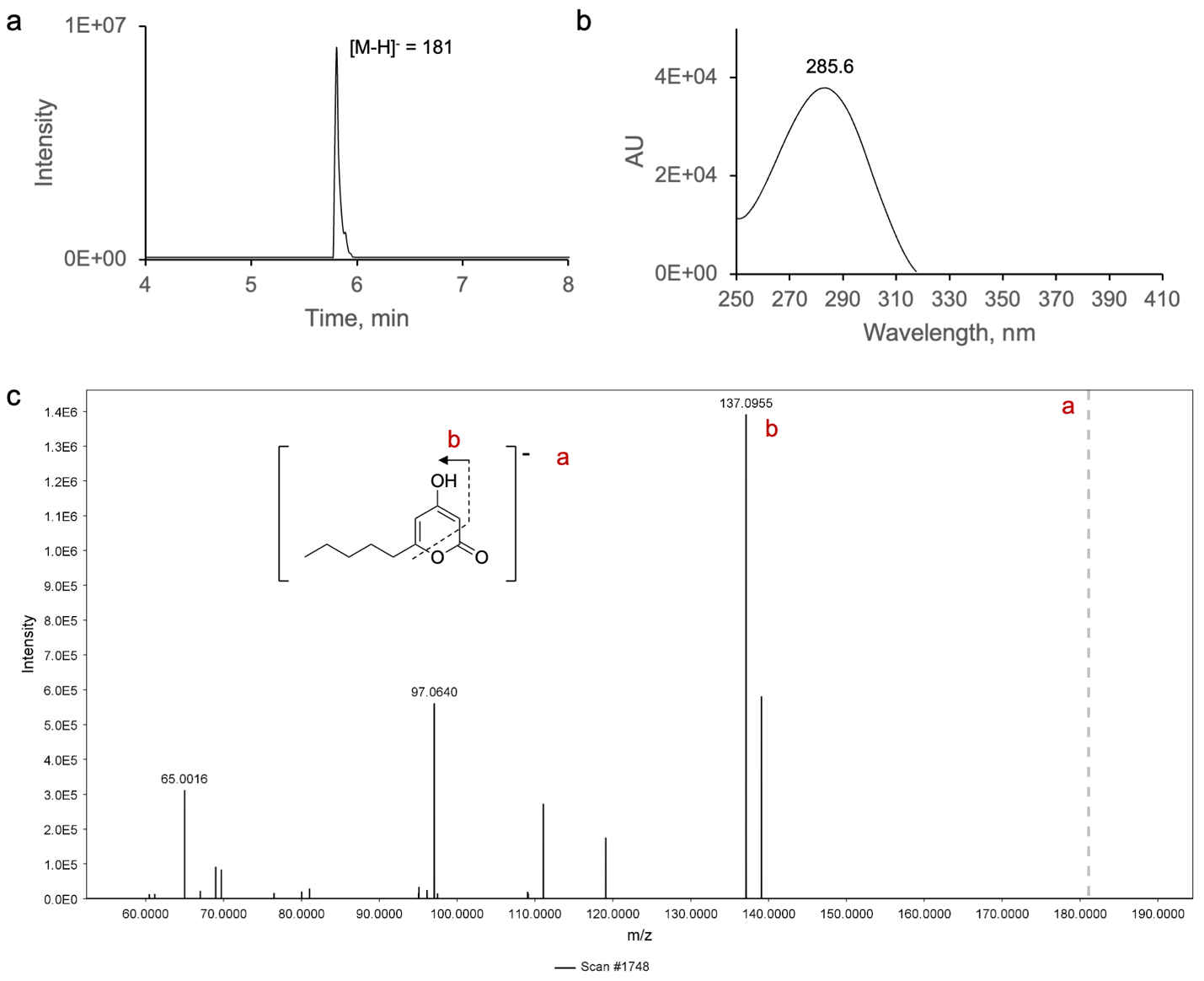


**Figure S19.** Spectral data analysis of product **7a**. a) Low-resolution LC-MS analysis of the EtOAc extract of the enzymatic reaction of AserPKS2 with **7**; extracted ion chromatogram of the predicted *m/z* of 181 (negative mode). Y-axis shows relative ion intensity. b) Corresponding UV absorption spectrum. c) ESI-HR-MS/MS (negative mode) with ions matching expected fragments of **7a**; observed *m/z* = 181.0861 (theoretical *m/z* = 181.0870, calculated for [C_10_H_13_O_3_]^-^). The precursor ion is indicated with a dashed grey line.


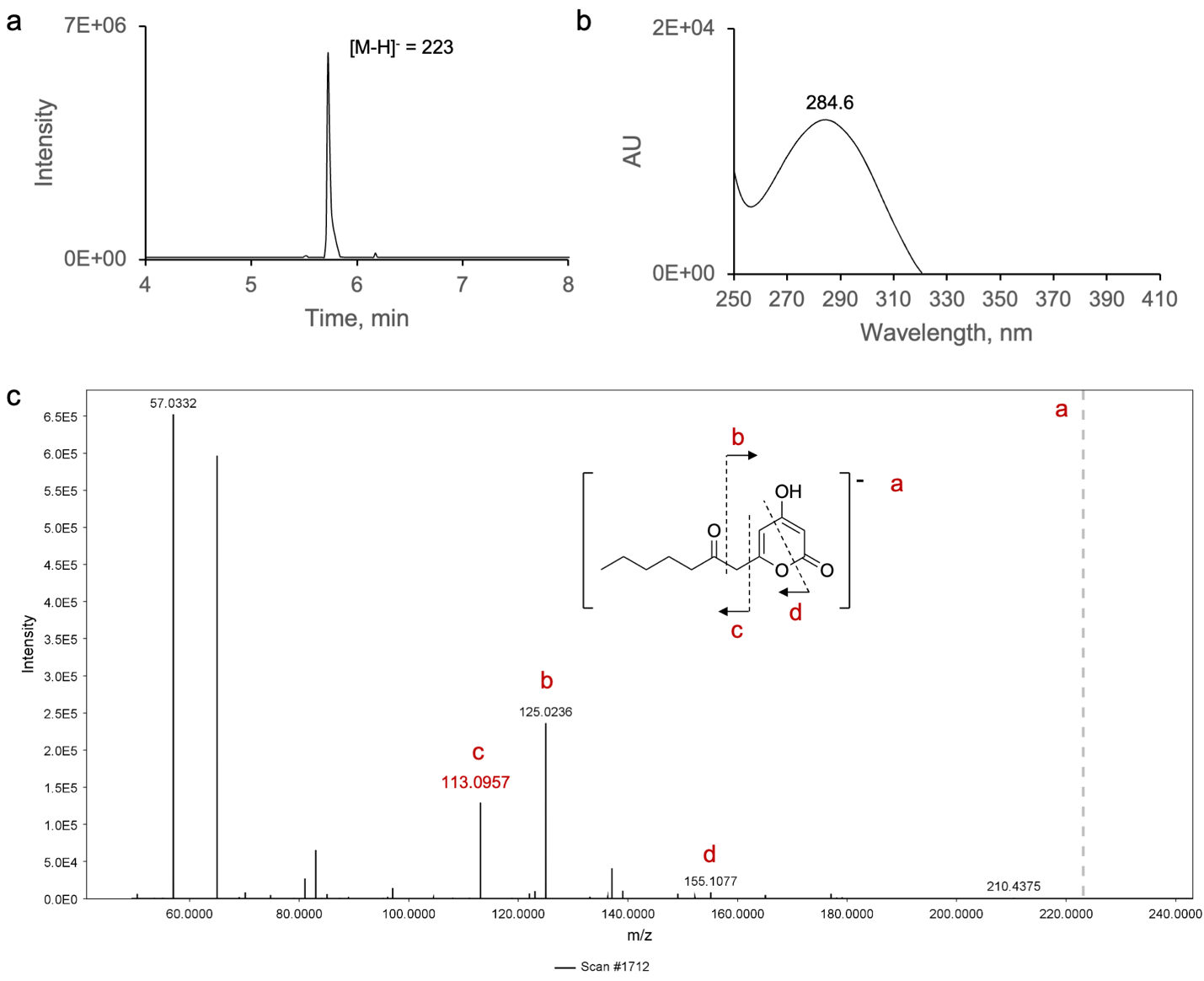


**Figure S20.** Spectral data analysis of product **7b**. a) Low-resolution LC-MS analysis of the EtOAc extract of the enzymatic reaction of AserPKS2 with **7**; extracted ion chromatogram of the predicted *m/z* of 223 (negative mode). Y-axis shows relative ion intensity. b) Corresponding UV absorption spectrum. c) ESI-HR-MS/MS (negative mode) with ions matching expected fragments of **7b**; observed *m/z* = 223.0973 (theoretical *m/z* = 223.0976, calculated for [C_12_H_15_O_4_]^-^). The precursor ion is indicated with a dashed grey line.


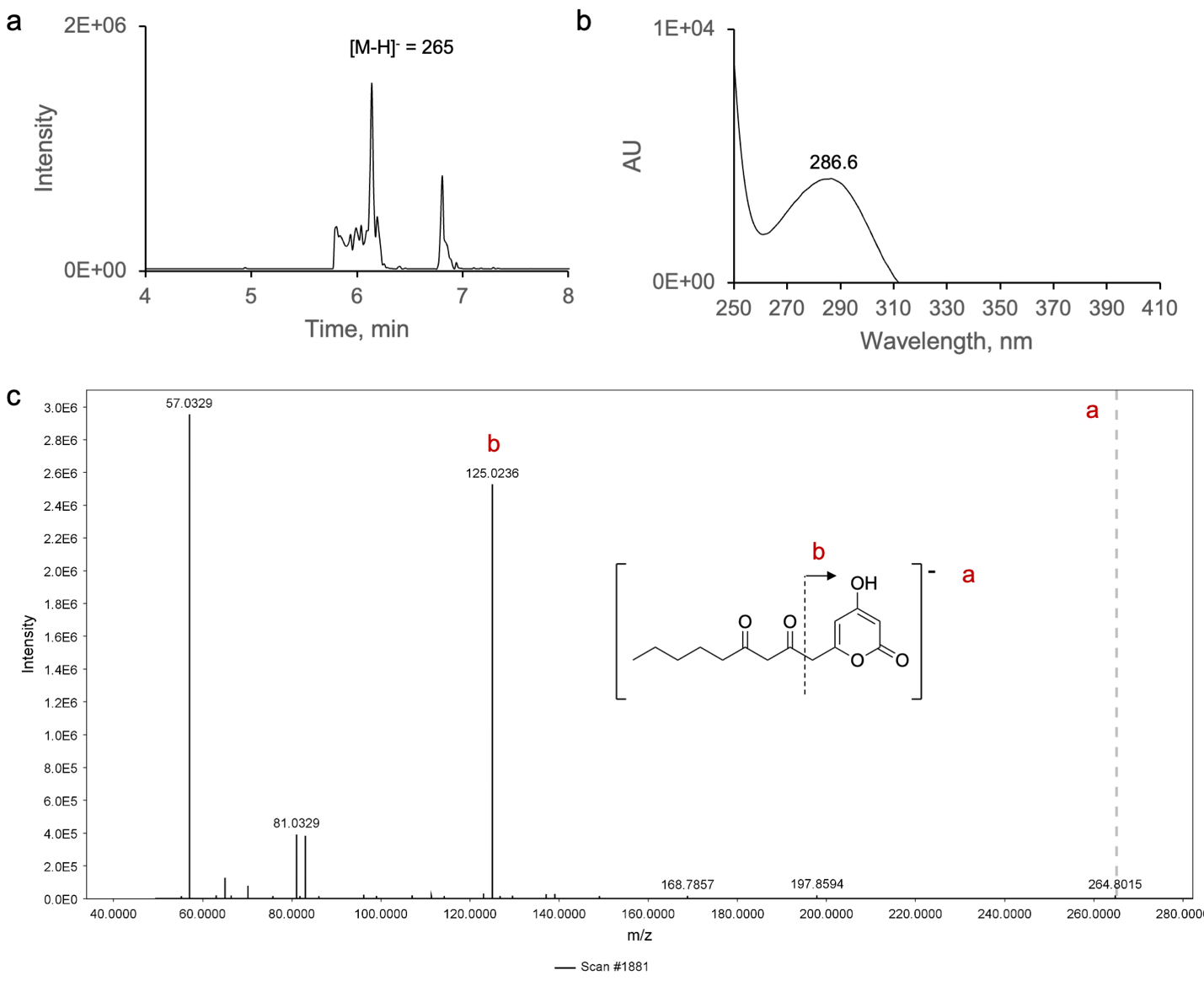


**Figure S21.** Spectral data analysis of product **7c**. a) Low-resolution LC-MS analysis of the EtOAc extract of the enzymatic reaction of HargPKS1 with **7**; extracted ion chromatogram of the predicted *m/z* of 265 (negative mode). Y-axis shows relative ion intensity. b) Corresponding UV absorption spectrum. c) ESI-HR-MS/MS (negative mode) with ions matching expected fragments of **7c**; observed *m/z* = 265.1083 (theoretical *m/z* = 265.1081, calculated for [C_14_H_17_O_5_]^-^). The precursor ion is indicated with a dashed grey line.


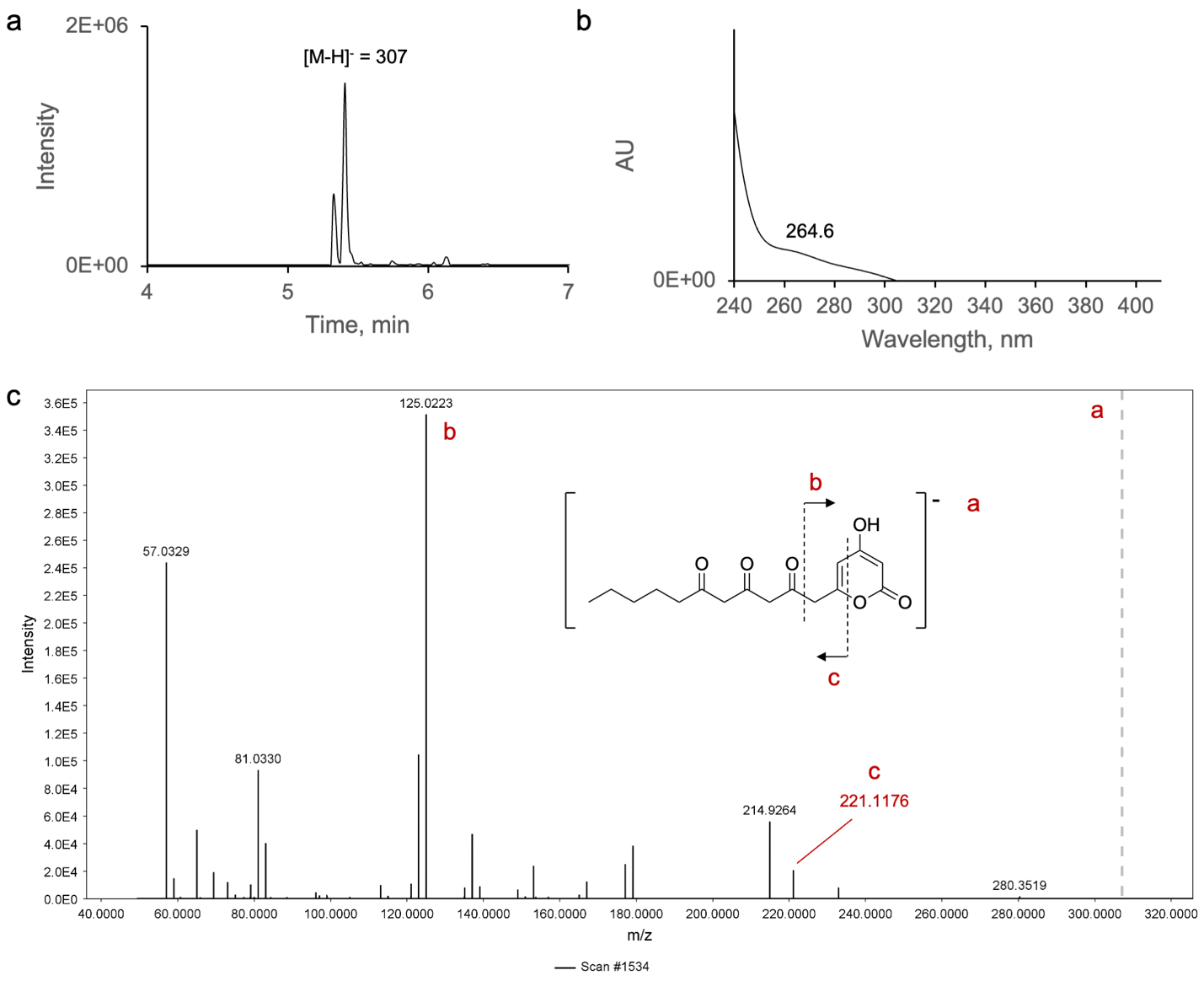


**Figure S22.** Spectral data analysis of product **7d**. a) Low-resolution LC-MS analysis of the EtOAc extract of the enzymatic reaction of HargPKS1 with **7**; extracted ion chromatogram of the predicted *m/z* of 307 (negative mode). Y-axis shows relative ion intensity. b) Corresponding UV absorption spectrum. c) ESI-HR-MS/MS (negative mode) with ions matching expected fragments of **7d**; observed *m/z* = 307.1190 (theoretical *m/z* = 307.1187, calculated for [C_16_H_19_O_6_]^-^). The precursor ion is indicated with a dashed grey line.


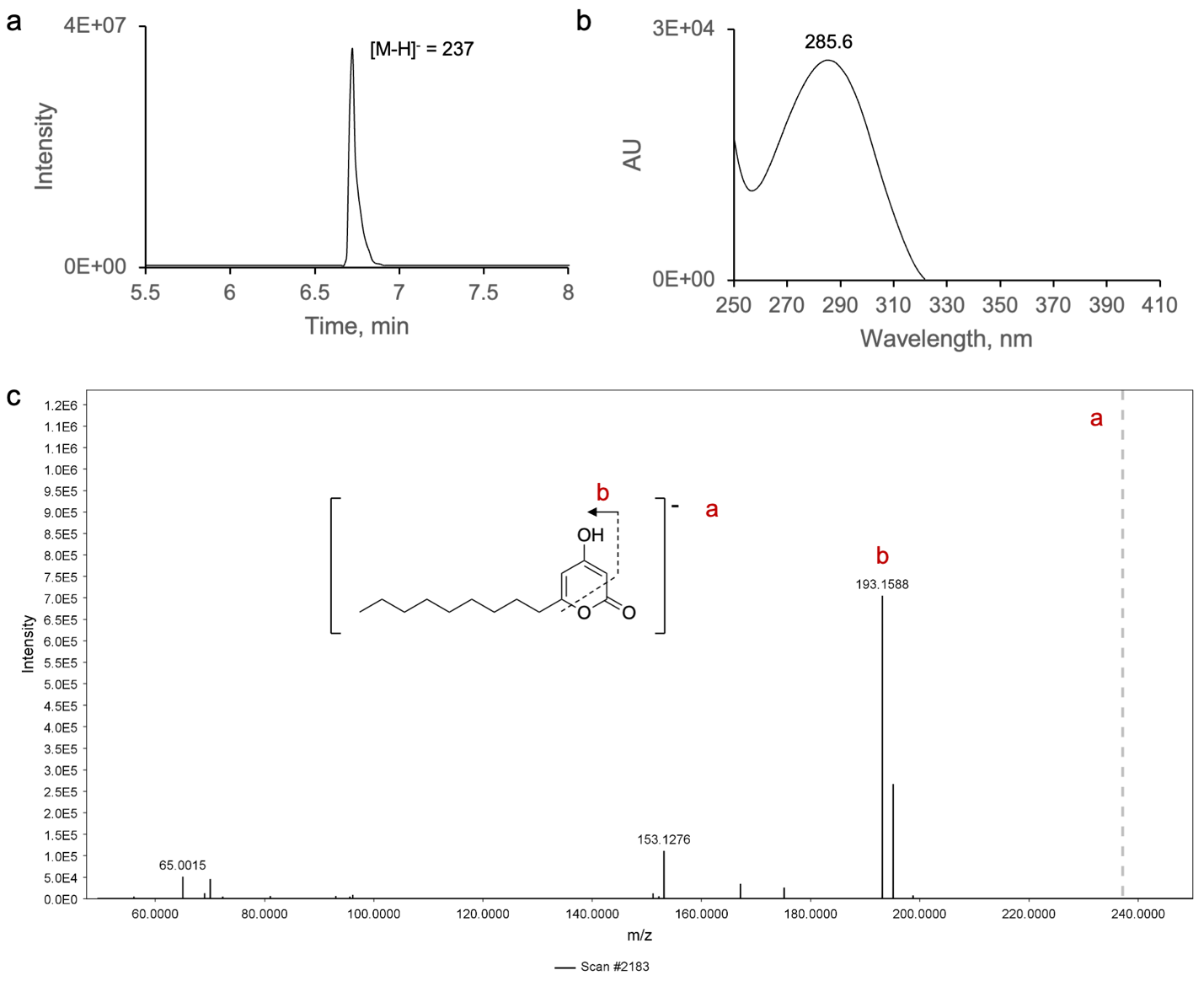


**Figure S23.** Spectral data analysis of product **8a**. a) Low-resolution LC-MS analysis of the EtOAc extract of the enzymatic reaction of HargPKS1 with **8**; extracted ion chromatogram of the predicted *m/z* of 237 (negative mode). Y-axis shows relative ion intensity. b) Corresponding UV absorption spectrum. c) ESI-HR-MS/MS (negative mode) with ions matching expected fragments of **8a**; observed *m/z* = 237.1495 (theoretical *m/z* = 237.1496, calculated for [C_14_H_21_O_3_]^-^). The precursor ion is indicated with a dashed grey line.


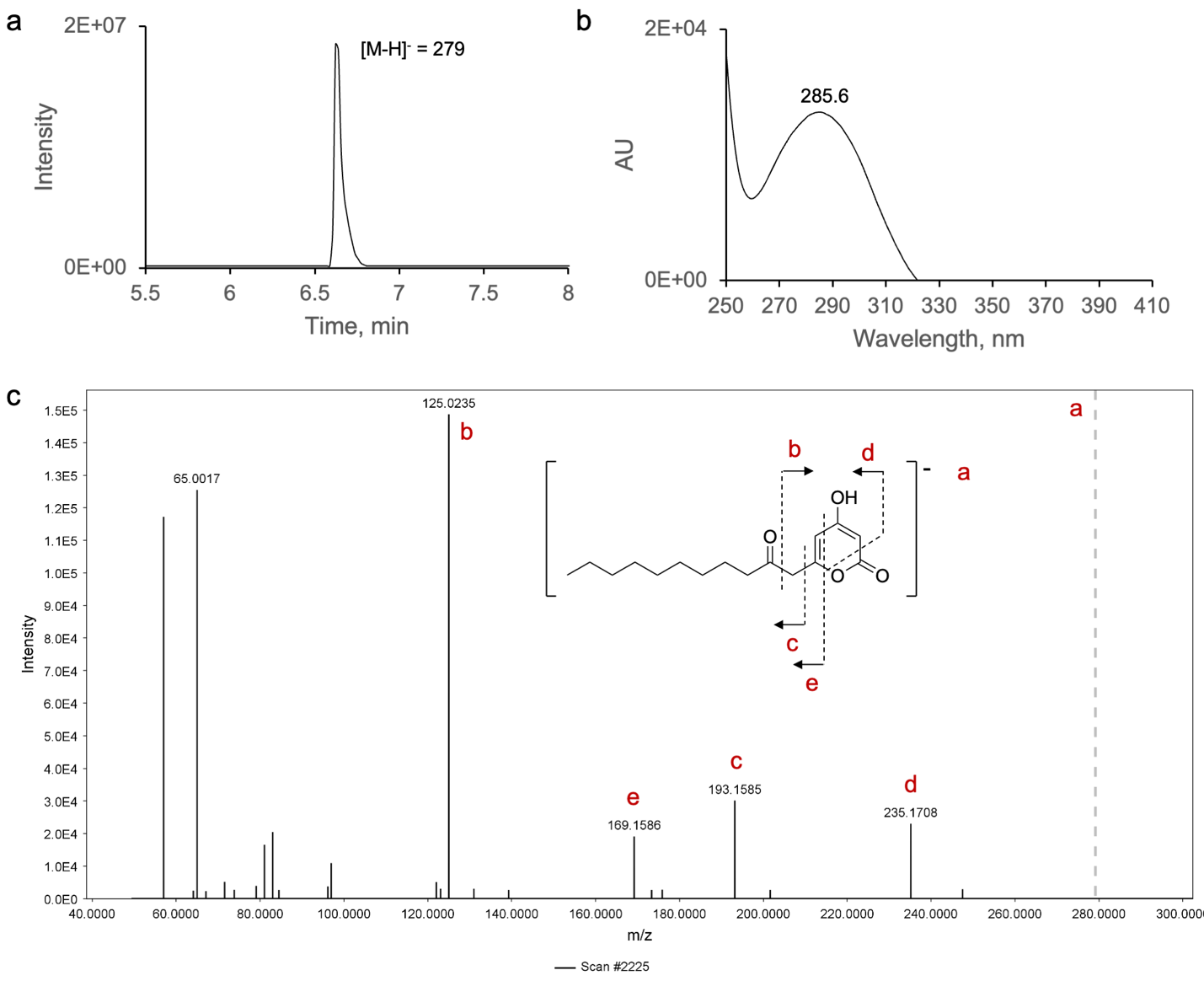


**Figure S24.** Spectral data analysis of product **8b**. a) Low-resolution LC-MS analysis of the EtOAc extract of the enzymatic reaction of DhelPKS with **8**; extracted ion chromatogram of the predicted *m/z* of 279 (negative mode). Y-axis shows relative ion intensity. b) Corresponding UV absorption spectrum. c) ESI-HR-MS/MS (negative mode) with ions matching expected fragments of **8b**; observed *m/z* = 279.1603 (theoretical *m/z* = 279.1602, calculated for [C_16_H_23_O_4_]^-^). The precursor ion is indicated with a dashed grey line.


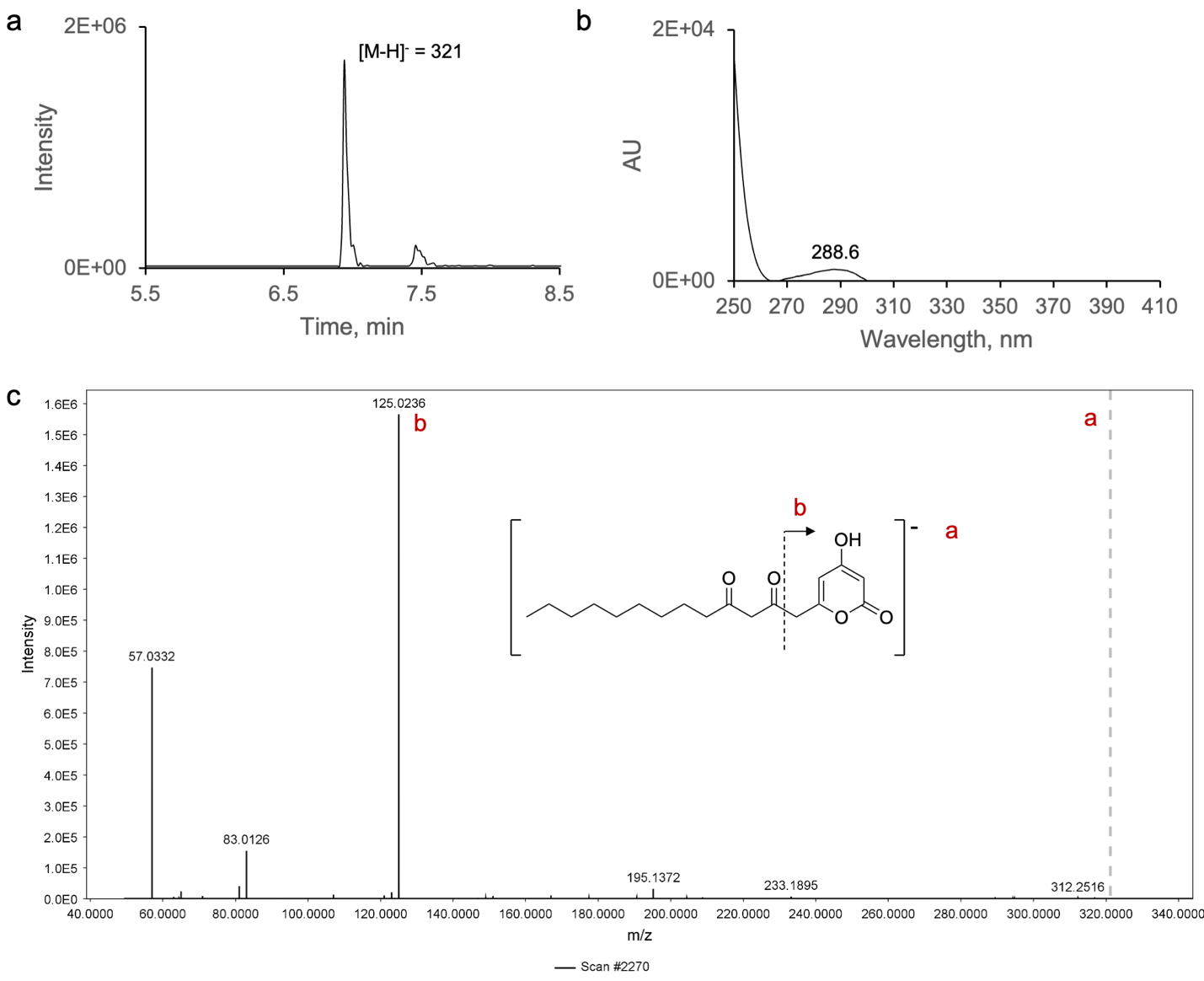


**Figure S25.** Spectral data analysis of product **8c**. a) Low-resolution LC-MS analysis of the EtOAc extract of the enzymatic reaction of AsesPKS with **8**; extracted ion chromatogram of the predicted *m/z* of 321 (negative mode). Y-axis shows relative ion intensity. b) Corresponding UV absorption spectrum. c) ESI-HR-MS/MS (negative mode) with ions matching expected fragments of **8c**; observed *m/z* = 321.1711 (theoretical *m/z* = 321.1707, calculated for [C_18_H_25_O_5_]^-^). The precursor ion is indicated with a dashed grey line.


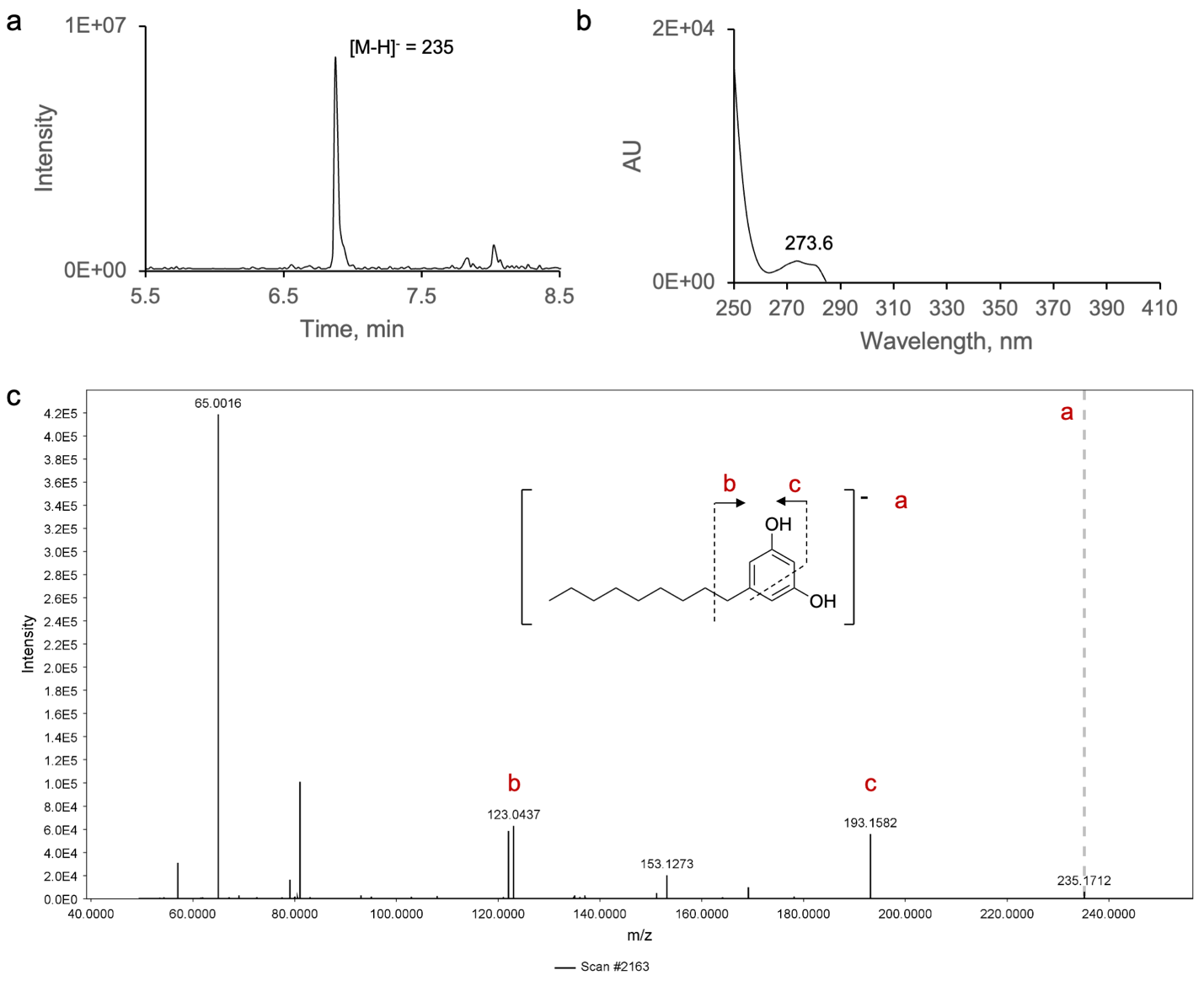


**Figure S26.** Spectral data analysis of product **8d**. a) Low-resolution LC-MS analysis of the EtOAc extract of the enzymatic reaction of DhelPKS with **8**; extracted ion chromatogram of the predicted *m/z* of 235 (negative mode). Y-axis shows relative ion intensity. b) Corresponding UV absorption spectrum. c) ESI-HR-MS/MS (negative mode) with ions matching expected fragments of **8d**; observed *m/z* = 235.1701 (theoretical *m/z* = 235.1704, calculated for [C_15_H_23_O_2_]^-^). The precursor ion is indicated with a dashed grey line.


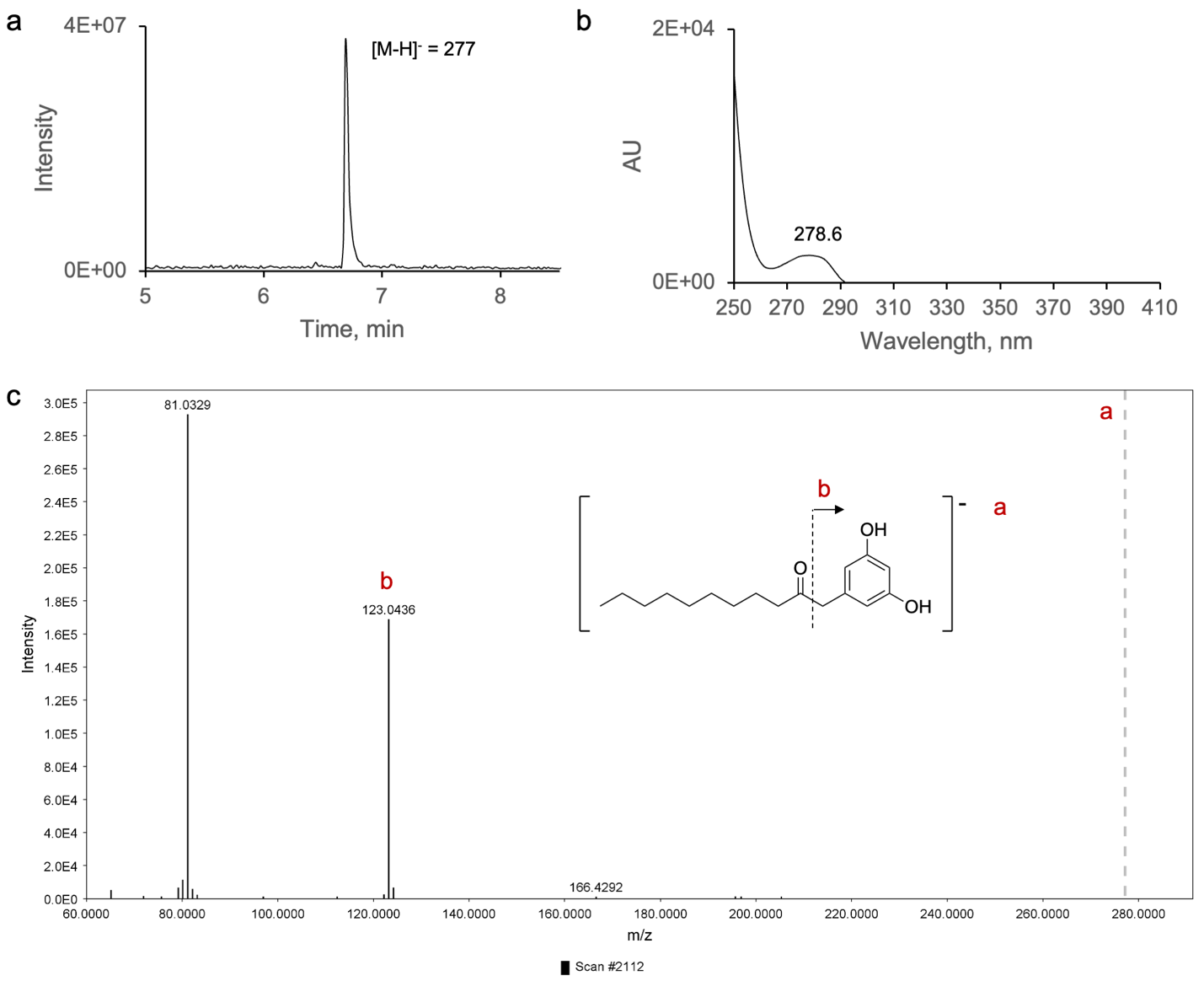


**Figure S27.** Spectral data analysis of product **8e**. a) Low-resolution LC-MS analysis of the EtOAc extract of the enzymatic reaction of AcosPKS with **8**; extracted ion chromatogram of the predicted *m/z* of 277 (negative mode). Y-axis shows relative ion intensity. b) Corresponding UV absorption spectrum. c) ESI-HR-MS/MS (negative mode) with ions matching expected fragments of **8e**; observed *m/z* = 277.1805 (theoretical *m/z* = 277.1809, calculated for [C_17_H_25_O_3_]^-^). The precursor ion is indicated with a dashed grey line.


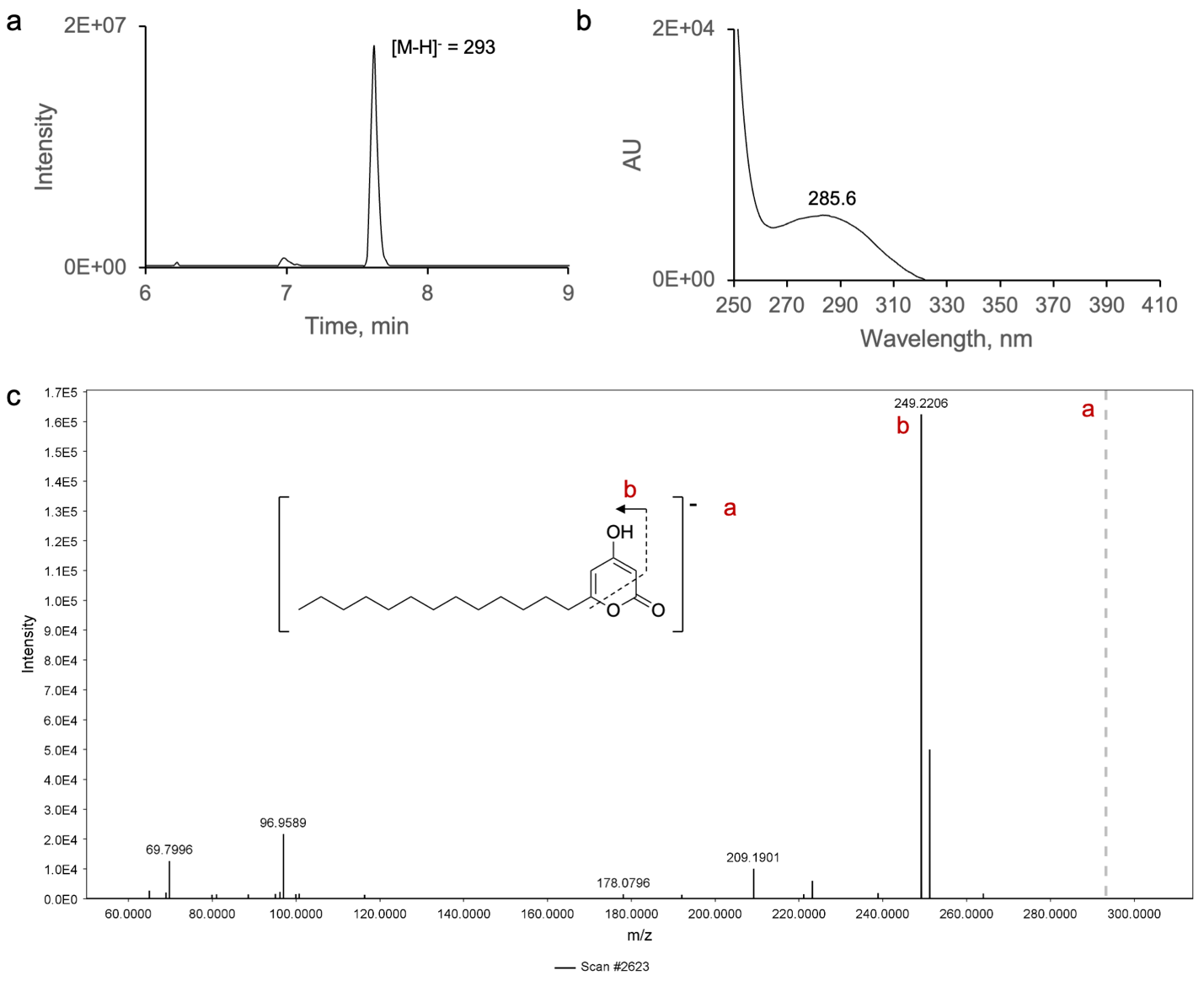


**Figure S28.** Spectral data analysis of product **9a**. a) Low-resolution LC-MS analysis of the EtOAc extract of the enzymatic reaction of DhelPKS with **9**; extracted ion chromatogram of the predicted *m/z* of 293 (negative mode). Y-axis shows relative ion intensity. b) Corresponding UV absorption spectrum. c) ESI-HR-MS/MS (negative mode) with ions matching expected fragments of **9a**; observed *m/z* = 293.2127 (theoretical *m/z* = 293.2122, calculated for [C_18_H_29_O_3_]^-^). The precursor ion is indicated with a dashed grey line.


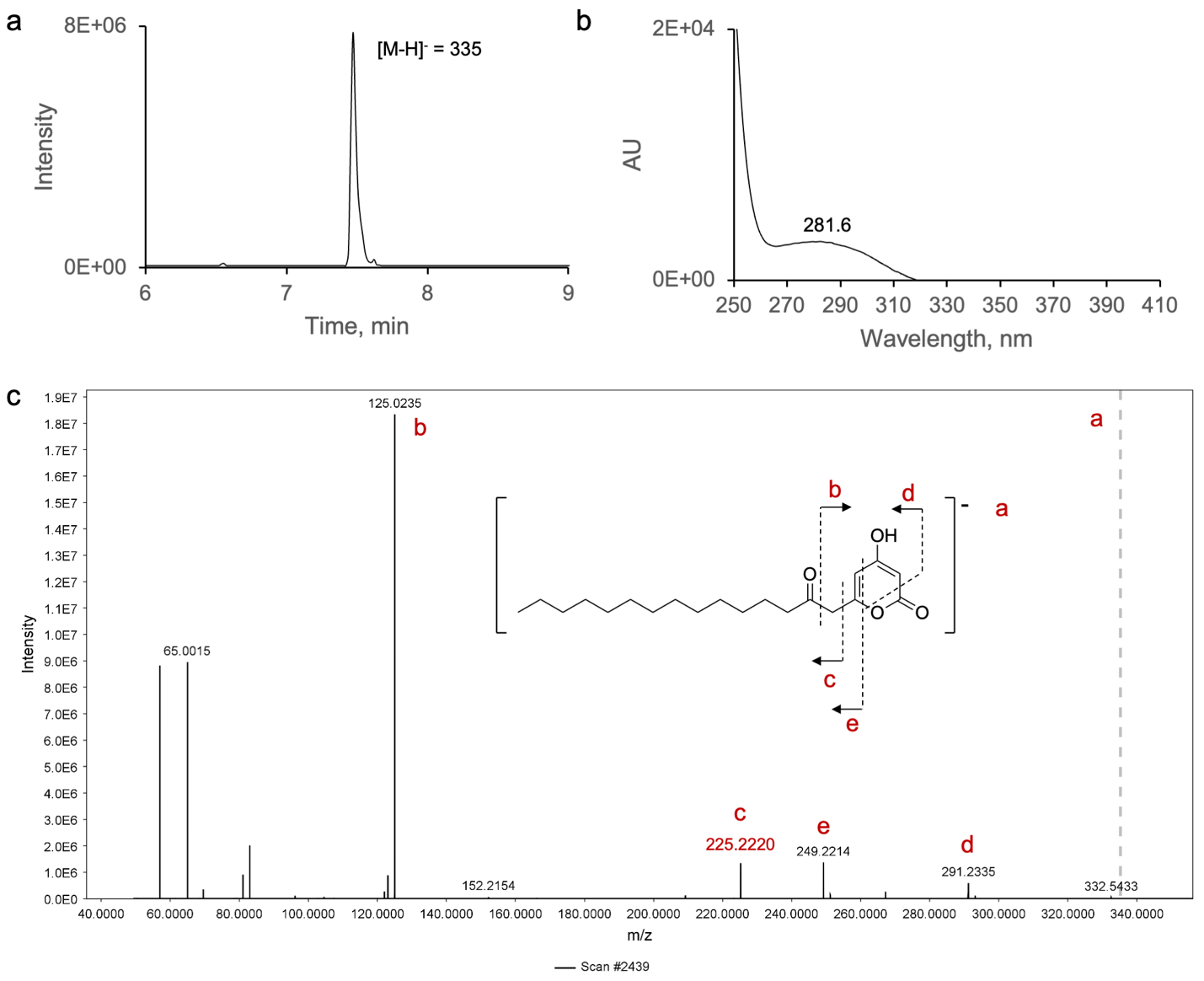


**Figure S29.** Spectral data analysis of product **9b**. a) Low-resolution LC-MS analysis of the EtOAc extract of the enzymatic reaction of DhelPKS with **9**; extracted ion chromatogram of the predicted *m/z* of 335 (negative mode). Y-axis shows relative ion intensity. b) Corresponding UV absorption spectrum. c) ESI-HR-MS/MS (negative mode) with ions matching expected fragments of **9b**; observed *m/z* = 335.2231 (theoretical *m/z* = 335.2228, calculated for [C_20_H_31_O_4_]^-^). The precursor ion is indicated with a dashed grey line.


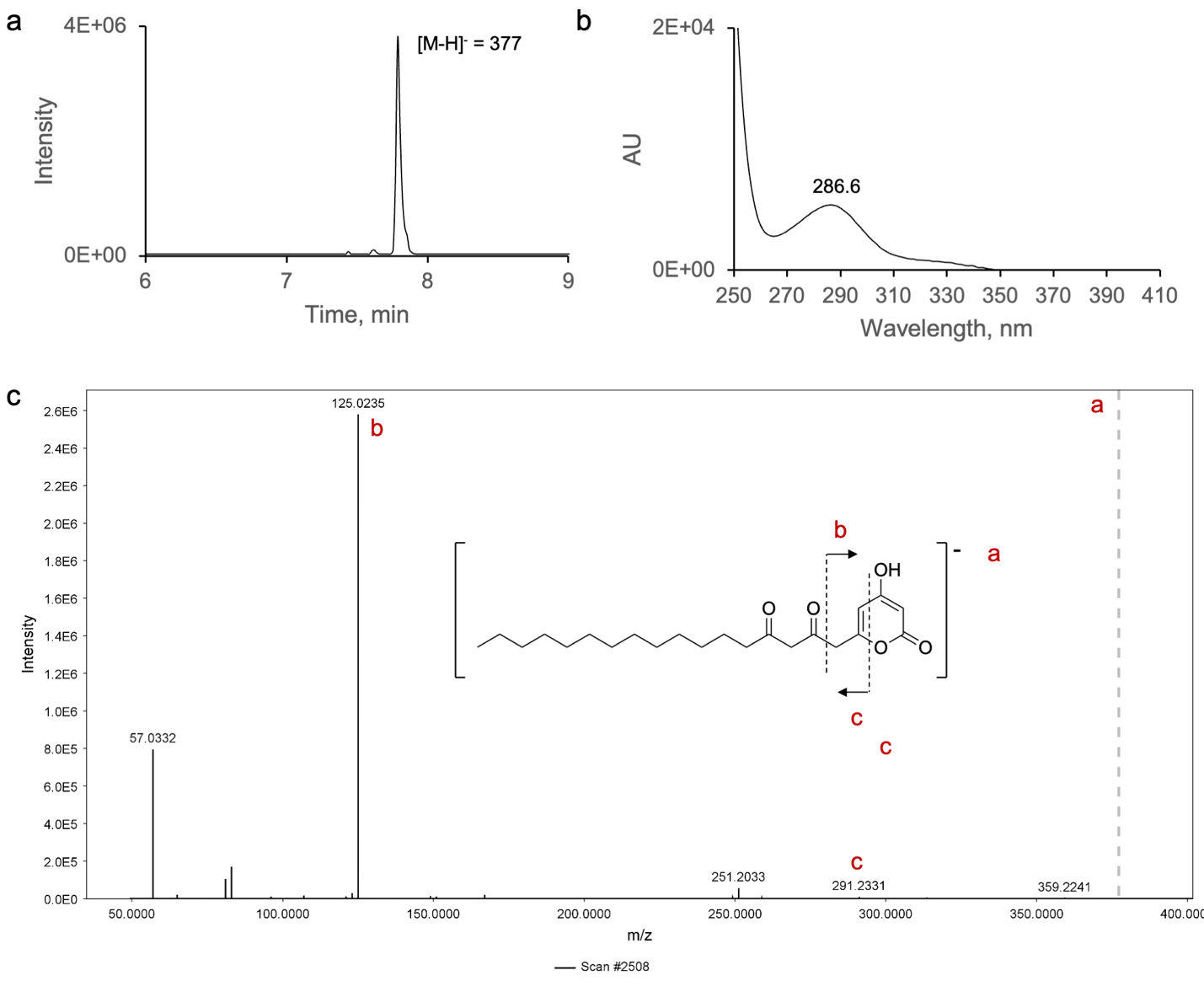


**Figure S30.** Spectral data analysis of product **9c**. a) Low-resolution LC-MS analysis of the EtOAc extract of the enzymatic reaction of HargPKS1 with **9**; extracted ion chromatogram of the predicted *m/z* of 377 (negative mode). Y-axis shows relative ion intensity. b) Corresponding UV absorption spectrum. c) ESI-HR-MS/MS (negative mode) with ions matching expected fragments of **9c**; observed *m/z* = 377.2338 (theoretical *m/z* = 377.2333, calculated for [C_22_H_33_O_5_]^-^). The precursor ion is indicated with a dashed grey line.


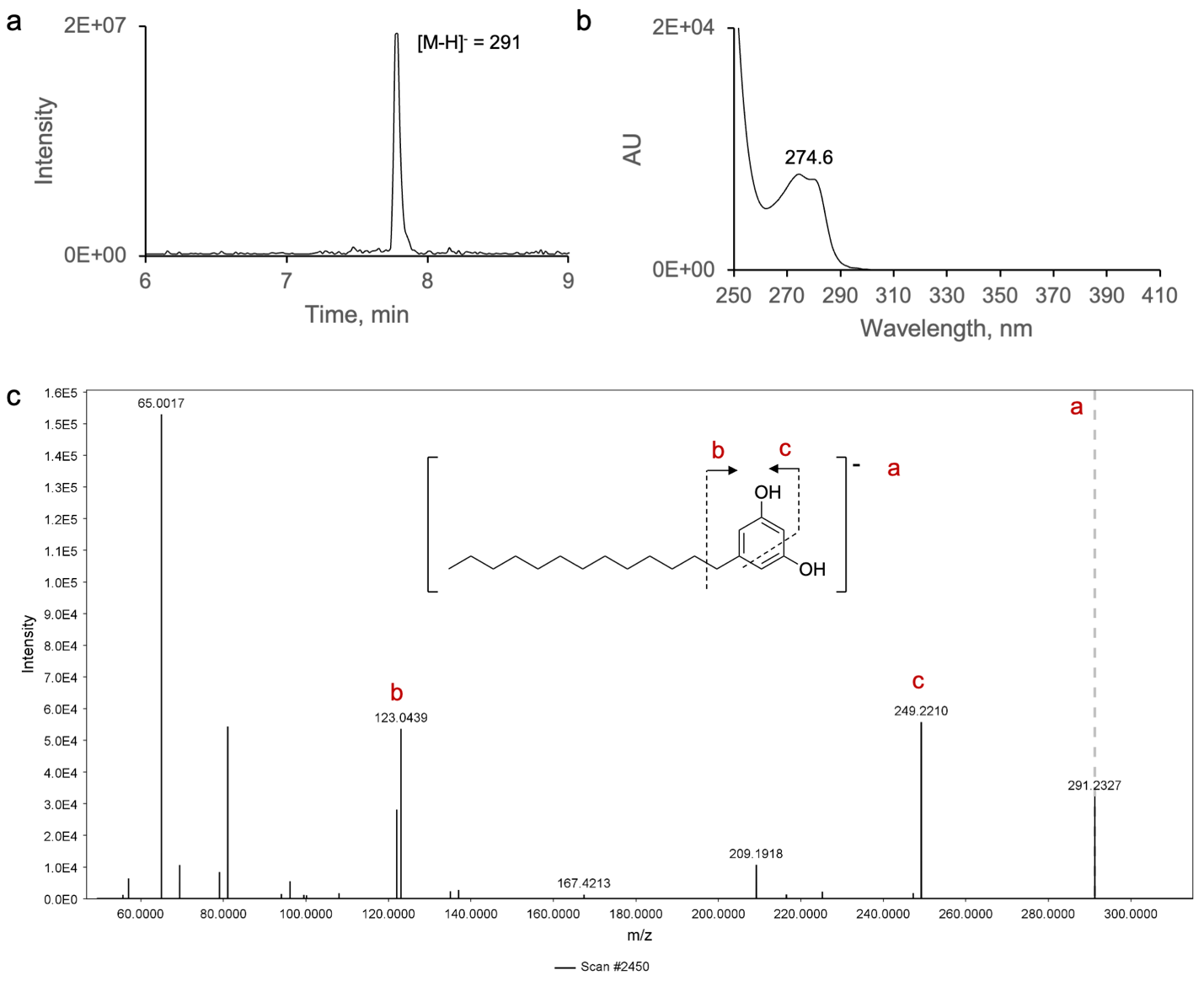


**Figure S31.** Spectral data analysis of product **9d**. a) Low-resolution LC-MS analysis of the EtOAc extract of the enzymatic reaction of DhelPKS with **9**; extracted ion chromatogram of the predicted *m/z* of 291 (negative mode). Y-axis shows relative ion intensity. b) Corresponding UV absorption spectrum. c) ESI-HR-MS/MS (negative mode) with ions matching expected fragments of **9d**; observed *m/z* = 291.2330 (theoretical *m/z* = 291.2330, calculated for [C_19_H_31_O_2_]^-^). The precursor ion is indicated with a dashed grey line.


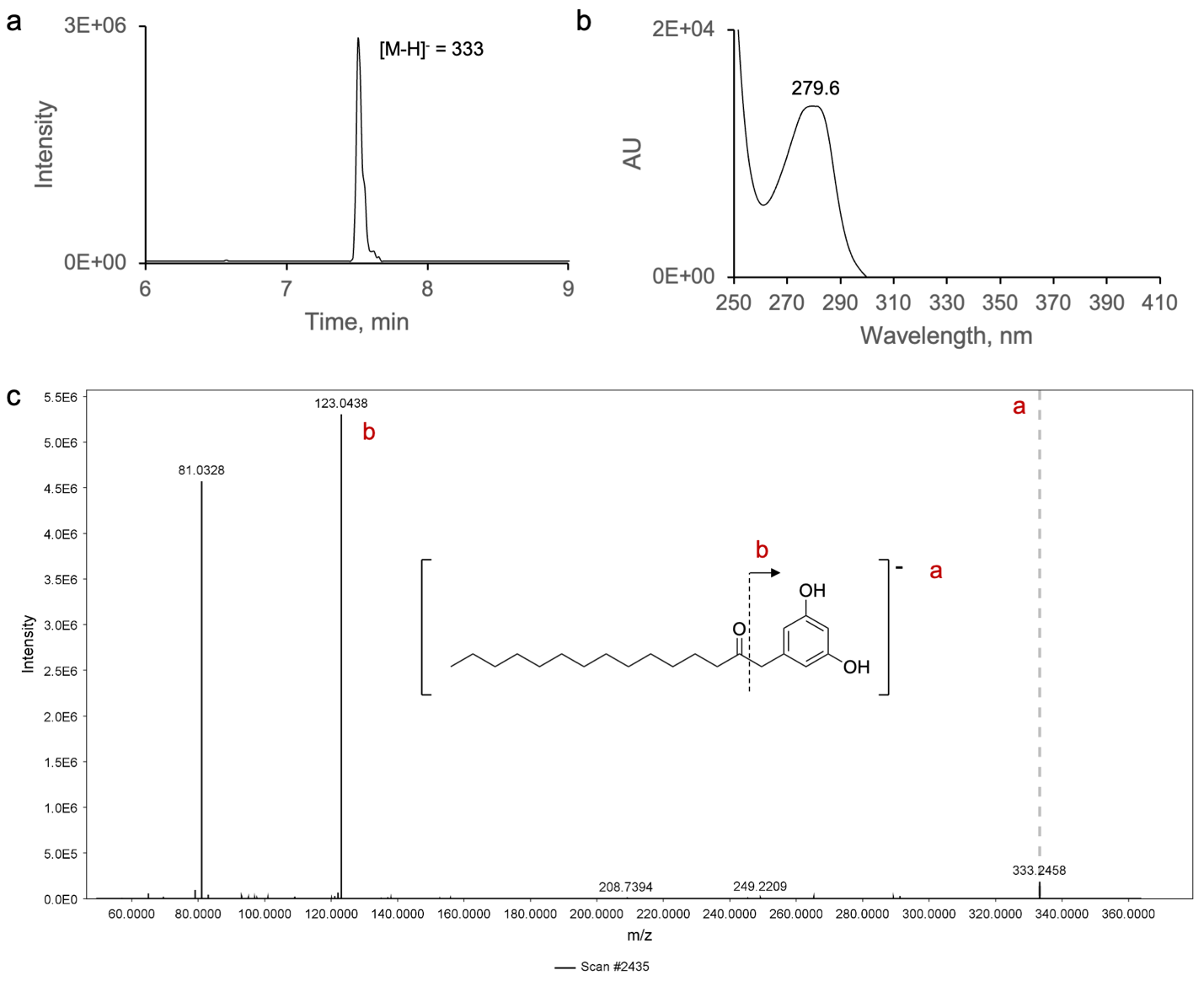


**Figure S32.** Spectral data analysis of product **9e**. a) Low-resolution LC-MS analysis of the EtOAc extract of the enzymatic reaction of AserPKS2 with **9**; extracted ion chromatogram of the predicted *m/z* of 333 (negative mode). Y-axis shows relative ion intensity. b) Corresponding UV absorption spectrum. c) ESI-HR-MS/MS (negative mode) with ions matching expected fragments of **9e**; observed *m/z* = 333.2438 (theoretical *m/z* = 333.2435, calculated for [C_21_H_33_O_3_]^-^). The precursor ion is indicated with a dashed grey line.


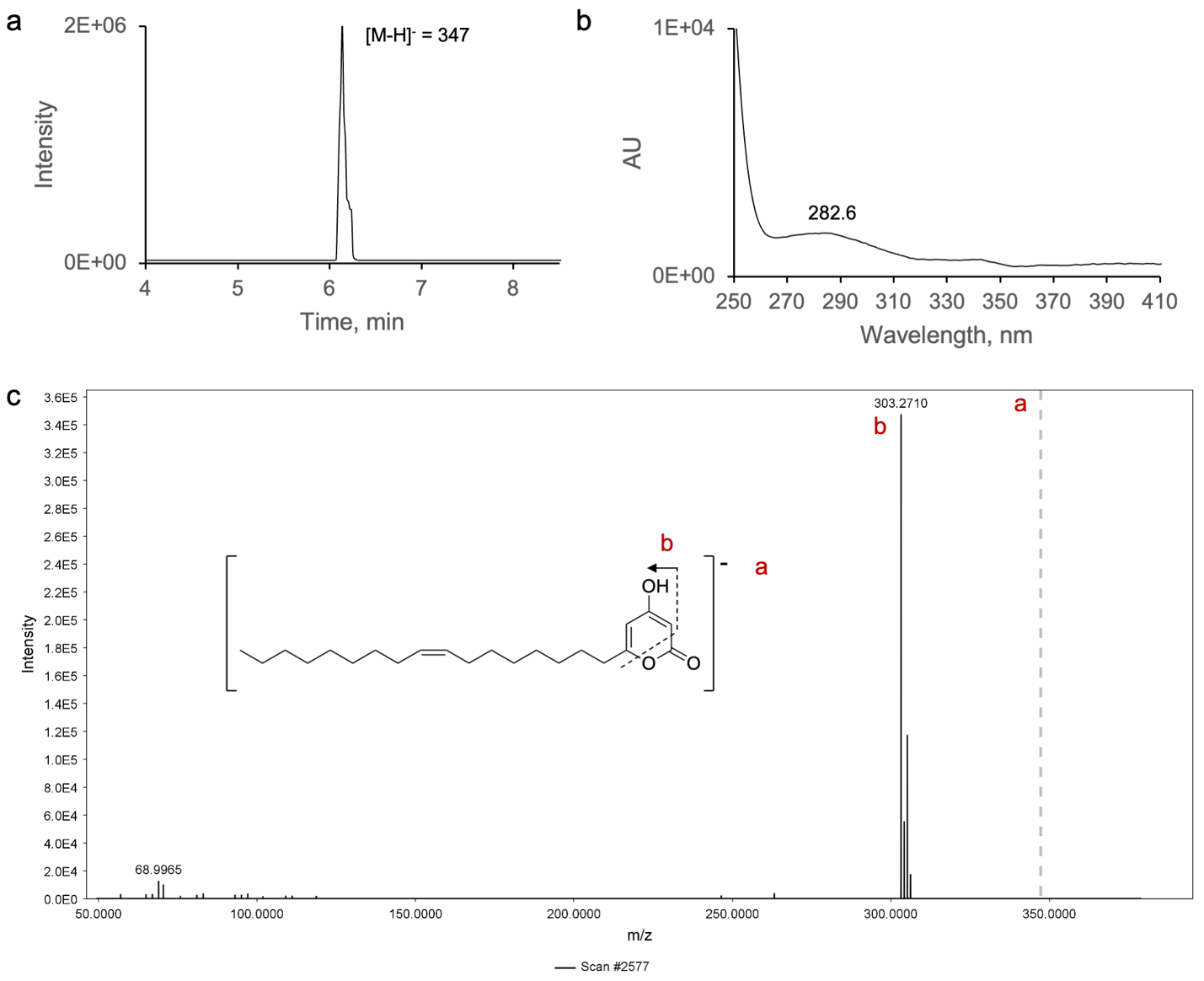


**Figure S33.** Spectral data analysis of product **10a**. a) Low-resolution LC-MS analysis of the EtOAc extract of the enzymatic reaction of VmalPKS with **10**; extracted ion chromatogram of the predicted *m/z* of 347 (negative mode). Y-axis shows relative ion intensity. b) Corresponding UV absorption spectrum. c) ESI-HR-MS/MS (negative mode) with ions matching expected fragments of **10a**; observed *m/z* = 347.2590 (theoretical *m/z* = 347.2592, calculated for [C_22_H_35_O_3_]^-^). The precursor ion is indicated with a dashed grey line.


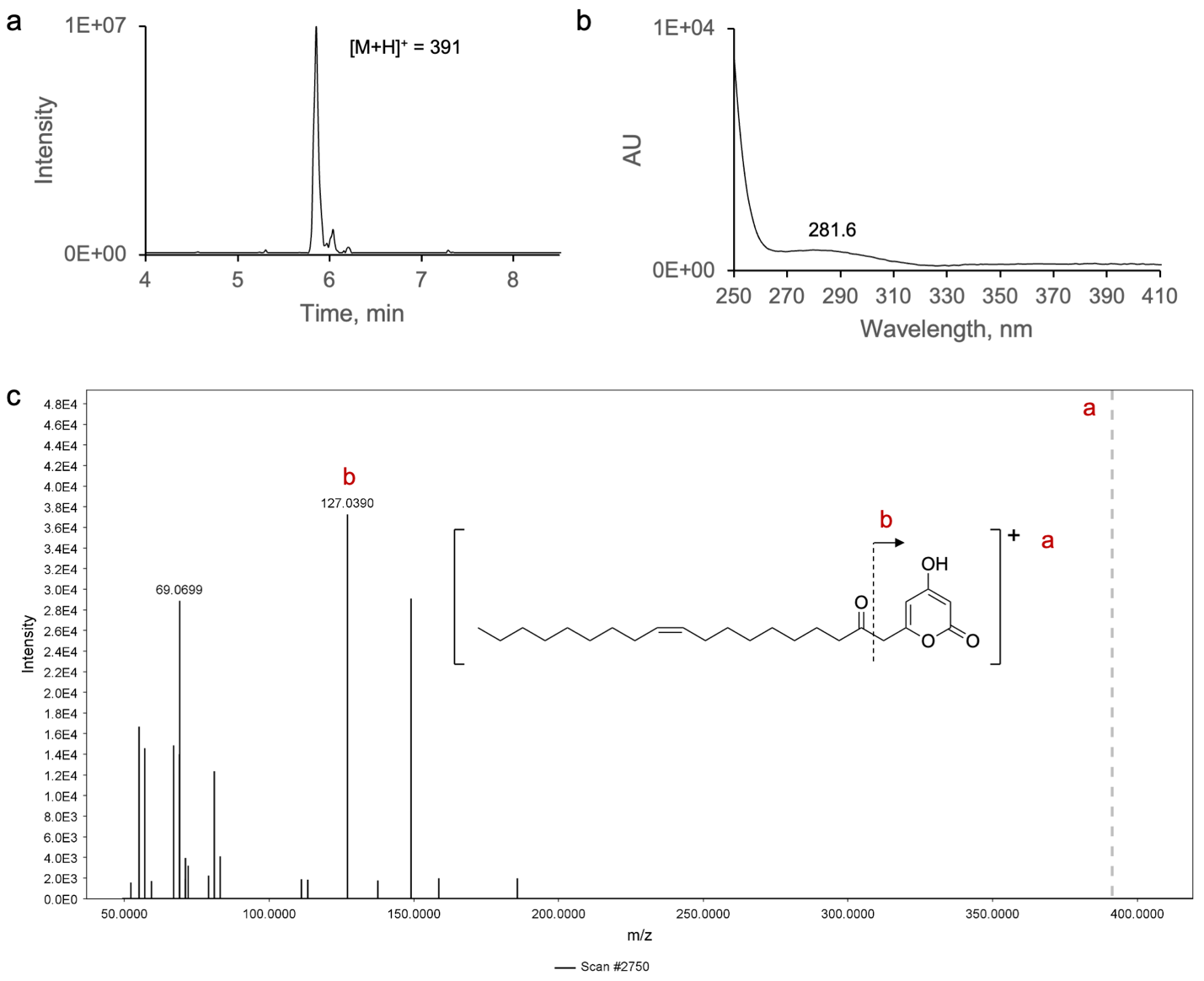


**Figure S34.** Spectral data analysis of product **10b**. a) Low-resolution LC-MS analysis of the EtOAc extract of the enzymatic reaction of AastPKS with **10**; extracted ion chromatogram of the predicted *m/z* of 391 (positive mode). Y-axis shows relative ion intensity. b) Corresponding UV absorption spectrum. c) ESI-HR-MS/MS (positive mode) with ions matching expected fragments of **10b**; observed *m/z* = 391.2847 (theoretical *m/z* = 391.2843, calculated for [C_24_H_39_O_4_]^+^). The precursor ion is indicated with a dashed grey line.


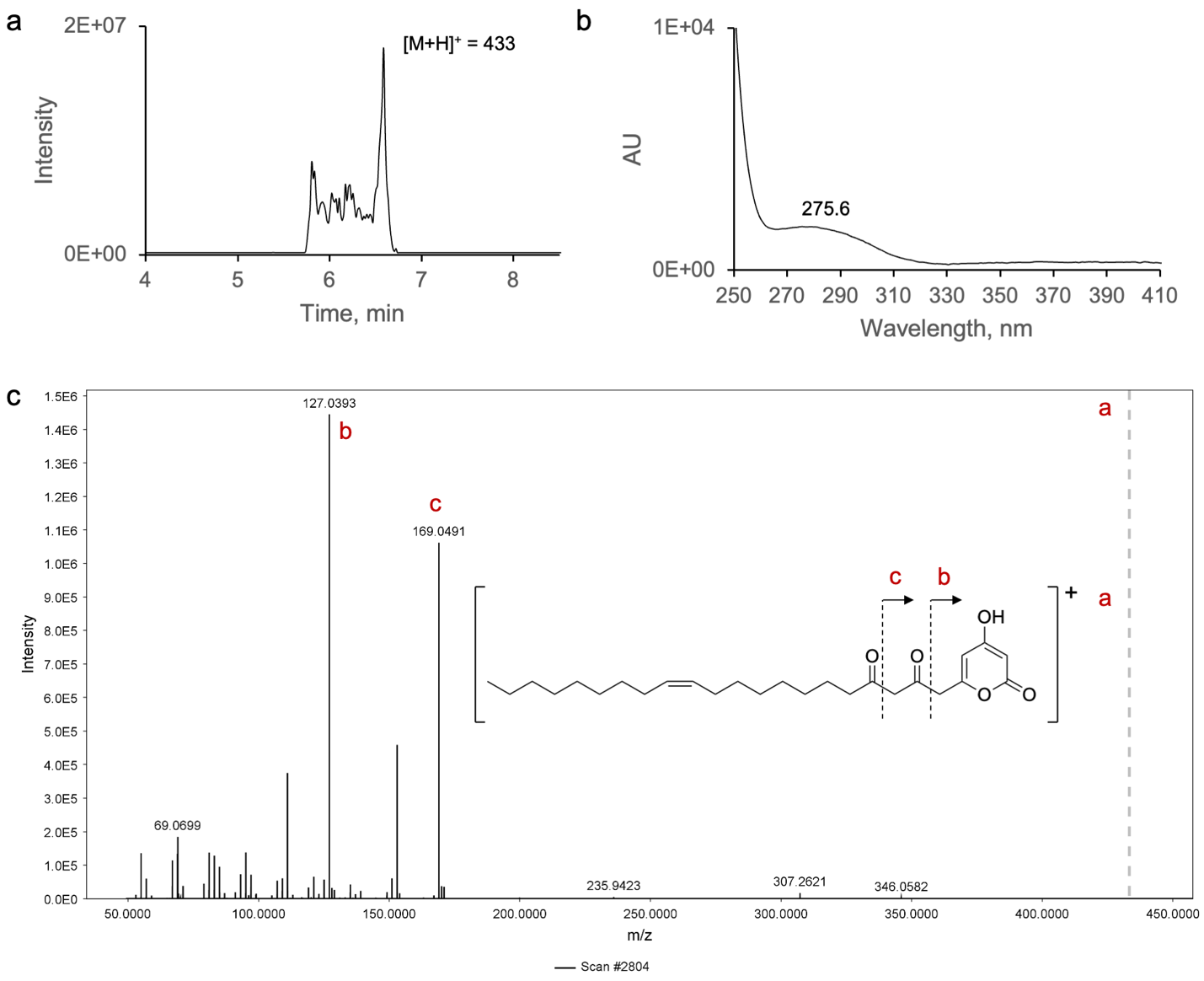


**Figure S35.** Spectral data analysis of product **10c**. a) Low-resolution LC-MS analysis of the EtOAc extract of the enzymatic reaction of AastPKS with **10**; extracted ion chromatogram of the predicted *m/z* of 433 (positive mode). Y-axis shows relative ion intensity. b) Corresponding UV absorption spectrum. c) ESI-HR-MS/MS (positive mode) with ions matching expected fragments of **10c**; observed *m/z* = 433.2955 (theoretical *m/z* = 433.2949, calculated for [C_26_H_41_O_5_]^+^). The precursor ion is indicated with a dashed grey line.


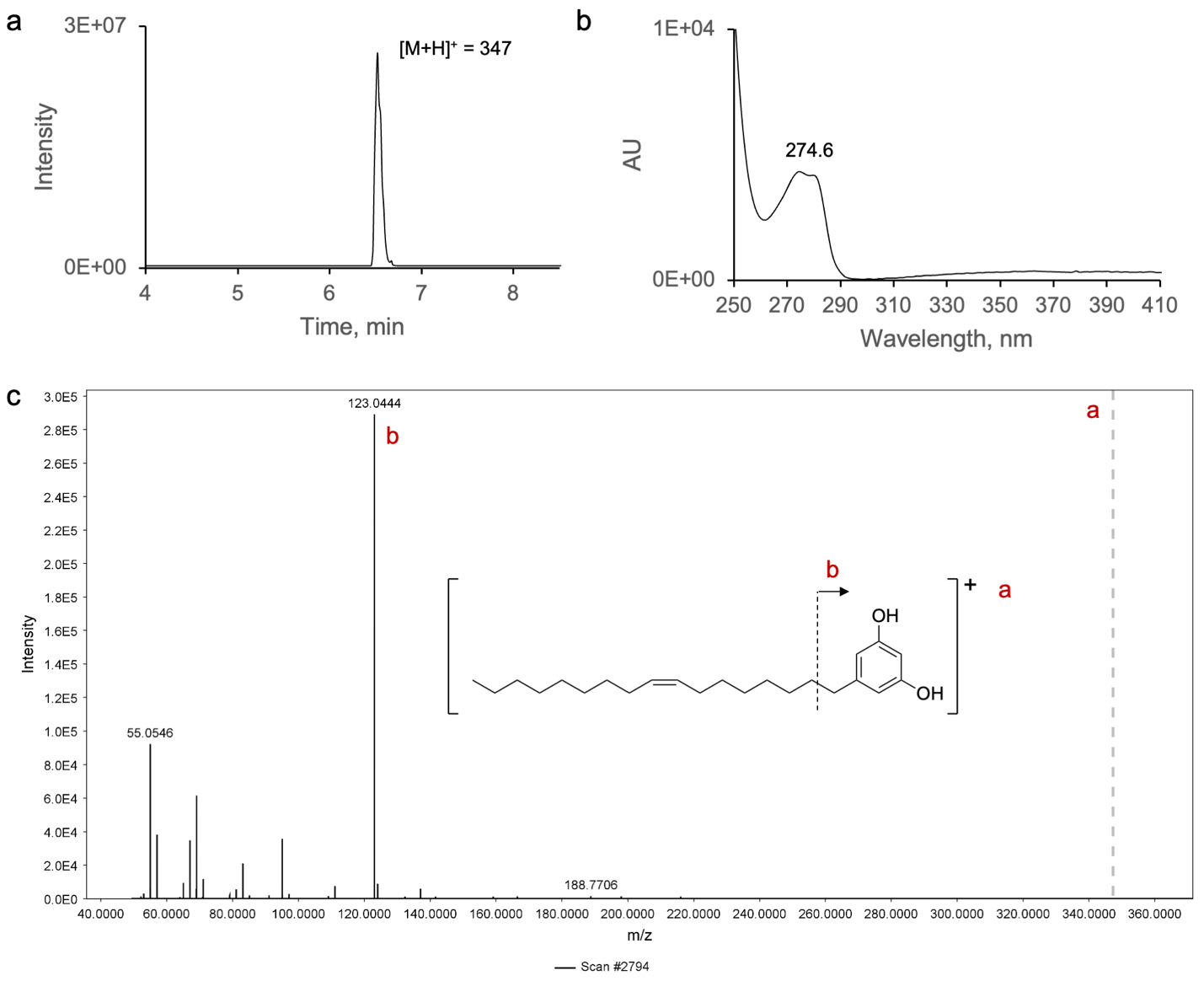


**Figure S36.** Spectral data analysis of product **10d**. a) Low-resolution LC-MS analysis of the EtOAc extract of the enzymatic reaction of AserPKS2 with **10**; extracted ion chromatogram of the predicted *m/z* of 347 (positive mode). Y-axis shows relative ion intensity. b) Corresponding UV absorption spectrum. c) ESI-HR-MS/MS (positive mode) with ions matching expected fragments of **10d**; observed *m/z* = 347.2948 (theoretical *m/z* = 347.2945, calculated for [C_23_H_39_O_2_]^+^). The precursor ion is indicated with a dashed grey line.


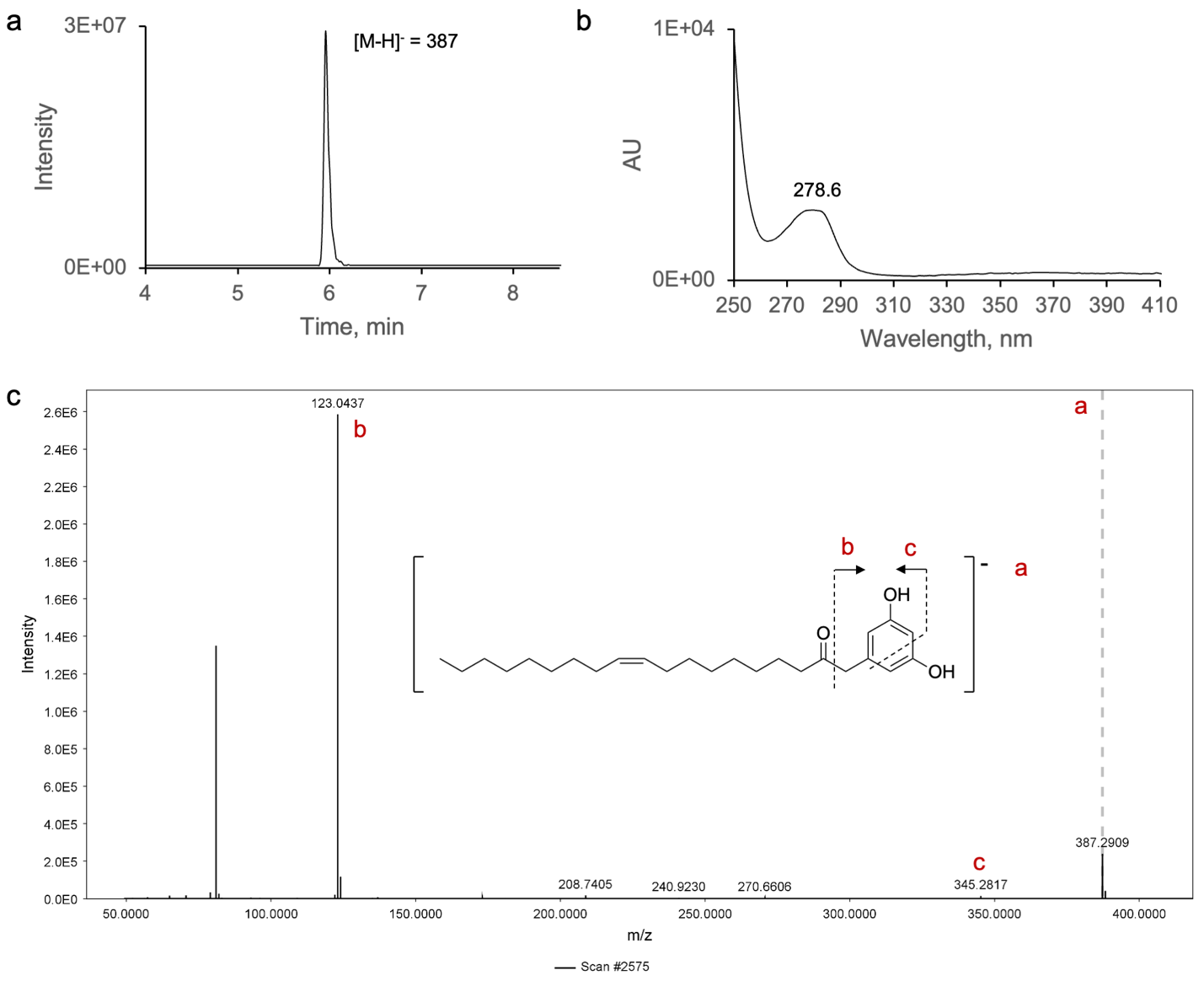


**Figure S37.** Spectral data analysis of product **10e**. a) Low-resolution LC-MS analysis of the EtOAc extract of the enzymatic reaction of VmalPKS with **10**; extracted ion chromatogram of the predicted *m/z* of 387 (negative mode). Y-axis shows relative ion intensity. b) Corresponding UV absorption spectrum. c) ESI-HR-MS/MS (negative mode) with ions matching expected fragments of **10e**; observed *m/z* = 387.2904 (theoretical *m/z* = 387.2905, calculated for C25H39O3-). The precursor ion is indicated with a dashed grey line.


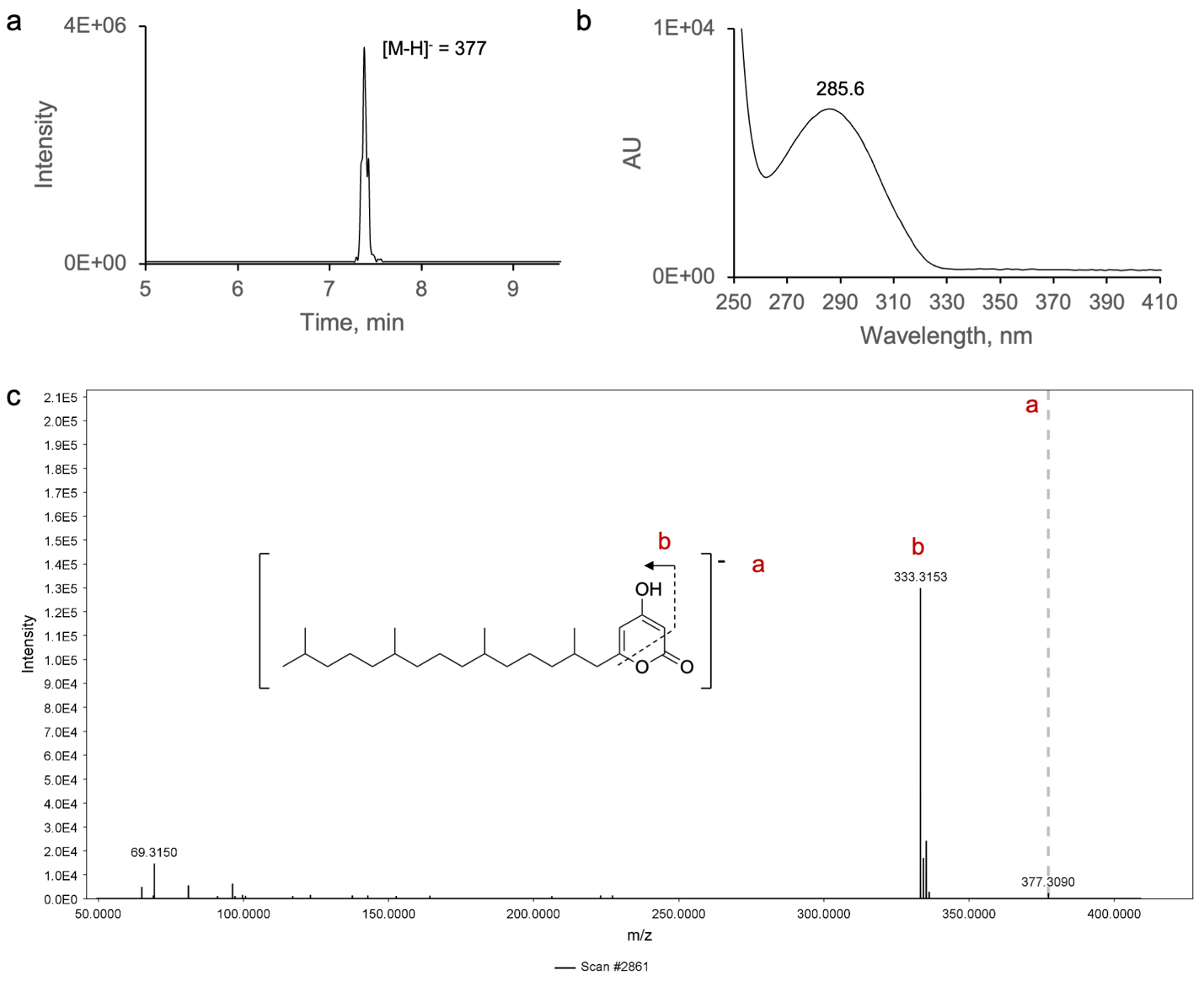


**Figure S38.** Spectral data analysis of product **11a**. a) Low-resolution LC-MS analysis of the EtOAc extract of the enzymatic reaction of AserPKS2 with **11**; extracted ion chromatogram of the predicted *m/z* of 377 (negative mode). Y-axis shows relative ion intensity. b) Corresponding UV absorption spectrum. c) ESI-HR-MS/MS (negative mode) with ions matching expected fragments of **11a**; observed *m/z* = 377.3059 (theoretical *m/z* = 377.3061, calculated for [C_24_H_41_O_3_]^-^). The precursor ion is indicated with a dashed grey line.


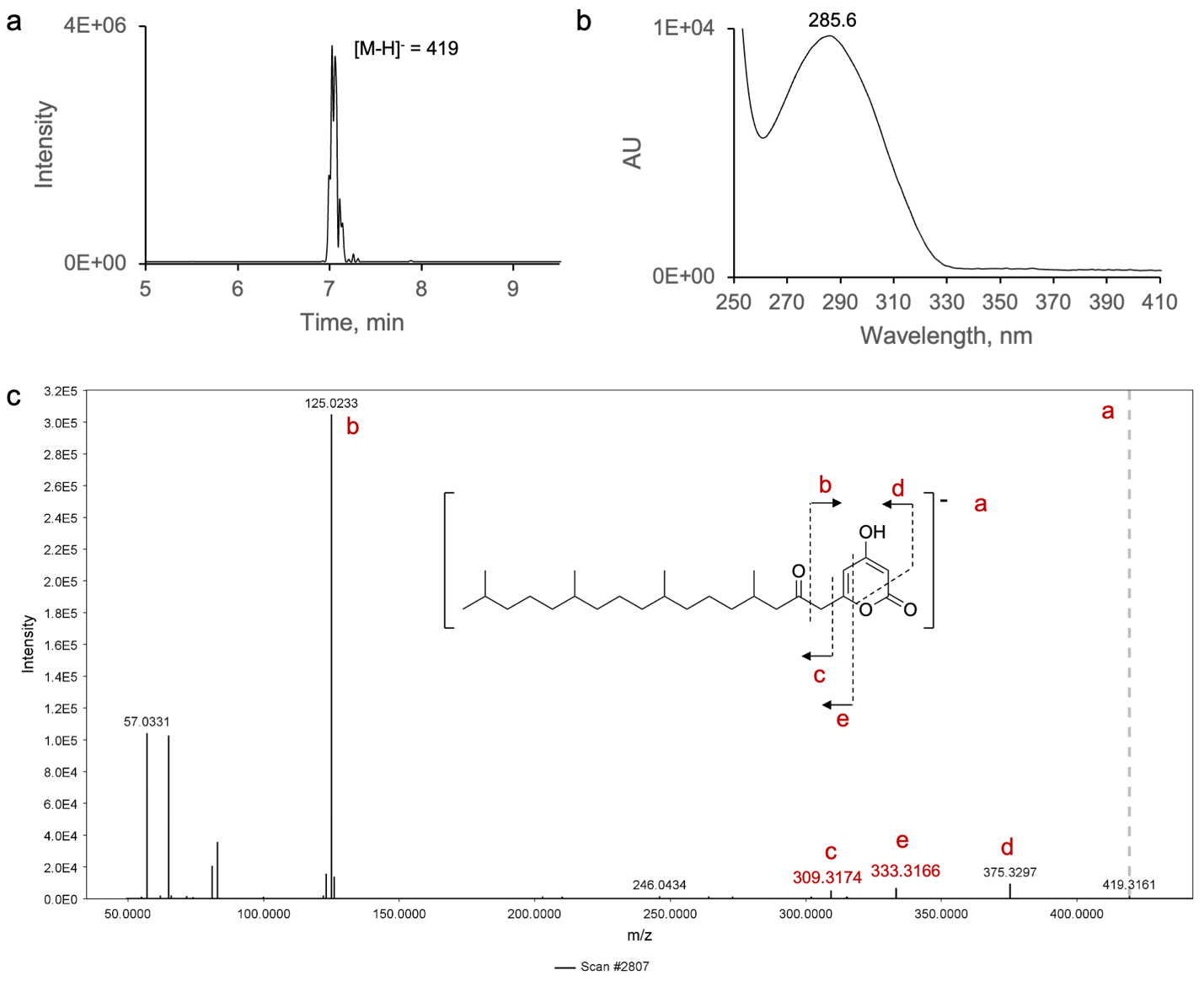


**Figure S39.** Spectral data analysis of product **11b**. a) Low-resolution LC-MS analysis of the EtOAc extract of the enzymatic reaction of AserPKS2 with **11**; extracted ion chromatogram of the predicted *m/z* of 419 (negative mode). Y-axis shows relative ion intensity. b) Corresponding UV absorption spectrum. c) ESI-HR-MS/MS (negative mode) with ions matching expected fragments of **11b**; observed *m/z* = 419.3165 (theoretical *m/z* = 419.3167, calculated for [C_26_H_43_O_4_]^-^). The precursor ion is indicated with a dashed grey line.

**
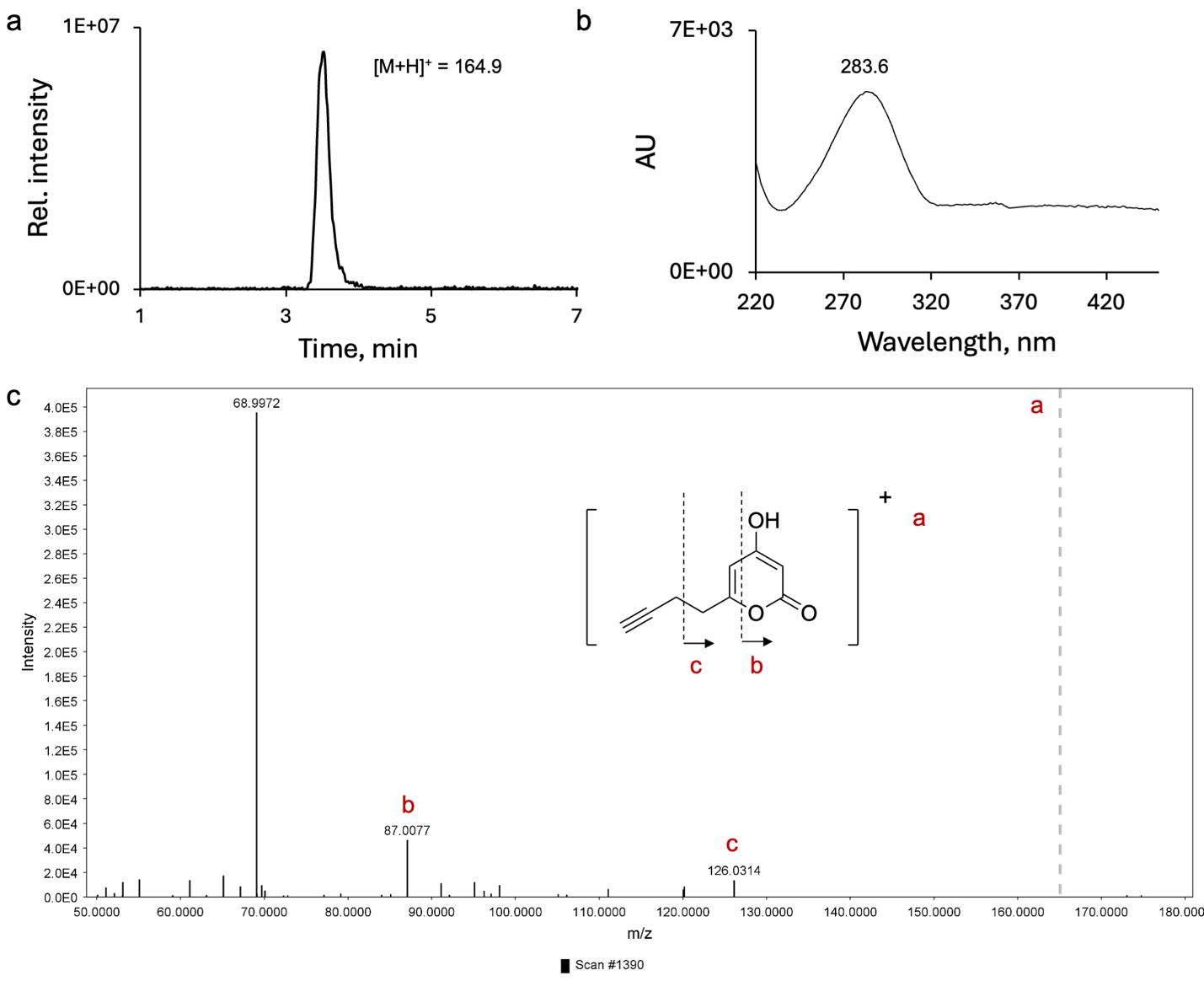
**

**Figure S40.** Spectral data analysis of product **13a**. a) Low-resolution LC-MS analysis of the EtOAc extract of the enzymatic reaction of HargPKS1 with **13**; extracted ion chromatogram of the predicted *m/z* of 165 (positive mode). Y-axis shows relative ion intensity. b) Corresponding UV absorption spectrum. c) ESI-HR-MS/MS (negative mode) with ions matching expected fragments of **13a**; observed *m/z* = 165.0545 (theoretical *m/z* = 165.0546, calculated for [C_9_H_9_O_3_]^+^). The precursor ion is indicated with a dashed grey line.

**
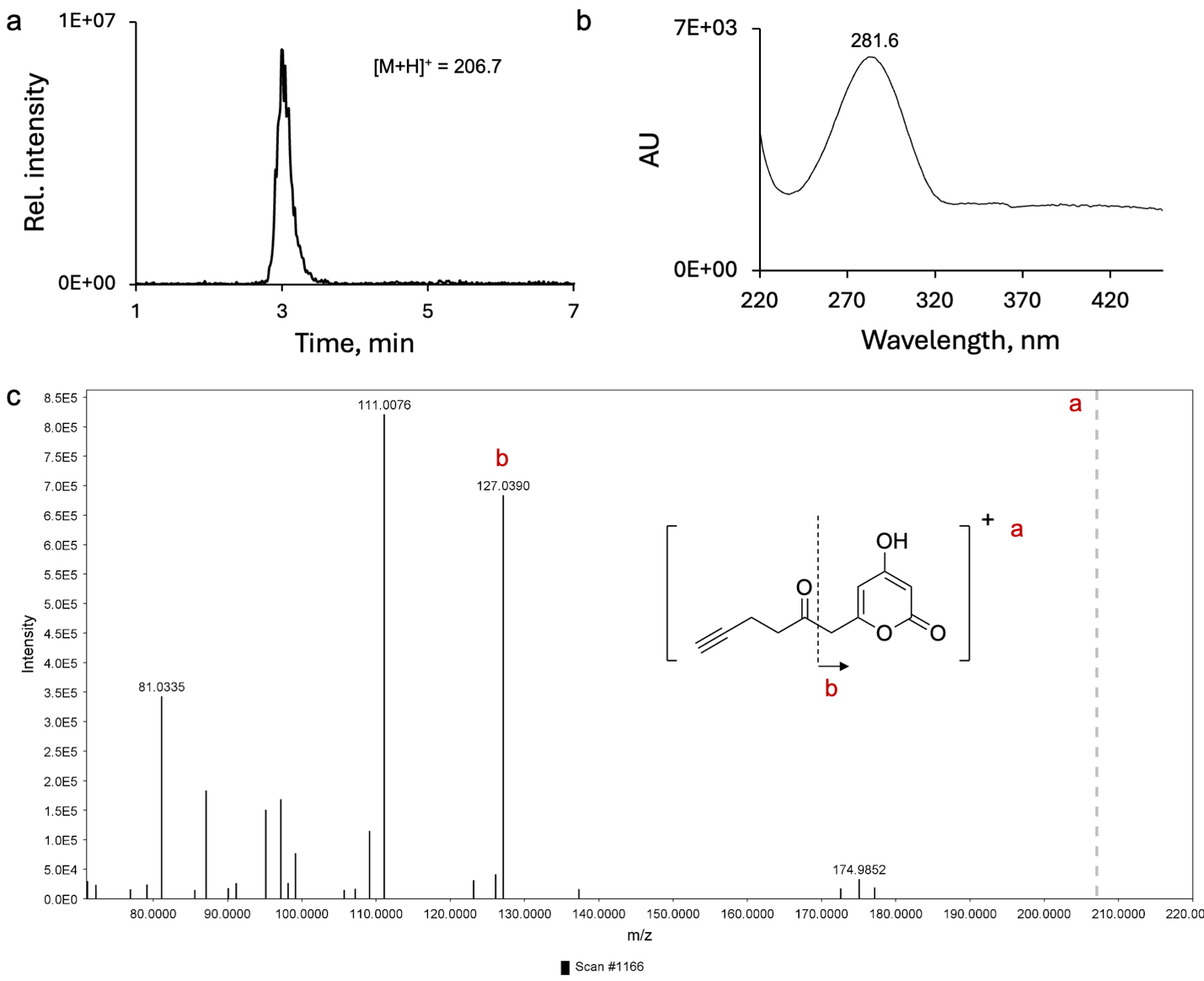
**

**Figure S41.** Spectral data analysis of product **13b**. a) Low-resolution LC-MS analysis of the EtOAc extract of the enzymatic reaction of HargPKS1 with **13**; extracted ion chromatogram of the predicted *m/z* of 207 (positive mode). Y-axis shows relative ion intensity. b) Corresponding UV absorption spectrum. c) ESI-HR-MS/MS (negative mode) with ions matching expected fragments of **13b**; observed *m/z* = 207.0649 (theoretical *m/z* = 207.0652, calculated for [C_11_H_11_O_4_]^+^). The precursor ion is indicated with a dashed grey line.

**
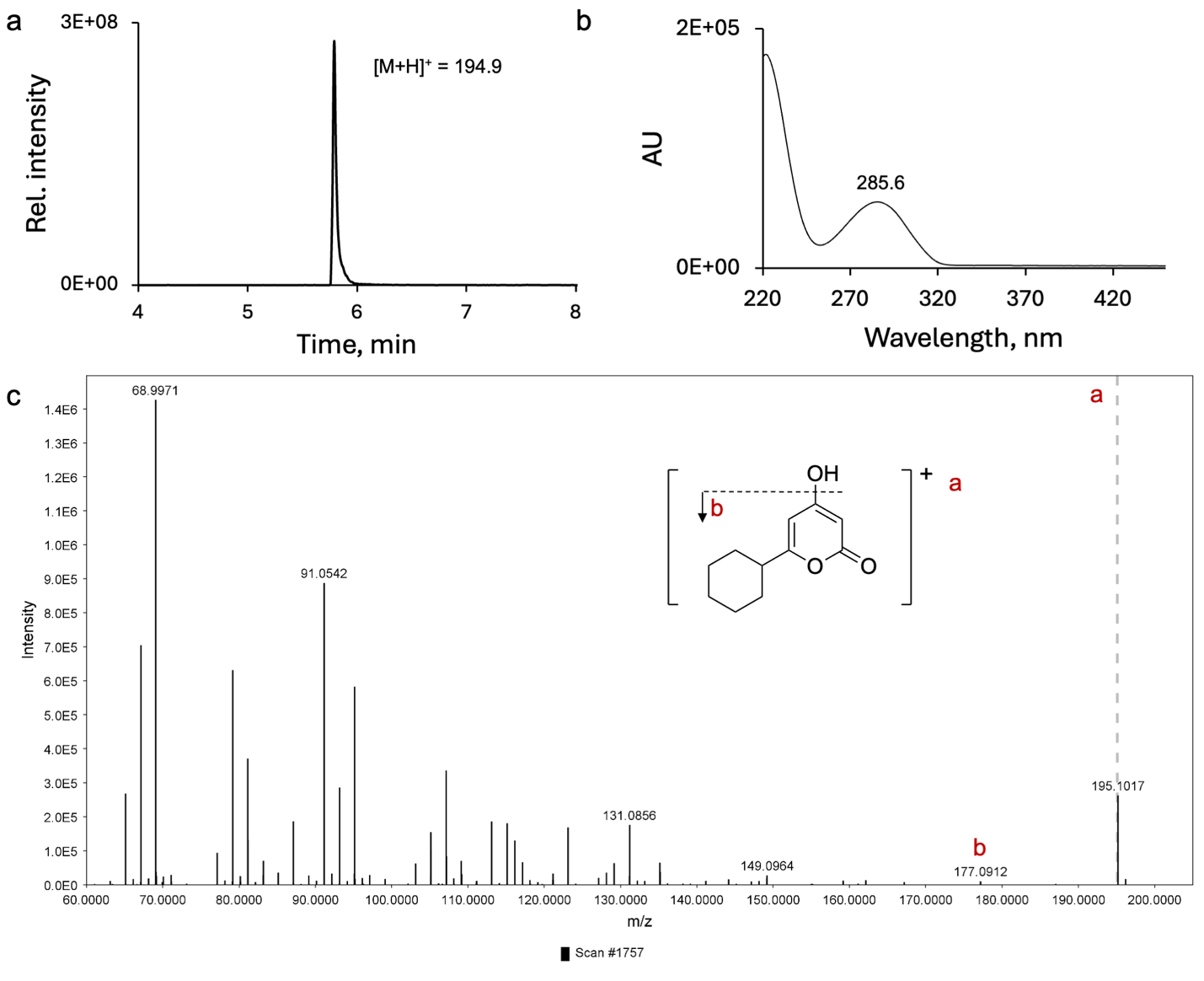
**

**Figure S42.** Spectral data analysis of product **14a**. a) Low-resolution LC-MS analysis of the EtOAc extract of the enzymatic reaction of AiizPKS with **14**; extracted ion chromatogram of the predicted *m/z* of 195 (positive mode). Y-axis shows relative ion intensity. b) Corresponding UV absorption spectrum. c) ESI-HR-MS/MS (negative mode) with ions matching expected fragments of **14a**; observed *m/z* = 195.1014 (theoretical *m/z* = 195.1016, calculated for [C_11_H_15_O_3_]^+^). The precursor ion is indicated with a dashed grey line.

**
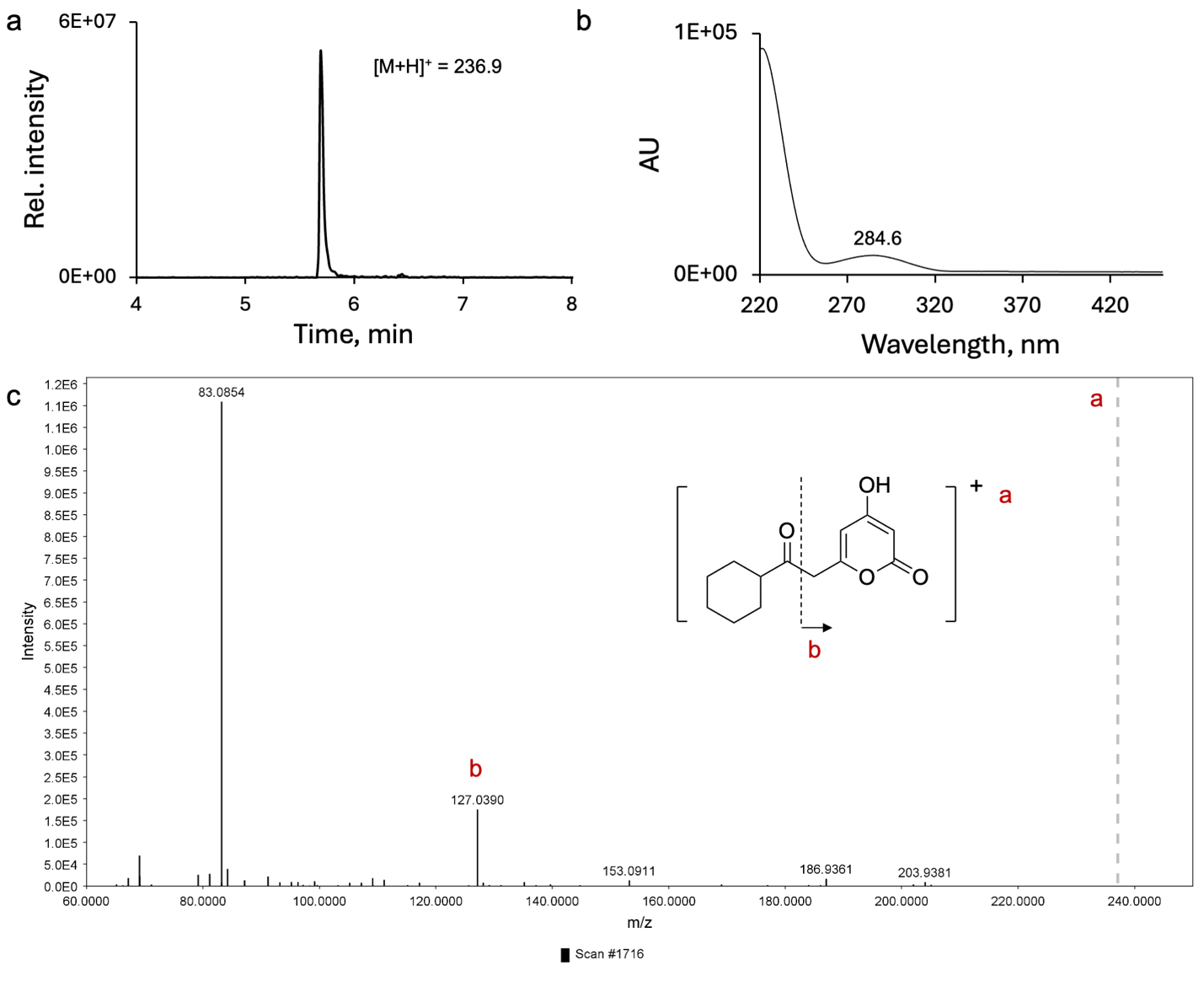
**

**Figure S43.** Spectral data analysis of product **14b**. a) Low-resolution LC-MS analysis of the EtOAc extract of the enzymatic reaction of AiizPKS with **14**; extracted ion chromatogram of the predicted *m/z* of 237 (positive mode). Y-axis shows relative ion intensity. b) Corresponding UV absorption spectrum. c) ESI-HR-MS/MS (negative mode) with ions matching expected fragments of **14b**; observed *m/z* = 237.1118 (theoretical *m/z* = 237.1121, calculated for [C_13_H_17_O_4_]^+^). The precursor ion is indicated with a dashed grey line.

**
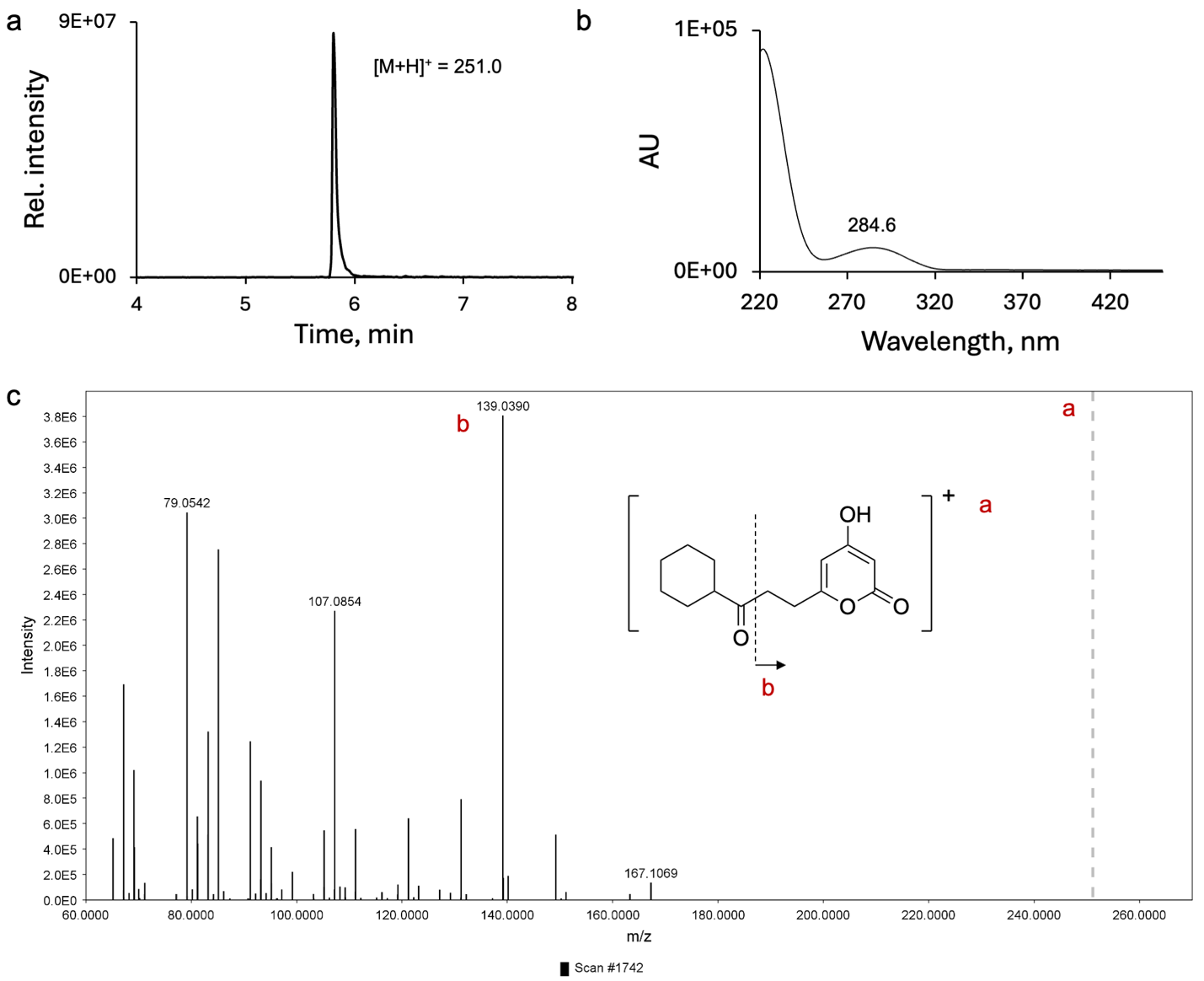
**

**Figure S44.** Spectral data analysis of product **15a**. a) Low-resolution LC-MS analysis of the EtOAc extract of the enzymatic reaction of AiizPKS with **15**; extracted ion chromatogram of the predicted *m/z* of 251 (positive mode). Y-axis shows relative ion intensity. b) Corresponding UV absorption spectrum. c) ESI-HR-MS/MS (negative mode) with ions matching expected fragments of **15a**; observed *m/z* = 251.1275 (theoretical *m/z* = 251.1278, calculated for [C_14_H_19_O_4_]^+^). The precursor ion is indicated with a dashed grey line.

**
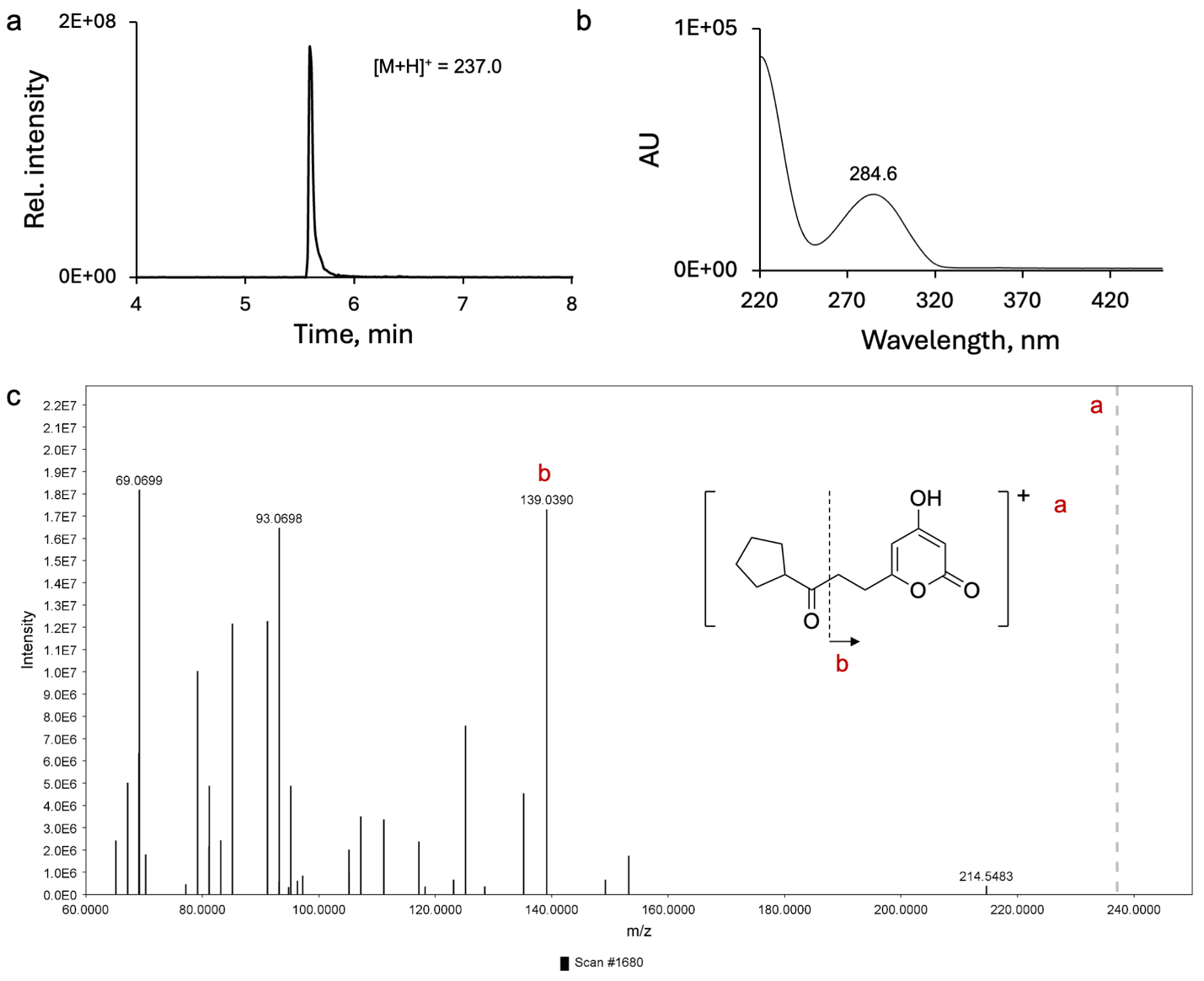
**

**Figure S45.** Spectral data analysis of product **16a**. a) Low-resolution LC-MS analysis of the EtOAc extract of the enzymatic reaction of TtonPKS with **16**; extracted ion chromatogram of the predicted *m/z* of 237 (positive mode). Y-axis shows relative ion intensity. b) Corresponding UV absorption spectrum. c) ESI-HR-MS/MS (negative mode) with ions matching expected fragments of **16a**; observed *m/z* = 237.1118 (theoretical *m/z* = 237.1121, calculated for [C_13_H_17_O_4_]^+^). The precursor ion is indicated with a dashed grey line.

**
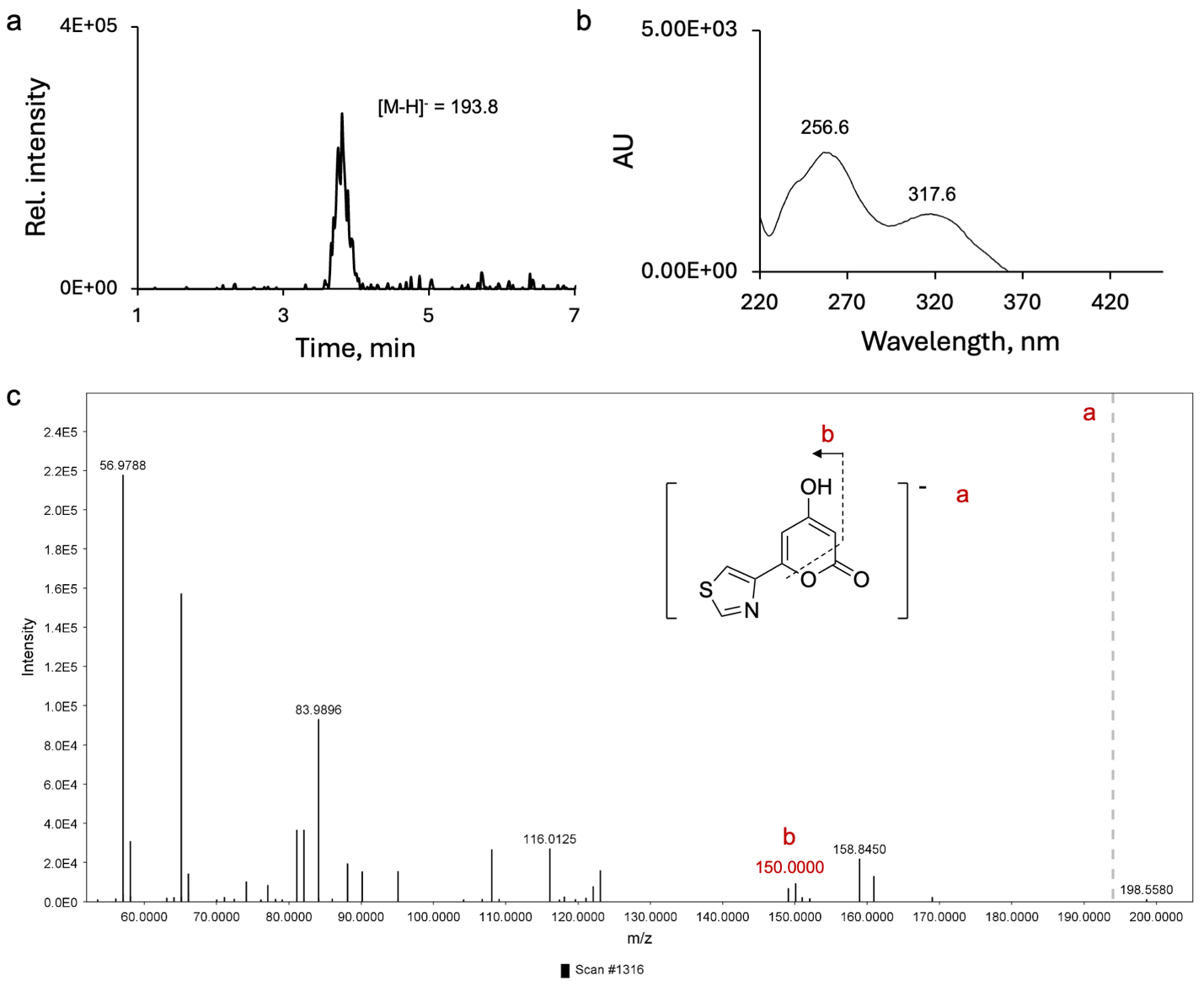
**

**Figure S46.** Spectral data analysis of product **17a**. a) Low-resolution LC-MS analysis of the EtOAc extract of the enzymatic reaction of AiizPKS with **17**; extracted ion chromatogram of the predicted *m/z* of 194 (negative mode). Y-axis shows relative ion intensity. b) Corresponding UV absorption spectrum. c) ESI-HR-MS/MS (negative mode) with ions matching expected fragments of **17a**; observed *m/z* = 193.9903 (theoretical *m/z* = 193.9917, calculated for [C_8_H_4_NO_3_S]^-^). The precursor ion is indicated with a dashed grey line.

**
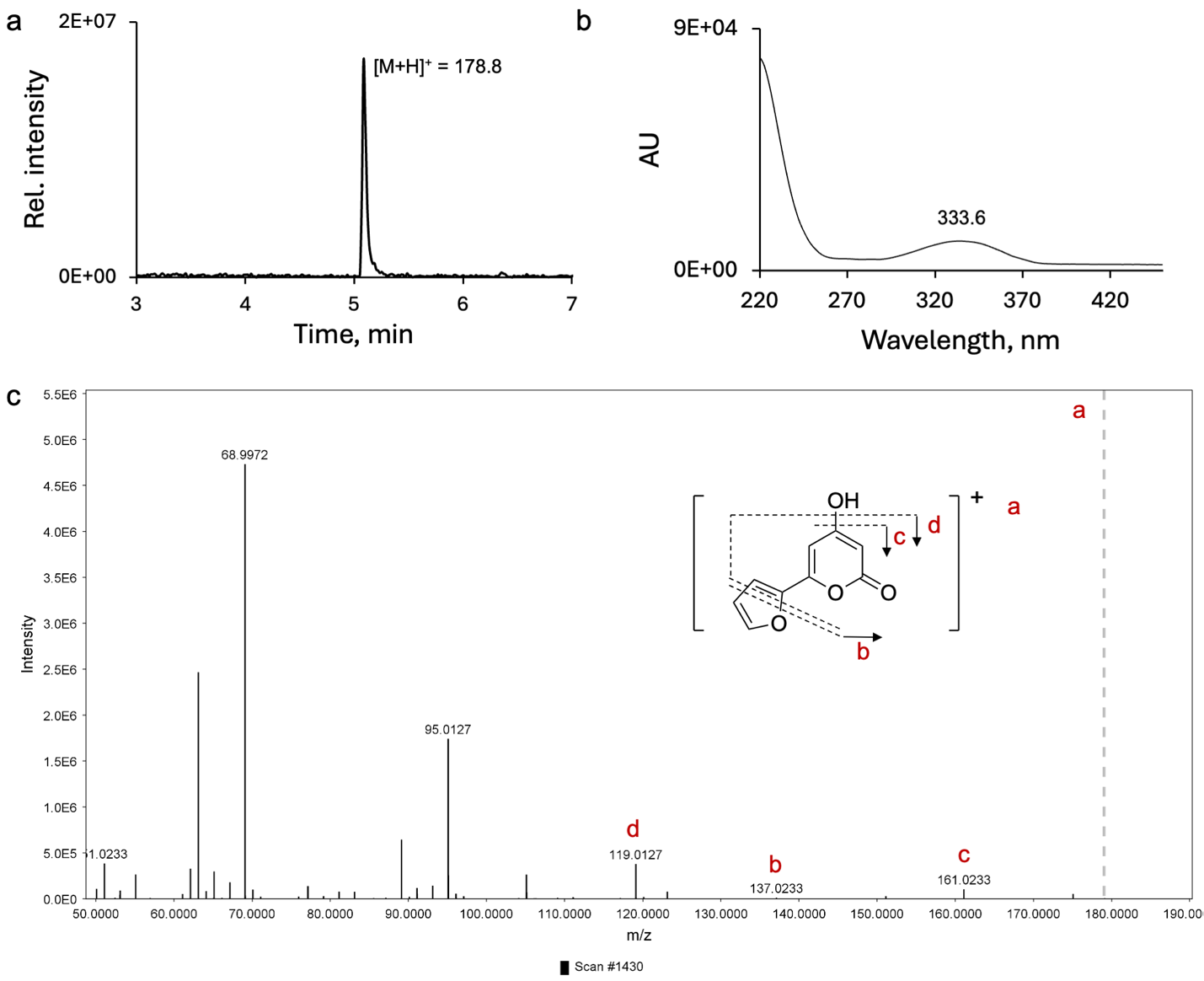
**

**Figure S47.** Spectral data analysis of product **18a**. a) Low-resolution LC-MS analysis of the EtOAc extract of the enzymatic reaction of AastPKS with **18**; extracted ion chromatogram of the predicted *m/z* of 179 (positive mode). Y-axis shows relative ion intensity. b) Corresponding UV absorption spectrum. c) ESI-HR-MS/MS (negative mode) with ions matching expected fragments of **18a**; observed *m/z* = 179.0338 (theoretical *m/z* = 179.0339, calculated for [C_9_H_7_O_4_]^+^). The precursor ion is indicated with a dashed grey line.

**
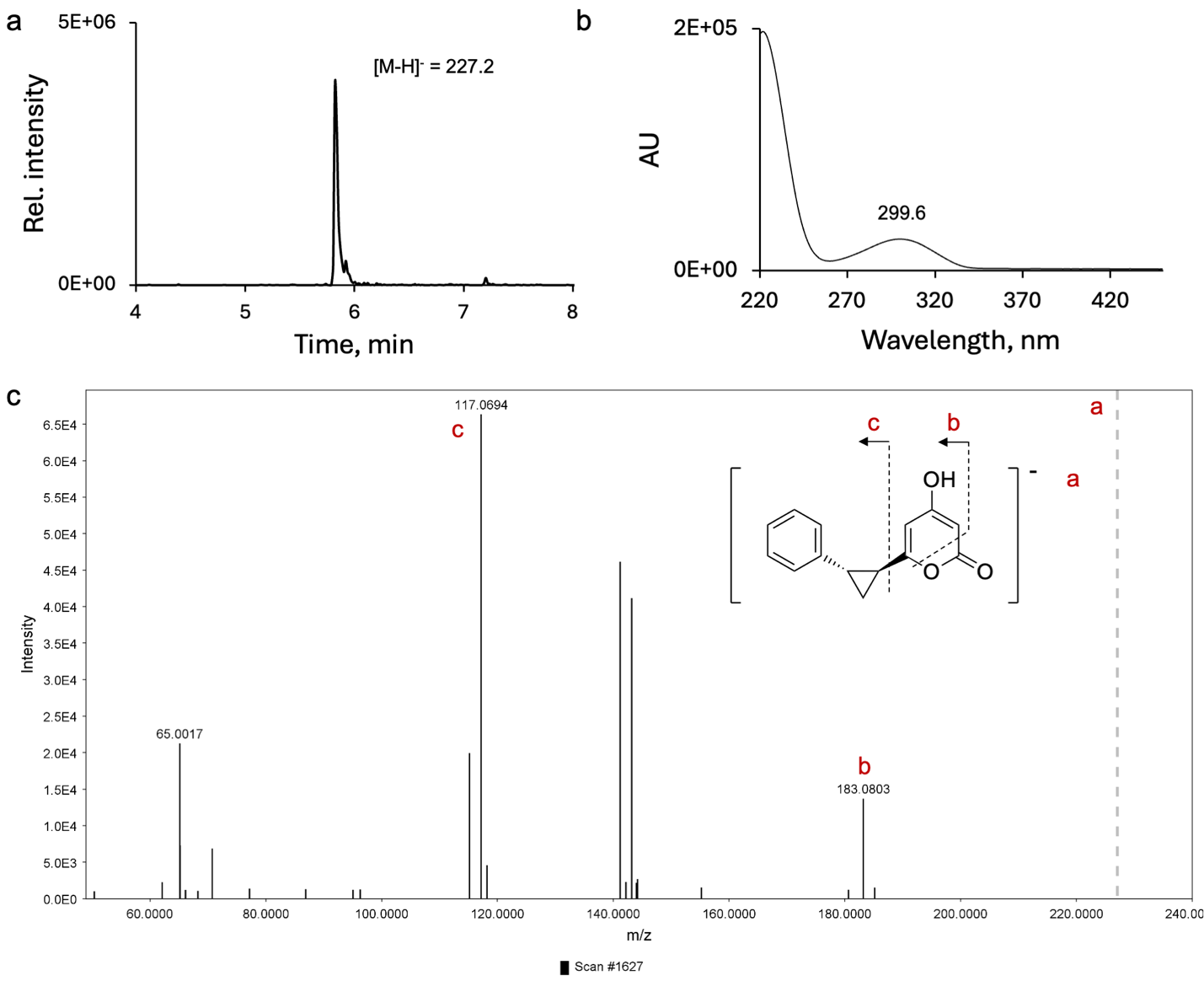
**

**Figure S48.** Spectral data analysis of product **19a**. a) Low-resolution LC-MS analysis of the EtOAc extract of the enzymatic reaction of TtonPKS with **19**; extracted ion chromatogram of the predicted *m/z* of 227 (negative mode). Y-axis shows relative ion intensity. b) Corresponding UV absorption spectrum. c) ESI-HR-MS/MS (negative mode) with ions matching expected fragments of **19a**; observed *m/z* = 227.0711 (theoretical *m/z* = 227.0714, calculated for [C_14_H_11_O_3_]^-^). The precursor ion is indicated with a dashed grey line.

**
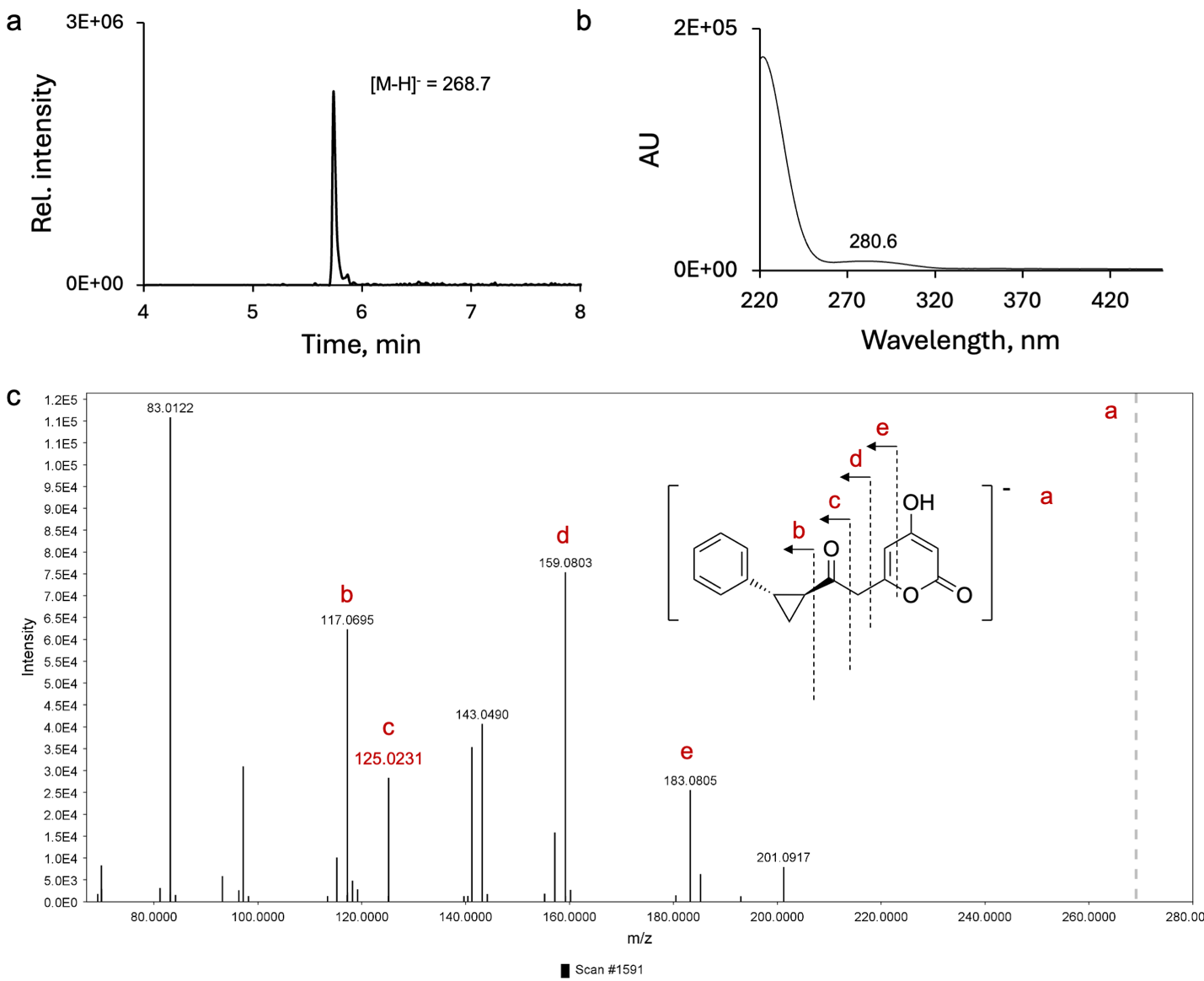
**

**Figure S49.** Spectral data analysis of product **19b**. a) Low-resolution LC-MS analysis of the EtOAc extract of the enzymatic reaction of TtonPKS with **19**; extracted ion chromatogram of the predicted *m/z* of 269 (negative mode). Y-axis shows relative ion intensity. b) Corresponding UV absorption spectrum. c) ESI-HR-MS/MS (negative mode) with ions matching expected fragments of **19b**; observed *m/z* = 269.0821 (theoretical *m/z* = 269.0819, calculated for [C_16_H_13_O_4_]^-^). The precursor ion is indicated with a dashed grey line.

**
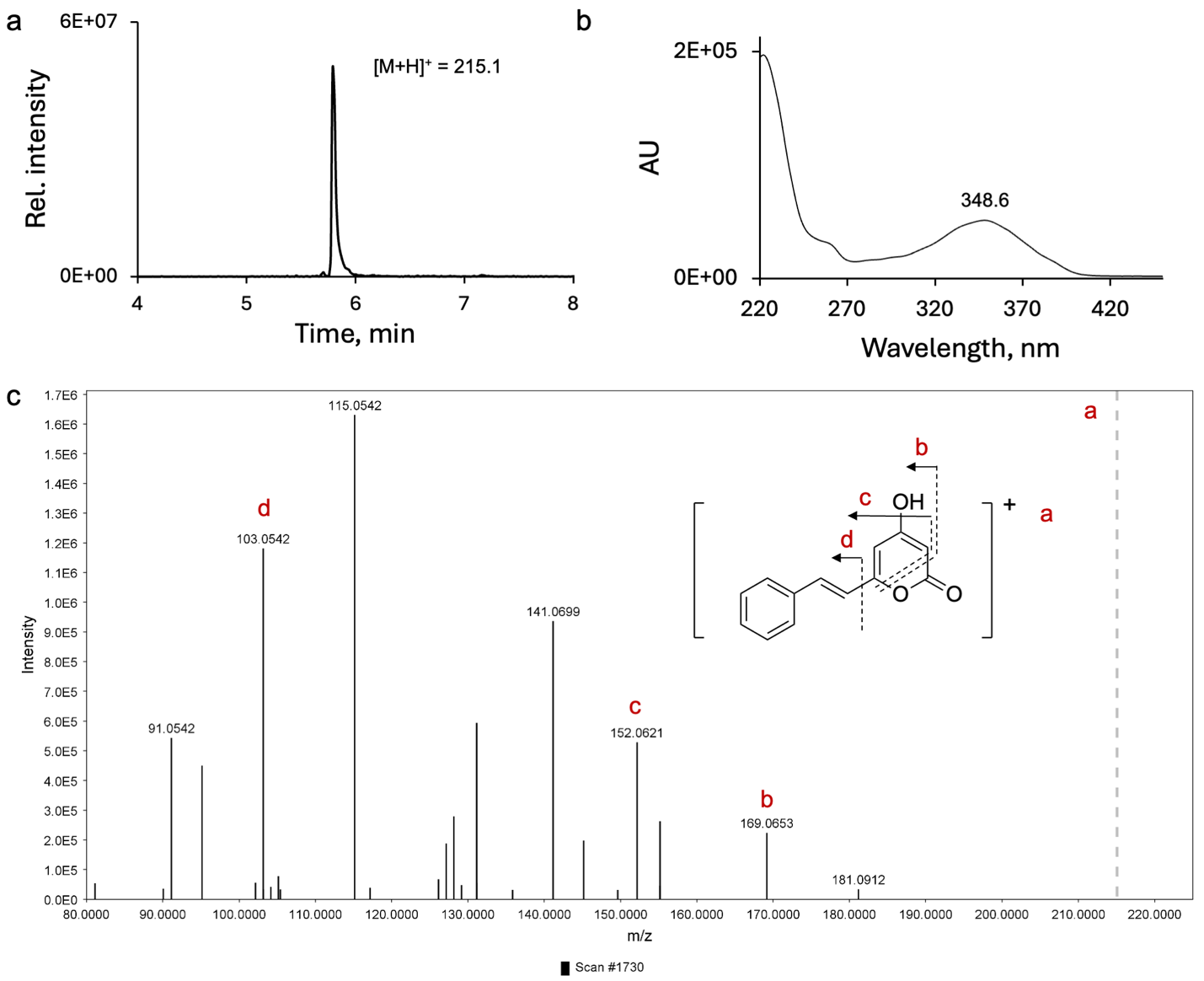
**

**Figure S50.** Spectral data analysis of product **20a**. a) Low-resolution LC-MS analysis of the EtOAc extract of the enzymatic reaction of TtonPKS with **20**; extracted ion chromatogram of the predicted *m/z* of 215 (positive mode). Y-axis shows relative ion intensity. b) Corresponding UV absorption spectrum. c) ESI-HR-MS/MS (negative mode) with ions matching expected fragments of **20a**; observed *m/z* = 215.0701 (theoretical *m/z* = 215.0703, calculated for [C_13_H_11_O_3_]^+^). The precursor ion is indicated with a dashed grey line.

**
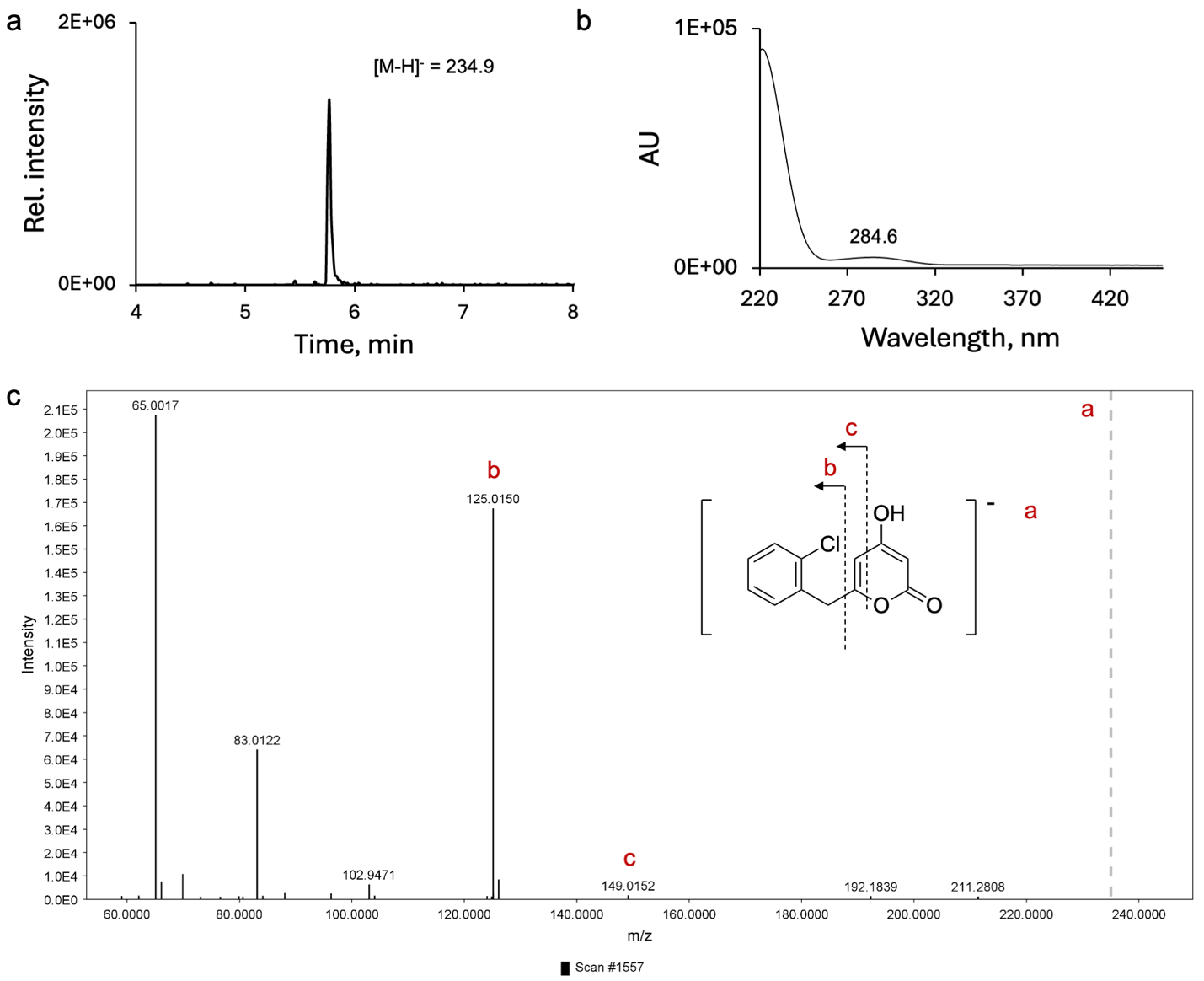
**

**Figure S51.** Spectral data analysis of product **21a**. a) Low-resolution LC-MS analysis of the EtOAc extract of the enzymatic reaction of HargPKS1 with **21**; extracted ion chromatogram of the predicted *m/z* of 235 (negative mode). Y-axis shows relative ion intensity. b) Corresponding UV absorption spectrum. c) ESI-HR-MS/MS (negative mode) with ions matching expected fragments of **21a**; observed *m/z* = 235.0166 (theoretical *m/z* = 235.0167, calculated for [C_12_H_8_ClO_3_]^-^). The precursor ion is indicated with a dashed grey line.

**
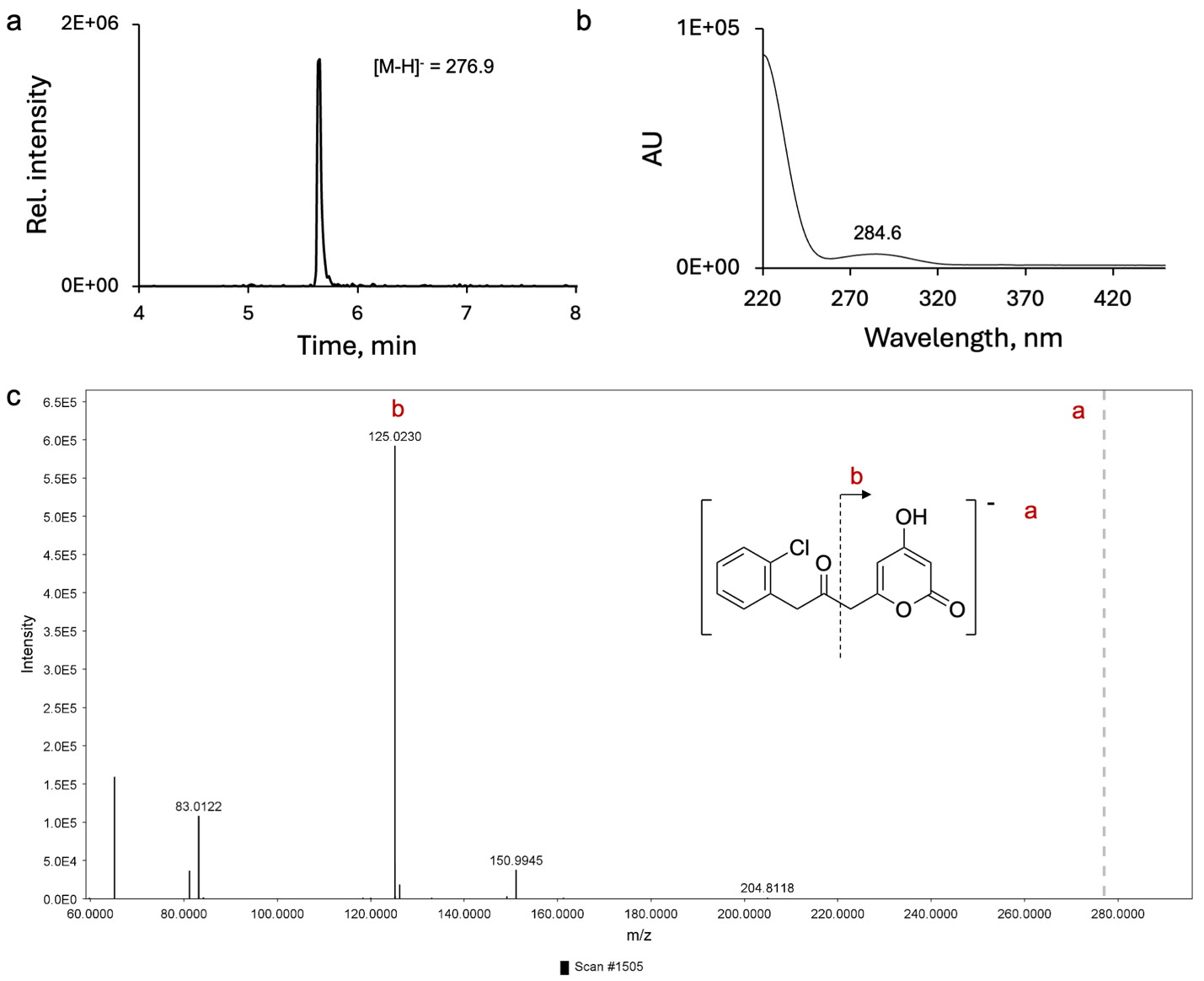
**

**Figure S52.** Spectral data analysis of product **21b**. a) Low-resolution LC-MS analysis of the EtOAc extract of the enzymatic reaction of HargPKS1 with **21**; extracted ion chromatogram of the predicted *m/z* of 277 (negative mode). Y-axis shows relative ion intensity. b) Corresponding UV absorption spectrum. c) ESI-HR-MS/MS (negative mode) with ions matching expected fragments of **21b**; observed *m/z* = 277.0274 (theoretical *m/z* = 277.0273, calculated for [C_14_H_10_ClO_4_]^-^). The precursor ion is indicated with a dashed grey line.

**
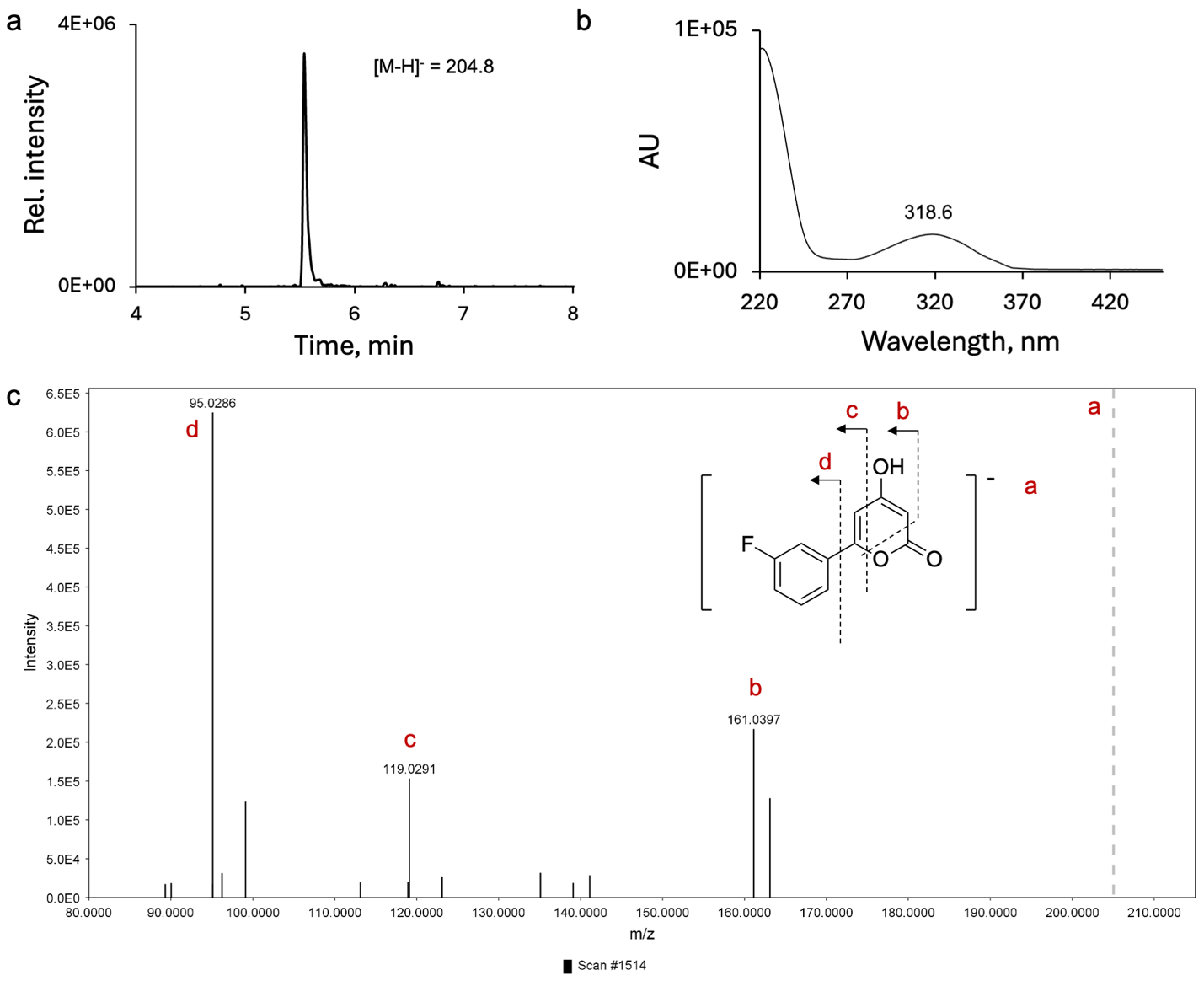
**

**Figure S53.** Spectral data analysis of product **22a**. a) Low-resolution LC-MS analysis of the EtOAc extract of the enzymatic reaction of TtonPKS with **22**; extracted ion chromatogram of the predicted *m/z* of 205 (negative mode). Y-axis shows relative ion intensity. b) Corresponding UV absorption spectrum. c) ESI-HR-MS/MS (negative mode) with ions matching expected fragments of **22a**; observed *m/z* = 205.0299 (theoretical *m/z* = 205.0306, calculated for [C_11_H_6_FO_3_]^-^). The precursor ion is indicated with a dashed grey line.

**
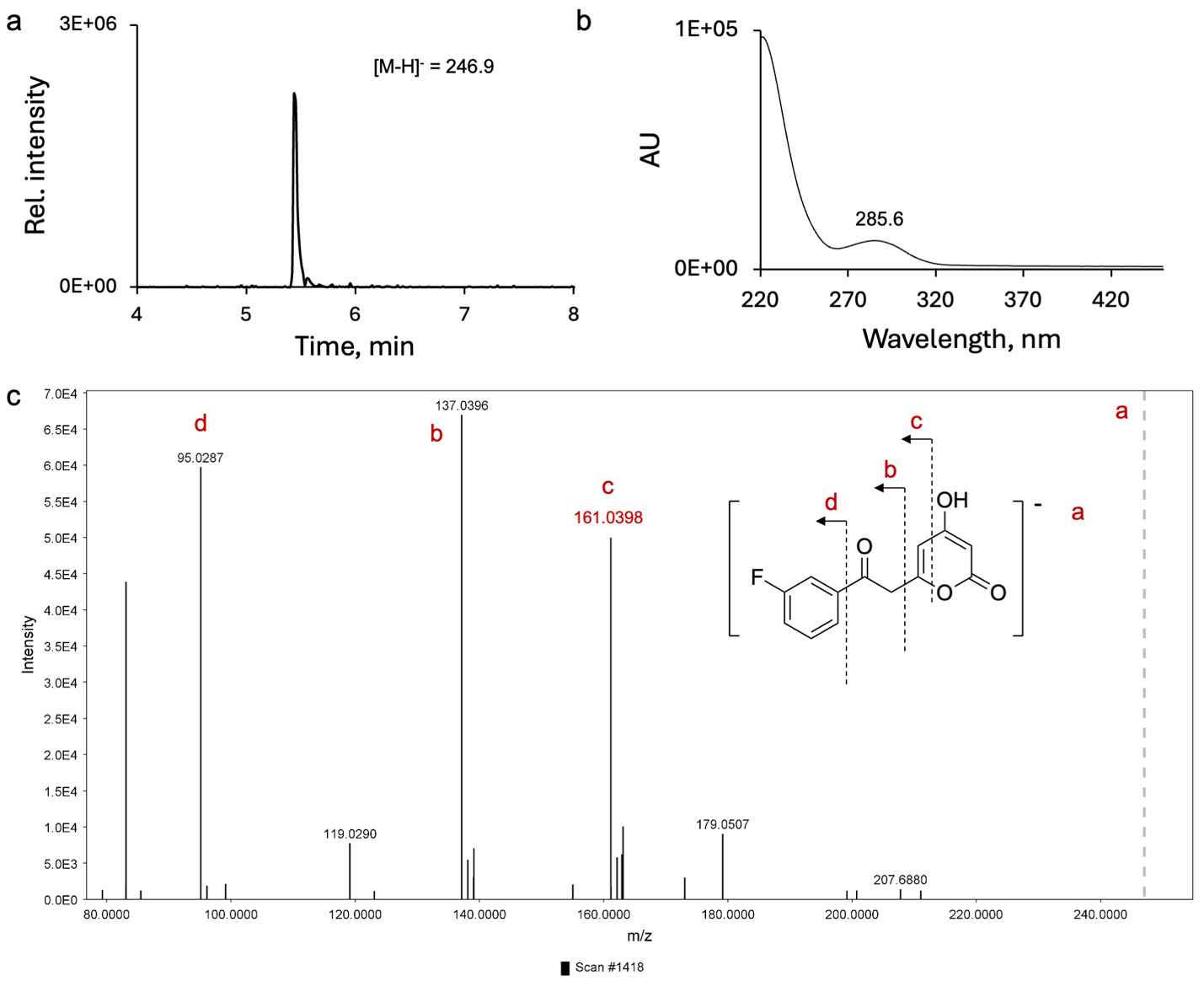
**

**Figure S54.** Spectral data analysis of product **22b**. a) Low-resolution LC-MS analysis of the EtOAc extract of the enzymatic reaction of TtonPKS with **22**; extracted ion chromatogram of the predicted *m/z* of 247 (negative mode). Y-axis shows relative ion intensity. b) Corresponding UV absorption spectrum. c) ESI-HR-MS/MS (negative mode) with ions matching expected fragments of **22b**; observed *m/z* = 247.0412 (theoretical *m/z* = 247.0412, calculated for [C_13_H_8_FO_4_]^-^). The precursor ion is indicated with a dashed grey line.

**
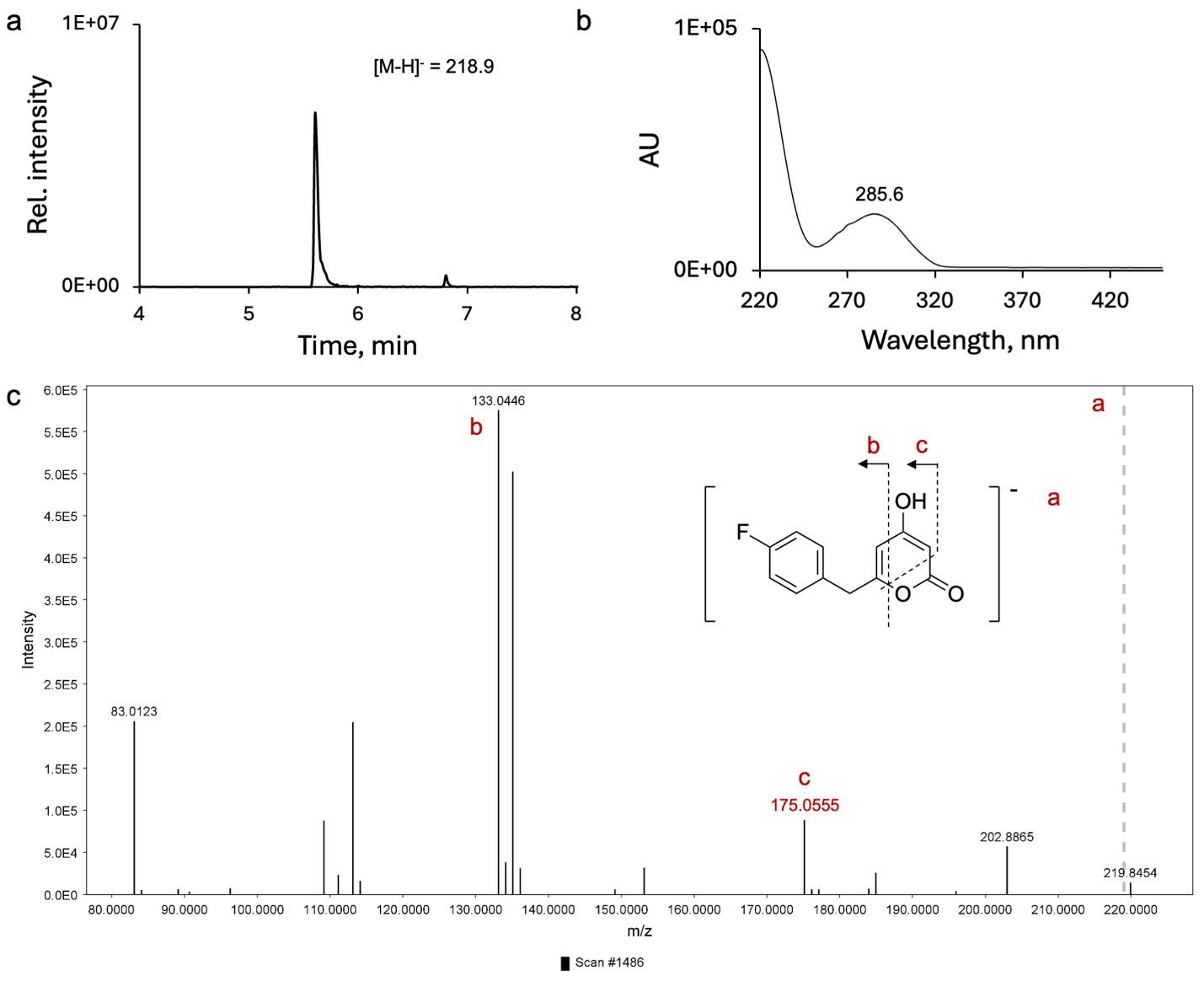
**

**Figure S55.** Spectral data analysis of product **23a**. a) Low-resolution LC-MS analysis of the EtOAc extract of the enzymatic reaction of AiizPKS with **23**; extracted ion chromatogram of the predicted *m/z* of 219 (negative mode). Y-axis shows relative ion intensity. b) Corresponding UV absorption spectrum. c) ESI-HR-MS/MS (negative mode) with ions matching expected fragments of **23a**; observed *m/z* = 219.0458 (theoretical *m/z* = 219.0463, calculated for [C_12_H_8_FO_3_]^-^). The precursor ion is indicated with a dashed grey line.

**
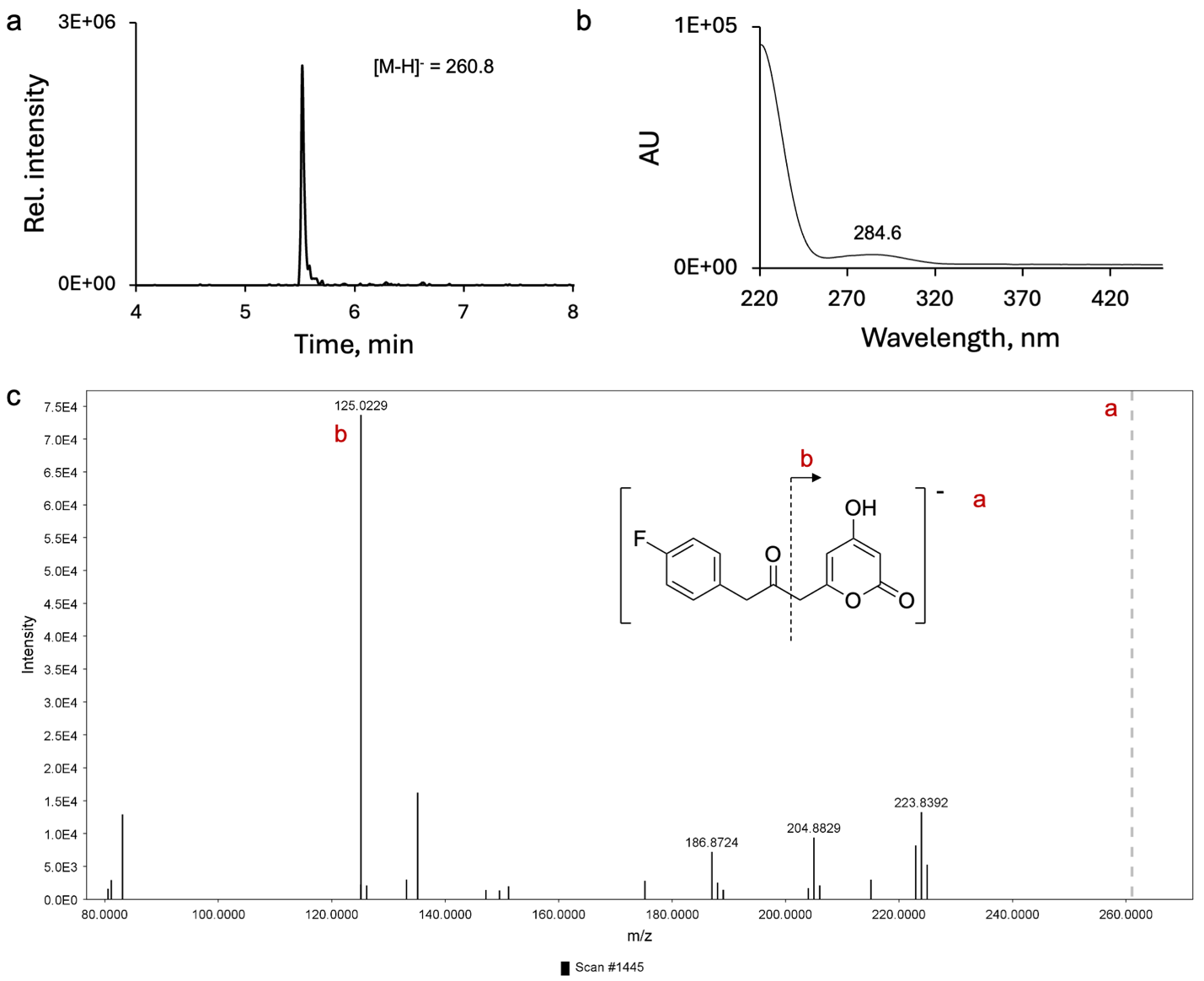
**

**Figure S56.** Spectral data analysis of product **23b**. a) Low-resolution LC-MS analysis of the EtOAc extract of the enzymatic reaction of AiizPKS with **23**; extracted ion chromatogram of the predicted *m/z* of 261 (negative mode). Y-axis shows relative ion intensity. b) Corresponding UV absorption spectrum. c) ESI-HR-MS/MS (negative mode) with ions matching expected fragments of **23b**; observed *m/z* = 261.0569 (theoretical *m/z* = 261.0569, calculated for [C_14_H_10_FO_4_]^-^). The precursor ion is indicated with a dashed grey line.

**
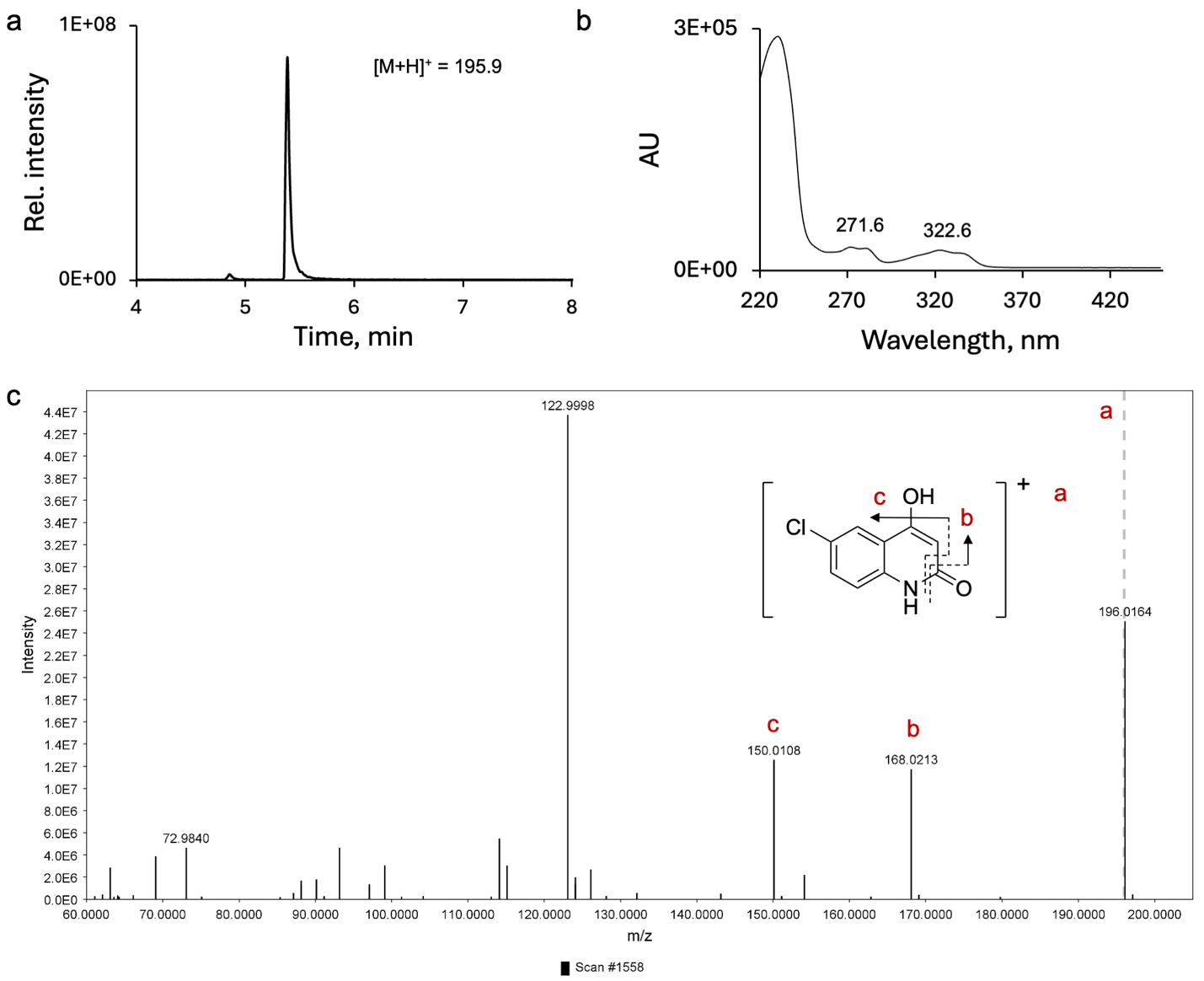
**

**Figure S57.** Spectral data analysis of product **24a**. a) Low-resolution LC-MS analysis of the EtOAc extract of the enzymatic reaction of AiizPKS with **24**; extracted ion chromatogram of the predicted *m/z* of 196 (positive mode). Y-axis shows relative ion intensity. b) Corresponding UV absorption spectrum. c) ESI-HR-MS/MS (negative mode) with ions matching expected fragments of **24a**; observed *m/z* = 196.0164 (theoretical *m/z* = 196.0160, calculated for [C_9_H_7_ClNO_2_]^+^). The precursor ion is indicated with a dashed grey line.

**
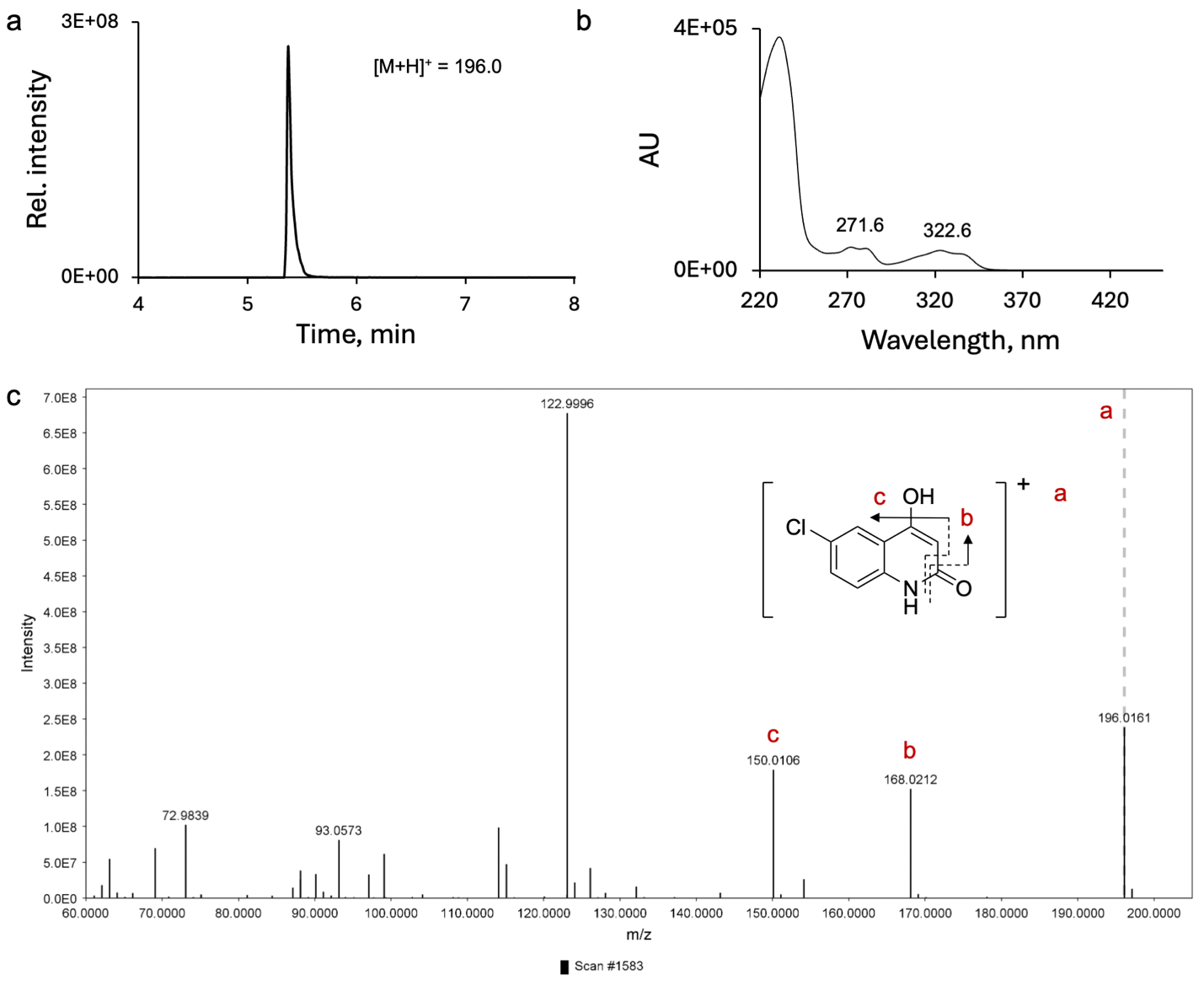
**

**Figure S58.** Spectral data analysis of the authentic standard of compound **24a**. a) Low-resolution LC-MS analysis of **24a**; extracted ion chromatogram of the predicted *m/z* of 196 (positive mode). Y-axis shows relative ion intensity. b) Corresponding UV absorption spectrum. c) ESI-HR-MS/MS (negative mode) with ions matching expected fragments of **24a**; observed *m/z* = 196.0161 (theoretical *m/z* = 196.0160, calculated for [C_9_H_7_ClNO_2_]^+^). The precursor ion is indicated with a dashed grey line.

**
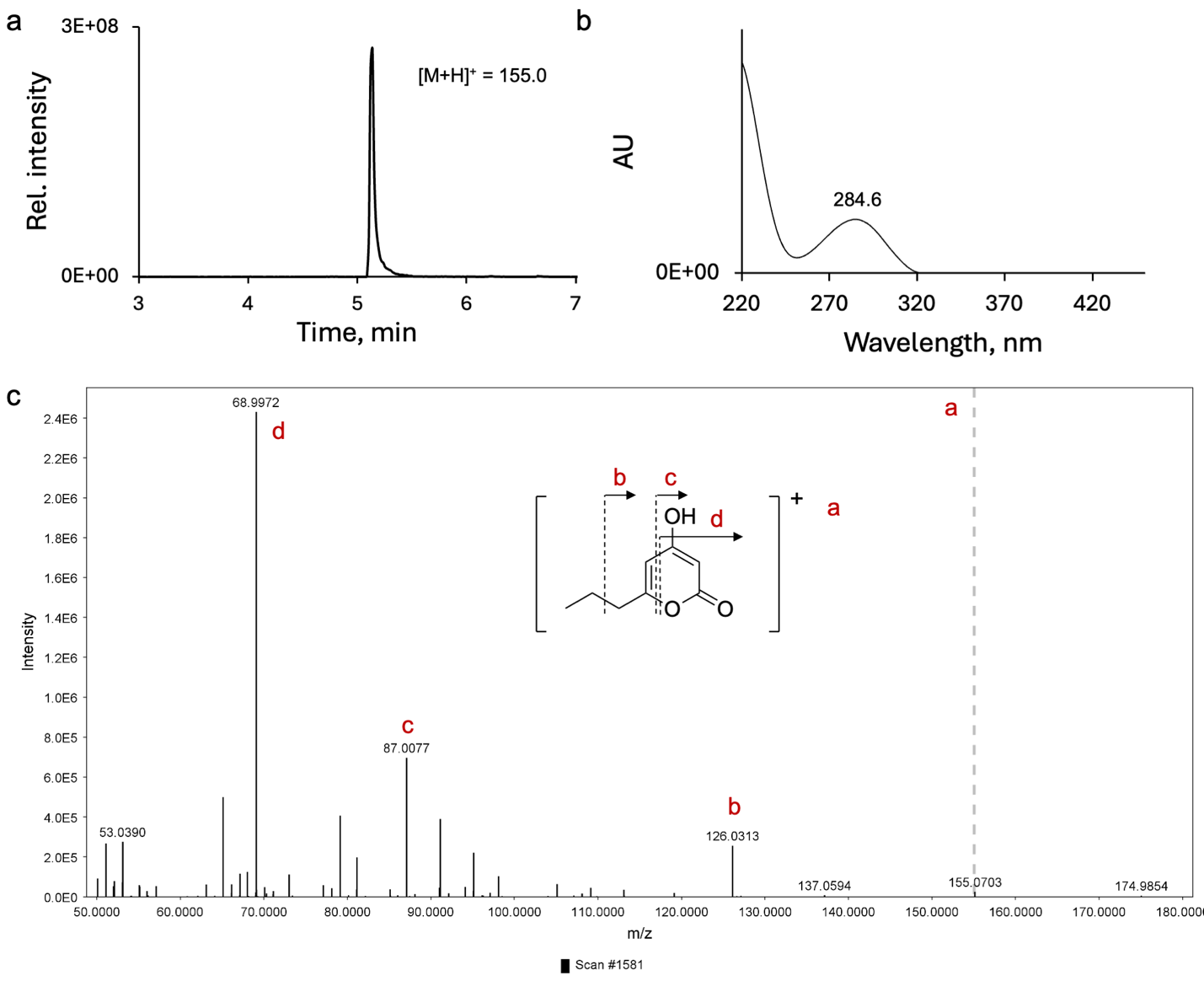
**

**Figure S59.** Spectral data analysis of product **27a**. a) Low-resolution LC-MS analysis of the EtOAc extract of the enzymatic reaction of AgalPKS with **27**; extracted ion chromatogram of the predicted *m/z* of 155 (positive mode). Y-axis shows relative ion intensity. b) Corresponding UV absorption spectrum. c) ESI-HR-MS/MS (negative mode) with ions matching expected fragments of **27a**; observed *m/z* = 155.0702 (theoretical *m/z* = 155.0703, calculated for [C_8_H_11_O_3_]^+^). The precursor ion is indicated with a dashed grey line.

**
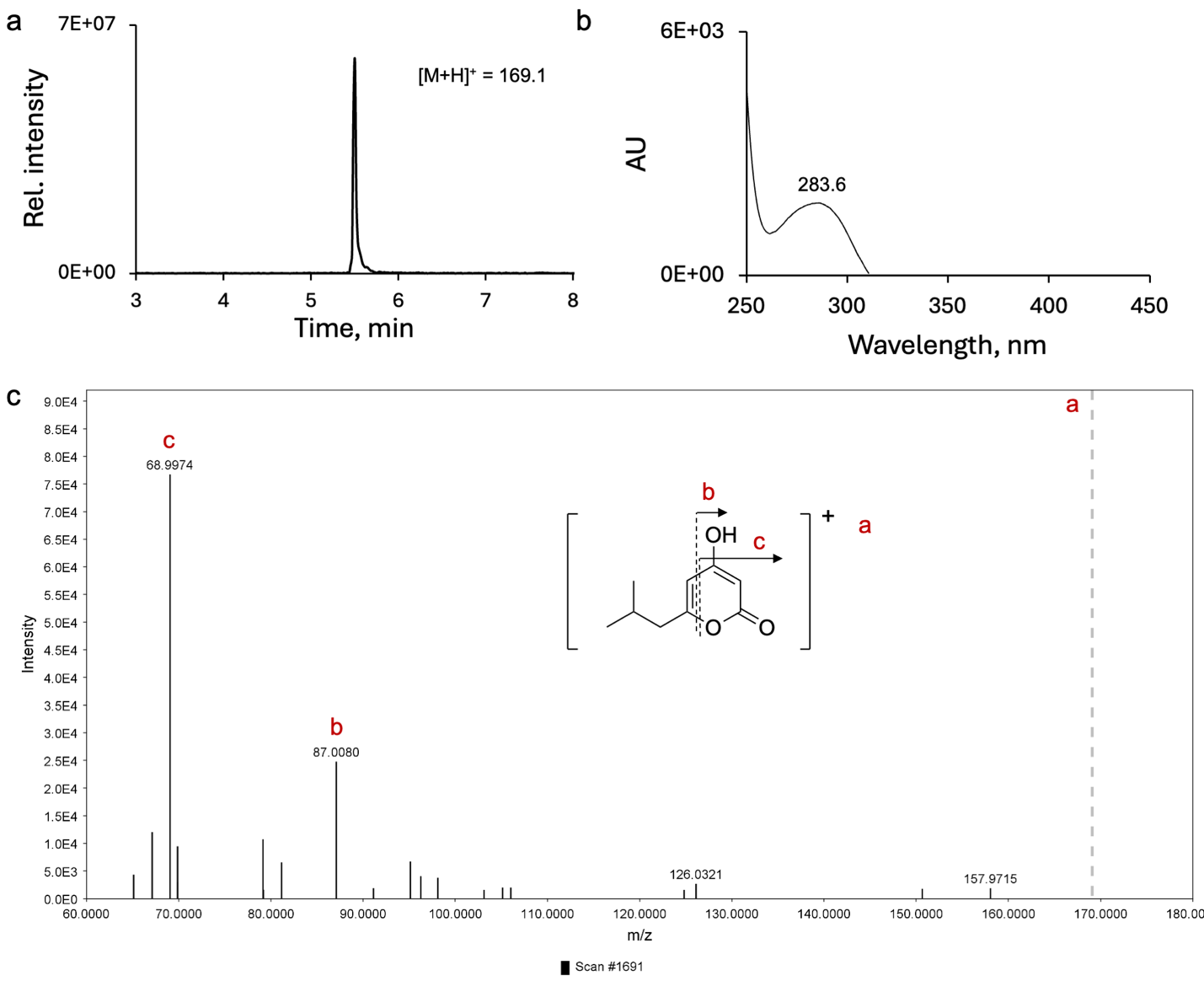
**

**Figure S60.** Spectral data analysis of product **31a**. a) Low-resolution LC-MS analysis of the EtOAc extract of the enzymatic reaction of PansPKS with **31**; extracted ion chromatogram of the predicted *m/z* of 169 (positive mode). Y-axis shows relative ion intensity. b) Corresponding UV absorption spectrum. c) ESI-HR-MS/MS (negative mode) with ions matching expected fragments of **31a**; observed *m/z* = 169.0860 (theoretical *m/z* = 169.0859, calculated for [C_9_H_13_O_3_]^+^). The precursor ion is indicated with a dashed grey line.


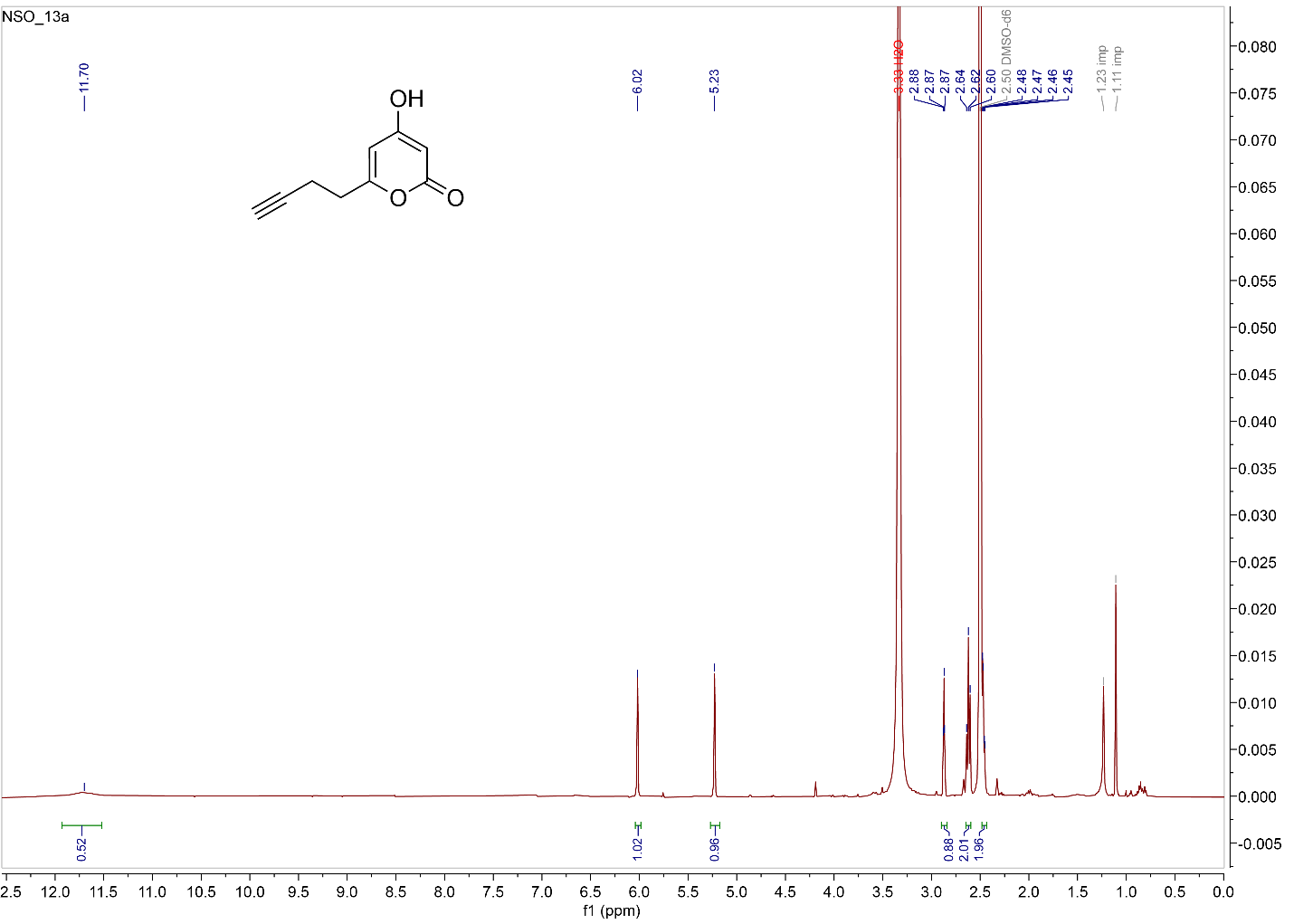


#### **Figure S61.** ^1^H NMR spectrum of compound **13a**.


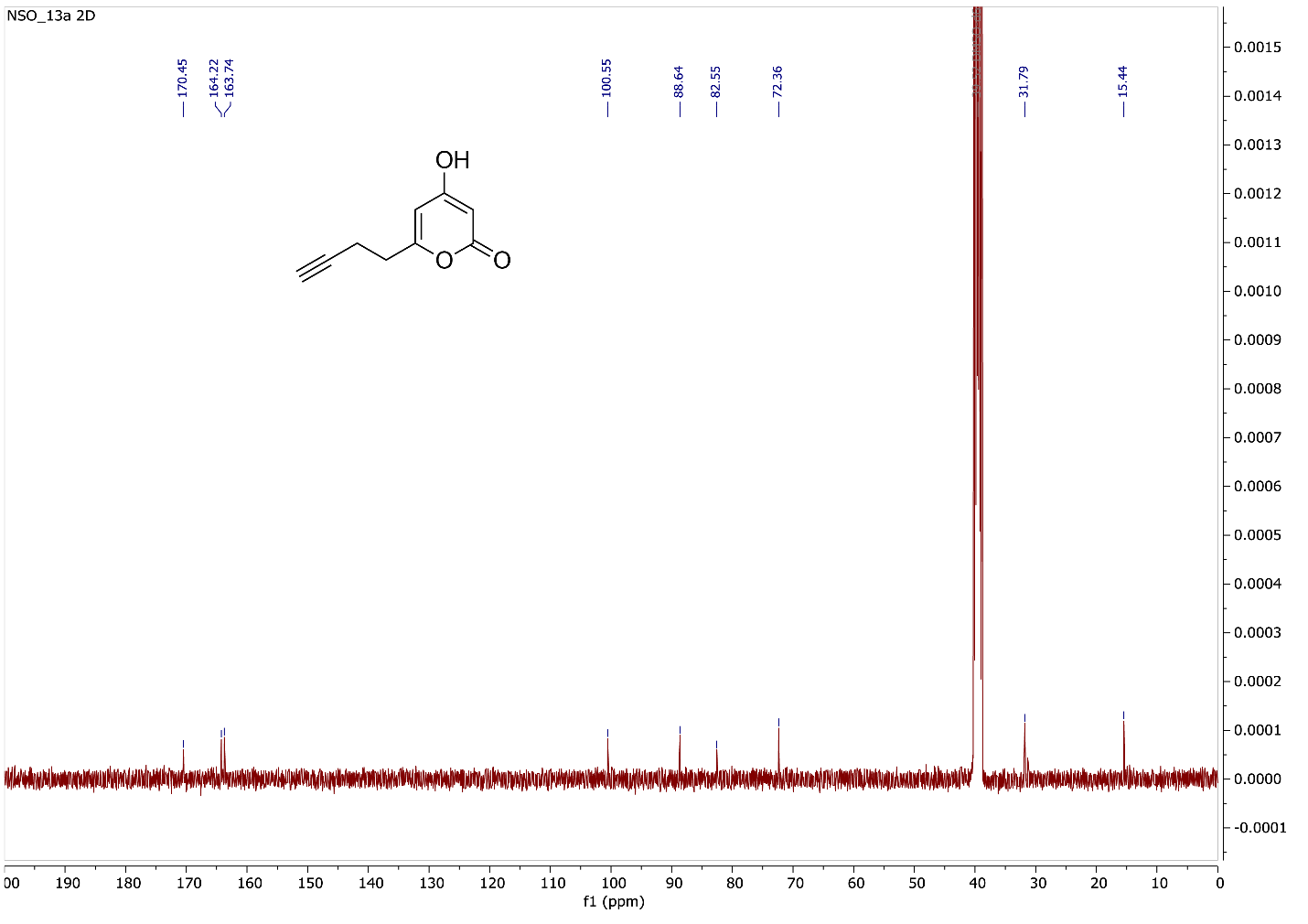


#### **Figure S62.** ^13^C NMR spectrum of compound **13a**.


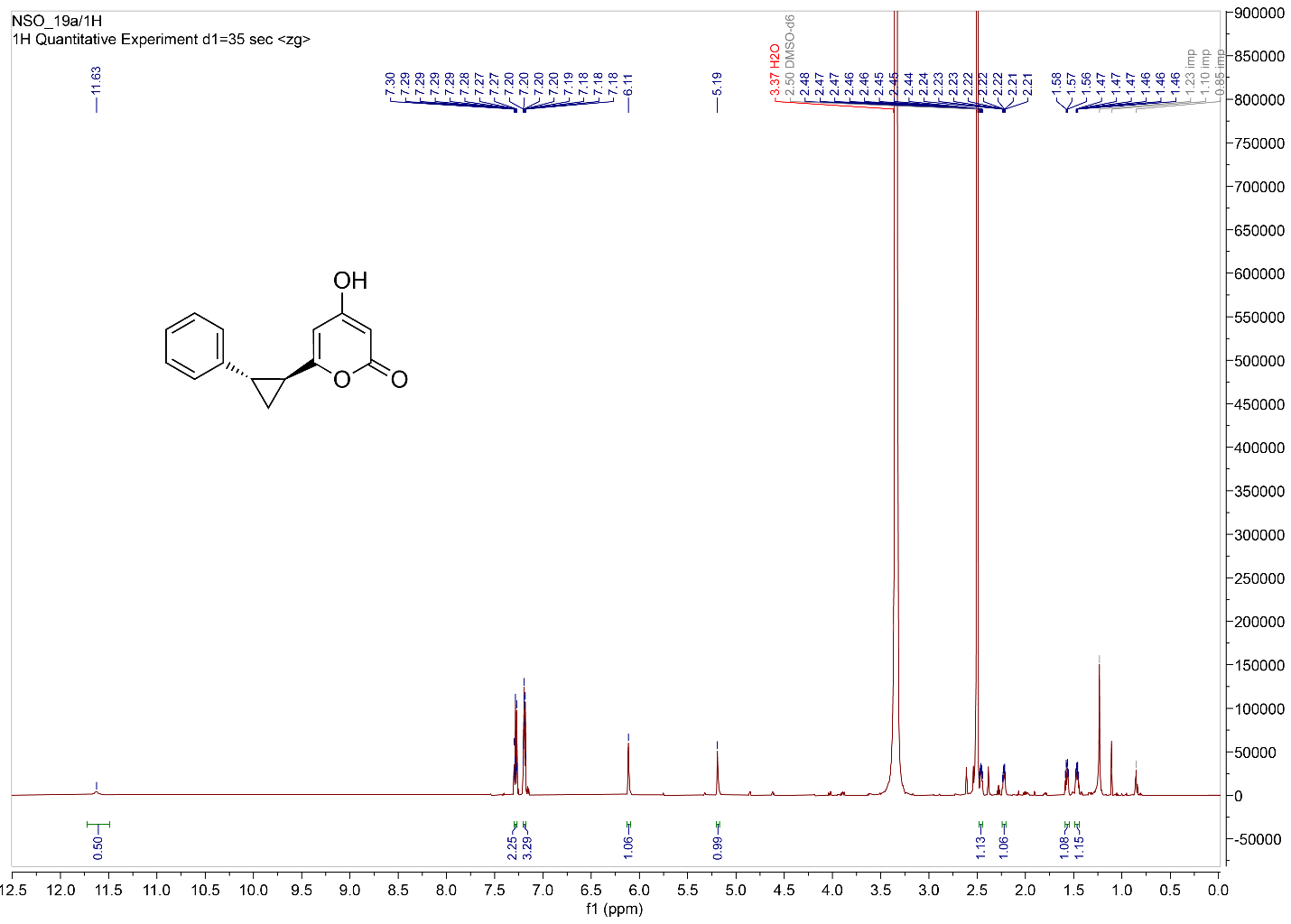


#### **Figure S63.** ^1^H NMR spectrum of compound **19a**.


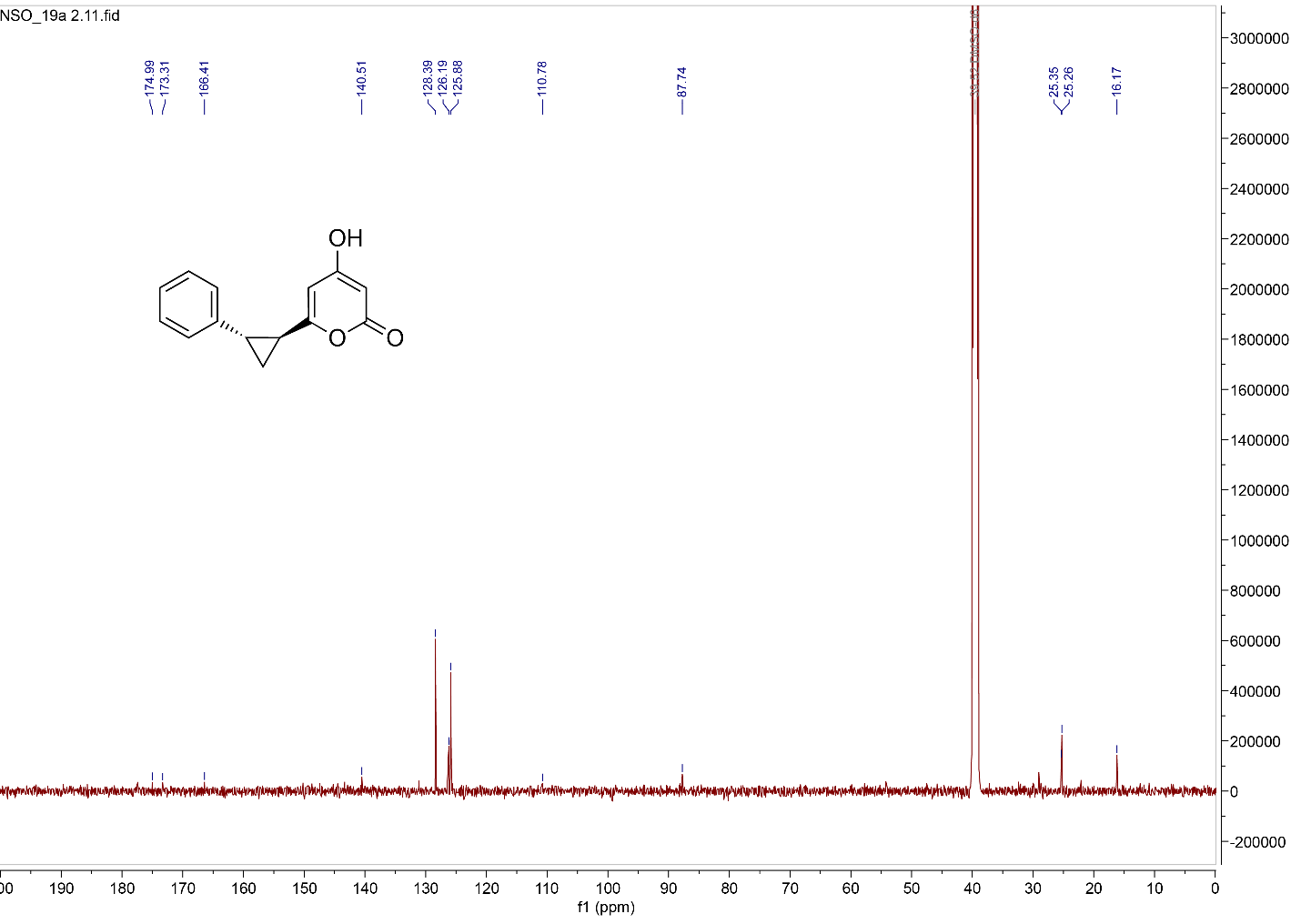


**Figure S64**. ^13^C NMR spectrum of compound **19a**.


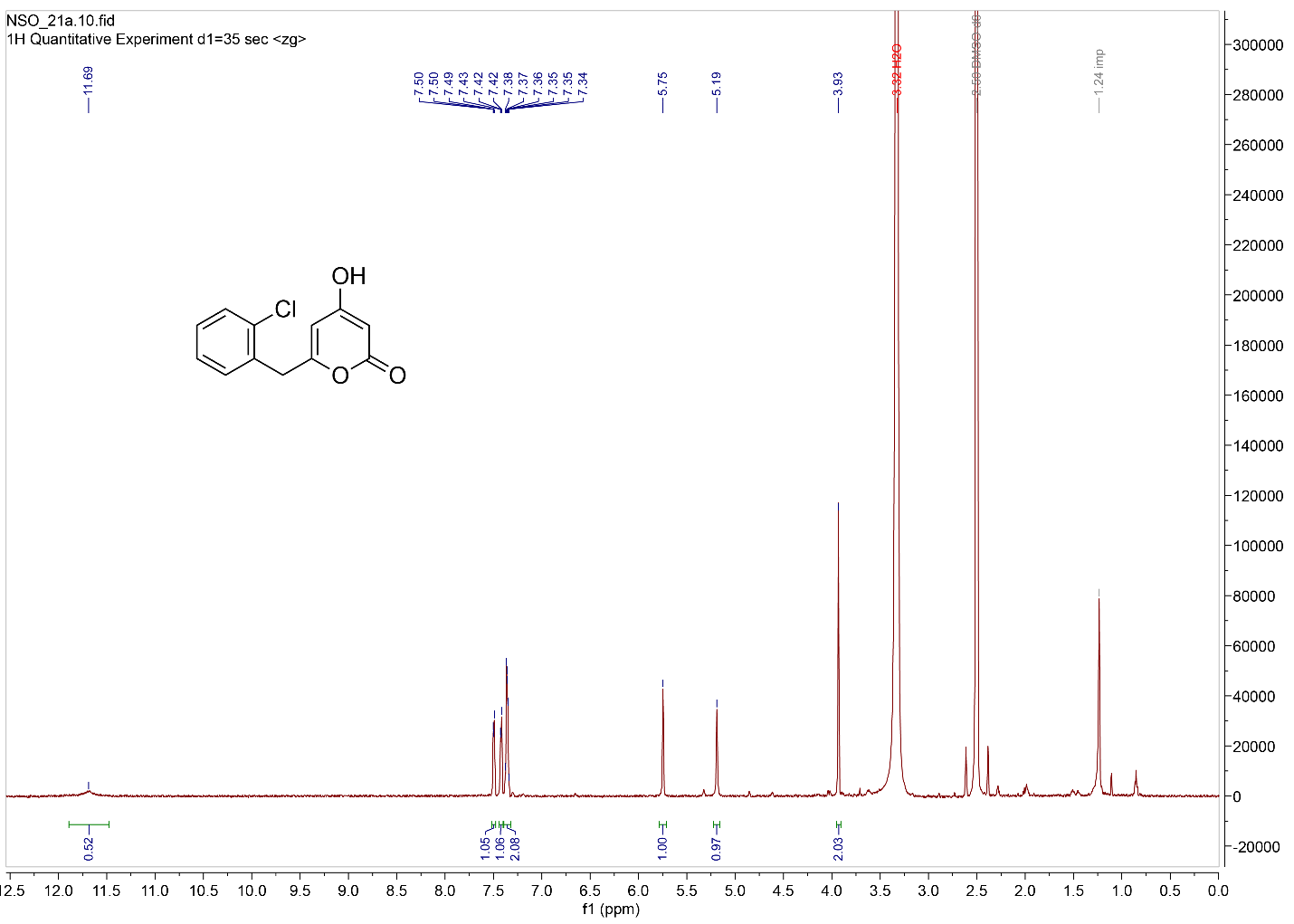


#### **Figure S65.** ^1^H NMR spectrum of compound **21a**.


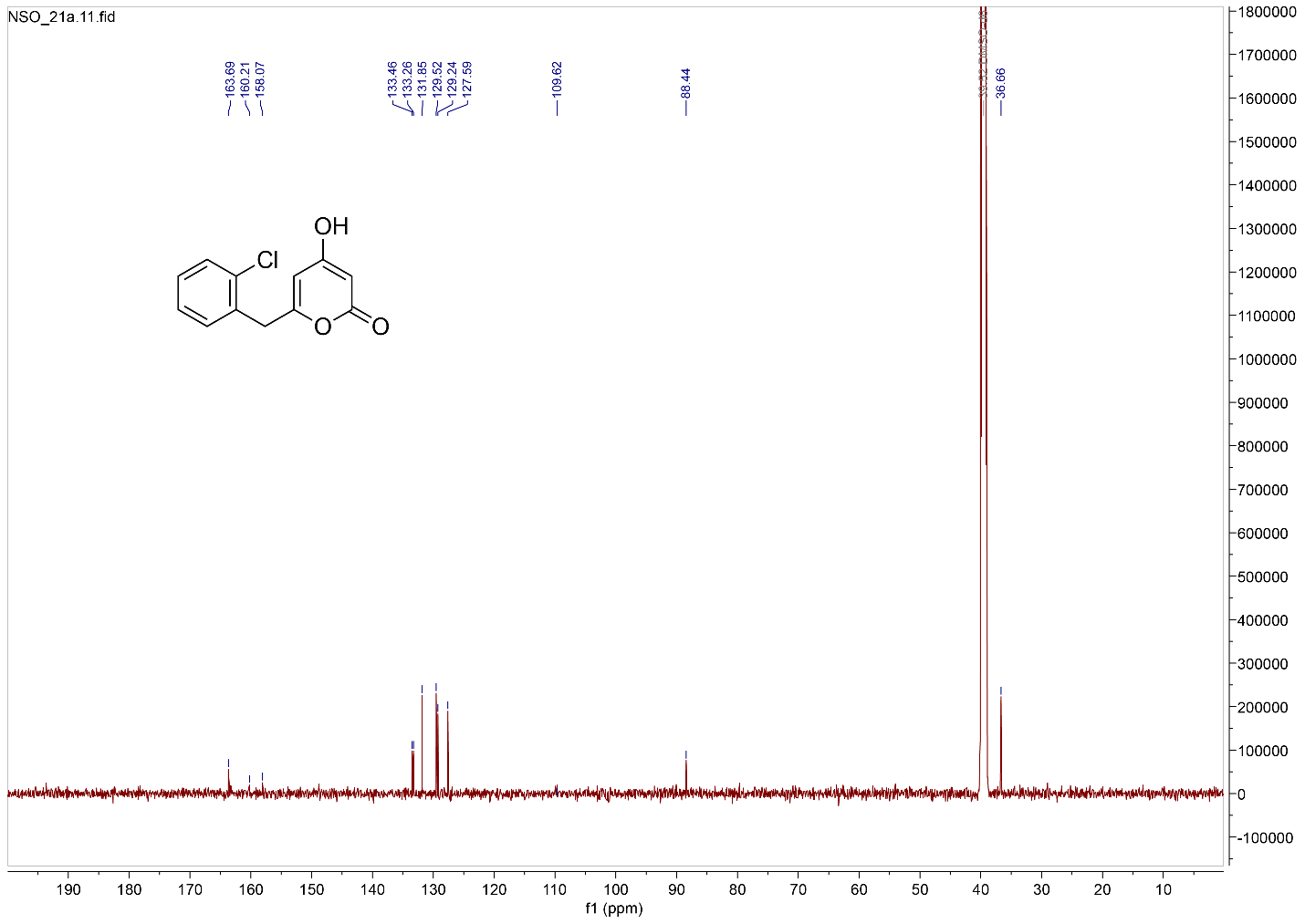


#### **Figure S66.** ^13^C NMR spectrum of compound **21a**.


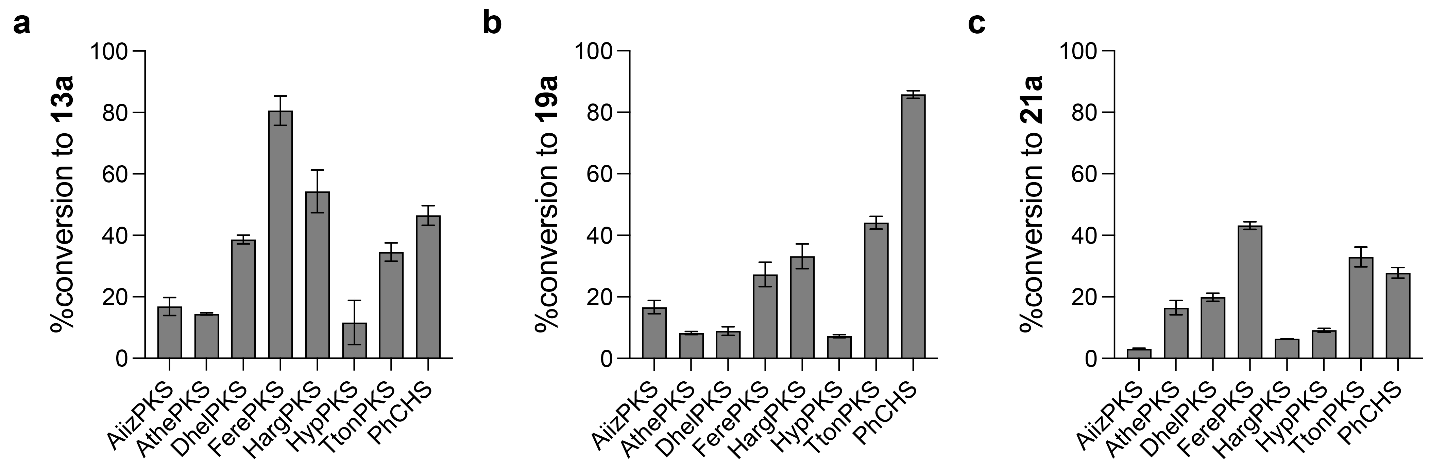


**Figure S67.** Conversion of substrates **13**, **19** and **21** by purified fungal T3PKSs and PhCHS. a) conversion to product **13a**; b) conversion to product **19a**; a) conversion to product **21a**. Reaction mixture: 0.5 mM substrate, 1 mM malonyl-CoA and 25 µM purified enzyme in 100 mM HEPES/NaOH pH 8; total reaction volume: 25 µL; incubation at 37 °C for 16 h.


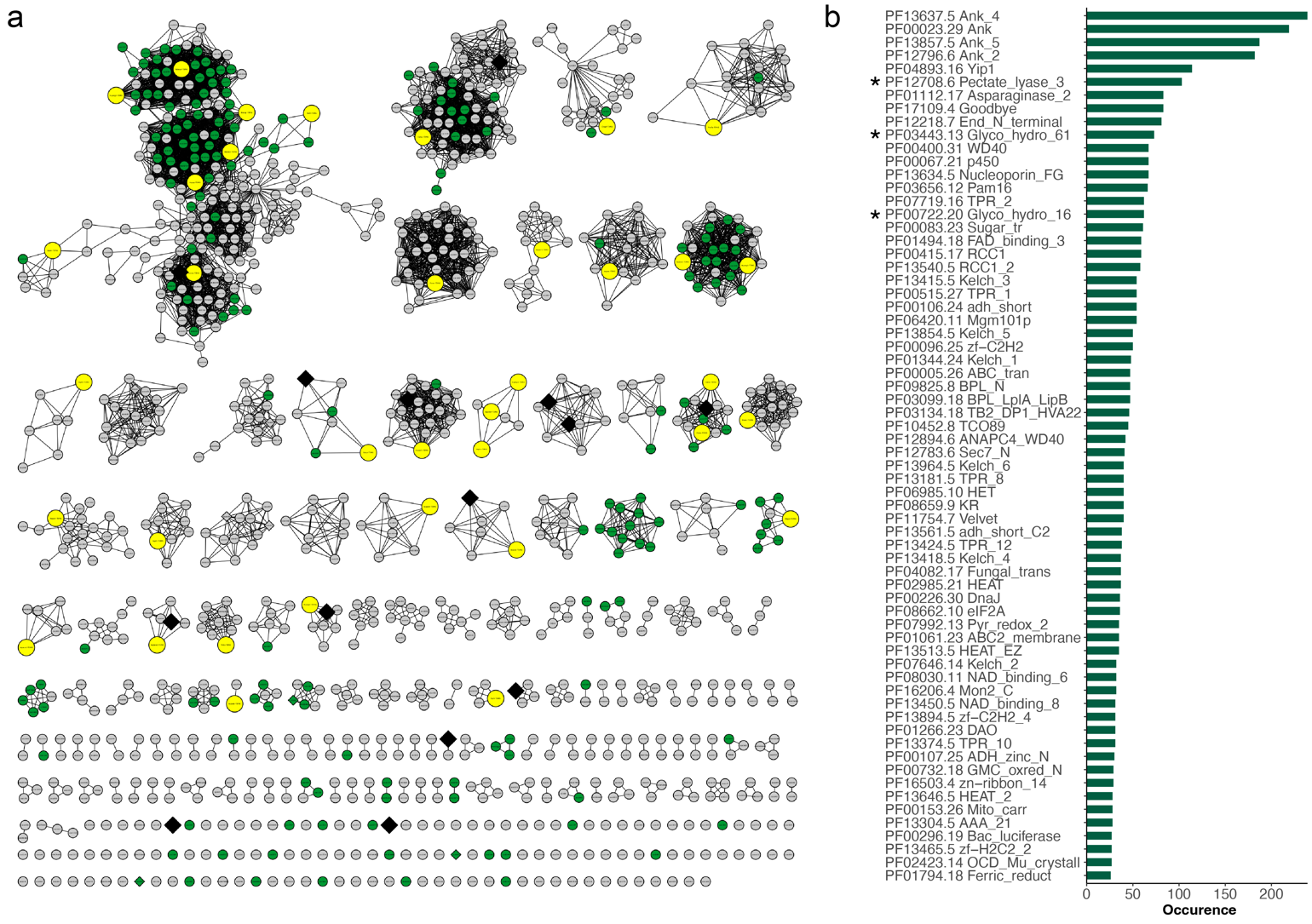


**Figure S68.** T3PKSs originating from endophytic or phytopathogenic fungi. a) Sequence similarity network of the fungal T3PKSs with 127 sequences from endophytic or phytopathogenic fungi represented as green nodes. The sequences selected for characterisation in this study are represented as yellow nodes. b) The most abundant PFAM domains in the neighbourhood of these PKSs; putative carbohydrate-active enzymes (CAZymes) are marked with an asterisk.


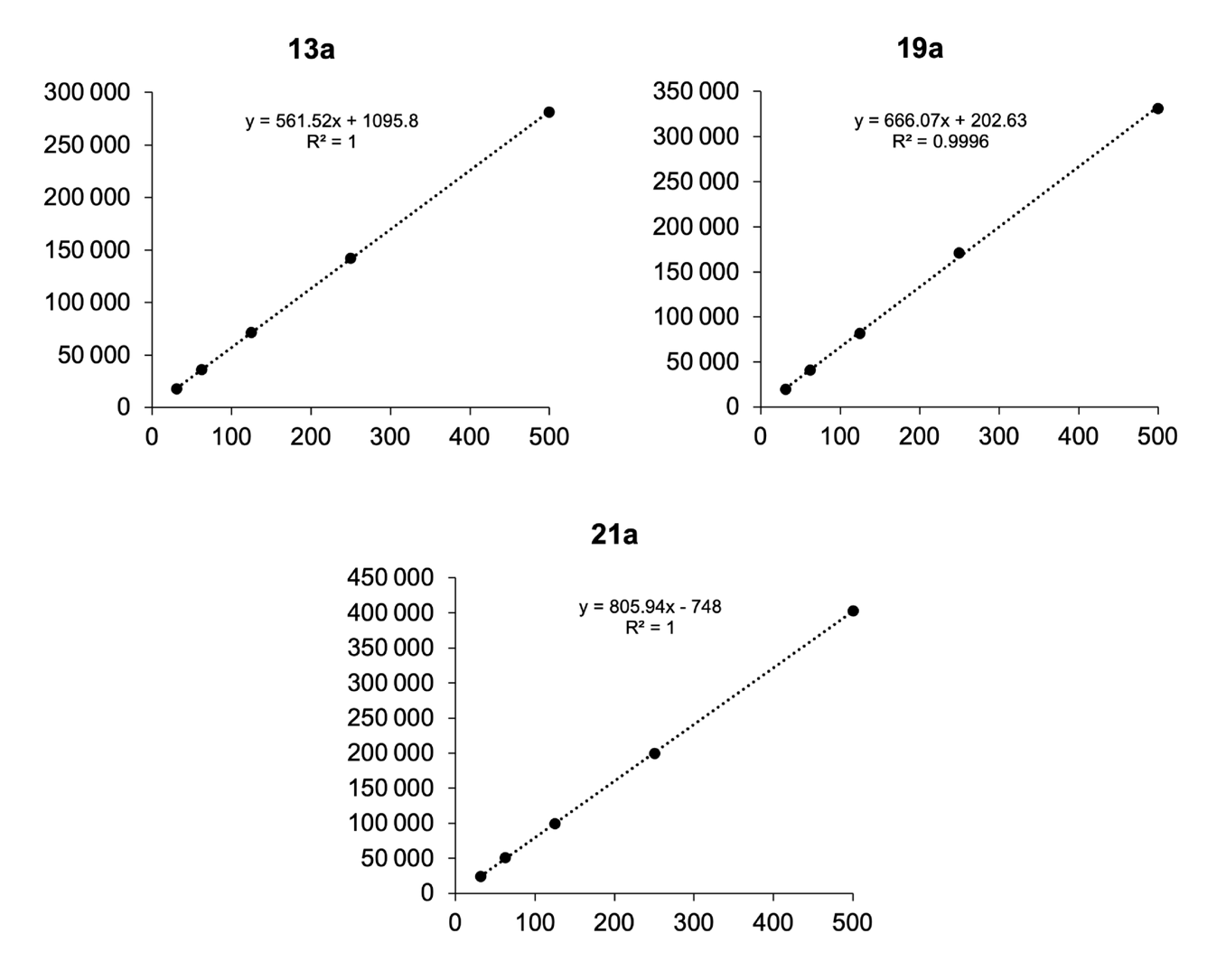


**Figure S69.** Calibration plots for products **13a**, **19a** and **21a.** The plots were used to calculate the conversion of substrates 13, 19 and 21 by fungal T3PKSs and PhCHS.

## Supporting results

### Machine learning proposes amino acid residues important for substrate specificity

While our model makes accurate predictions of activity for new enzymes and substrates, it does not provide insight into the decision-making process. Therefore, we employed a descriptive machine learning approach to pinpoint the residues that might be important for substrate specificity (Figure S70a). Instead of featurising the whole protein sequence, we retrieved active site residues based on the naringenin-bound structure of MsCHS (PDB: 1CGK), as well as those lining the fatty acid-binding tunnel according to the structure of ORAS (PDB: 3UET). We excluded fully conserved residues from this analysis. For all 31 active fungal PKSs, we featurised these 41 amino acids and used them together with the activity data for the ML model training. For this purpose, the Decision Tree approach was used as a simplistic model that is easier to interpret compared to more sophisticated algorithms.


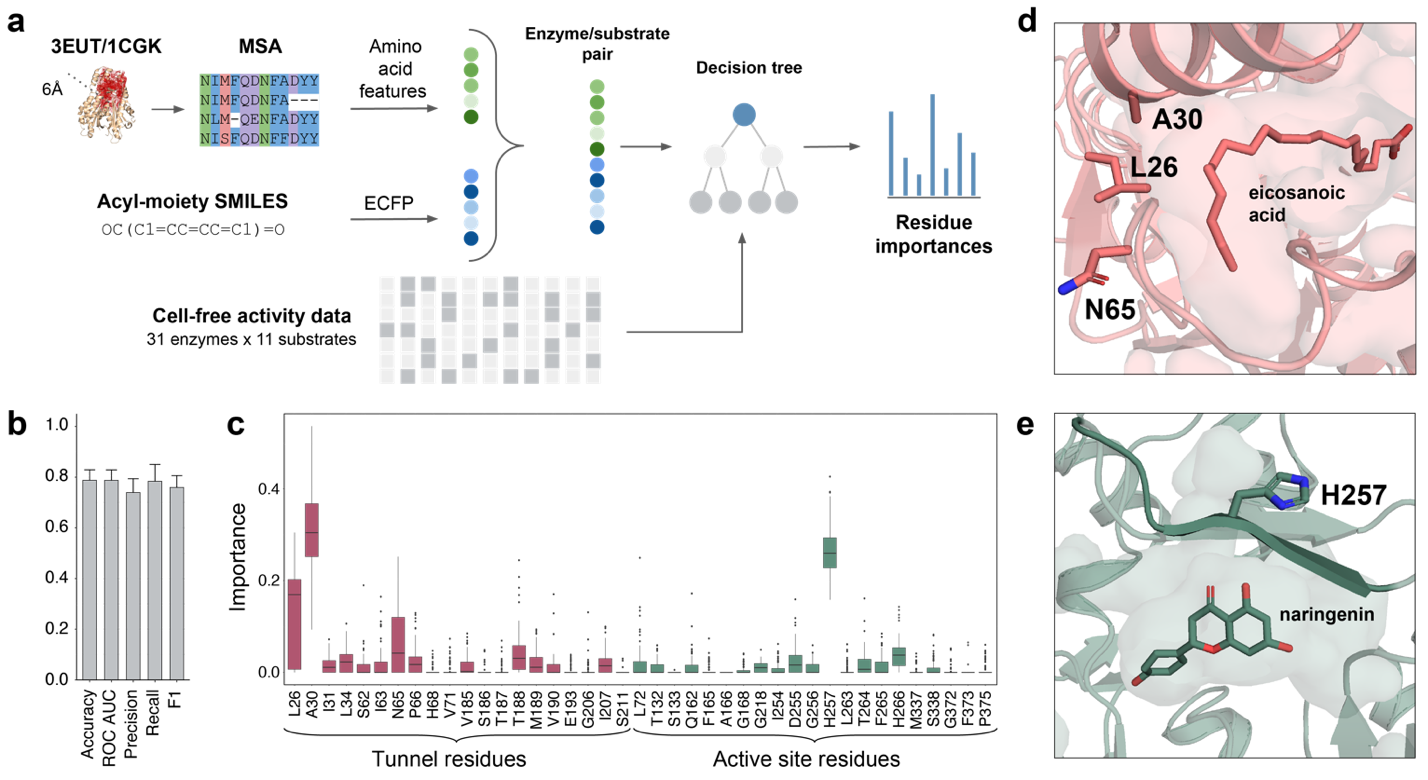


**Figure S70.** Descriptive machine learning model. a) Workflow used to train the descriptive machine learning model in this study. MSA - multiple sequence alignment; ECFP - extended-connectivity fingerprints. 3EUT – crystal structure of ORAS complexed with eicosanoic acid^[70]^; 1CGK – crystal structure of MsCHS complexed with naringenin^[71]^. b) Performance metrics of the Decision Tree model trained on the cell-free activity profiling dataset with per-residue molecular descriptor and MACCS Keys feature vectors; error bars indicate standard deviation. c) Importances of the active site and substrate tunnel residues represented as boxplots (n= 100). Numbering of the tunnel residues is taken from ORAS; numbering of the active site residues is taken from MsCHS. Cartoon representation of d) the substrate tunnel in ORAS and e) active site in MsCHS with the three most important residues shown as sticks (blue: nitrogen atoms, red: oxygen atoms).

The resulting model achieved 79.4 ± 4.0% accuracy and 0.76 ± 0.045 F1 score (Figure S70b). The analysis of feature importances in the model showed that tunnel residues L26, A30 and N65, as well as the active site residue H257, were the most important for classification accuracy (Figure S70c-e). L26, A30 and N65 are the residues capping the surface end of the substrate tunnel in ORAS. The role of these residues is currently understudied, but it is conceivable that they are important for the orientation of longer-chain fatty acyl substrates. Interestingly, position 26 is mostly occupied by the hydrophobic amino acids leucine, isoleucine, valine and methionine except in two enzymes, XylPKS (cysteine) and AtamPKS2 (arginine). XylPKS was only active on two substrates (**7** and **10**), whereas AtamPKS2 was inactive. This may indicate that sequence variation in this position leads to a stricter substrate specificity, possibly outside the scope of the tested substrates. The sequence diversity at positions 30 and 65 is quite high across the selected T3PKS and we were not able to rationalise their individual contributions to the substrate specificities.

While the active site residue H257 is too distant to directly interact with the substrate, it likely participates in salt bridge formation between the T3PKS monomers^[72]^. Its mutation to lysine was shown to contribute to a shift in the cyclisation specificity of MsCHS towards a resorcinol product^[73]^ but there are no literature reports on its role in substrate specificity. Within our set of fungal T3PKS, this position is occupied by a negatively charged glutamate residue in the enzymes stemming from Sordariomycetes, as well as FerePKS, PtriPKS, and the inactive AtamPKS2 (Figure S71). Functionally, these enzymes appear to prefer aliphatic starter units (**7**-**10**) and substrate **3**, although CgloPKS and FerePKS can also accept substrate **1**.

Overall, the descriptive ML model does not allow the immediate rationalisation of the individual contributions of the selected amino acid positions to substrate selection, since there might also be synergistic effects of these positions at play. It may, however, provide an interesting starting point for future enzyme engineering campaigns.


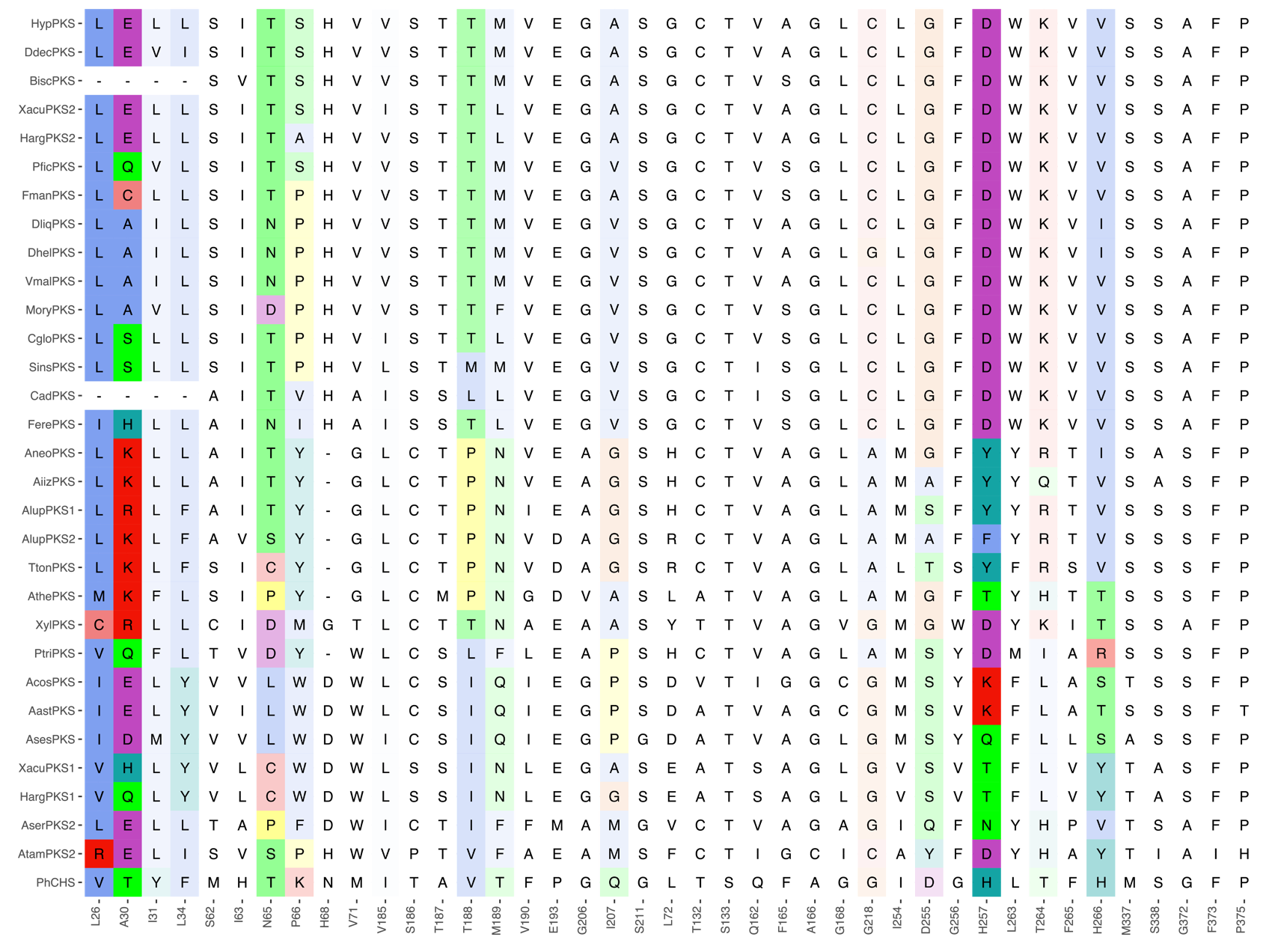


**Figure S71.** Multiple sequence alignment of the amino acids lining active site and substrate-binding tunnel in the 31 expressed T3PKSs. The residues are highlighted using Clustal X Default Colouring; colour intensity is scaled according to residue importances as determined by the descriptive ML model.

## Supporting References

[1] T. Nakamura, K. D. Yamada, K. Tomii, K. Katoh, “Parallelization of MAFFT for large-scale multiple sequence alignments” *Bioinformatics* **2018**, *34*, 2490–2492.

[2] M. Steinegger, J. Söding, “MMseqs2 enables sensitive protein sequence searching for the analysis of massive data sets” *Nat Biotechnol* **2017**, *35*, 1026–1028.

[3] I. V. Grigoriev, R. Nikitin, S. Haridas, A. Kuo, R. Ohm, R. Otillar, R. Riley, A. Salamov, X. Zhao, F. Korzeniewski, T. Smirnova, H. Nordberg, I. Dubchak, I. Shabalov, “MycoCosm portal: Gearing up for 1000 fungal genomes” *Nucleic Acids Research* **2014**, *42*, D699–D704.

[4] T. Paysan-Lafosse, M. Blum, S. Chuguransky, T. Grego, B. L. Pinto, G. A. Salazar, M. L. Bileschi, P. Bork, A. Bridge, L. Colwell, J. Gough, D. H. Haft, I. Letunić, A. Marchler-Bauer, H. Mi, D. A. Natale, C. A. Orengo, A. P. Pandurangan, C. Rivoire, C. J. A. Sigrist, I. Sillitoe, N. Thanki, P. D. Thomas, S. C. E. Tosatto, C. H. Wu, A. Bateman, “InterPro in 2022” *Nucleic Acids Res* **2023**, *51*, D418–D427.

[5] N. Oberg, R. Zallot, J. A. Gerlt, “EFI-EST, EFI-GNT, and EFI-CGFP: Enzyme Function Initiative (EFI) Web Resource for Genomic Enzymology Tools” *Journal of Molecular Biology* **2023**, *435*, 168018.

[6] P. Shannon, A. Markiel, O. Ozier, N. S. Baliga, J. T. Wang, D. Ramage, N. Amin, B. Schwikowski, T. Ideker, “Cytoscape: A Software Environment for Integrated Models of Biomolecular Interaction Networks” *Genome Res* **2003**, *13*, 2498–2504.

[7] W. Shen, S. Le, Y. Li, F. Hu, “SeqKit: A Cross-Platform and Ultrafast Toolkit for FASTA/Q File Manipulation” *PLOS ONE* **2016**, *11*, e0163962.

[8] K. Blin, S. Shaw, H. E. Augustijn, Z. L. Reitz, F. Biermann, M. Alanjary, A. Fetter, B. R. Terlouw, W. W. Metcalf, E. J. N. Helfrich, G. P. van Wezel, M. H. Medema, T. Weber, “antiSMASH 7.0: new and improved predictions for detection, regulation, chemical structures and visualisation” *Nucleic Acids Research* **2023**, *51*, W46–W50.

[9] T. Hackl, M. Ankenbrand, B. van Adrichem, D. Wilkins, K. Haslinger, **2024**, arXiv preprint, DOI: 10.48550/arXiv.2411.13556.

[10] P. Püllmann, A. Knorrscheidt, J. Münch, P. R. Palme, W. Hoehenwarter, S. Marillonnet, M. Alcalde, B. Westermann, M. J. Weissenborn, “A modular two yeast species secretion system for the production and preparative application of unspecific peroxygenases” *Communications Biology* **2021**, *4*, 1–20.

[11] S. Cabantous, G. S. Waldo, “*In vivo* and *in vitro* protein solubility assays using split GFP” *Nat Methods* **2006**, *3*, 845–854.

[12] R. Schmid, S. Heuckeroth, A. Korf, A. Smirnov, O. Myers, T. S. Dyrlund, R. Bushuiev, K. J. Murray, N. Hoffmann, M. Lu, A. Sarvepalli, Z. Zhang, M. Fleischauer, K. Dührkop, M. Wesner, S. J. Hoogstra, E. Rudt, O. Mokshyna, C. Brungs, K. Ponomarov, L. Mutabdžija, T. Damiani, C. J. Pudney, M. Earll, P. O. Helmer, T. R. Fallon, T. Schulze, A. Rivas-Ubach, A. Bilbao, H. Richter, L.-F. Nothias, M. Wang, M. Orešič, J.-K. Weng, S. Böcker, A. Jeibmann, H. Hayen, U. Karst, P. C. Dorrestein, D. Petras, X. Du, T. Pluskal, “Integrative analysis of multimodal mass spectrometry data in MZmine 3” *Nat Biotechnol* **2023**, *41*, 447–449.

[13] C. Rautengarten, E. Baidoo, J. D. Keasling, H. V. Scheller, “A Simple Method for Enzymatic Synthesis of Unlabeled and Radiolabeled Hydroxycinnamate-CoA” *Bioenerg. Res.* **2010**, *3*, 115–122.

[14] J. P. Coleman, L. L. Hudson, S. L. McKnight, J. M. Farrow, M. W. Calfee, C. A. Lindsey, E. C. Pesci, “*Pseudomonas aeruginosa* PqsA Is an Anthranilate-Coenzyme A Ligase” *J Bacteriol* **2008**, *190*, 1247–1255.

[15] A. Kawaguchi, T. Yoshimura, S. Okuda, “A new method for the preparation of acyl-CoA thioesters” *J Biochem* **1981**, *89*, 337–339.

[16] H. Yang, V. A. Zingaro, J. Lincoff, H. Tom, S. Oikawa, J. A. Oses-Prieto, Q. Edmondson, I. Seiple, H. Shah, S. Kajimura, A. L. Burlingame, M. Grabe, D. Ruggero, “Remodelling of the translatome controls diet and its impact on tumorigenesis” *Nature* **2024**, *633*, 189–197.

[17] G. Landrum, P. Tosco, B. Kelley, R. Rodriguez, D. Cosgrove, R. Vianello, sriniker, P. Gedeck, G. Jones, NadineSchneider, E. Kawashima, D. Nealschneider, A. Dalke, M. Swain, B. Cole, S. Turk, A. Savelev, A. Vaucher, M. Wójcikowski, I. Take, V. F. Scalfani, R. Walker, K. Ujihara, D. Probst, tadhurst-cdd, guillaume godin, A. Pahl, J. Lehtivarjo, F. Bérenger, strets123 **2024**, DOI 10.5281/zenodo.13469390.

[18] A. Elnaggar, M. Heinzinger, C. Dallago, G. Rehawi, Y. Wang, L. Jones, T. Gibbs, T. Feher, C. Angerer, M. Steinegger, D. Bhowmik, B. Rost, “ProtTrans: Toward Understanding the Language of Life Through Self-Supervised Learning” *IEEE Transactions on Pattern Analysis and Machine Intelligence* **2022**, *44*, 7112–7127.

[19] F. Pedregosa, G. Varoquaux, A. Gramfort, V. Michel, B. Thirion, O. Grisel, M. Blondel, P. Prettenhofer, R. Weiss, V. Dubourg, J. Vanderplas, A. Passos, D. Cournapeau, M. Brucher, M. Perrot, E. Duchesnay, G. Louppe, “Scikit-learn: Machine Learning in Python” *Journal of Machine Learning Research* **2012**, *12*.

[20] D. Osorio, P. Rondón-Villarreal, R. Torres, “Peptides: A Package for Data Mining of Antimicrobial Peptides” *The R Journal* **2015**, *7*, 4–14.

[21] J. Jumper, R. Evans, A. Pritzel, T. Green, M. Figurnov, O. Ronneberger, K. Tunyasuvunakool, R. Bates, A. Žídek, A. Potapenko, A. Bridgland, C. Meyer, S. A. A. Kohl, A. J. Ballard, A. Cowie, B. Romera-Paredes, S. Nikolov, R. Jain, J. Adler, T. Back, S. Petersen, D. Reiman, E. Clancy, M. Zielinski, M. Steinegger, M. Pacholska, T. Berghammer, S. Bodenstein, D. Silver, O. Vinyals, A. W. Senior, K. Kavukcuoglu, P. Kohli, D. Hassabis, “Highly accurate protein structure prediction with AlphaFold” *Nature* **2021**, *596*, 583–589.

[22] H. Wickham, *ggplot2*, Springer International Publishing, Cham, **2016**.

[23] Z. Gu, “Complex heatmap visualization” *iMeta* **2022**, *1*, e43.

[24] I. Kjærbølling, T. Vesth, J. C. Frisvad, J. L. Nybo, S. Theobald, S. Kildgaard, T. I. Petersen, A. Kuo, A. Sato, E. K. Lyhne, M. E. Kogle, A. Wiebenga, R. S. Kun, R. J. M. Lubbers, M. R. Mäkelä, K. Barry, M. Chovatia, A. Clum, C. Daum, S. Haridas, G. He, K. LaButti, A. Lipzen, S. Mondo, J. Pangilinan, R. Riley, A. Salamov, B. A. Simmons, J. K. Magnuson, B. Henrissat, U. H. Mortensen, T. O. Larsen, R. P. de Vries, I. V. Grigoriev, M. Machida, S. E. Baker, M. R. Andersen, “A comparative genomics study of 23 *Aspergillus* species from section *Flavi*” *Nat Commun* **2020**, *11*, 1106.

[25] T. C. Vesth, J. L. Nybo, S. Theobald, J. C. Frisvad, T. O. Larsen, K. F. Nielsen, J. B. Hoof, J. Brandl, A. Salamov, R. Riley, J. M. Gladden, P. Phatale, M. T. Nielsen, E. K. Lyhne, M. E. Kogle, K. Strasser, E. McDonnell, K. Barry, A. Clum, C. Chen, K. LaButti, S. Haridas, M. Nolan, L. Sandor, A. Kuo, A. Lipzen, M. Hainaut, E. Drula, A. Tsang, J. K. Magnuson, B. Henrissat, A. Wiebenga, B. A. Simmons, M. R. Mäkelä, R. P. de Vries, I. V. Grigoriev, U. H. Mortensen, S. E. Baker, M. R. Andersen, “Investigation of inter- and intraspecies variation through genome sequencing of *Aspergillus* section *Nigri*” *Nat Genet* **2018**, *50*, 1688–1695.

[26] M. E. E. Franco, J. H. Wisecaver, A. E. Arnold, Y.-M. Ju, J. C. Slot, S. Ahrendt, L. P. Moore, K. E. Eastman, K. Scott, Z. Konkel, S. J. Mondo, A. Kuo, R. D. Hayes, S. Haridas, B. Andreopoulos, R. Riley, K. LaButti, J. Pangilinan, A. Lipzen, M. Amirebrahimi, J. Yan, C. Adam, K. Keymanesh, V. Ng, K. Louie, T. Northen, E. Drula, B. Henrissat, H.-M. Hsieh, K. Youens-Clark, F. Lutzoni, J. Miadlikowska, D. C. Eastwood, R. C. Hamelin, I. V. Grigoriev, J. M. U’Ren, “Ecological generalism drives hyperdiversity of secondary metabolite gene clusters in xylarialean endophytes” *New Phytol* **2022**, *233*, 1317–1330.

[27] D. G. Knapp, J. B. Németh, K. Barry, M. Hainaut, B. Henrissat, J. Johnson, A. Kuo, J. H. P. Lim, A. Lipzen, M. Nolan, R. A. Ohm, L. Tamás, I. V. Grigoriev, J. W. Spatafora, L. G. Nagy, G. M. Kovács, “Comparative genomics provides insights into the lifestyle and reveals functional heterogeneity of dark septate endophytic fungi” *Sci Rep* **2018**, *8*, 6321.

[28] N. Hensen, L. Bonometti, I. Westerberg, I. O. Brännström, S. Guillou, S. Cros-Aarteil, S. Calhoun, S. Haridas, A. Kuo, S. Mondo, J. Pangilinan, R. Riley, K. LaButti, B. Andreopoulos, A. Lipzen, C. Chen, M. Yan, C. Daum, V. Ng, A. Clum, A. Steindorff, R. A. Ohm, F. Martin, P. Silar, D. O. Natvig, C. Lalanne, V. Gautier, S. L. Ament-Velásquez, Å. Kruys, M. I. Hutchinson, A. J. Powell, K. Barry, A. N. Miller, I. V. Grigoriev, R. Debuchy, P. Gladieux, M. Hiltunen Thorén, H. Johannesson, “Genome-scale phylogeny and comparative genomics of the fungal order Sordariales” *Mol Phylogenet Evol* **2023**, *189*, 107938.

[29] F. Mesny, S. Miyauchi, T. Thiergart, B. Pickel, L. Atanasova, M. Karlsson, B. Hüttel, K. W. Barry, S. Haridas, C. Chen, D. Bauer, W. Andreopoulos, J. Pangilinan, K. LaButti, R. Riley, A. Lipzen, A. Clum, E. Drula, B. Henrissat, A. Kohler, I. V. Grigoriev, F. M. Martin, S. Hacquard, “Genetic determinants of endophytism in the *Arabidopsis* root mycobiome” *Nat Commun* **2021**, *12*, 7227.

[30] R. Baroncelli, F. Scala, M. Vergara, M. R. Thon, M. Ruocco, “Draft whole-genome sequence of the *Diaporthe helianthi* 7/96 strain, causal agent of sunflower stem canker” *Genom Data* **2016**, *10*, 151–152.

[31] V. A. Vicente, V. A. Weiss, A. Bombassaro, L. F. Moreno, F. F. Costa, R. T. Raittz, A. C. Leão, R. R. Gomes, A. L. Bocca, G. Fornari, R. J. A. de Castro, J. Sun, H. Faoro, M. Z. Tadra-Sfeir, V. Baura, E. Balsanelli, S. R. Almeida, S. S. Dos Santos, M. de M. Teixeira, M. S. Soares Felipe, M. M. F. do Nascimento, F. O. Pedrosa, M. B. Steffens, D. Attili-Angelis, M. J. Najafzadeh, F. Queiroz-Telles, E. M. Souza, S. De Hoog, “Comparative Genomics of Sibling Species of *Fonsecaea* Associated with Human Chromoblastomycosis” *Front Microbiol* **2017**, *8*, 1924.

[32] E.-M. Niehaus, M. Münsterkötter, R. H. Proctor, D. W. Brown, A. Sharon, Y. Idan, L. Oren-Young, C. M. Sieber, O. Novák, A. Pěnčík, D. Tarkowská, K. Hromadová, S. Freeman, M. Maymon, M. Elazar, S. A. Youssef, E. S. M. El-Shabrawy, A. B. A. Shalaby, P. Houterman, N. L. Brock, I. Burkhardt, E. A. Tsavkelova, J. S. Dickschat, P. Galuszka, U. Güldener, B. Tudzynski, “Comparative ‘Omics’ of the *Fusarium fujikuroi* Species Complex Highlights Differences in Genetic Potential and Metabolite Synthesis” *Genome Biol Evol* **2016**, *8*, 3574–3599.

[33] R. A. Dean, N. J. Talbot, D. J. Ebbole, M. L. Farman, T. K. Mitchell, M. J. Orbach, M. Thon, R. Kulkarni, J.-R. Xu, H. Pan, N. D. Read, Y.-H. Lee, I. Carbone, D. Brown, Y. Y. Oh, N. Donofrio, J. S. Jeong, D. M. Soanes, S. Djonovic, E. Kolomiets, C. Rehmeyer, W. Li, M. Harding, S. Kim, M.-H. Lebrun, H. Bohnert, S. Coughlan, J. Butler, S. Calvo, L.-J. Ma, R. Nicol, S. Purcell, C. Nusbaum, J. E. Galagan, B. W. Birren, “The genome sequence of the rice blast fungus *Magnaporthe grisea*” *Nature* **2005**, *434*, 980–986.

[34] C. B. Harder, S. Miyauchi, M. Virágh, A. Kuo, E. Thoen, B. Andreopoulos, D. Lu, I. Skrede, E. Drula, B. Henrissat, E. Morin, A. Kohler, K. Barry, K. LaButti, A. Salamov, A. Lipzen, Z. Merényi, B. Hegedüs, P. Baldrian, M. Stursova, H. Weitz, A. Taylor, M. Koriabine, E. Savage, I. V. Grigoriev, L. G. Nagy, F. Martin, H. Kauserud, “Extreme overall mushroom genome expansion in Mycena s.s. irrespective of plant hosts or substrate specializations” *Cell Genom* **2024**, *4*, 100586.

[35] E. Espagne, O. Lespinet, F. Malagnac, C. Da Silva, O. Jaillon, B. M. Porcel, A. Couloux, J.-M. Aury, B. Ségurens, J. Poulain, V. Anthouard, S. Grossetete, H. Khalili, E. Coppin, M. Déquard-Chablat, M. Picard, V. Contamine, S. Arnaise, A. Bourdais, V. Berteaux-Lecellier, D. Gautheret, R. P. de Vries, E. Battaglia, P. M. Coutinho, E. G. Danchin, B. Henrissat, R. E. Khoury, A. Sainsard-Chanet, A. Boivin, B. Pinan-Lucarré, C. H. Sellem, R. Debuchy, P. Wincker, J. Weissenbach, P. Silar, “The genome sequence of the model ascomycete fungus *Podospora anserina*” *Genome Biol* **2008**, *9*, R77.

[36] X. Wang, X. Zhang, L. Liu, M. Xiang, W. Wang, X. Sun, Y. Che, L. Guo, G. Liu, L. Guo, C. Wang, W.-B. Yin, M. Stadler, X. Zhang, X. Liu, “Genomic and transcriptomic analysis of the endophytic fungus *Pestalotiopsis fici* reveals its lifestyle and high potential for synthesis of natural products” *BMC Genomics* **2015**, *16*, 28.

[37] J. C. Nielsen, S. Grijseels, S. Prigent, B. Ji, J. Dainat, K. F. Nielsen, J. C. Frisvad, M. Workman, J. Nielsen, “Global analysis of biosynthetic gene clusters reveals vast potential of secondary metabolite production in Penicillium species” *Nature Microbiology* **2017**, *2*, 1–9.

[38] V. A. Manning, I. Pandelova, B. Dhillon, L. J. Wilhelm, S. B. Goodwin, A. M. Berlin, M. Figueroa, M. Freitag, J. K. Hane, B. Henrissat, W. H. Holman, C. D. Kodira, J. Martin, R. P. Oliver, B. Robbertse, W. Schackwitz, D. C. Schwartz, J. W. Spatafora, B. G. Turgeon, C. Yandava, S. Young, S. Zhou, Q. Zeng, I. V. Grigoriev, L.-J. Ma, L. M. Ciuffetti, “Comparative genomics of a plant-pathogenic fungus, *Pyrenophora tritici-repentis*, reveals transduplication and the impact of repeat elements on pathogenicity and population divergence” *G3 (Bethesda)* **2013**, *3*, 41–63.

[39] J. M. Palmer, K. P. Drees, J. T. Foster, D. L. Lindner, “Extreme sensitivity to ultraviolet light in the fungal pathogen causing white-nose syndrome of bats” *Nat Commun* **2018**, *9*, 35.

[40] Y. Shang, G. Xiao, P. Zheng, K. Cen, S. Zhan, C. Wang, “Divergent and Convergent Evolution of Fungal Pathogenicity” *Genome Biol Evol* **2016**, *8*, 1374–1387.

[41] D. A. Martinez, B. G. Oliver, Y. Gräser, J. M. Goldberg, W. Li, N. M. Martinez-Rossi, M. Monod, E. Shelest, R. C. Barton, E. Birch, A. A. Brakhage, Z. Chen, S. J. Gurr, D. Heiman, J. Heitman, I. Kosti, A. Rossi, S. Saif, M. Samalova, C. W. Saunders, T. Shea, R. C. Summerbell, J. Xu, S. Young, Q. Zeng, B. W. Birren, C. A. Cuomo, T. C. White, “Comparative genome analysis of *Trichophyton rubrum* and related dermatophytes reveals candidate genes involved in infection” *mBio* **2012**, *3*, e00259-00212.

[42] Z. Yin, H. Liu, Z. Li, X. Ke, D. Dou, X. Gao, N. Song, Q. Dai, Y. Wu, J.-R. Xu, Z. Kang, L. Huang, “Genome sequence of *Valsa canker* pathogens uncovers a potential adaptation of colonization of woody bark” *New Phytologist* **2015**, *208*, 1202–1216.

[43] N. Funa, T. Awakawa, S. Horinouchi, “Pentaketide resorcylic acid synthesis by type III polyketide synthase from *Neurospora crassa*” *Journal of Biological Chemistry* **2007**, *282*, 14476–14481.

[44] J. Li, Y. Luo, J. K. Lee, H. Zhao, “Cloning and characterization of a type III polyketide synthase from *Aspergillus niger*” *Bioorganic and Medicinal Chemistry Letters* **2011**, *21*, 6085–6089.

[45] K. Kirimura, S. Watanabe, K. Kobayashi, “Heterologous gene expression and functional analysis of a type III polyketide synthase from *Aspergillus niger* NRRL 328” *Biochemical and Biophysical Research Communications* **2016**, *473*, 1106–1110.

[46] D. Yu, J. Zeng, D. Chen, J. Zhan, “Characterization and reconstitution of a new fungal type III polyketide synthase from *Aspergillus oryzae*” *Enzyme and Microbial Technology* **2010**, *46*, 575–580.

[47] M. Jeya, T. S. Kim, M. K. Tiwari, J. Li, H. Zhao, J. K. Lee, “The *Botrytis cinerea* type III polyketide synthase shows unprecedented high catalytic efficiency toward long chain acyl-CoAs” *Molecular BioSystems* **2012**, *8*, 2864–2867.

[48] L. Sun, S. Wang, S. Zhang, D. Yu, Y. Qin, H. Huang, W. Wang, J. Zhan, “Identification of a type III polyketide synthase involved in the biosynthesis of spirolaxine” *Applied Microbiology and Biotechnology* **2016**, *100*, 7103–7113.

[49] D. Ramakrishnan, M. K. Tiwari, G. Manoharan, T. Sairam, R. Thangamani, J. K. Lee, J. Marimuthu, “Molecular characterization of two alkylresorcylic acid synthases from *Sordariomycetes* fungi” *Enzyme and Microbial Technology* **2018**, *115*, 16–22.

[50] M. Hashimoto, T. Koen, H. Takahashi, C. Suda, K. Kitamoto, I. Fujii, “*Aspergillus oryzae* CsyB Catalyzes the Condensation of Two β-Ketoacyl-CoAs to Form 3-Acetyl-4-hydroxy-6-alkyl-α-pyrone*” *Journal of Biological Chemistry* **2014**, *289*, 19976–19984.

[51] L. Martinelli, V. Redou, B. Cochereau, L. Delage, N. Hymery, E. Poirier, C. Le Meur, G. Le Foch, L. Cladiere, M. Mehiri, N. Demont-Caulet, L. Meslet-Cladiere, “Identification and Characterization of a New Type III Polyketide Synthase from a Marine Yeast, *Naganishia uzbekistanensis*” *Marine Drugs* **2020**, *18*, 637.

[52] G. Manoharan, T. Sairam, R. Thangamani, D. Ramakrishnan, M. K Tiwari, J.-K. Lee, J. Marimuthu, “Identification and characterization of type III polyketide synthase genes from culturable endophytes of ethnomedicinal plants” *Enzyme Microb Technol* **2019**, *131*, 109396.

[53] M. S. Resmi, P. Verma, R. S. Gokhale, E. V. Soniya, “Identification and Characterization of a Type III Polyketide Synthase Involved in Quinolone Alkaloid Biosynthesis from *Aegle marmelos* Correa” *J Biol Chem* **2013**, *288*, 7271–7281.

[54] T. Mori, Y. Shimokawa, T. Matsui, K. Kinjo, R. Kato, H. Noguchi, S. Sugio, H. Morita, I. Abe, “Cloning and Structure-Function Analyses of Quinolone- and Acridone-producing Novel Type III Polyketide Synthases from *Citrus microcarpa*” *Journal of Biological Chemistry* **2013**, *288*, 28845–28858.

[55] I. Abe, T. Abe, K. Wanibuchi, H. Noguchi, “Enzymatic Formation of Quinolone Alkaloids by a Plant Type III Polyketide Synthase” *Org. Lett.* **2006**, *8*, 6063–6065.

[56] T. Abe, H. Morita, H. Noma, T. Kohno, H. Noguchi, I. Abe, “Structure function analysis of benzalacetone synthase from *Rheum palmatum*” *Bioorganic & Medicinal Chemistry Letters* **2007**, *17*, 3161–3166.

[57] I. Abe, Y. Takahashi, H. Morita, H. Noguchi, “Benzalacetone synthase” *European Journal of Biochemistry* **2001**, *268*, 3354–3359.

[58] M. Matsuzawa, Y. Katsuyama, N. Funa, S. Horinouchi, “Alkylresorcylic acid synthesis by type III polyketide synthases from rice *Oryza sativa*” *Phytochemistry* **2010**, *71*, 1059–1067.

[59] N. Funa, Y. Ohnishi, Y. Ebizuka, S. Horinouchi, “Properties and substrate specificity of RppA, a chalcone synthase-related polyketide synthase in Streptomyces griseus” *J Biol Chem* **2002**, *277*, 4628–4635.

[60] N. Funa, H. Ozawa, A. Hirata, S. Horinouchi, “Phenolic lipid synthesis by type III polyketide synthases is essential for cyst formation in Azotobacter vinelandii” *Proceedings of the National Academy of Sciences of the United States of America* **2006**, *103*, 6356–6361.

[61] R. Álvarez-Álvarez, A. Botas, S. M. Albillos, A. Rumbero, J. F. Martín, P. Liras, “Molecular genetics of naringenin biosynthesis, a typical plant secondary metabolite produced by Streptomyces clavuligerus” *Microb Cell Fact* **2015**, *14*, 1–12.

[62] C. Nakano, H. Ozawa, G. Akanuma, N. Funa, S. Horinouchi, “Biosynthesis of Aliphatic Polyketides by Type III Polyketide Synthase and Methyltransferase in Bacillus subtilis” *J Bacteriol* **2009**, *191*, 4916–4923.

[63] W. Zha, S. B. Rubin-Pitel, H. Zhao, “Characterization of the Substrate Specificity of PhlD, a Type III Polyketide Synthase from *Pseudomonas fluorescens**” *Journal of Biological Chemistry* **2006**, *281*, 32036–32047.

[64] S. Grüschow, T. J. Buchholz, W. Seufert, J. S. Dordick, D. H. Sherman, “Substrate Profile Analysis and ACP-Mediated Acyl Transfer in *Streptomyces coelicolor* Type III Polyketide Synthases” *ChemBioChem* **2007**, *8*, 863–868.

[65] J. Cortés, J. Velasco, G. Foster, A. P. Blackaby, B. A. M. Rudd, B. Wilkinson, “Identification and cloning of a type III polyketide synthase required for diffusible pigment biosynthesis in Saccharopolyspora erythraea” *Molecular microbiology* **2002**, *44*, 1213–1224.

[66] S. Li, S. Grüschow, J. S. Dordick, D. H. Sherman, “Molecular Analysis of the Role of Tyrosine 224 in the Active Site of *Streptomyces coelicolor* RppA, a Bacterial Type III Polyketide Synthase*” *Journal of Biological Chemistry* **2007**, *282*, 12765–12772.

[67] B. Peng, L. Zhang, S. He, R. Oerlemans, W. J. Quax, M. R. Groves, K. Haslinger, “Engineering a Plant Polyketide Synthase for the Biosynthesis of Methylated Flavonoids” *J. Agric. Food Chem.* **2024**, *72*, 529–539.

[68] K. Haslinger, T. Hackl, K. L. J. Prather, “Rapid *in vitro* prototyping of O-methyltransferases for pathway applications in *Escherichia coli*” *Cell Chem Biol* **2021**, *28*, 876-886.e4.

[69] J. C. Navarro-Muñoz, J. Collemare, “Evolutionary Histories of Type III Polyketide Synthases in Fungi” *Frontiers in Microbiology* **2020**, *10*, 3018.

[70] S. B. Rubin-Pitel, H. Zhang, T. Vu, J. S. Brunzelle, H. Zhao, S. K. Nair, “Distinct Structural Elements Dictate the Specificity of the Type III Pentaketide Synthase from Neurospora crassa” *Chemistry and Biology* **2008**, *15*, 1079–1090.

[71] J. L. Ferrer, J. M. Jez, M. E. Bowman, R. A. Dixon, J. P. Noel, “Structure of chalcone synthase and the molecular basis of plant polyketide biosynthesis” *Nature Structural Biology* **1999**, *6*, 775–784.

[72] M. Vijayanathan, A. K. Vadakkepat, K. R. Mahendran, A. Sharaf, K. E. H. Frandsen, D. Bandyopadhyay, M. R. Pillai, E. V. Soniya, “Structural and mechanistic insights into Quinolone Synthase to address its functional promiscuity” *Commun Biol* **2024**, *7*, 1–14.

[73] J. P. Noel, M. B. Austin, M. E. Bowman, *Methods of Producing Polyketide Synthase Mutants and Compositions and Uses Thereof*, **2009**, US20090271888A1.
