# Supplementary material for: Unravelling the Functional Diversity of Type III Polyketide Synthases in Fungi
Source: Angew Chem Int Ed Engl. 2025 Sep 4;64(44):e202514786. doi: 10.1002/anie.202514786 (PMC12559476; doi:10.1002/anie.202514786)
Supplement: Supplementary file 2 — Supporting Information [file ANIE-64-e202514786-s001.zip › Supporting_Files/Supporting_File_6.pdf]

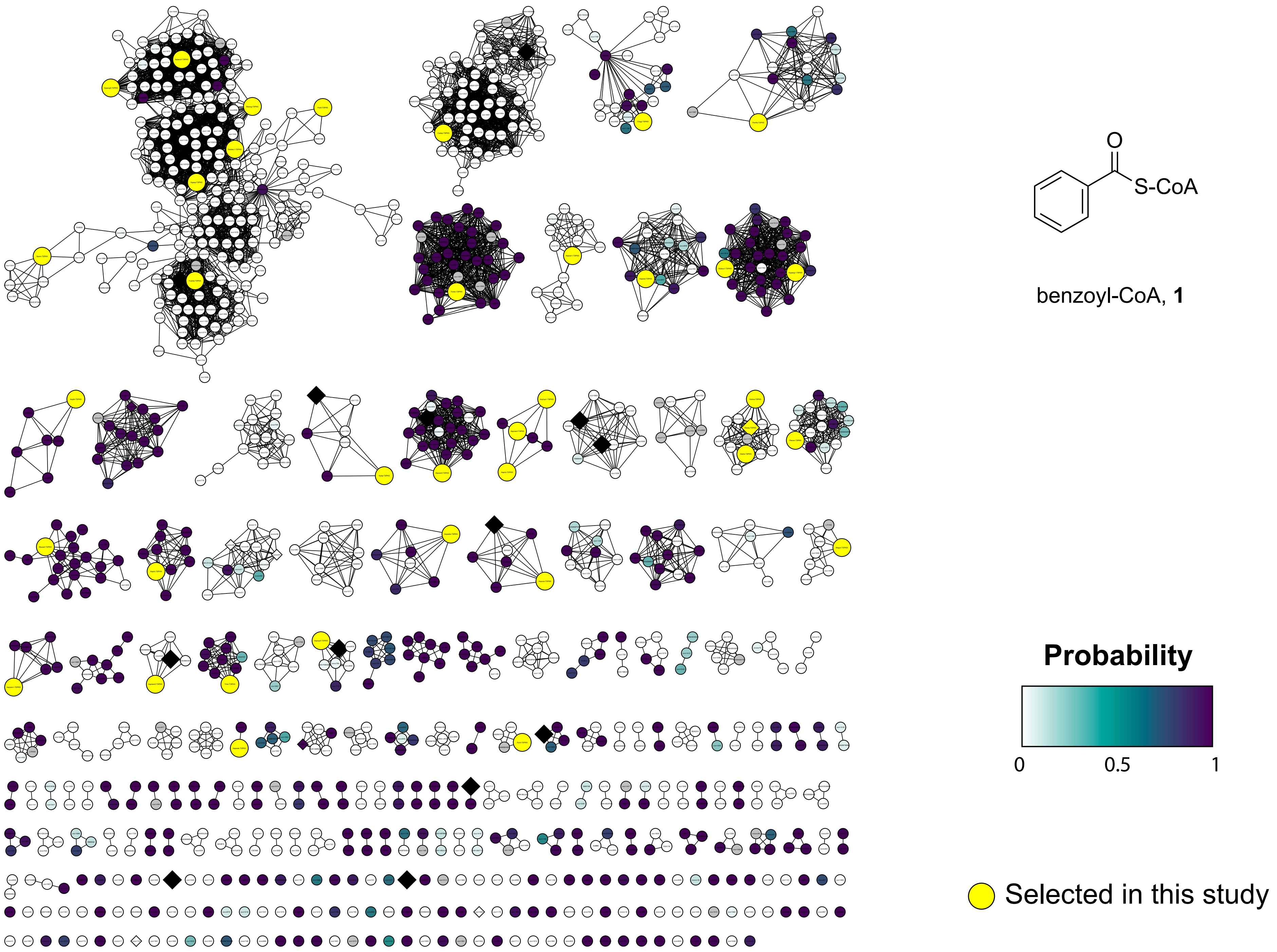
O
S-CoA
benzoyl-CoA, **1**
Probability
0
0.5
1
Selected in this study

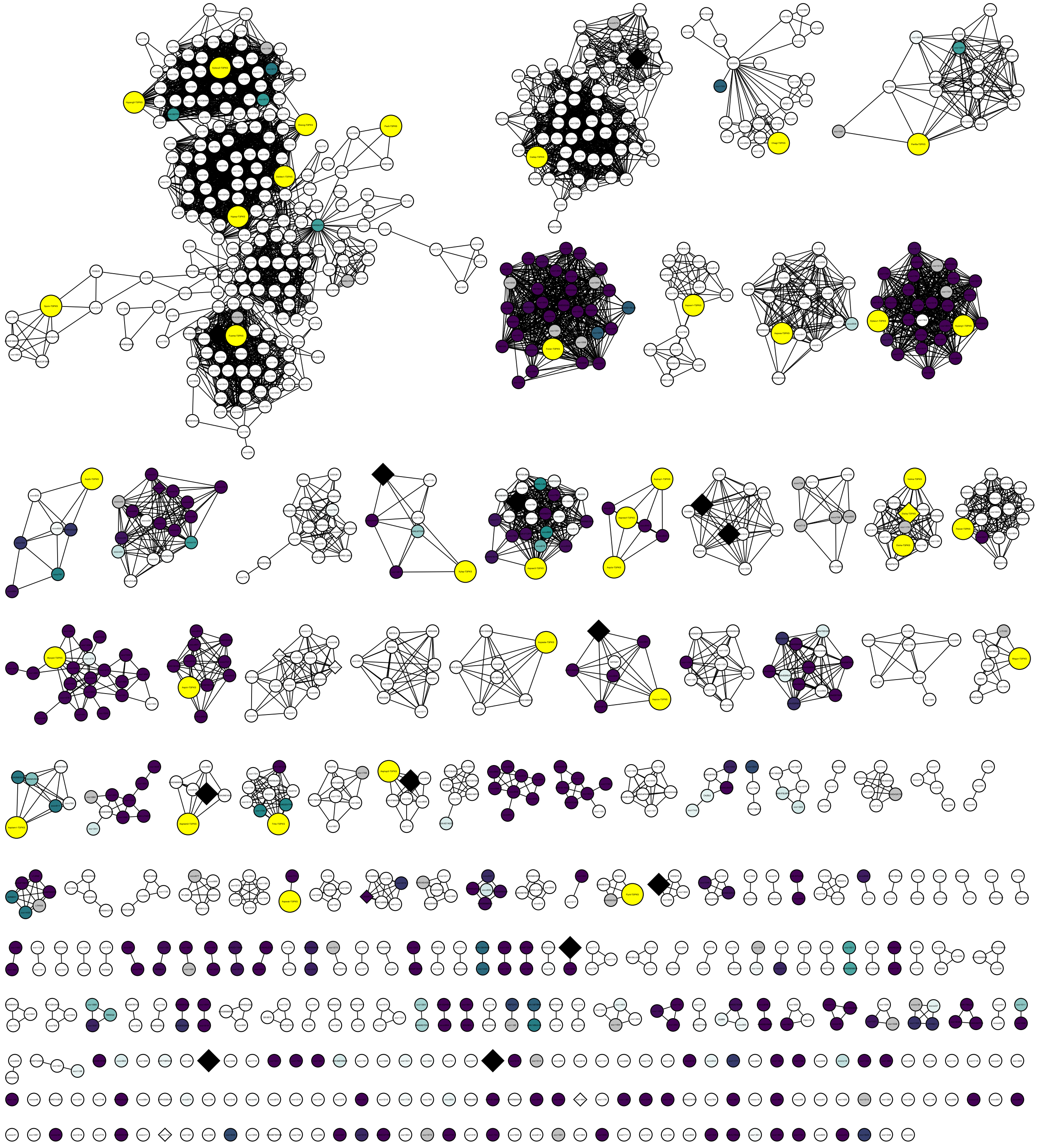

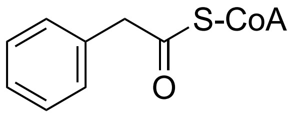

phenylacetyl-CoA, **2**

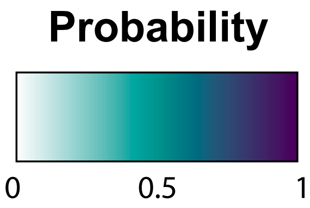

Selected in this study

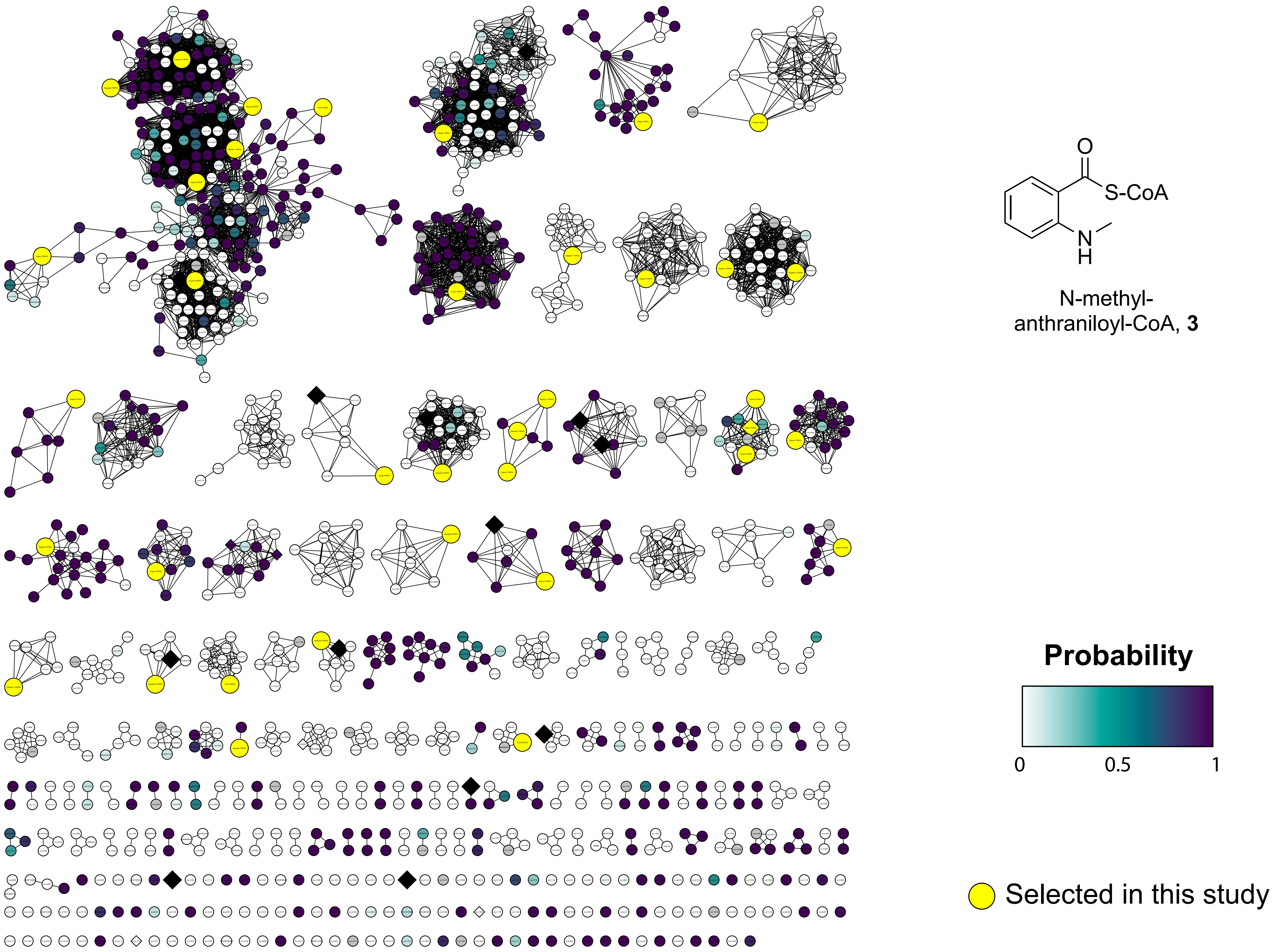
O
S-CoA
N
H
N-methyl-
anthraniloyl-CoA, 3
Probability
0
0.5
1
Selected in this study

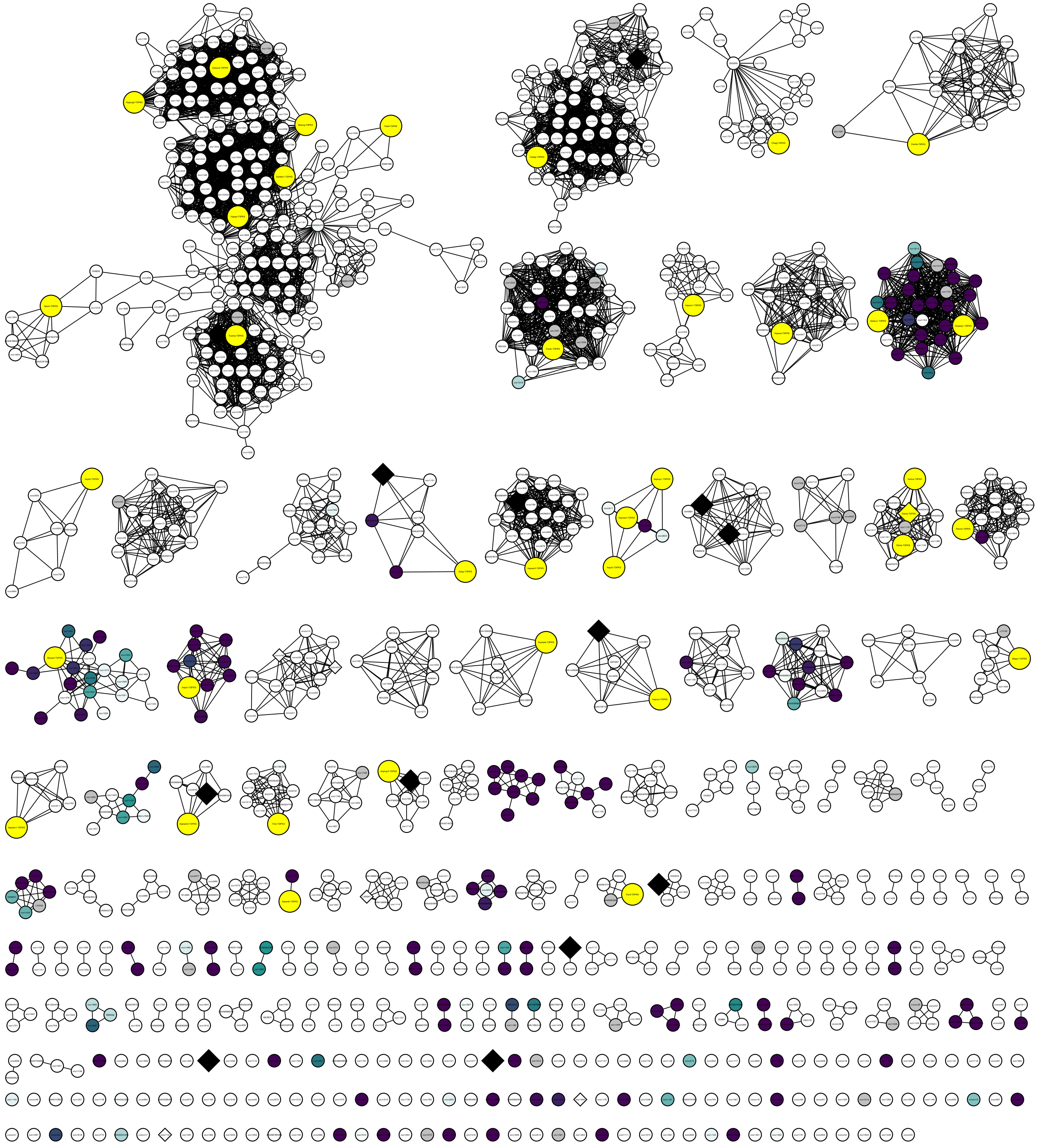

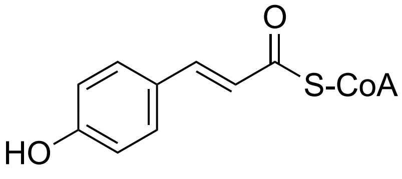

p-coumaroyl-CoA, **4**

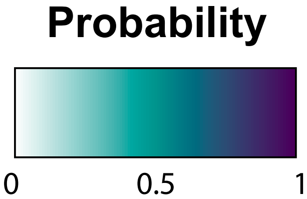

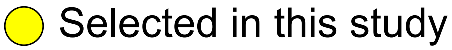

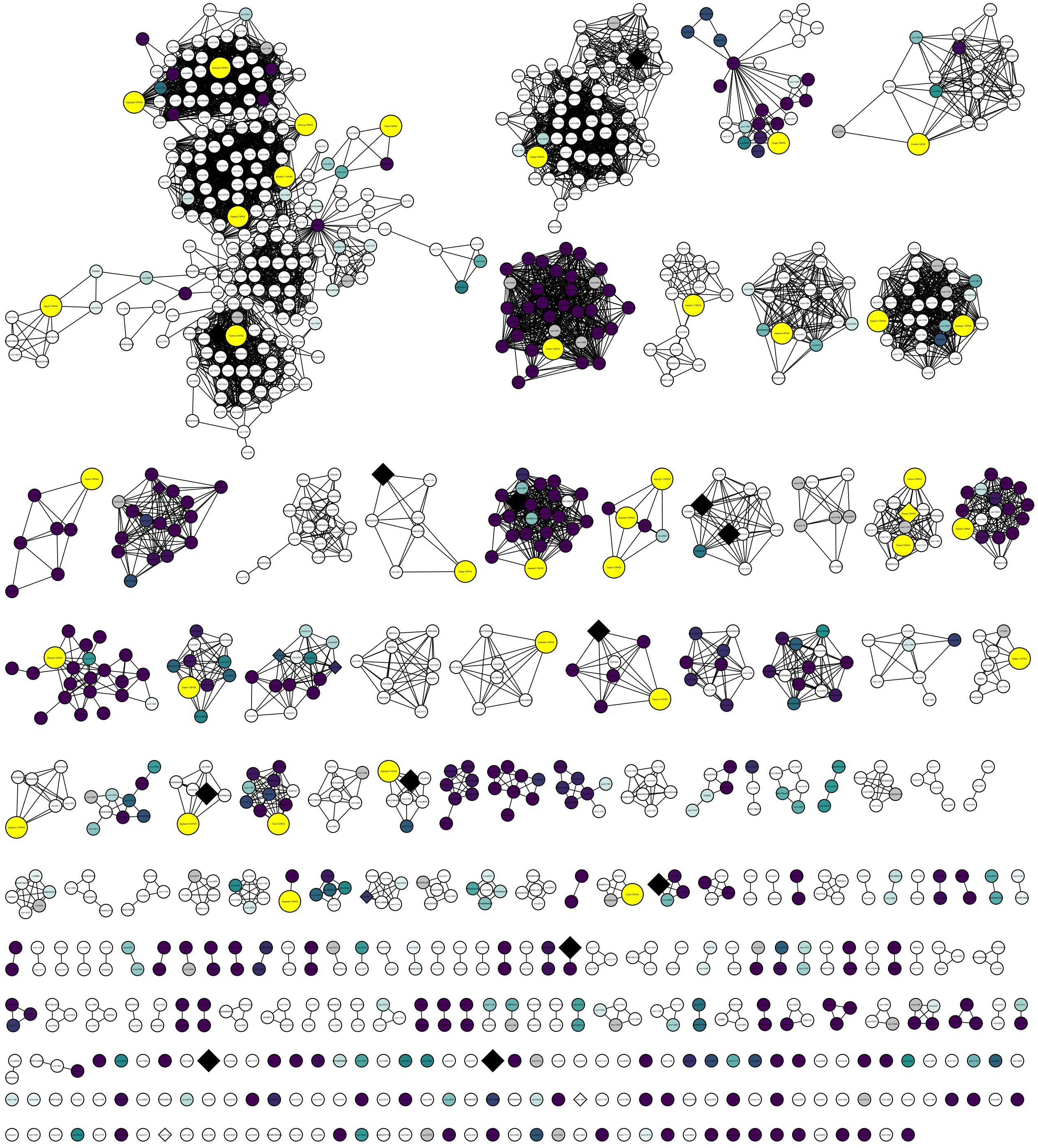

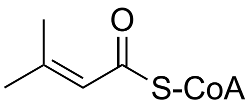

β-methyl-
crotonyl-CoA, **5**

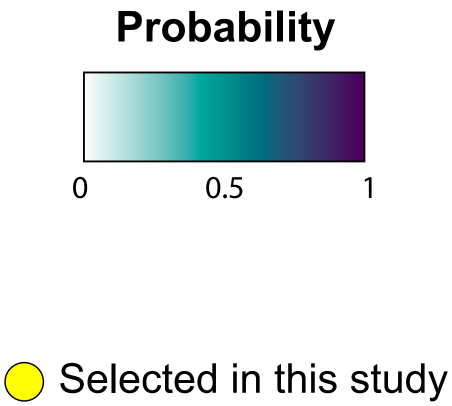

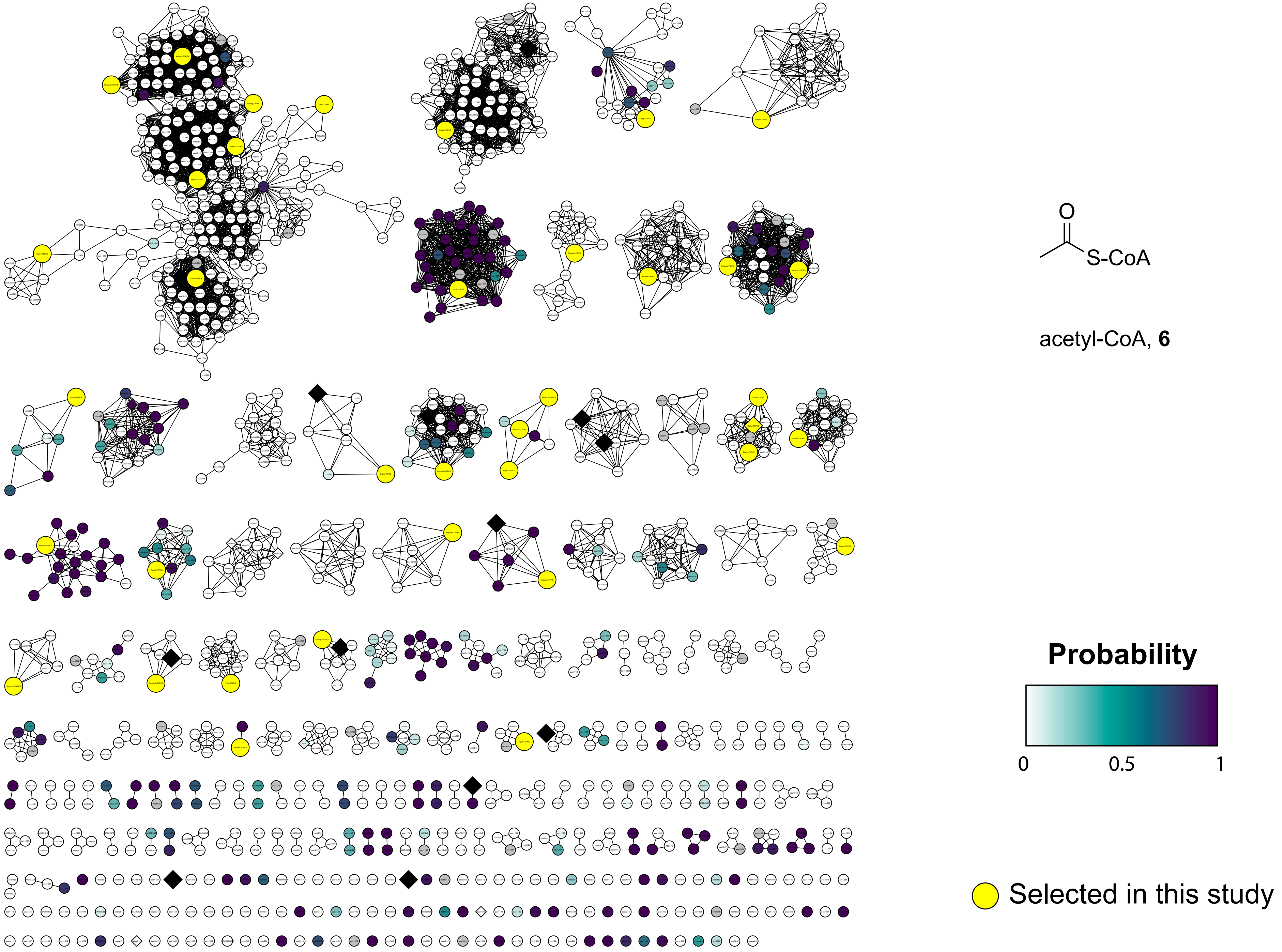

O
S-CoA
acetyl-CoA, **6**
Probability
0
0.5
1
Selected in this study

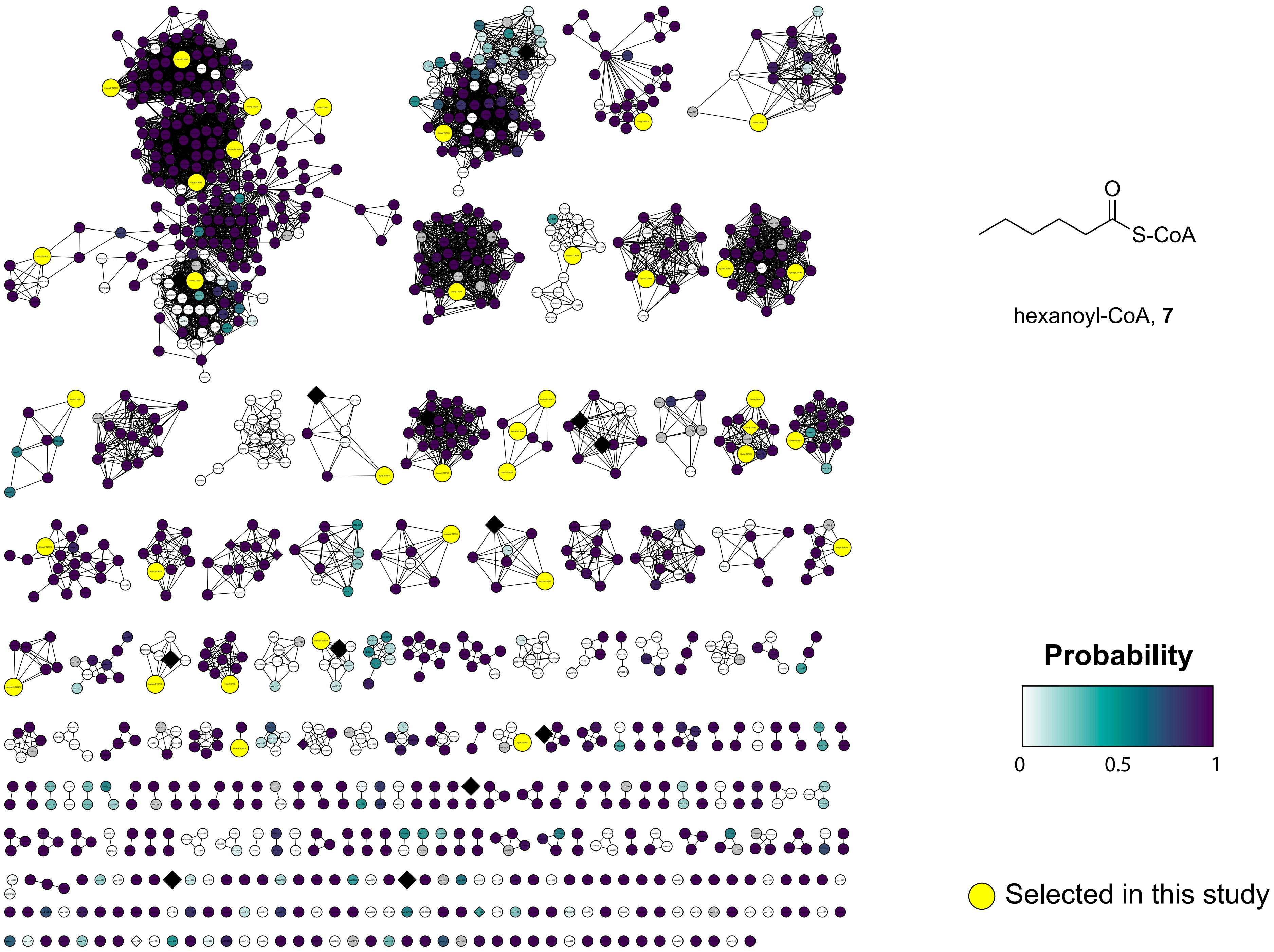
O
S-CoA
hexanoyl-CoA, **7**
Probability
0
0.5
1
Selected in this study

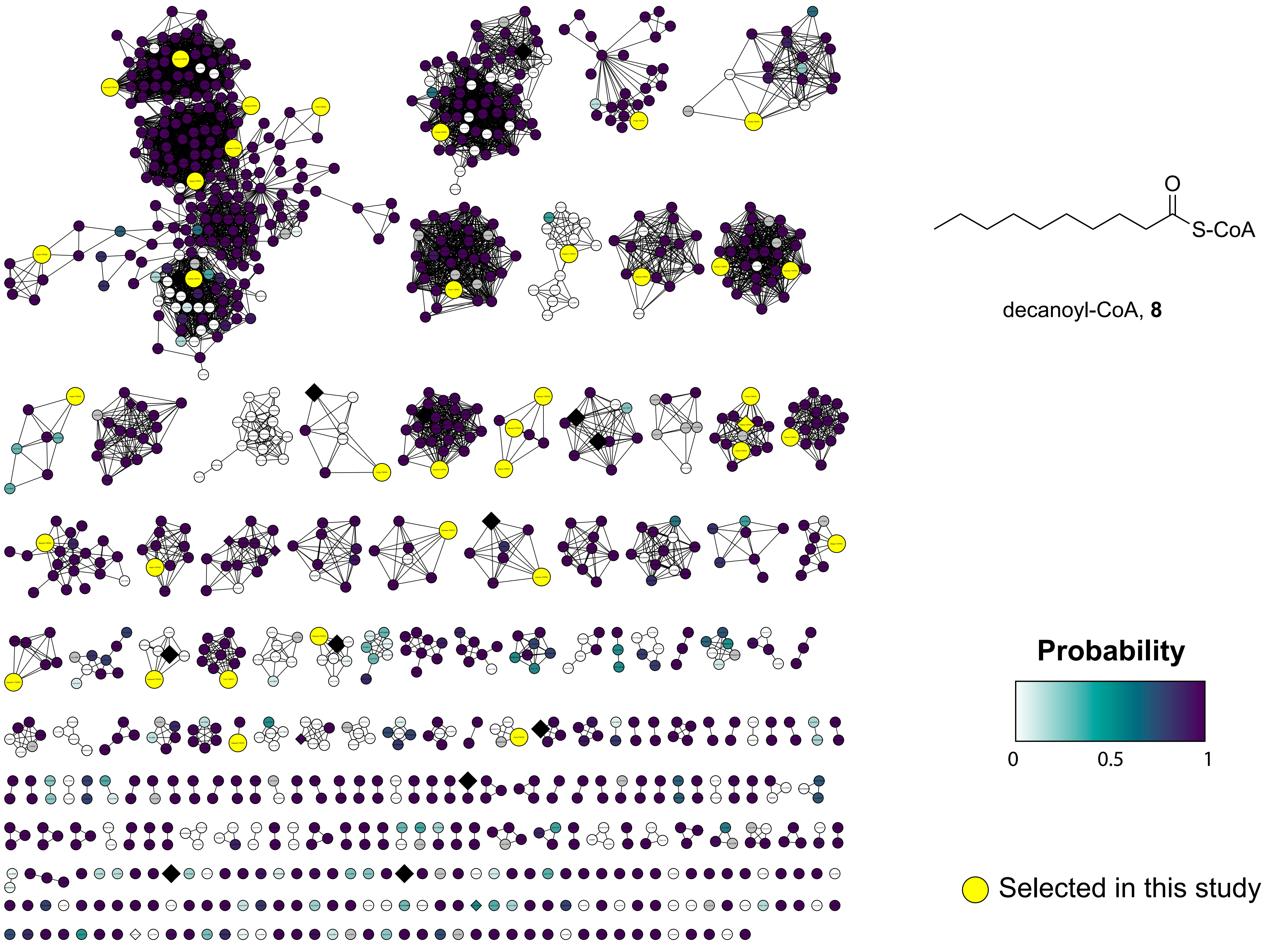
O
S-CoA
decanoyl-CoA, **8**
Probability
0
0.5
1
Selected in this study

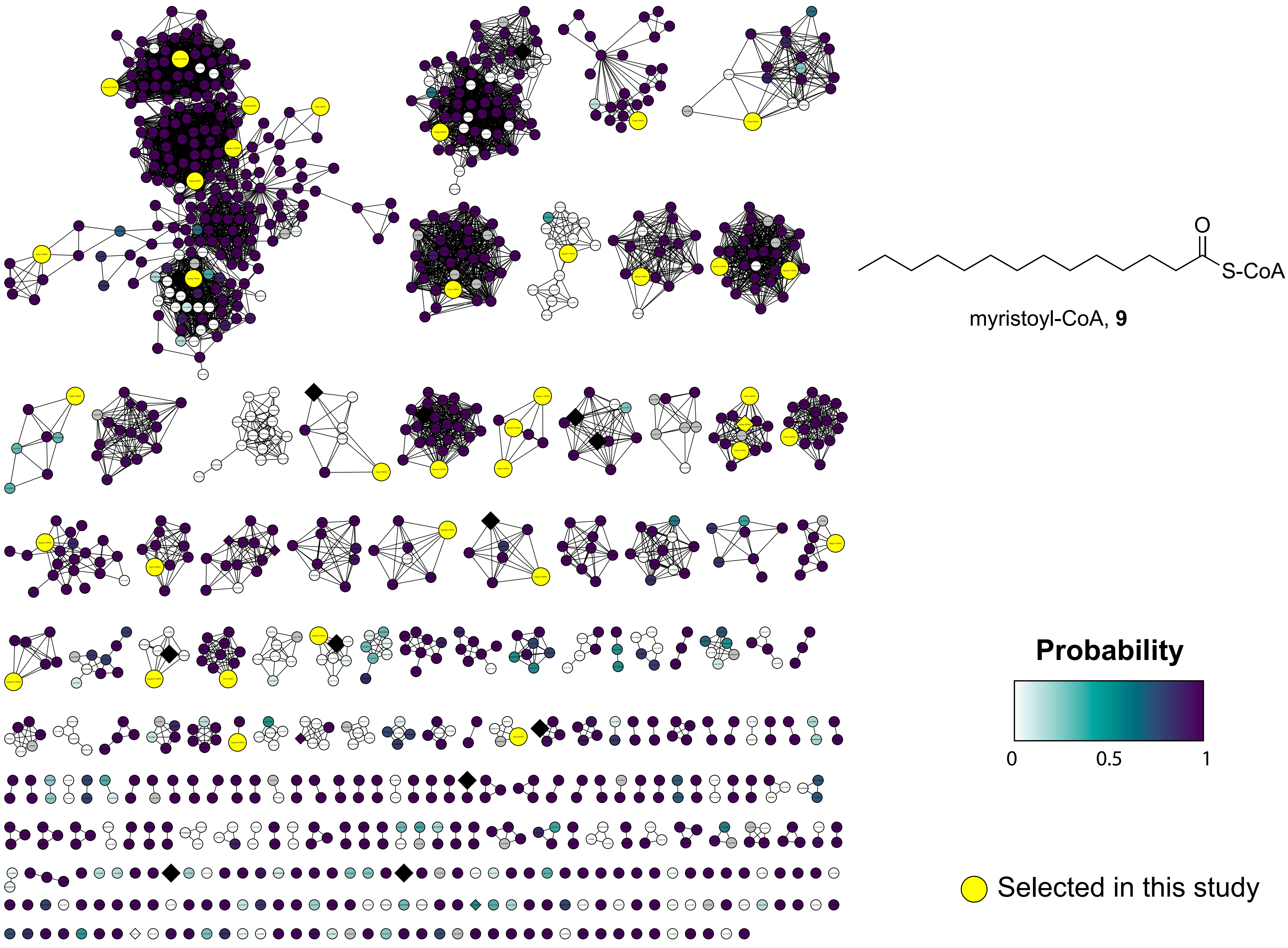
O
S-CoA
myristoyl-CoA, **9**
Probability
0
0.5
1
Selected in this study

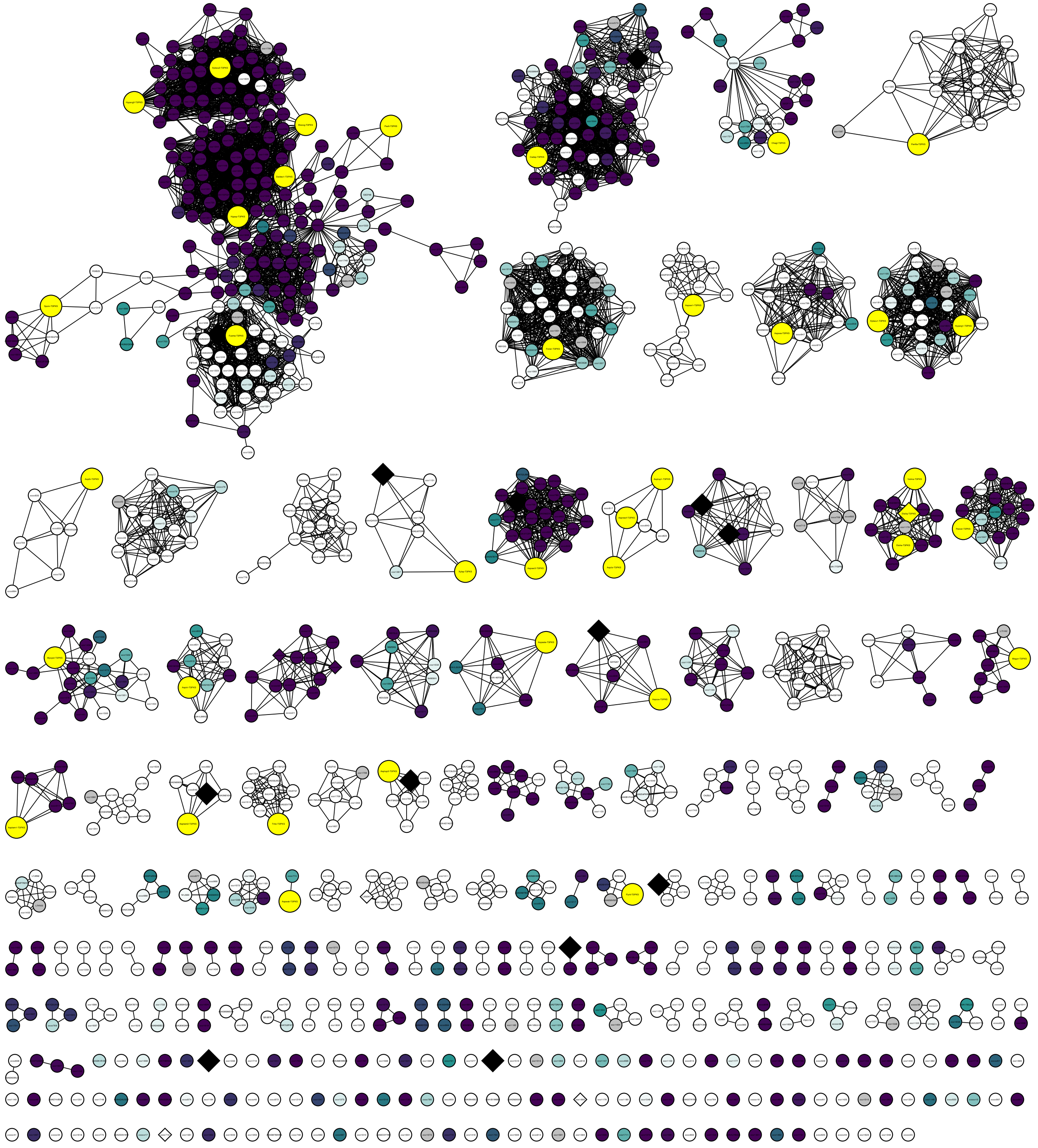

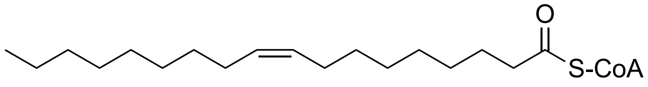

oleoyl-CoA, **10**

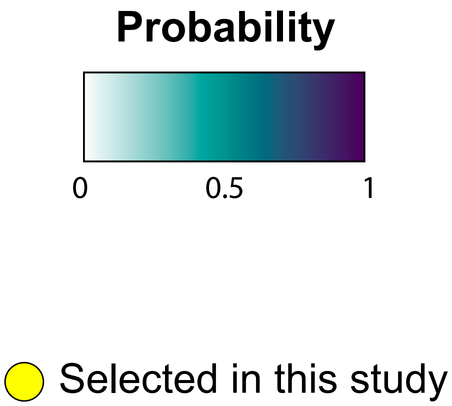

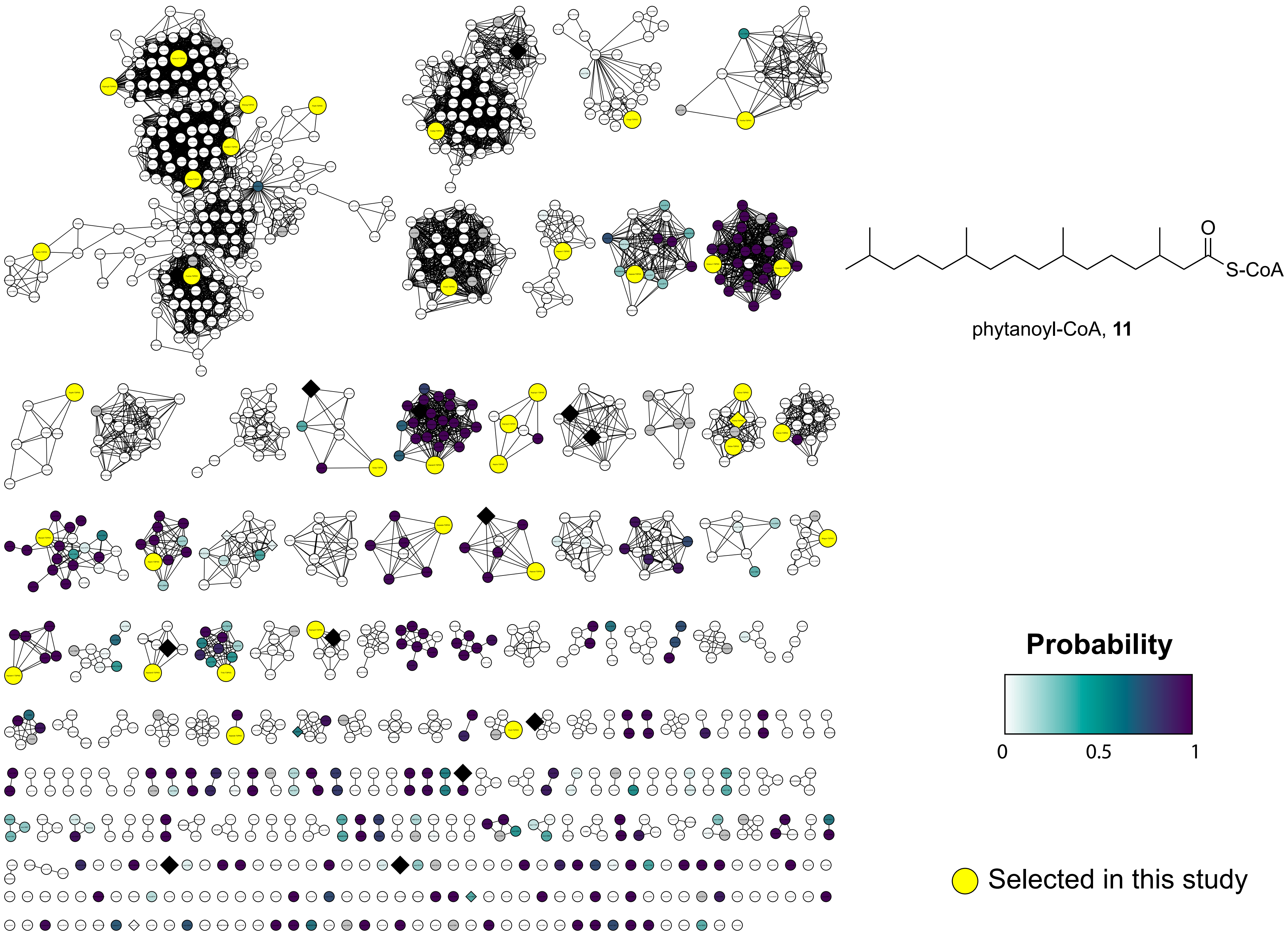
O
S-CoA
phytanoyl-CoA, **11**
Probability
0
0.5
1
Selected in this study

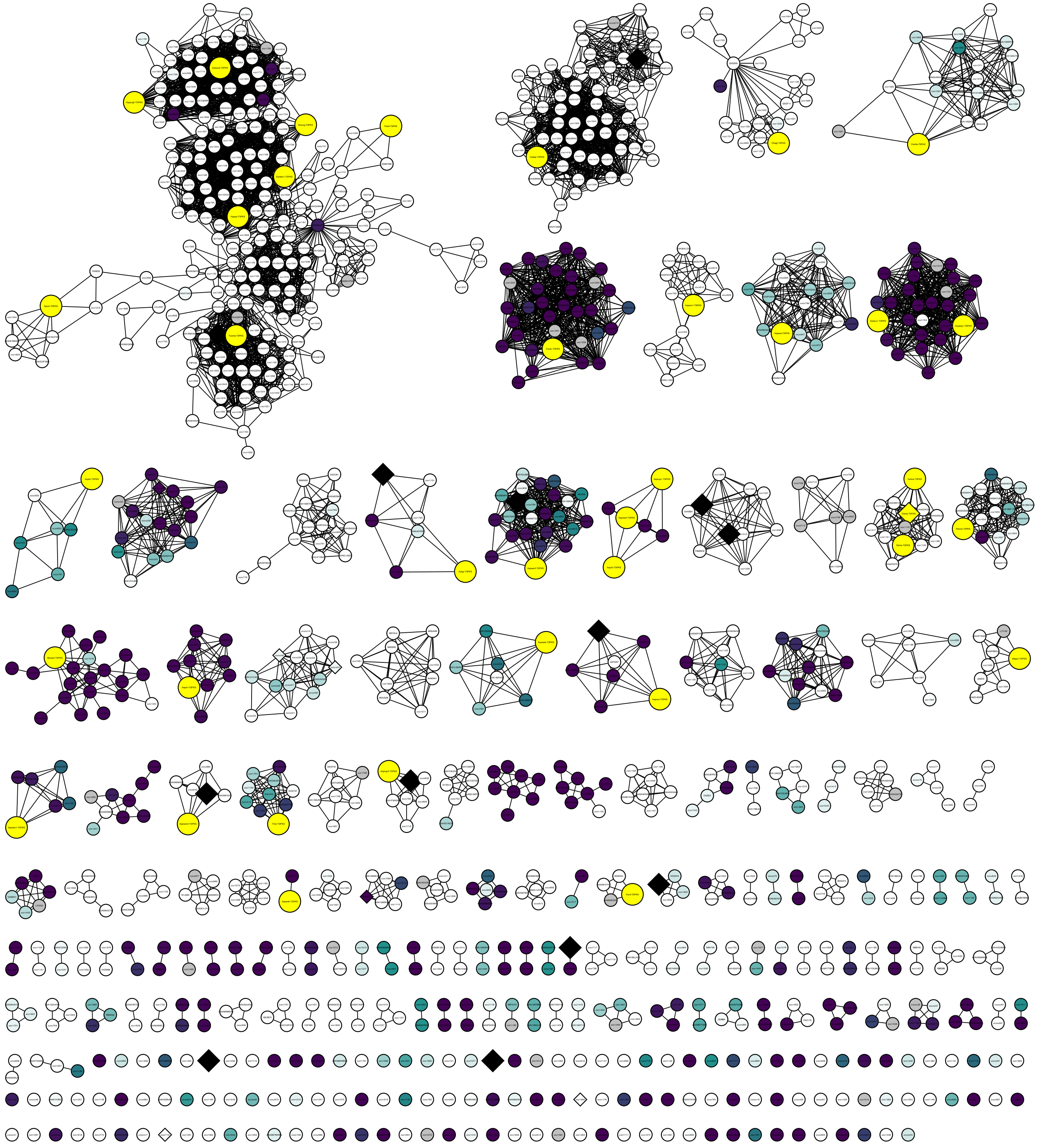

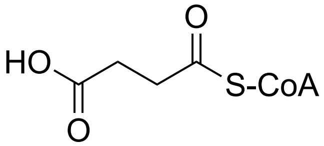

succinyl-CoA, **12**

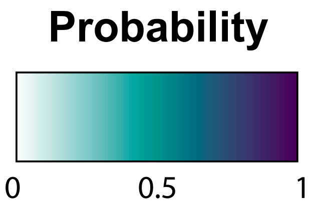

Selected in this study

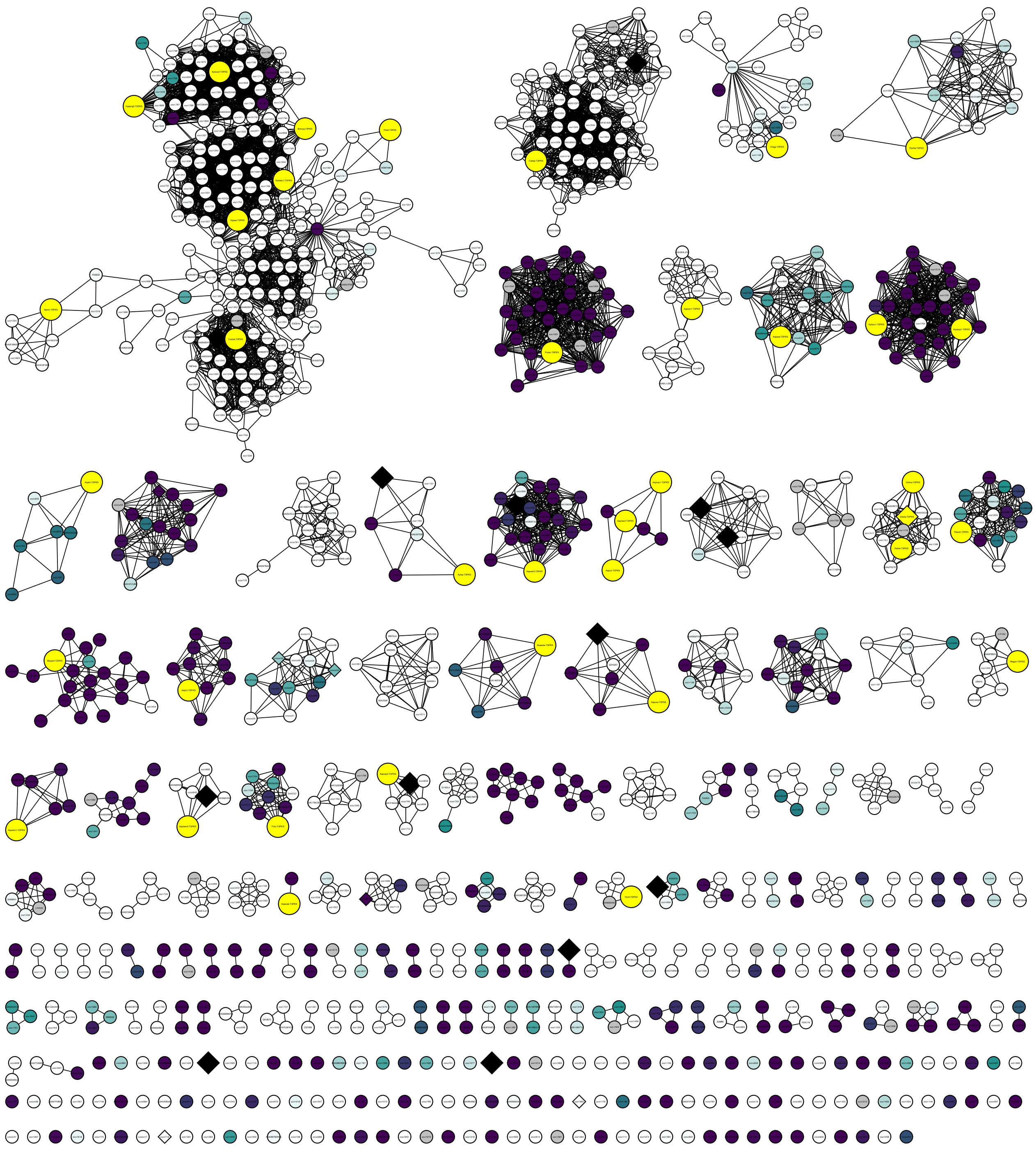

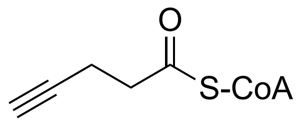
4-pentynoyl-CoA, **13**

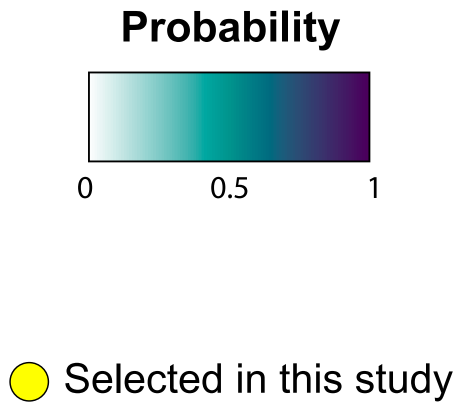

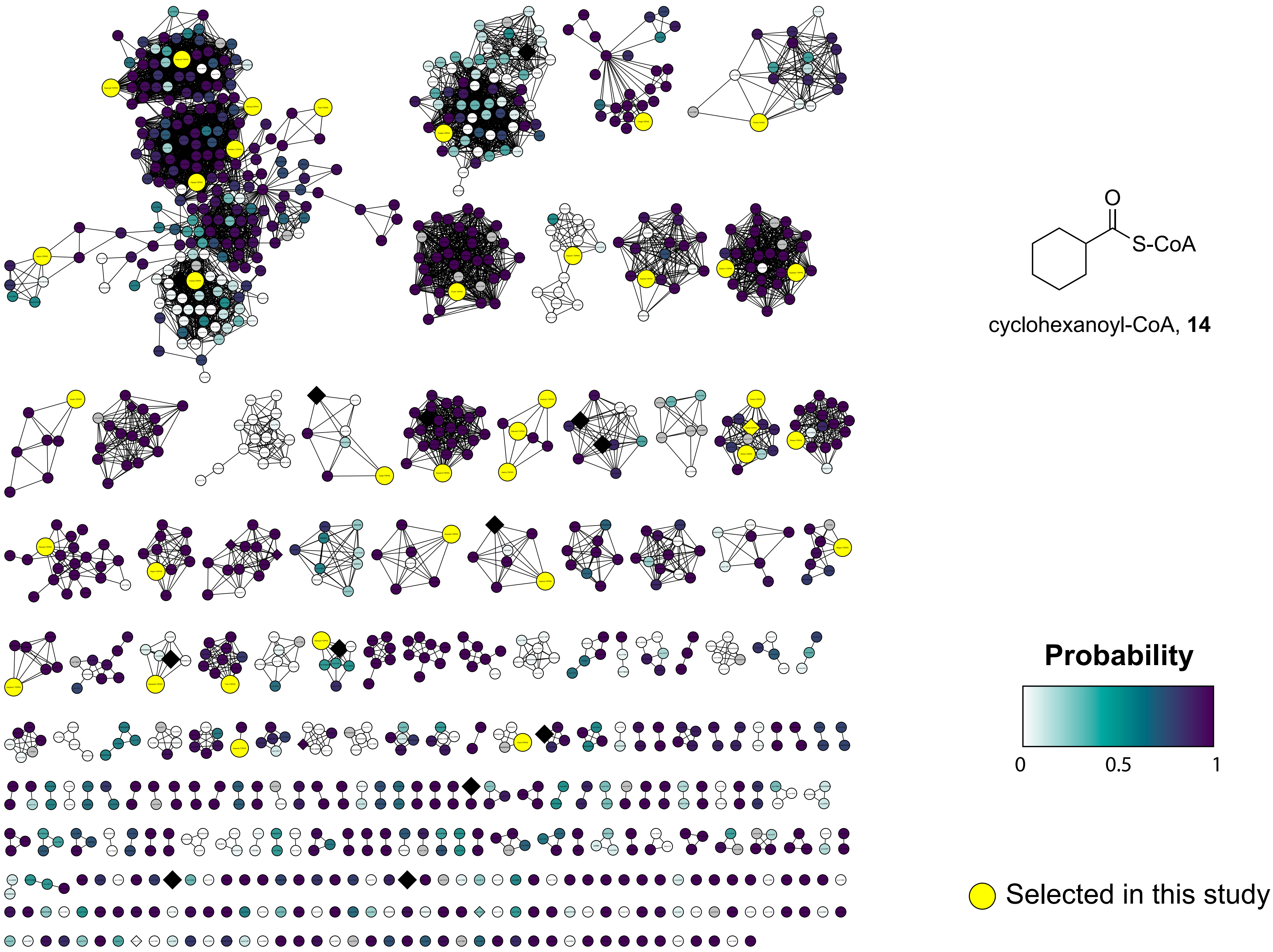
O
S-CoA
cyclohexanoyl-CoA, **14**
Probability
0
0.5
1
Selected in this study

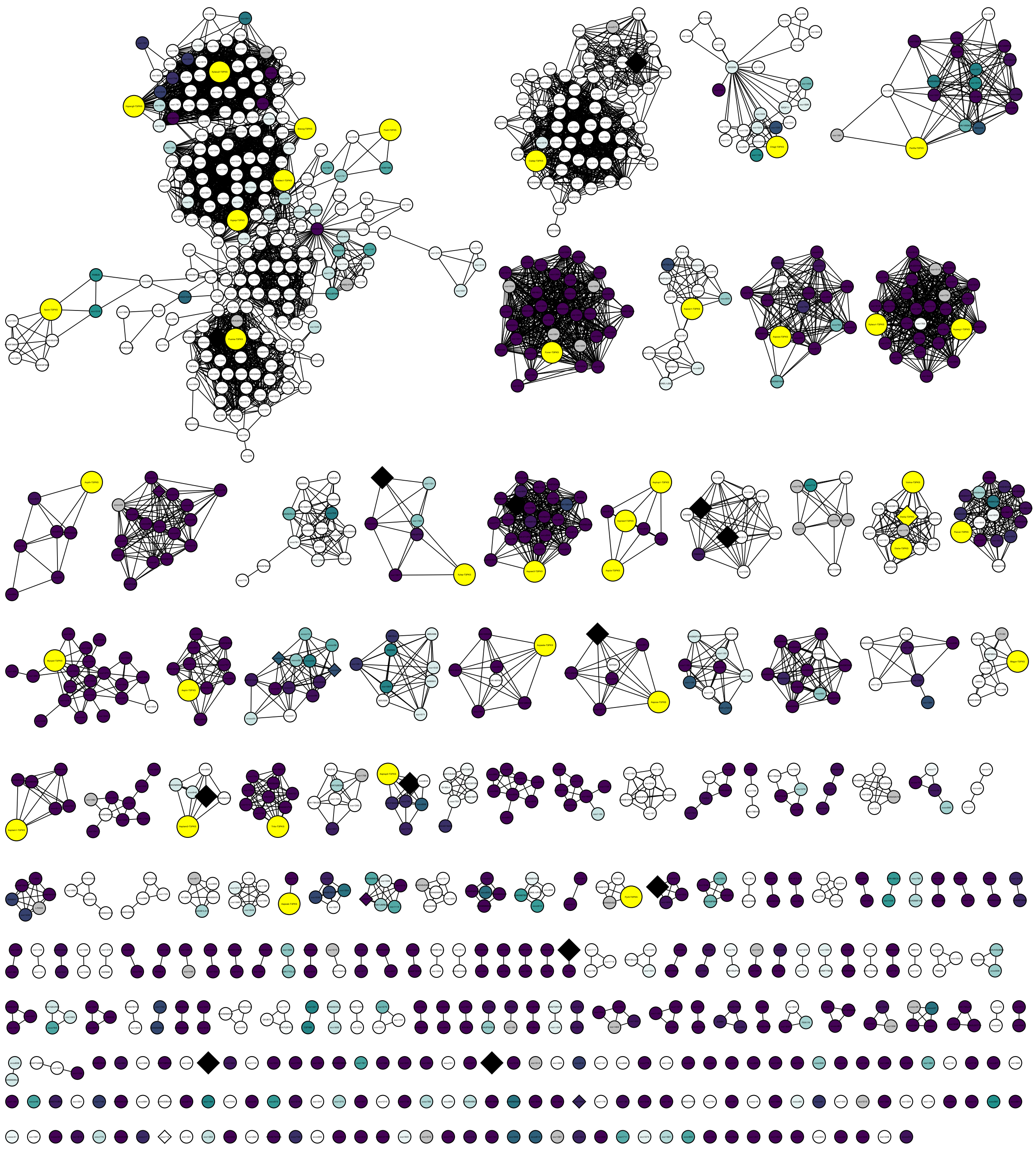

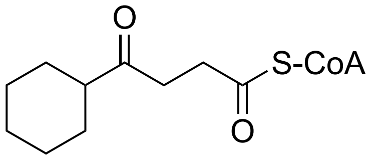

4-cyclohexyl-4-oxobutanoyl-CoA, **15**

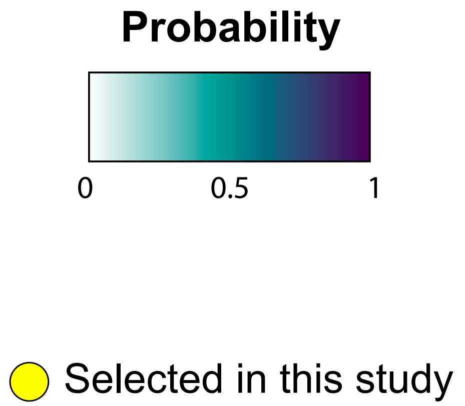

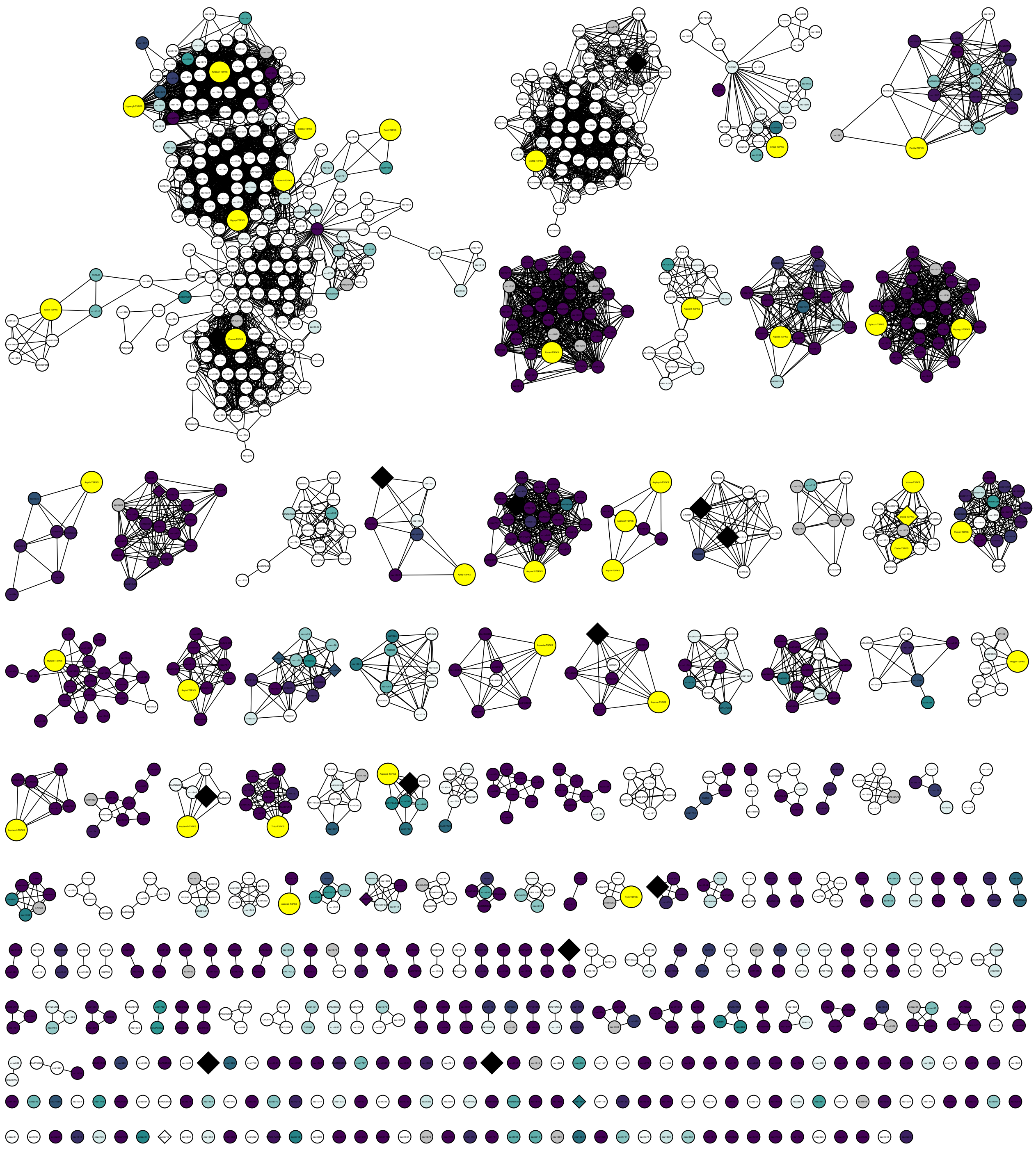

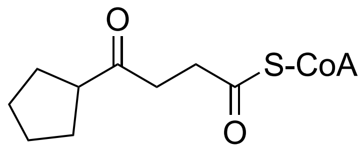

4-cyclopentyl-4-oxobutanoyl-CoA, **16**

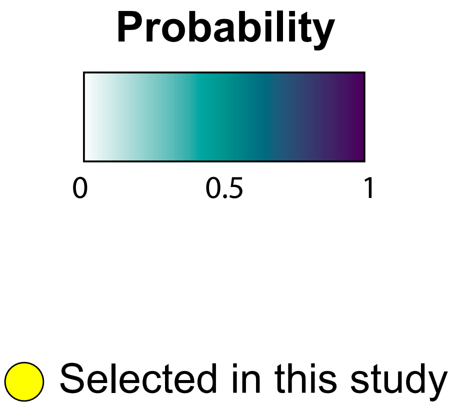

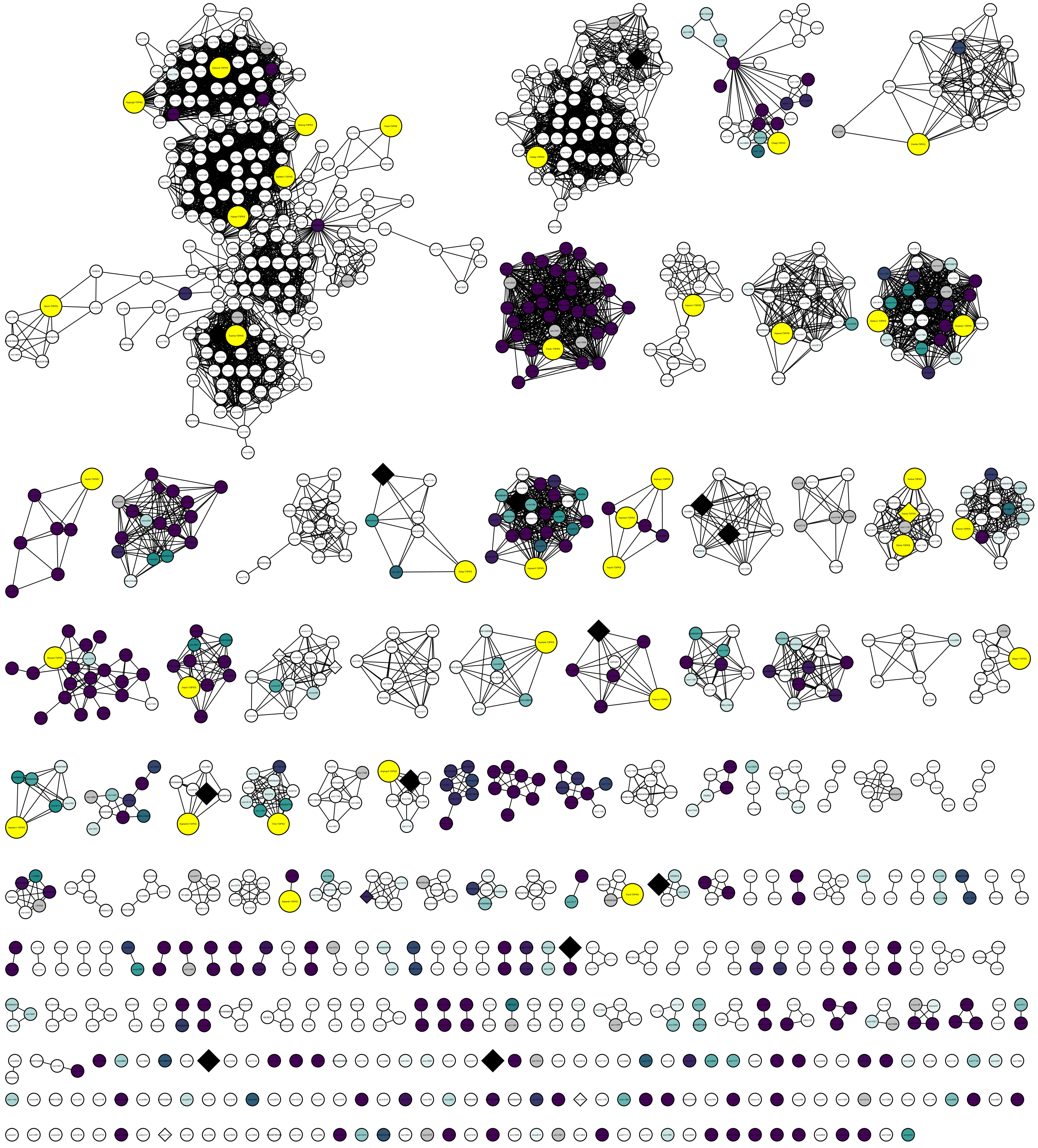

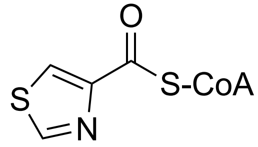

thiazole-4-carboxylyl-CoA, **17**

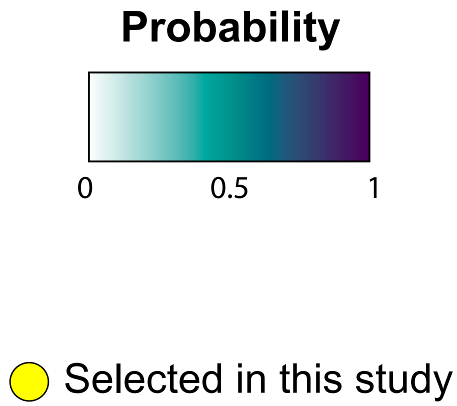

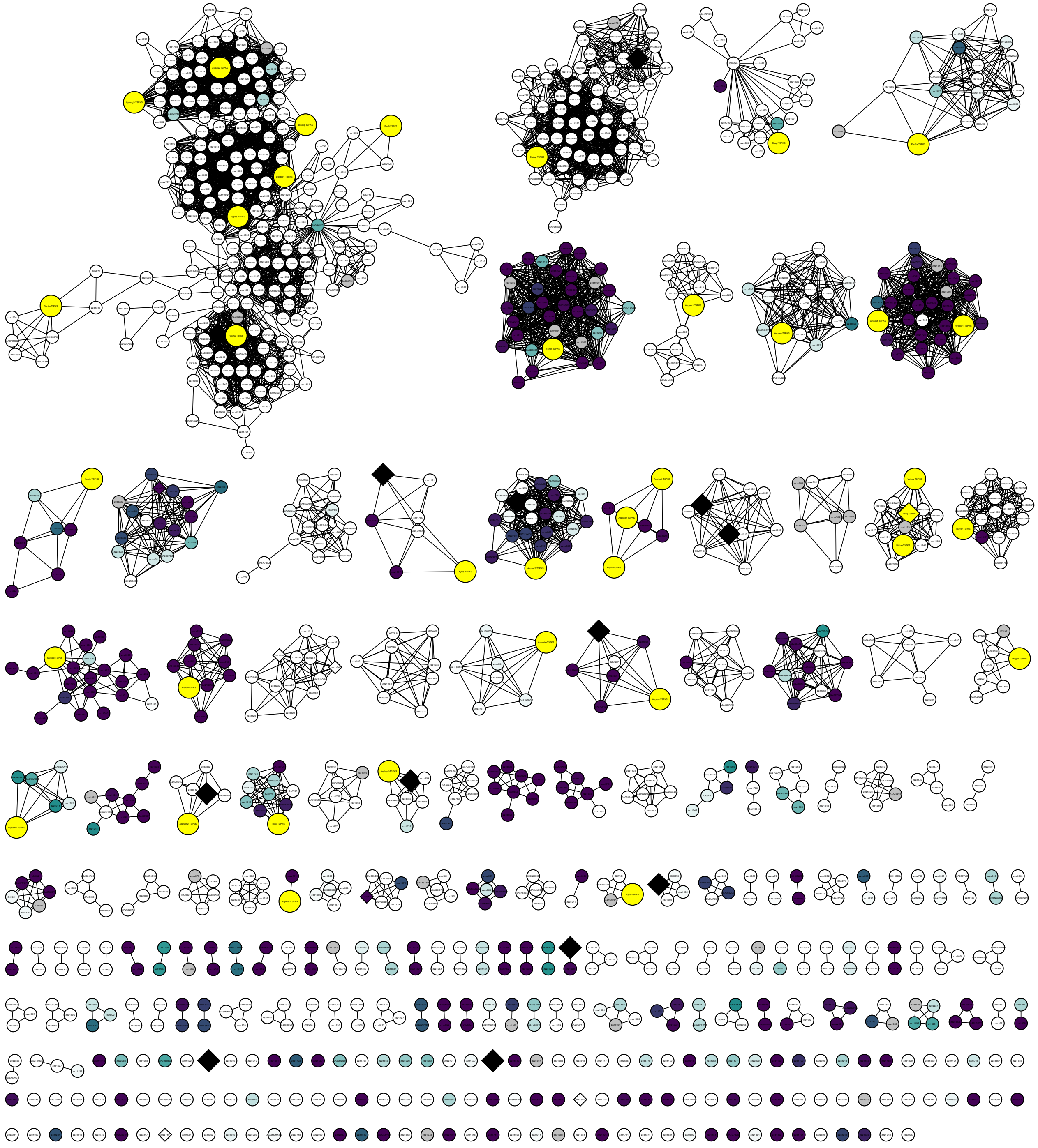

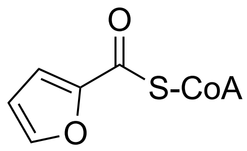

furoyl-CoA, **18**

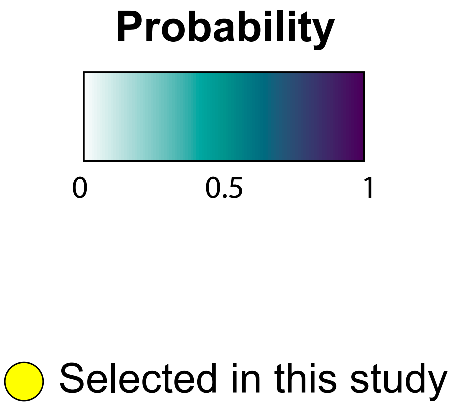

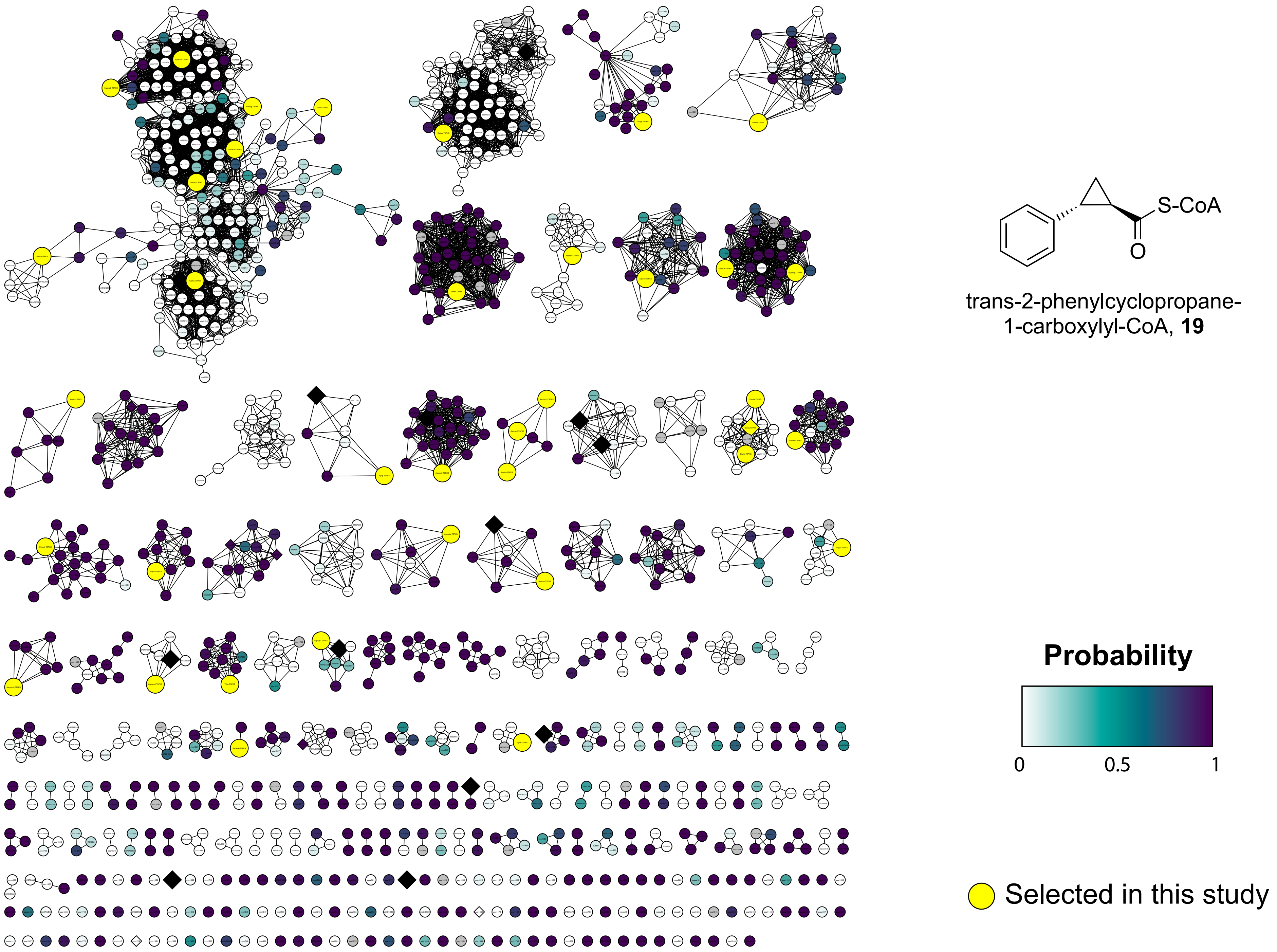
S-CoA
O
trans-2-phenylcyclopropane-1-carboxylyl-CoA, 19
Probability
0
0.5
1
Selected in this study

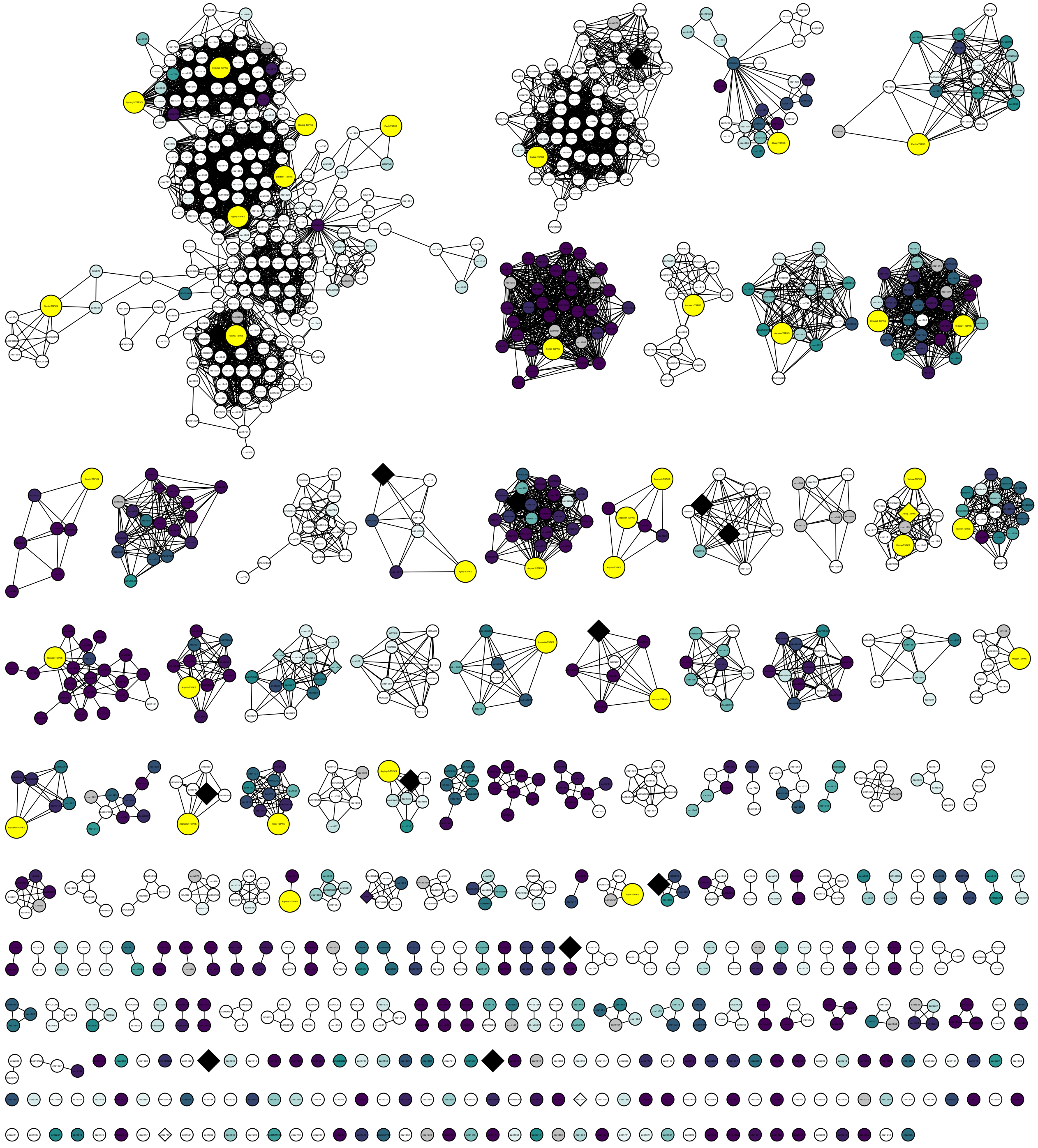

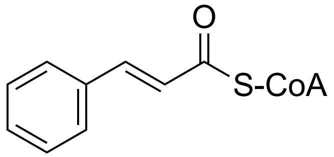

cinnamoyl-CoA, **20**

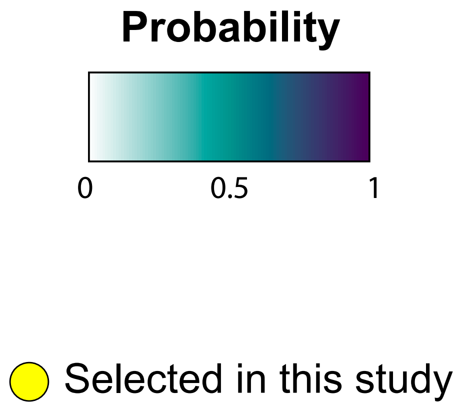

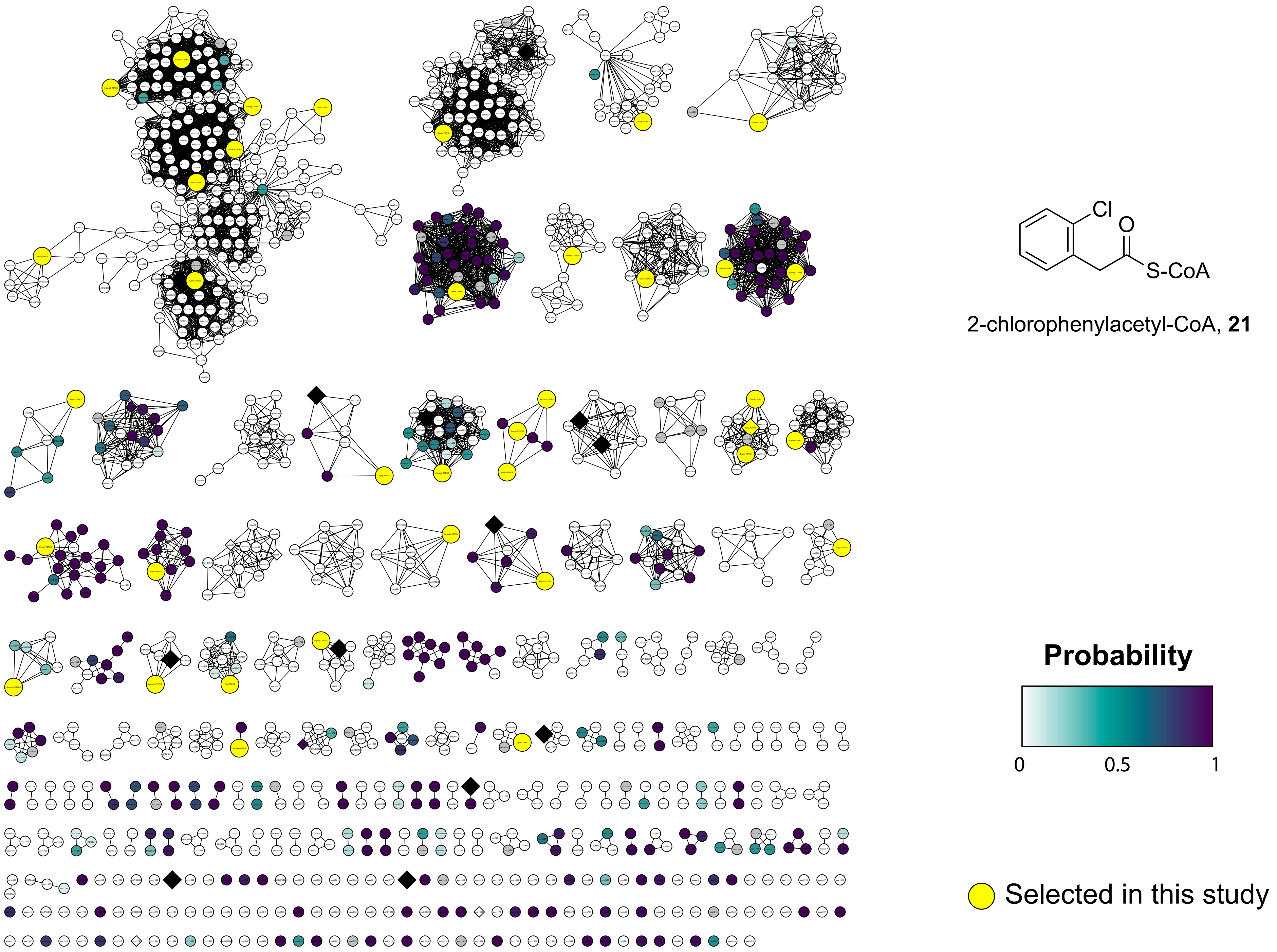
Cl
O
S-CoA
2-chlorophenylacetyl-CoA, 21
Probability
0
0.5
1
Selected in this study

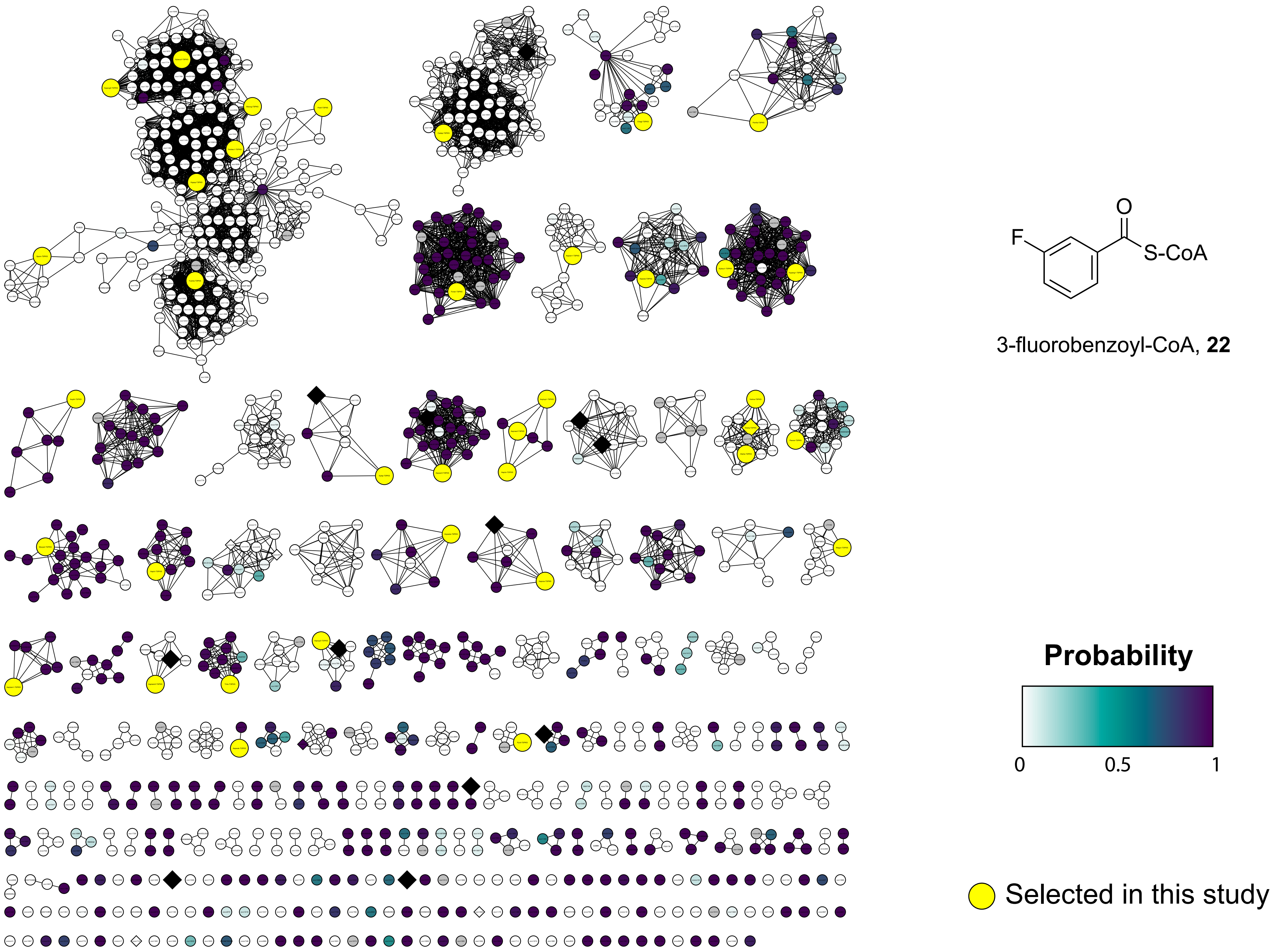

O
F
S-CoA
3-fluorobenzoyl-CoA, **22**
Probability
0
0.5
1
Selected in this study

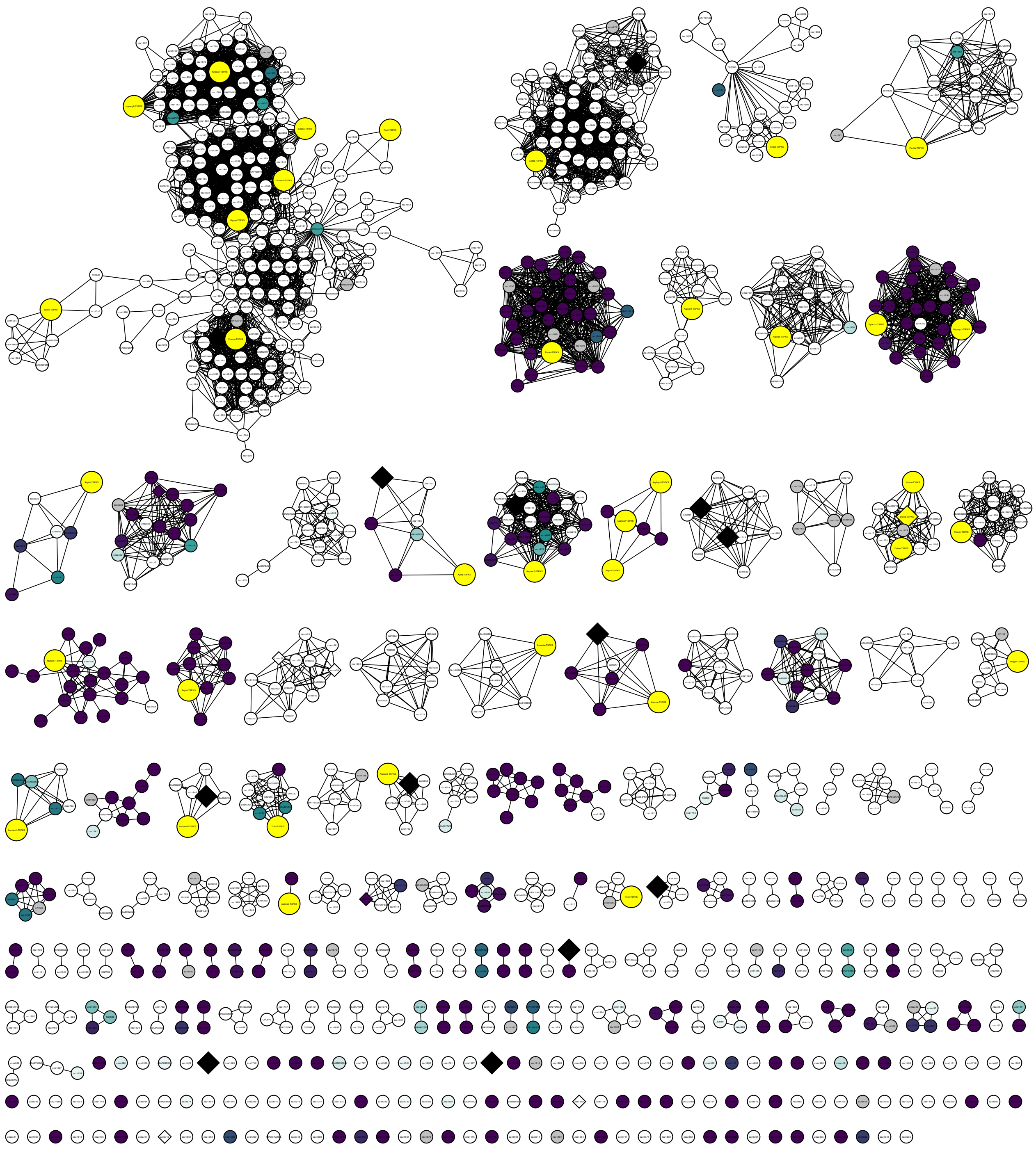

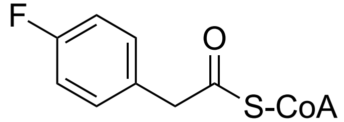

4-fluorophenylacetyl-CoA, **23**

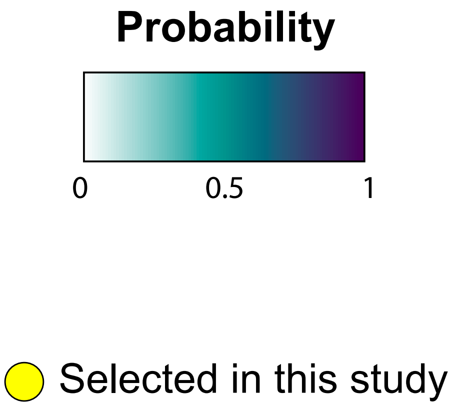

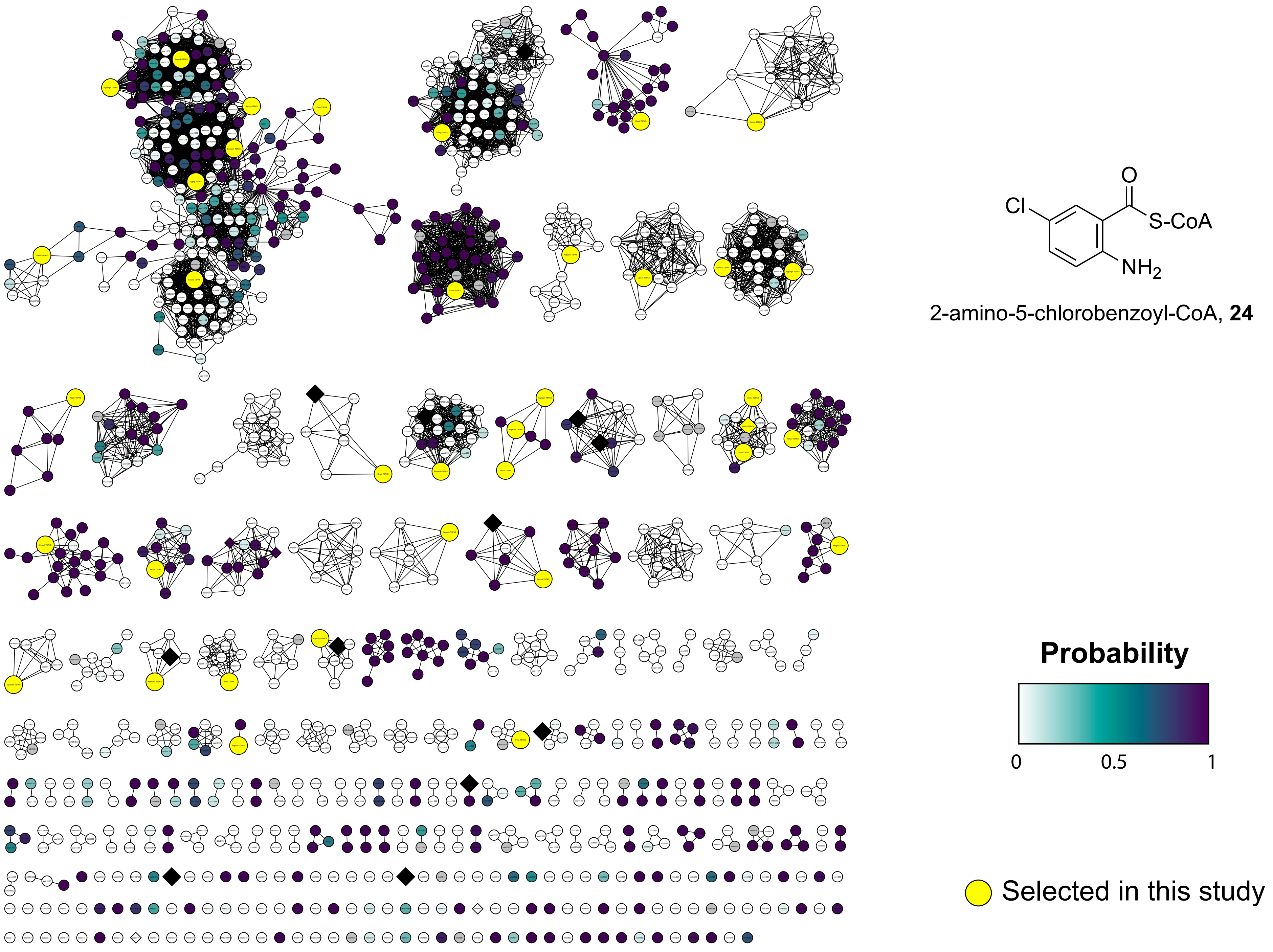

O
Cl
S-CoA
NH2
2-amino-5-chlorobenzoyl-CoA, 24
Probability
0
0.5
1
Selected in this study

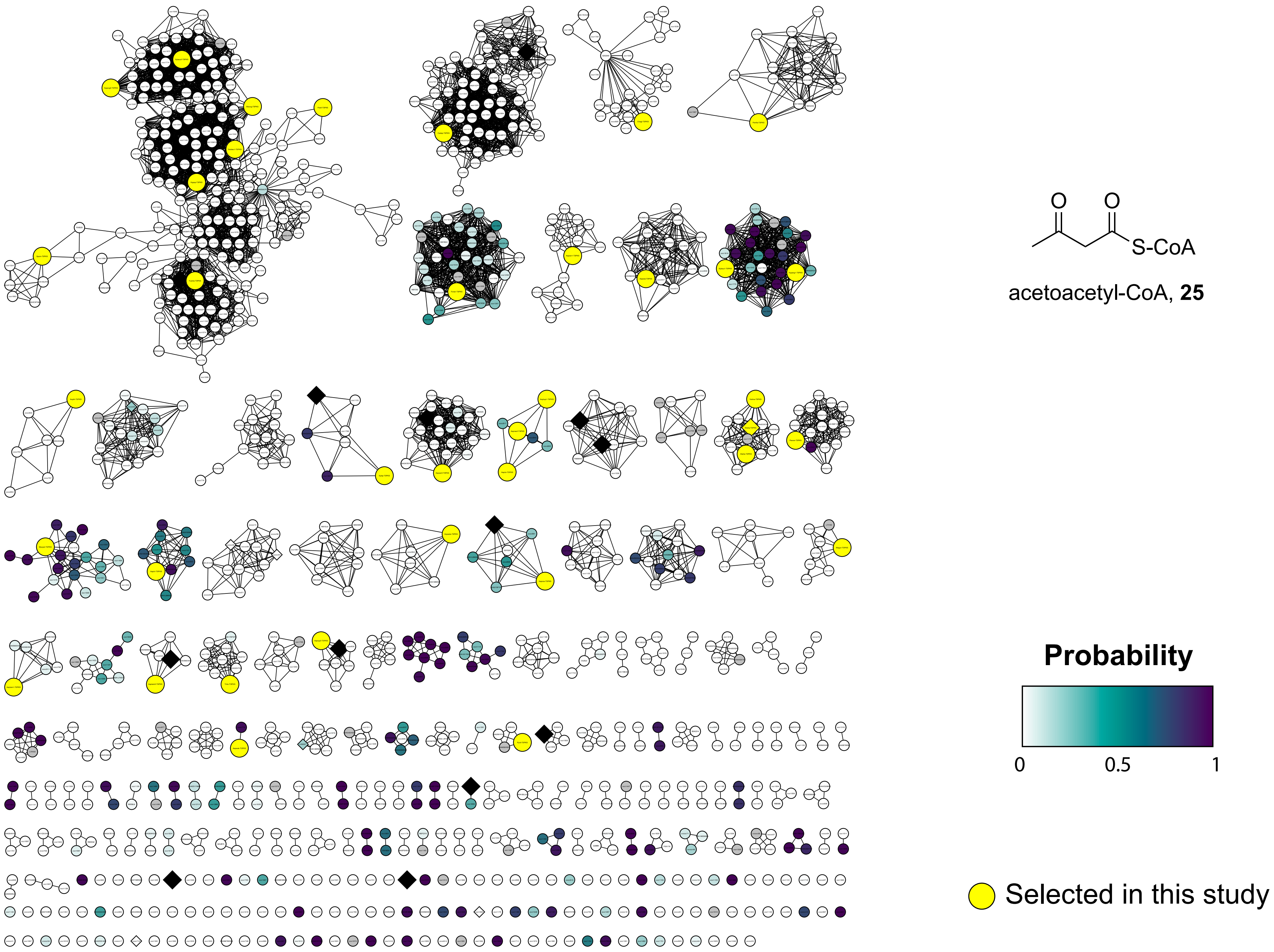
acetoacetyl-CoA, 25
Probability
0
0.5
1
Selected in this study

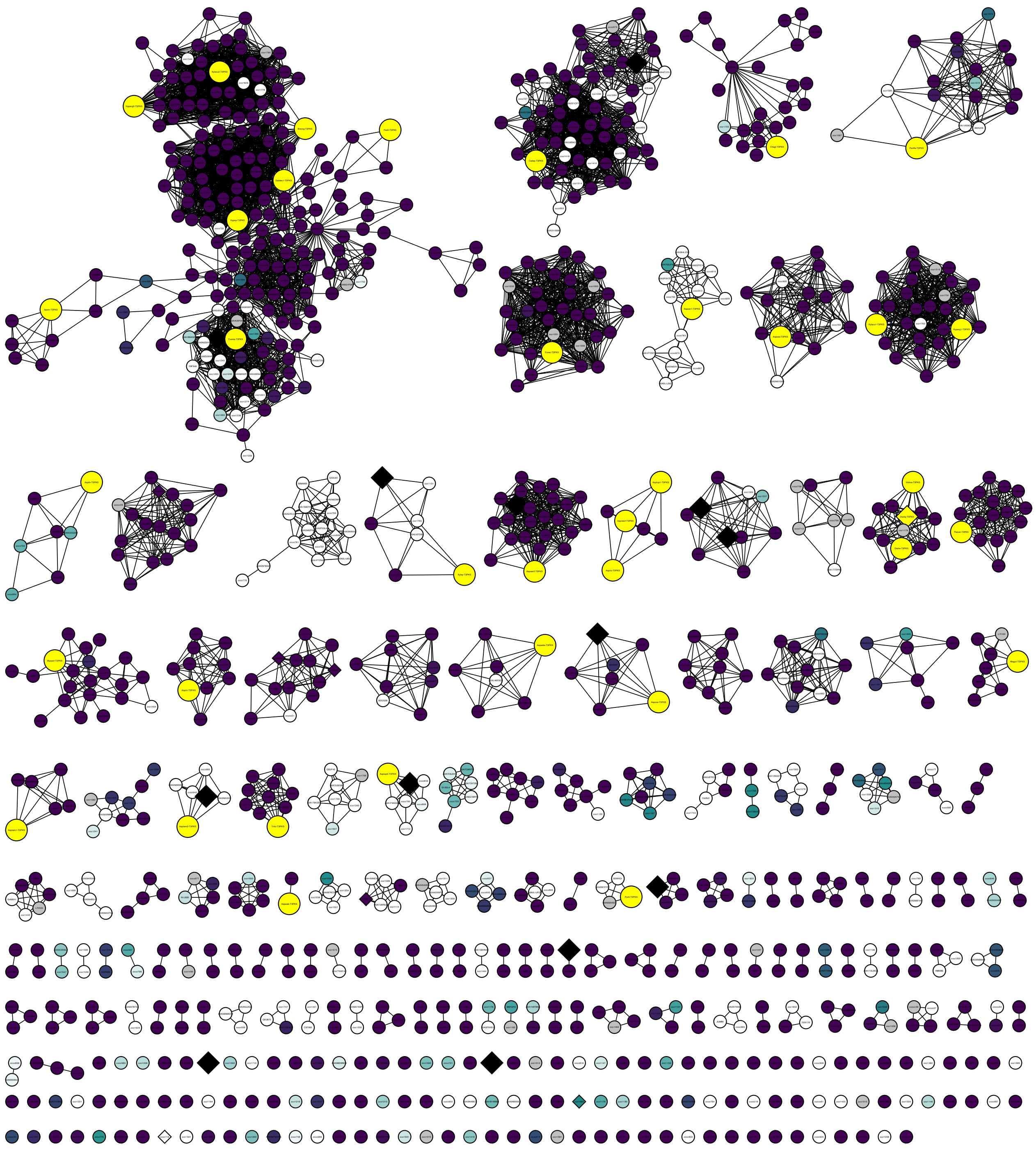

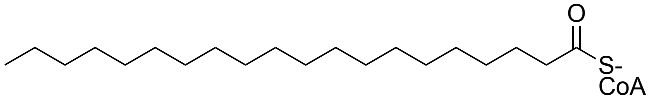

arachidoyl-CoA, **26**

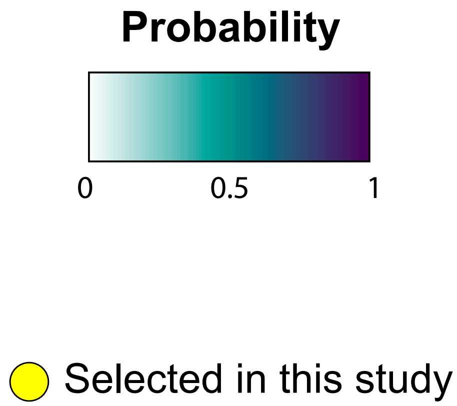

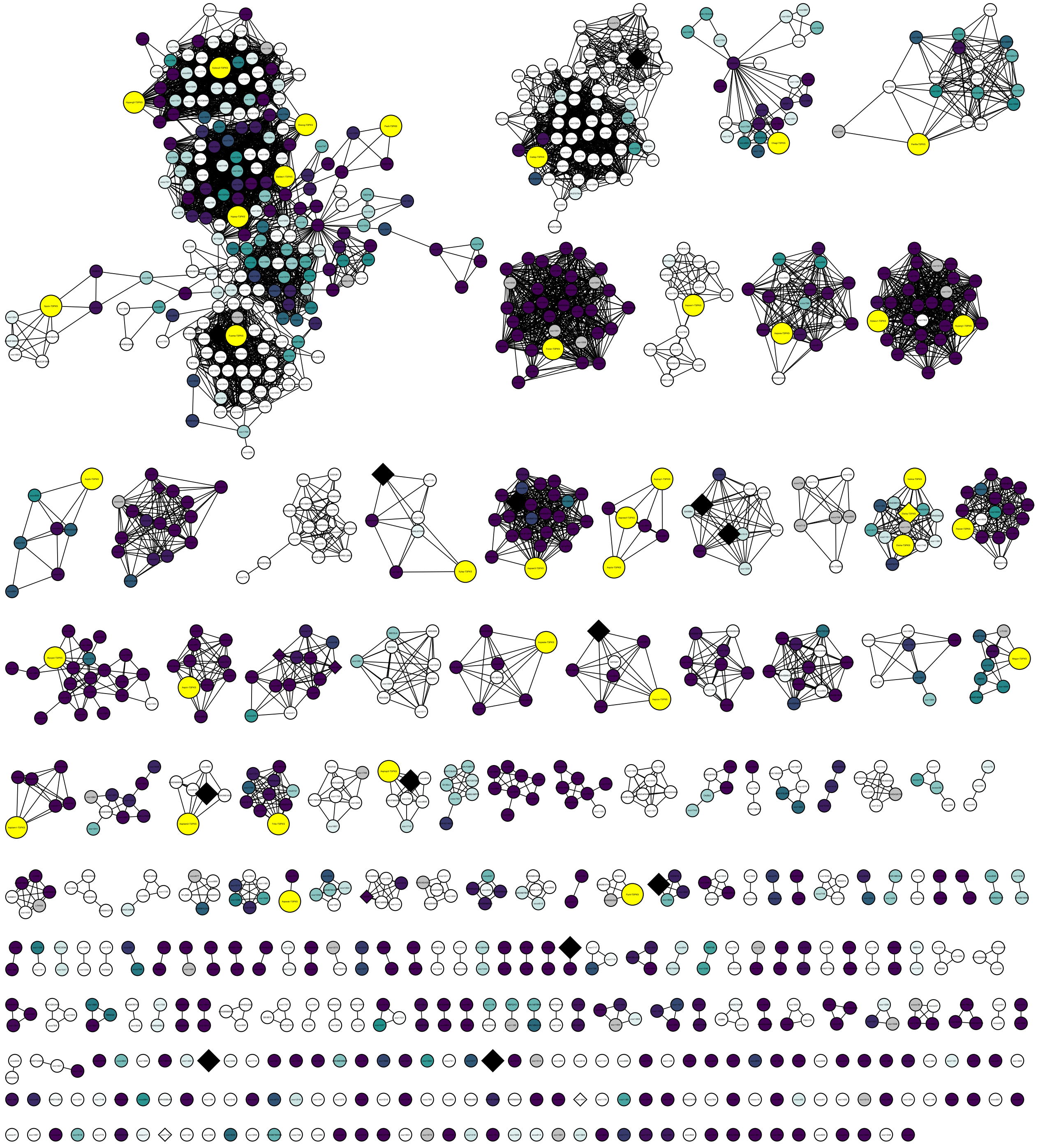

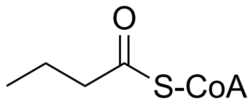

butyryl-CoA, **27**

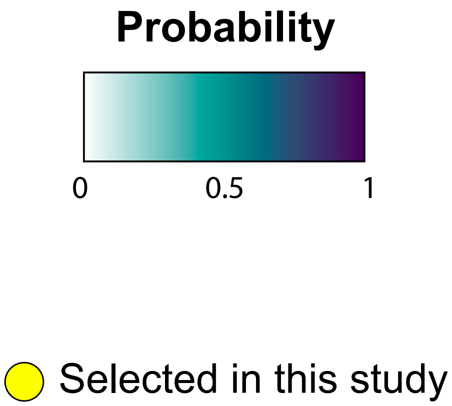

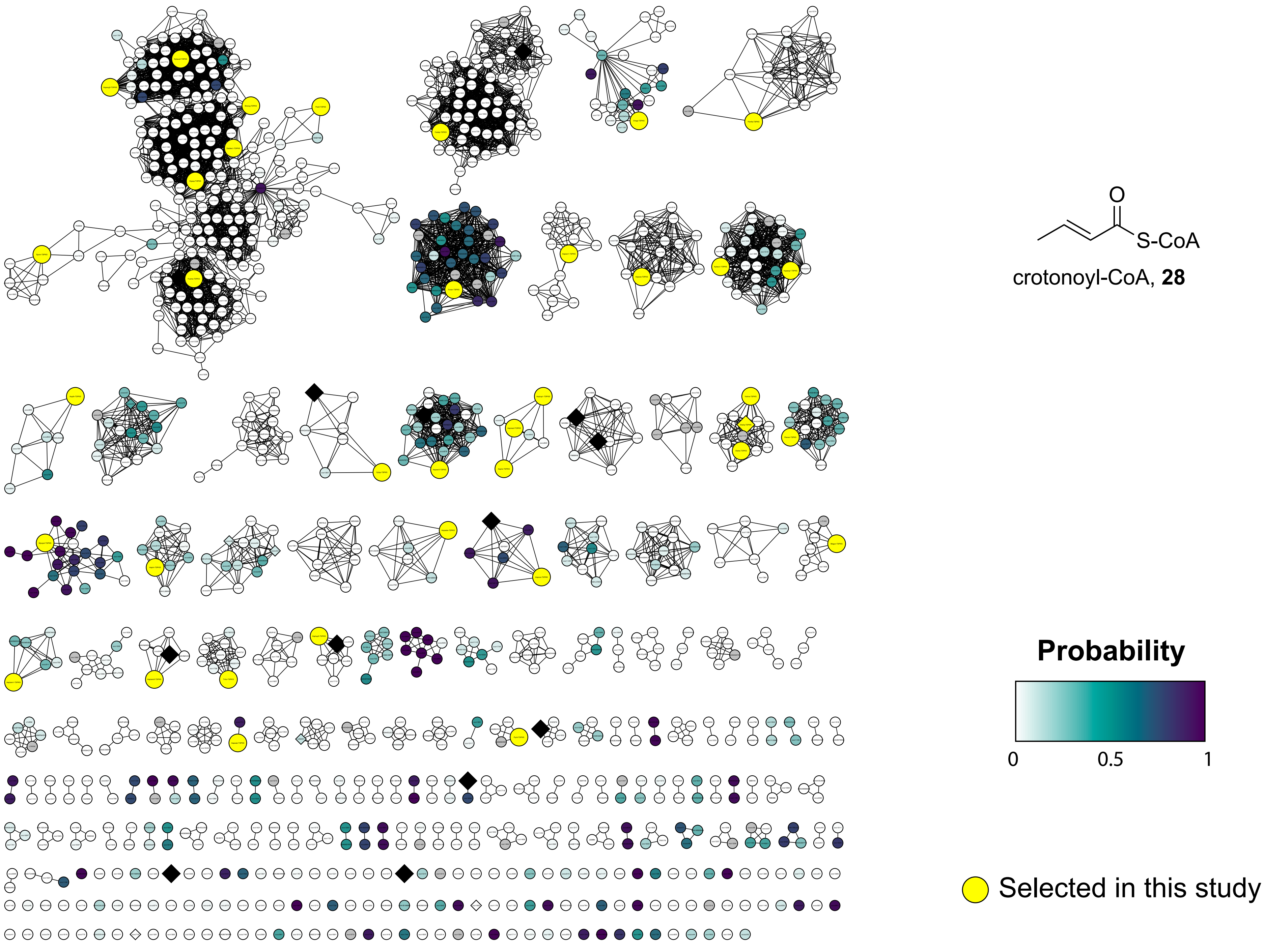
O
S-CoA
crotonoyl-CoA, **28**
Probability
0
0.5
1
Selected in this study

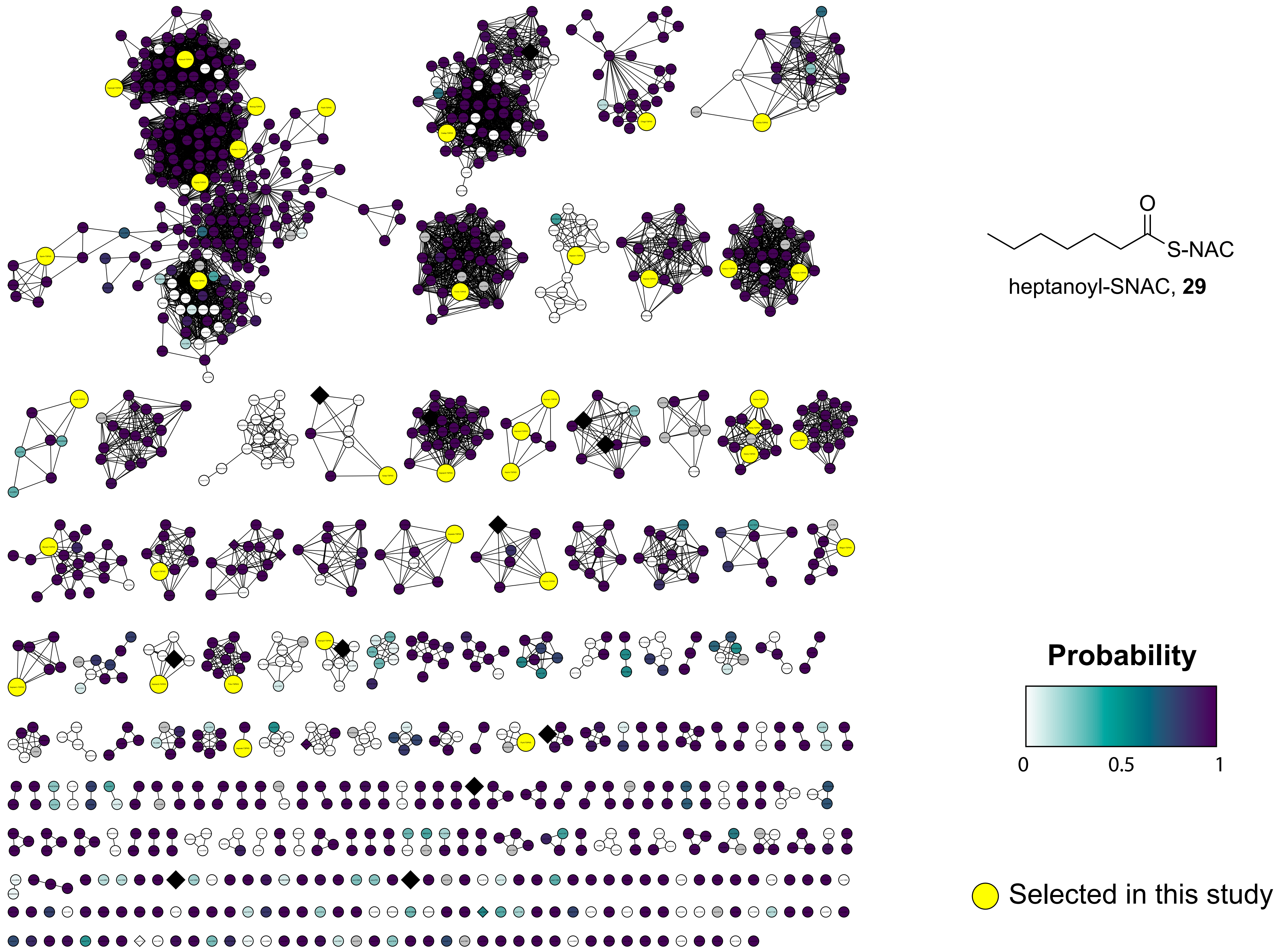
O
S-NAC
heptanoyl-SNAC, 29
Probability
0
0.5
1
Selected in this study

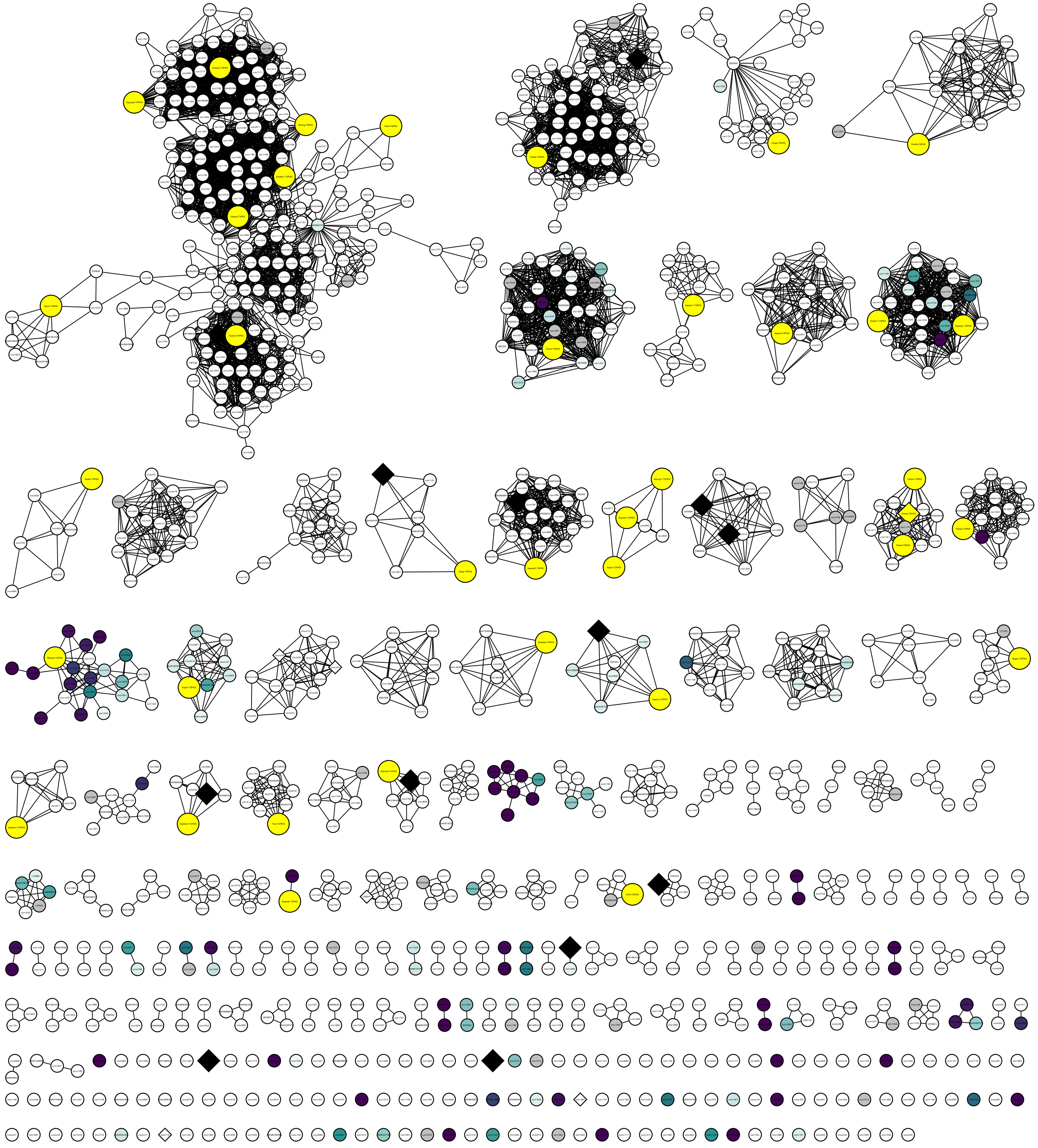

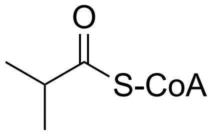

isobutyryl-CoA, **30**

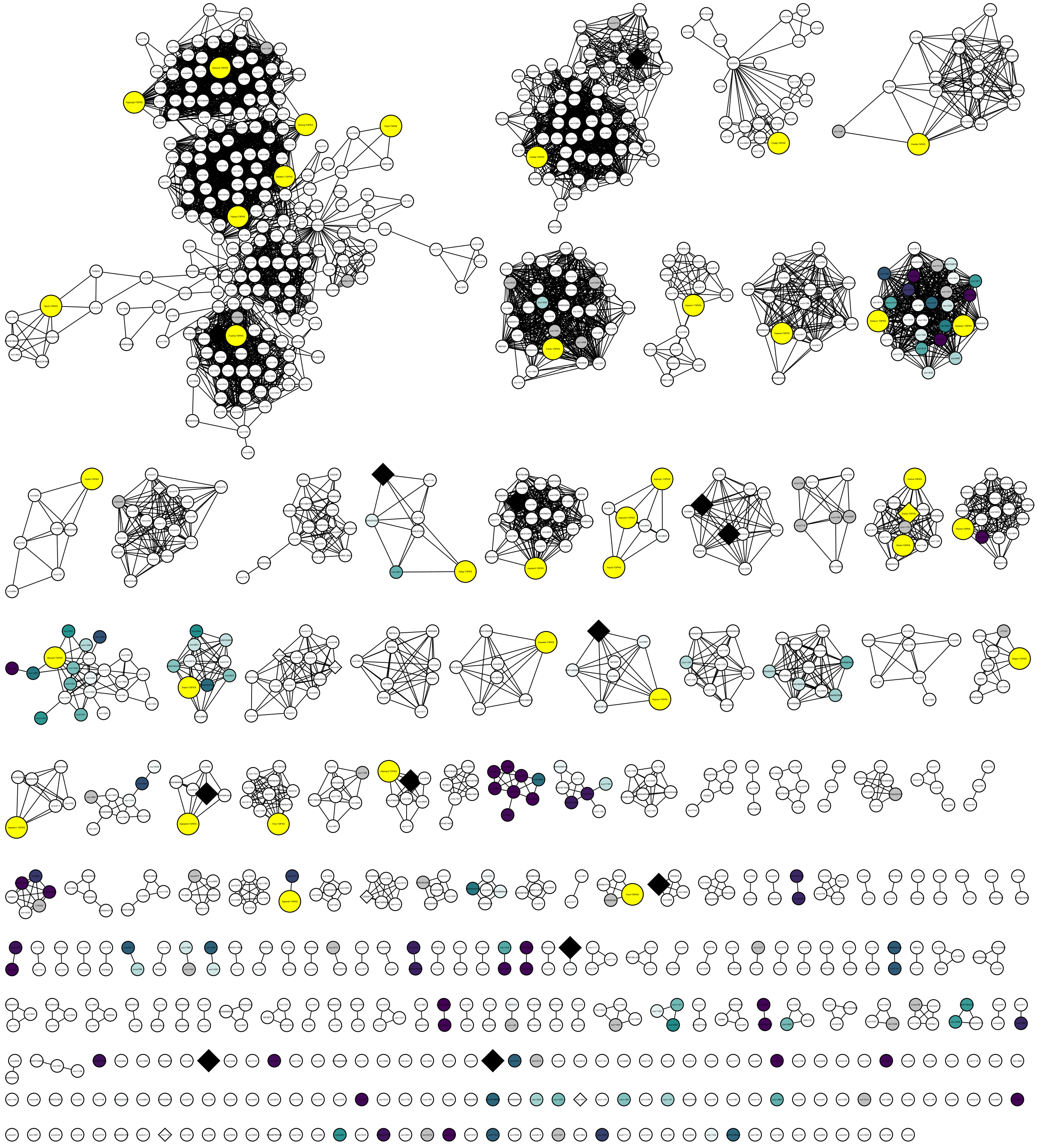

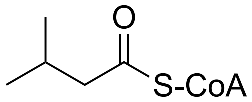

isovaleryl-CoA, **31**

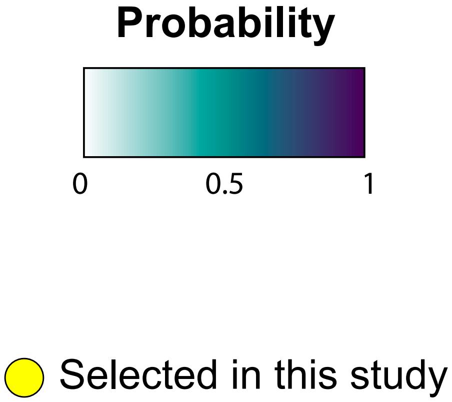

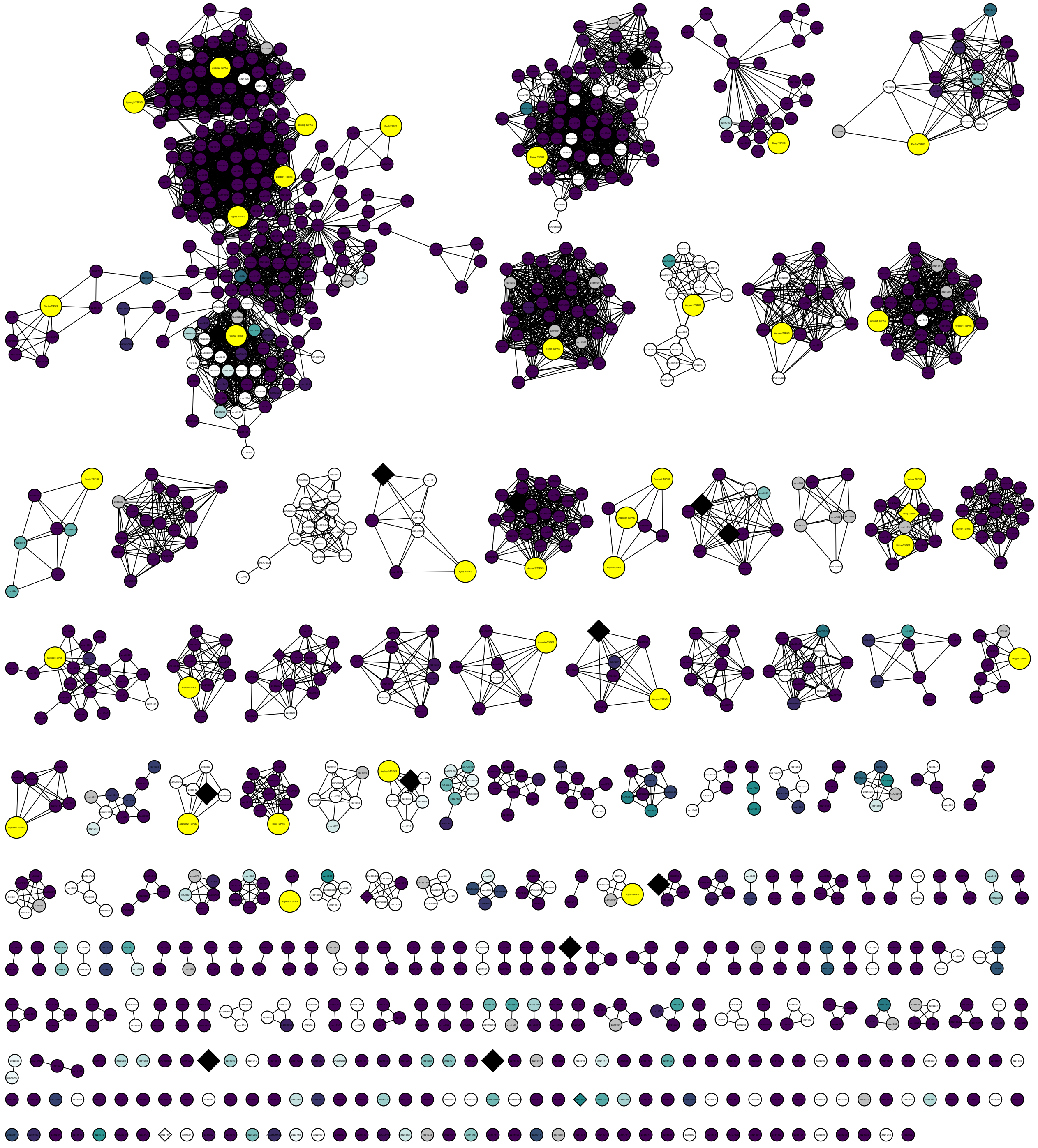

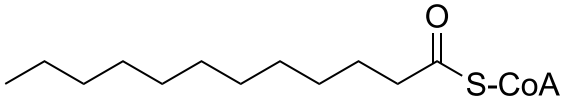

lauroyl-CoA, **32**

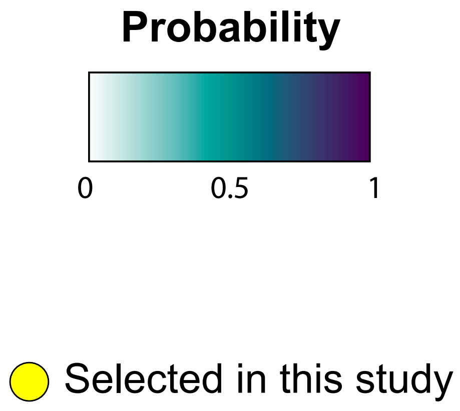

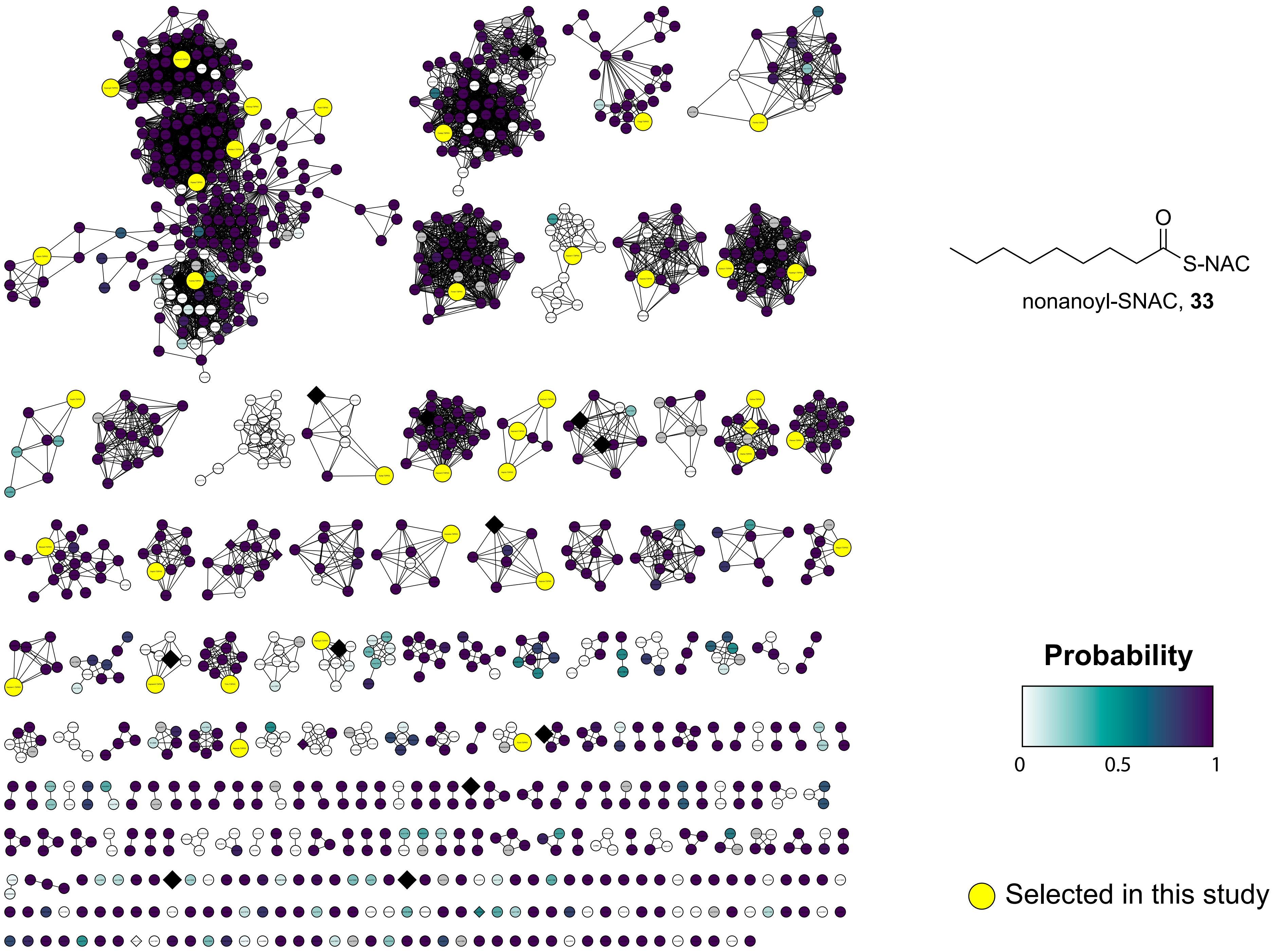
O
S-NAC
nonanoyl-SNAC, 33
Probability
0
0.5
1
Selected in this study

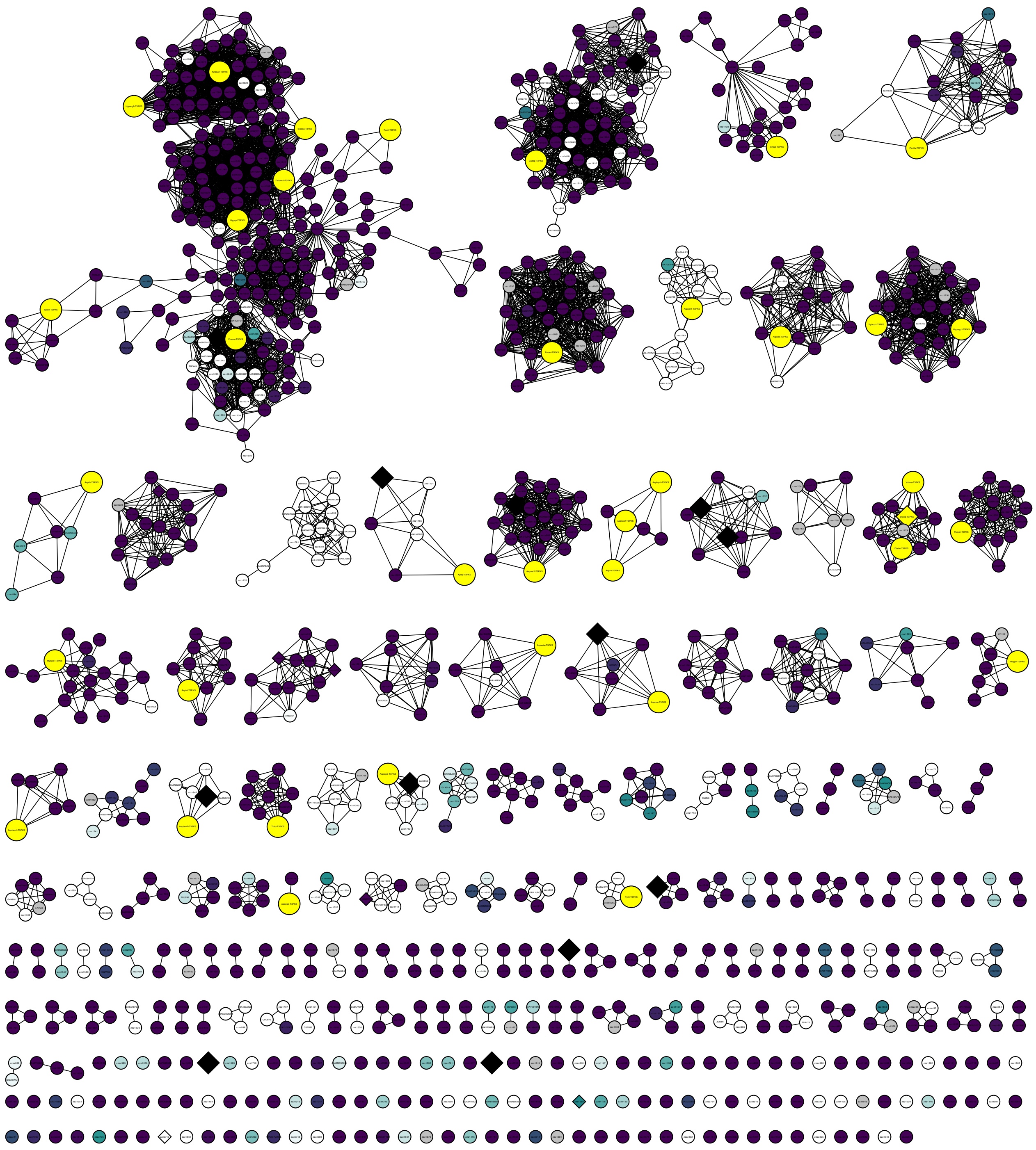

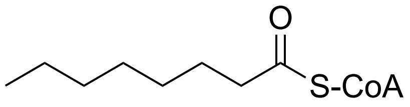

octanoyl-CoA, **34**

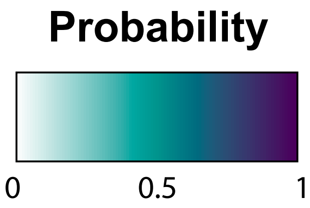

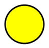
Selected in this study

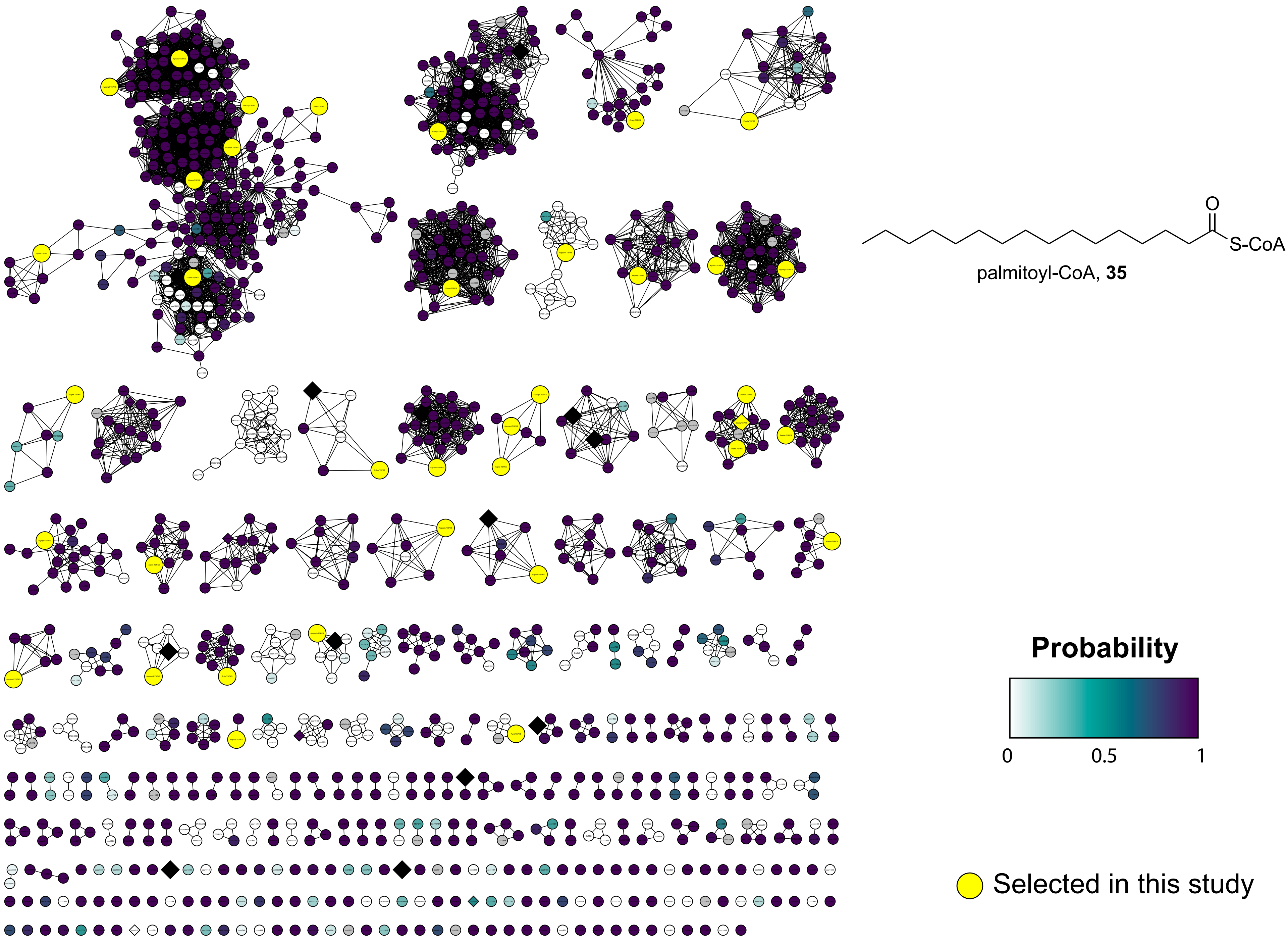
O
S-CoA
palmitoyl-CoA, **35**
Probability
0
0.5
1
Selected in this study

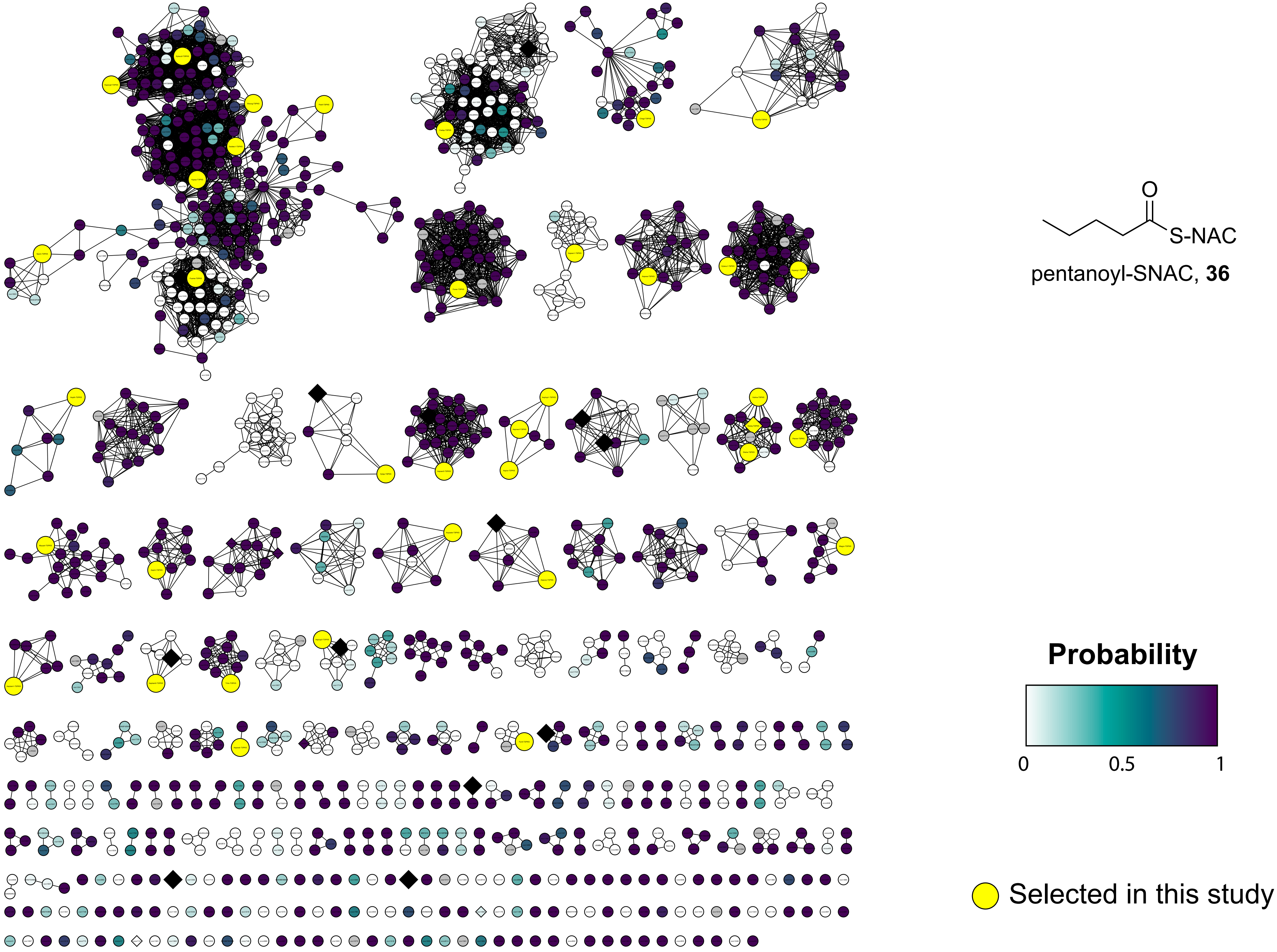
O
S-NAC
pentanoyl-SNAC, 36
Probability
0
0.5
1
Selected in this study

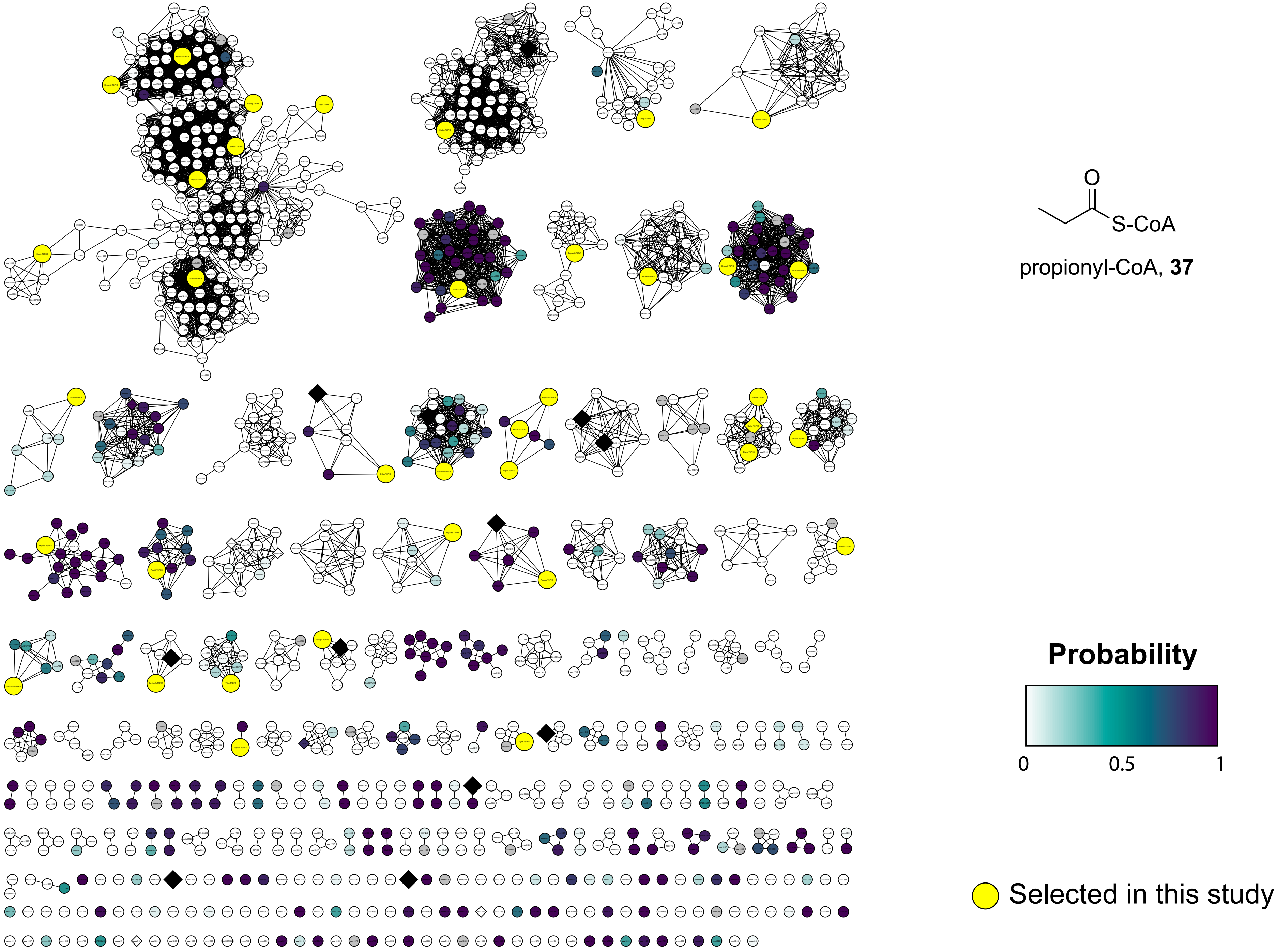
O
S-CoA
propionyl-CoA, **37**
Probability
0
0.5
1
Selected in this study

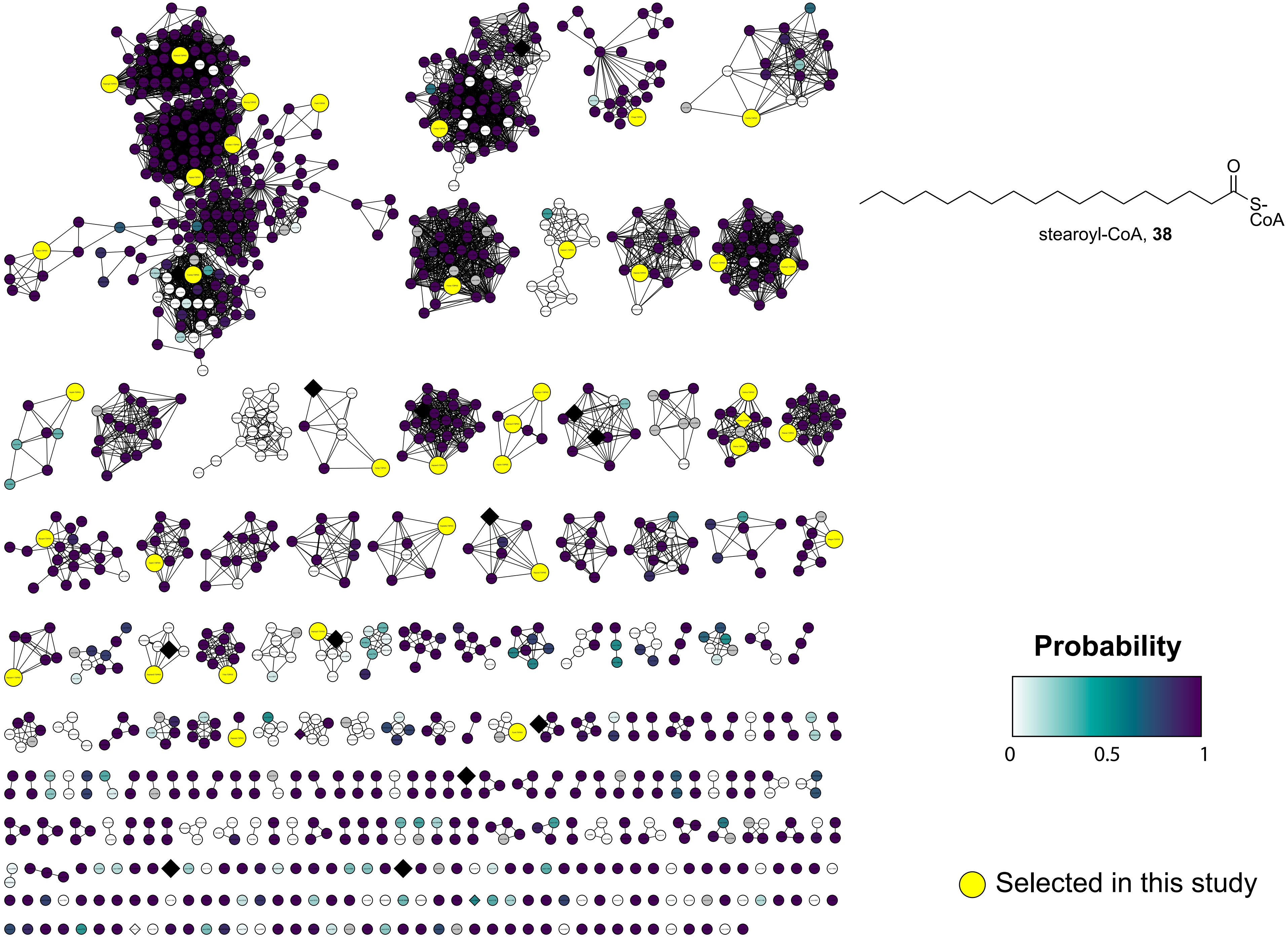
O
S-
CoA
stearoyl-CoA, **38**
Probability
0
0.5
1
Selected in this study
