# Supplementary material for: Unravelling the Functional Diversity of Type III Polyketide Synthases in Fungi
Source: Angew Chem Int Ed Engl. 2025 Sep 4;64(44):e202514786. doi: 10.1002/anie.202514786 (PMC12559476; doi:10.1002/anie.202514786)
Supplement: Supplementary file 2 — Supporting Information [file ANIE-64-e202514786-s001.zip › Supporting_Files/Supporting_File_8.pdf]

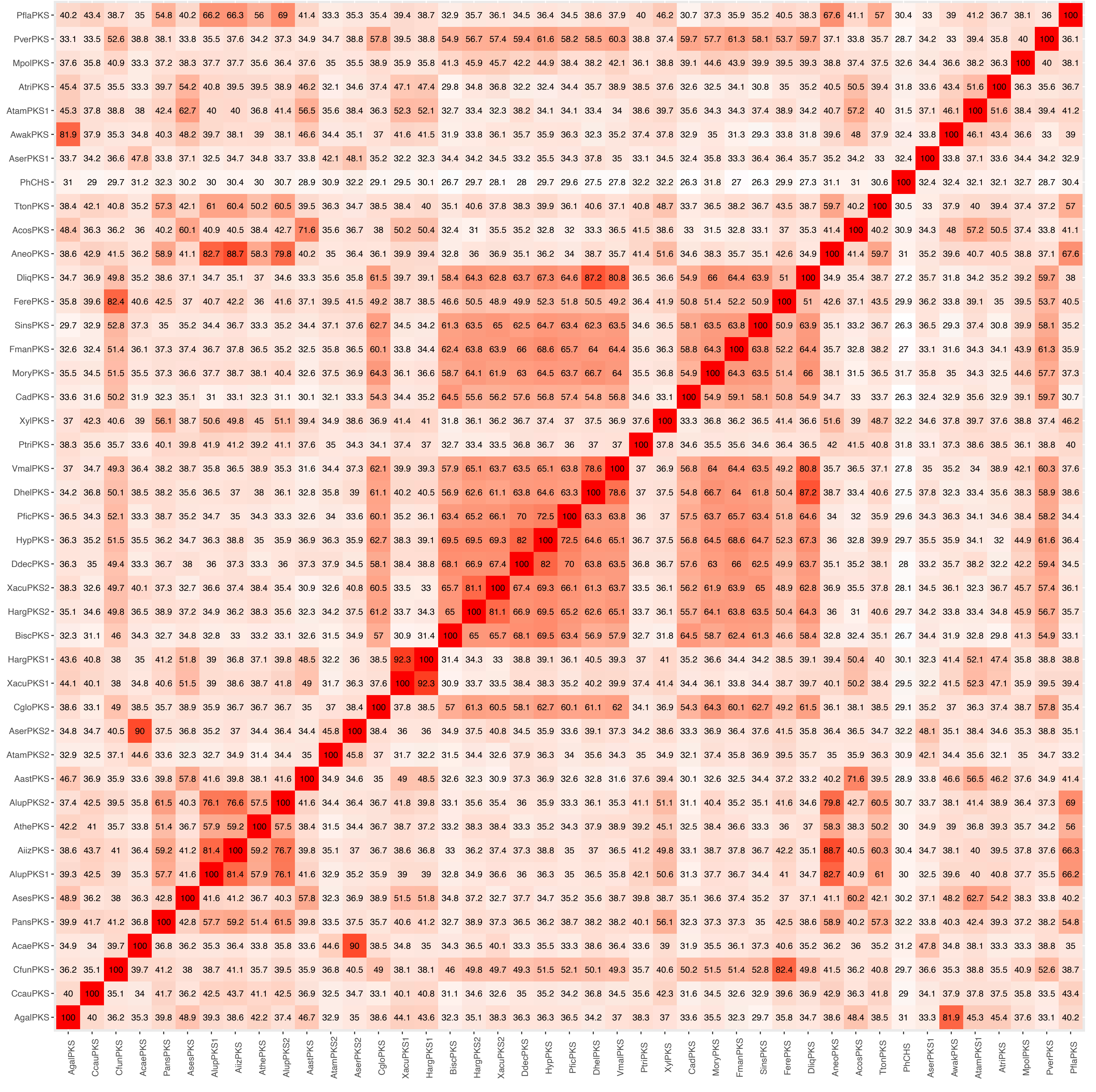
PflaPKS
PverPKS
MpolPKS
AtriPKS
AtamPKS1
AwakPKS
AserPKS1
PhCHS
TtonPKS
AcosPKS
AneoPKS
DliqPKS
FerePKS
SinsPKS
FmanPKS
MoryPKS
CadPKS
XylPKS
PtriPKS
VmalPKS
DhelPKS
PficPKS
HypPKS
DdecPKS
XacuPKS2
HargPKS2
BiscPKS
HargPKS1
XacuPKS1
CgloPKS
AserPKS2
AtamPKS2
AastPKS
AlupPKS2
AthePKS
AiizPKS
AlupPKS1
AsesPKS
PansPKS
AcaePKS
CfunPKS
CcauPKS
AgalPKS
AgalPKS
CcauPKS
CfunPKS
AcaePKS
PansPKS
AsesPKS
AlupPKS1
AiizPKS
AthePKS
AlupPKS2
AastPKS
AtamPKS2
AserPKS2
CgloPKS
XacuPKS1
HargPKS1
BiscPKS
HargPKS2
XacuPKS2
DdecPKS
HypPKS
PficPKS
DhelPKS
VmalPKS
PtriPKS
XylPKS
CadPKS
MoryPKS
FmanPKS
SinsPKS
FerePKS
DliqPKS
AneoPKS
AcosPKS
TtonPKS
PhCHS
AserPKS1
AwakPKS
AtamPKS1
AtriPKS
MpolPKS
PverPKS
PflaPKS
